# The Concise Guide to PHARMACOLOGY 2023/24: G protein-coupled receptors

**DOI:** 10.1111/bph.16177

**Published:** 2023-10

**Authors:** Stephen P. H. Alexander, Arthur Christopoulos, Anthony P. Davenport, Eamonn Kelly, Alistair A. Mathie, John A. Peters, Emma L. Veale, Jane F. Armstrong, Elena Faccenda, Simon D. Harding, Jamie A. Davies, Maria Pia Abbracchio, George Abraham, Alexander Agoulnik, Wayne Alexander, Khaled Al-hosaini, Magnus Bäck, Jillian G. Baker, Nicholas M. Barnes, Ross Bathgate, Jean-Martin Beaulieu, Annette G. Beck-Sickinger, Maik Behrens, Kenneth E. Bernstein, Bernhard Bettler, Nigel J. M. Birdsall, Victoria Blaho, Francois Boulay, Corinne Bousquet, Hans Bräuner-Osborne, Geoffrey Burnstock, Girolamo Caló, Justo P. Castaño, Kevin J. Catt, Stefania Ceruti, Paul Chazot, Nan Chiang, Bice Chini, Jerold Chun, Antonia Cianciulli, Olivier Civelli, Lucie H. Clapp, Réjean Couture, Helen M. Cox, Zsolt Csaba, Claes Dahlgren, Gordon Dent, Steven D. Douglas, Pascal Dournaud, Satoru Eguchi, Emanuel Escher, Edward J. Filardo, Tung Fong, Marta Fumagalli, Raul R. Gainetdinov, Michael L. Garelja, Marc de Gasparo, Craig Gerard, Marvin Gershengorn, Fernand Gobeil, Theodore L. Goodfriend, Cyril Goudet, Lukas Grätz, Karen J. Gregory, Andrew L. Gundlach, Jörg Hamann, Julien Hanson, Richard L. Hauger, Debbie L. Hay, Akos Heinemann, Deron Herr, Morley D. Hollenberg, Nicholas D. Holliday, Mastgugu Horiuchi, Daniel Hoyer, László Hunyady, Ahsan Husain, Adriaan P. IJzerman, Tadashi Inagami, Kenneth A. Jacobson, Robert T. Jensen, Ralf Jockers, Deepa Jonnalagadda, Sadashiva Karnik, Klemens Kaupmann, Jacqueline Kemp, Charles Kennedy, Yasuyuki Kihara, Takio Kitazawa, Pawel Kozielewicz, Hans-Jürgen Kreienkamp, Jyrki P. Kukkonen, Tobias Langenhan, Dan Larhammar, Katie Leach, Davide Lecca, John D. Lee, Susan E. Leeman, Jérôme Leprince, Xaria X. Li, Stephen J. Lolait, Amelie Lupp, Robyn Macrae, Janet Maguire, Davide Malfacini, Jean Mazella, Craig A. McArdle, Shlomo Melmed, Martin C. Michel, Laurence J. Miller, Vincenzo Mitolo, Bernard Mouillac, Christa E. Müller, Philip M. Murphy, Jean-Louis Nahon, Tony Ngo, Xavier Norel, Duuamene Nyimanu, Anne-Marie O’Carroll, Stefan Offermanns, Maria Antonietta Panaro, Marc Parmentier, Roger G. Pertwee, Jean-Philippe Pin, Eric R. Prossnitz, Mark Quinn, Rithwik Ramachandran, Manisha Ray, Rainer K. Reinscheid, Philippe Rondard, G. Enrico Rovati, Chiara Ruzza, Gareth J. Sanger, Torsten Schöneberg, Gunnar Schulte, Stefan Schulz, Deborah L. Segaloff, Charles N. Serhan, Khuraijam Dhanachandra Singh, Craig M. Smith, Leigh A. Stoddart, Yukihiko Sugimoto, Roger Summers, Valerie P. Tan, David Thal, Walter (Wally) Thomas, Pieter B. M. W. M. Timmermans, Kalyan Tirupula, Lawrence Toll, Giovanni Tulipano, Hamiyet Unal, Thomas Unger, Celine Valant, Patrick Vanderheyden, David Vaudry, Hubert Vaudry, Jean-Pierre Vilardaga, Christopher S. Walker, Ji Ming Wang, Donald T. Ward, Hans-Jürgen Wester, Gary B. Willars, Tom Lloyd Williams, Trent M. Woodruff, Chengcan Yao, Richard D. Ye

**Affiliations:** 1 School of Life Sciences, University of Nottingham Medical School, Nottingham, NG7 2UH, UK,; 2 Monash Institute of Pharmaceutical Sciences and Department of Pharmacology, Monash University, Parkville, Victoria 3052, Australia,; 3 Clinical Pharmacology Unit, University of Cambridge, Cambridge, CB2 0QQ, UK,; 4 School of Physiology, Pharmacology and Neuroscience, University of Bristol, Bristol, BS8 1TD, UK,; 5 School of Engineering, Arts, Science and Technology, University of Suffolk, Ipswich, IP4 1QJ, UK,; 6 Neuroscience Division, Medical Education Institute, Ninewells Hospital and Medical School, University of Dundee, Dundee, DD1 9SY, UK,; 7 Medway School of Pharmacy, The Universities of Greenwich and Kent at Medway, Anson Building, Central Avenue, Chatham Maritime, Chatham, Kent, ME4 4TB, UK,; 8 Centre for Discovery Brain Sciences, University of Edinburgh, Edinburgh, EH8 9XD, UK,; 9 University of Milan, Milan, Italy,; 10 Florida International University, Miami, USA,; 11 Emory University, Atlanta, USA,; 12 King Saud University, Riyadh, Kingdom of Saudi Arabia,; 13 Karolinska University Hospital, Stockholm, Sweden,; 14 University of Birmingham, Birmingham, UK,; 15 Florey Institute of Neuroscience and Mental Health, Melbourne, Australia,; 16 University of Toronto, Toronto, Canada,; 17 Leipzig University, Leipzig, Germany,; 18 Technical University of Munich, Freising, Germany,; 19 Cedars-Sinai Medical Center, Los Angeles, USA,; 20 University of Basel, Basel, Switzerland,; 21 The Francis Crick Institute, London, UK,; 22 Sanford Burnham Prebys Medical Discovery Institute, La Jolla, USA,; 23 University of Grenoble Alpes, Grenoble, France,; 24 French Institute of Health and Medical Research (INSERM), Toulouse, France,; 25 University of Copenhagen, Copenhagen, Denmark,; 26 University College London, London, UK,; 27 University of Padova, Padova, Italy,; 28 University of Córdoba, Córdoba, Spain,; 29 National Institute of Health, Bethesda, USA,; 30 Durham University, Durham, UK,; 31 Harvard University, Boston, USA,; 32 University of Milan Bicocca, Vedano al Lambro, Italy,; 33 University of California San Diego, La Jolla, USA,; 34 University of Bari, Bari, Italy,; 35 University of California Irvine, Irvine, USA,; 36 University of Montréal, Montréal, Canada,; 37 King’s College London, London, UK,; 38 French Institute of Health and Medical Research (INSERM), Paris, France,; 39 University of Gothenburg, Gothenburg, Sweden,; 40 Keele University, Keele, UK,; 41 University of Pennsylvania, Pennsylvania, USA,; 42 Temple University, Philadelphia, USA,; 43 University of Sherbrooke, Sherbrooke, Canada,; 44 University of Iowa, Iowa City, USA,; 45 Labcorp Drug Development, Somerset, USA,; 46 Saint Petersburg State University, Saint-Petersburg, Russia,; 47 University of Otago, Dunedin, New Zealand,; 48 MG Consulting Co, Basel, Switzerland,; 49 University of Wisconsin, Madison, USA,; 50 French National Centre for Scientific Research, Montpellier, France,; 51 Karolinska Institute, Stockholm, Sweden,; 52 Amsterdam University, Amsterdam, The Netherlands,; 53 University of Liège, Liège, Belgium,; 54 Medical University of Graz, Graz, Austria,; 55 San Diego State University, San Diego, USA,; 56 University of Calgary, Calgary, Canada,; 57 Ehime University, Ehime, Japan,; 58 University of Melbourne, Melbourne, Australia,; 59 Semmelweis University, Budapest, Hungary,; 60 Leiden University, Leiden, The Netherlands,; 61 Vanderbilt University, Nashville, USA,; 62 Lerner Research Institute, Cleveland, USA,; 63 Novartis, Basel, Switzerland,; 64 Cleveland Clinic Lerner Research Institute, Cleveland, USA,; 65 Strathclyde University, Glasgow, UK,; 66 Rakuno Gakuen University, Ebetsu, Japan,; 67 University of Hamburg, Hamburg, Germany,; 68 University of Helsinki, Helsinki, Finland,; 69 Uppsala University, Uppsala, Sweden,; 70 University of Queensland, Brisbane, Australia,; 71 University of Queensland, Queensland, Australia,; 72 Boston University, Boston, USA,; 73 University of Rouen, Rouen, France,; 74 Friedrich Schiller University Jena, Jena, Germany,; 75 University of Queensland, Queensland, Australia,; 76 University of Cambridge, Cambridge, UK,; 77 French National Centre for Scientific Research (CNRS), Valbonne, France,; 78 Johannes Gutenberg University Mainz, Mainz, Germany,; 79 Mayo Foundation for Medical Education and Research, Scottsdale, USA,; 80 University of Bonn, Bonn, Germany,; 81 Edge Hill University, Bethesda, USA,; 82 Max Planck Institute for Heart and Lung Research, Bad Nauheim, Germany,; 83 Free University of Brussels, Brussels, Belgium,; 84 University of Aberdeen, Aberdeen, UK,; 85 University of Montpellier, Montpellier, France,; 86 University of New Mexico, Albuquerque, USA,; 87 Montana State University, Bozeman, USA,; 88 University of Ferrara, Ferrara, Italy,; 89 Queen Mary University of London, London, UK,; 90 Deakin University, Waurn Ponds, Australia,; 91 Kumamoto University, Kumamoto, Japan,; 92 Monash University, Melbourne, Australia,; 93 Monash University, Melbourne, Australia,; 94 Kosan Biosciences Inc., San Francisco, USA,; 95 Florida Atlantic University, Jupiter, USA,; 96 University of Brescia, Brescia, Italy,; 97 Cleveland Clinic, Cleveland, USA,; 98 Maastricht University, Maastricht, The Netherlands,; 99 Monash University, Melbourne, Australia,; 100 University of Pittsburgh, Pittsburgh, USA,; 101 University of Auckland, Auckland, New Zealand,; 102 National Institute of Health, Frederick, USA,; 103 University of Manchester, Manchester, UK,; 104 Technical University of Munich, Munich, Germany,; 105 University of Leicester, Leicester, UK,; 106 University of Edinburgh, Edinburgh, UK,; 107 The Chinese University of Hong Kong, Shenzhen, China

## Abstract

The Concise Guide to PHARMACOLOGY 2023/24 is the sixth in this series of biennial publications. The Concise Guide provides concise overviews, mostly in tabular format, of the key properties of approximately 1800 drug targets, and about 6000 interactions with about 3900 ligands. There is an emphasis on selective pharmacology (where available), plus links to the open access knowledgebase source of drug targets and their ligands (https://www.guidetopharmacology.org), which provides more detailed views of target and ligand properties. Although the Concise Guide constitutes almost 500 pages, the material presented is substantially reduced compared to information and links presented on the website. It provides a permanent, citable, point‐in‐time record that will survive database updates. The full contents of this section can be found at http://onlinelibrary.wiley.com/doi/bph.16177. G protein‐coupled receptors are one of the six major pharmacological targets into which the Guide is divided, with the others being: ion channels, nuclear hormone receptors, catalytic receptors, enzymes and transporters. These are presented with nomenclature guidance and summary information on the best available pharmacological tools, alongside key references and suggestions for further reading. The landscape format of the Concise Guide is designed to facilitate comparison of related targets from material contemporary to mid‐2023, and supersedes data presented in the 2021/22, 2019/20, 2017/18, 2015/16 and 2013/14 Concise Guides and previous Guides to Receptors and Channels. It is produced in close conjunction with the Nomenclature and Standards Committee of the International Union of Basic and Clinical Pharmacology (NC‐IUPHAR), therefore, providing official IUPHAR classification and nomenclature for human drug targets, where appropriate.


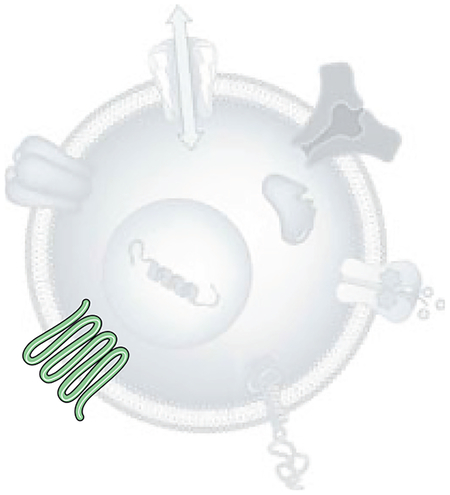


**Overview:** G protein-coupled receptors (GPCRs) are the largest class of membrane proteins in the human genome. The term “7TM receptor” is commonly used interchangeably with “GPCR”, although there are some receptors with seven transmembrane domains that do not signal through G proteins. GPCRs share a common architecture, each consisting of a single polypeptide with an extracellular N-terminus, an intracellular C-terminus and seven hydrophobic transmembrane domains (TM1-TM7) linked by three extracellular loops (ECL1-ECL3) and three intracellular loops (ICL1-ICL3). About 800 GPCRs have been identified in man, of which about half have sensory functions, mediating olfaction (~400), taste (33), light perception (10) and pheromone signalling (5) [1771]. The remaining ~350 non-sensory GPCRs mediate signalling by ligands that range in size from small molecules to peptides to large proteins; they are the targets for the majority of drugs in clinical usage [1966, 2141], although only a minority of these receptors are exploited therapeutically. The first classification scheme to be proposed for GPCRs [1329] divided them, on the basic of sequence homology, into six classes. These classes and their prototype members were as follows: **Class A** (rhodopsin-like), **Class B** (secretin receptor family), **Class C** (metabotropic glutamate), **Class D** (fungal mating pheromone receptors), **Class E** (cyclic AMP receptors) and **Class F** (frizzled/smoothened). Of these, classes D and E are not found in vertebrates. An alternative classification scheme “GRAFS” [2294] divides vertebrate GPCRs into five classes, overlapping with the A-F nomenclature, *viz*:

**Glutamate family** (class C), which includes metabotropic glutamate receptors, a calcium-sensing receptor and GABA_B_ receptors, as well as three taste type 1 receptors and a family of pheromone receptors (V2 receptors) that are abundant in rodents but absent in man [1771].

**Rhodopsin family** (class A), which includes receptors for a wide variety of small molecules, neurotransmitters, peptides and hormones, together with olfactory receptors, visual pigments, taste type 2 receptors and five pheromone receptors (V1 receptors).

Adhesion family GPCRs are phylogenetically related to class B receptors, from which they differ by possessing large extracellular N-termini that are autoproteolytically cleaved from their 7TM domains at a conserved “GPCR proteolysis site” (GPS) which lies within a much larger (~320 residue) “GPCR autoproteolysis-inducing” (GAIN) domain, an evolutionary ancient mofif also found in polycystic kidney disease 1 (PKD1)-like proteins, which has been suggested to be both required and sufficient for autoproteolysis [2088].

Frizzled family consists of 10 Frizzled proteins (FZD_1–10_) and Smoothened (SMO). The FZDs are activated by secreted lipoglycoproteins of the WNT family, whereas SMO is indirectly activated by the Hedgehog (HH) family of proteins acting on the transmembrane protein Patched (PTCH).

**Secretin family**, encoded by 15 genes in humans. The ligands for receptors in this family are polypeptide hormones of 27–141 amino acid residues; nine of the mammalian receptors respond to ligands that are structurally related to one another (glucagon, glucagon-like peptides (GLP-1, GLP-2), glucose-dependent insulinotropic polypeptide (GIP), secretin, vasoactive intestinal peptide (VIP), pituitary adenylate cyclase-activating polypeptide (PACAP) and growth-hormone-releasing hormone (GHRH)) [949].

**Table:** GPCR families

Family	Class A	Class B (Secretin)	Class C (Glutamate)	Adhesion	Frizzled
Receptors with known ligands	197	15	12	0	11
Orphans	87 (54)^a^	-	8 (1)^a^	26 (6)^a^	0
Sensory (olfaction)	390^b^^,^^c^	-	-	-	-
Sensory (vision)	10^d^ opsins	-	-	-	-
Sensory (taste)	30^c^ taste 2	-	3^c^ taste 1	-	-
Sensory (pheromone)	5^c^ vomeronasal 1	-	-	-	-
Total	719	15	22	33	11

a Numbers in brackets refer to orphan receptors for which an endogenous ligand has been proposed in at least one publication, see [532];

b [1950];

c [1771];

d [2556].

## Pseudogenes

A number of pseudogenes have been identified in the human genome, which, in some cases, have a shared ancestry with functional G protein-coupled receptors in other species, including rats and mice.

A curated list includes:

*ADGRE4P*, *GNRHR2*, *GPR79*, *HTR5BP*, *NPY6R*, *TAAR3P*, *TAAR4P*, *TAAR7P*, *TAS2R12P*, *TAS2R15P*, *TAS2R18P*, *TAS2R2P*, *TAS2R62P*, *TAS2R63P*, *TAS2R64P*, *TAS2R67P*, *TAS2R68P*, *TAS2R6P*. A more detailed listing containg further information can be viewed here.

## Odorant receptors

Odorant receptors are G protein-coupled receptors responsible for the detection of generally volatile compounds associated with olfaction. These are not currently included as they are not yet associated with extensive pharmacological data but are curated in the following databases: The gene list of olfactory receptors at HGNC, and curated by HORDE and ORDB.

## Orphan and other 7TM receptors

G protein-coupled receptors → Orphan and other 7TM receptors

**Overview**: This set contains ‘orphan’ G protein coupled receptors where the endogenous ligand(s) is not known.

### Class A Orphans

G protein-coupled receptors → Orphan and other 7TM receptors → Class A Orphans

**Overview**: Table 11 lists a number of putative GPCRs identified by **NC-IUPHAR** [712], for which preliminary evidence for an endogenous ligand has been published, or for which there exists a potential link to a disease, or disorder. These GPCRs have recently been reviewed in detail [532]. The GPCRs in Table 11 are all Class A, rhodopsin-like GPCRs. Class A orphan GPCRs not listed in Table 11 are putative GPCRs with as-yet unidentified endogenous ligands.

In addition the orphan receptors *GPR18*, *GPR55* and *GPR119* which are reported to respond to endogenous agents analogous to the endogenous cannabinoid ligands have been grouped together (GPR18, GPR55 and GPR119).

**Table:** 

Nomenclature	*GPR3*	*GPR4*
HGNC, UniProt	*GPR3*, P46089	*GPR4*, P46093
Endogenous ligands	–	Protons
Agonists	diphenyleneiodonium chloride [2880]	–
Comments	Sphingosine 1-phosphate was reported to be an endogenous agonist [2629], but this finding was not replicated in subsequent studies [2885]. Reported to activate adenylyl cyclase constitutively through G_s_ [638]. Gene disruption results in premature ovarian ageing [1449], reduced β-amyloid deposition [2561] and hypersensitivity to thermal pain [2229] in mice. First small molecule inverse agonist [1161] and agonists identified [2880].	An initial report suggesting activation by lysophosphatidylcholine and sphingosylphosphorylcholine [2955] has been retracted [1908]. GPR4, GPR65, GPR68 and GPR132 are now thought to function as proton-sensing receptors detecting acidic pH [532, 2344]. Gene disruption is associated with increased perinatal mortality and impaired vascular proliferation [2869]. Negative allosteric modulators of GPR4 have been reported [2587].

**Table:** 

Nomenclature	*GPR6*	*GPR12*	*GPR15*
HGNC, UniProt	*GPR6*, P46095	*GPR12*, P47775	*GPR15*, P49685
Comments	An initial report that sphingosine 1-phosphate (S1P) was a high-affinity ligand (EC_50_ value of 39nM) [1097, 2629] was not repeated in arrestin-based assays [2436, 2885]. Reported to activate adenylyl cyclase constitutively through G_s_ and to be located intracellularly [1971]. GPR6-deficient mice showed reduced striatal cyclic AMP production in vitro and selected alterations in instrumental conditioning in vivo. [1557].	Reports that sphingosine 1-phosphate is a ligand of GPR12 [1096, 2629] have not been replicated in arrestin-based assays [2436, 2885]. Gene disruption results in dyslipidemia and obesity [206].	Reported to act as a co-receptor for HIV [633]. In an infection-induced colitis model, Gpr15 knockout mice were more prone to tissue damage and inflammatory cytokine expression [1276].

**Table:** 

Nomenclature	*GPR17*	*GPR19*	*GPR20*	*GPR21*
HGNC, UniProt	*GPR17* *,* Q13304	*GPR19* *,* Q15760	*GPR20*, Q99678	*GPR21*, Q99679
Endogenous agonists	UDP-glucose [177, 461], LTC_4_ [461], UDP-galactose [177, 461], UDP [177, 461], LTD_4_ [461]	–	–	–
Agonists	–	adropin (ENHO, Q6UWT2) [2138]	–	–
Comments	Reported to be a dual leukotriene and UDP receptor [461]. Another group instead proposed that GPR17 functions as a negative regulator of the CysLT_1_ receptor response to leukotriene D_4_ (LTD_4_). For further discussion, see [532]. Reported to antagonize CysLT_1_ receptor signalling in vivo and in vitro [1612]. See reviews [110] and [532].	–	Reported to inhibit adenylyl cyclase constitutively through G_i/o_ [955]. GPR20 deficient mice exhibit hyperactivity characterised by increased total distance travelled in an open field test [274].	Gpr21 knockout mice were resistant to diet-induced obesity, exhibiting an increase in glucose tolerance and insulin sensitivity, as well as a modest lean phenotype [1961].

**Table:** 

Nomenclature	*GPR22*	*GPR25*	*GPR26*	*GPR27*	*GPR31*	*GPR32*	*GPR33*
HGNC, UniProt	*GPR22*, Q99680	*GPR25*, O00155	*GPR26*, Q8NDV2	*GPR27* *,* Q9NS67	*GPR31*, O00270	*GPR32*, O75388	*GPR33*, Q49SQ1
Potency order of endogenous ligands	–	–	–	–	–	resolvin D1 > LXA_4_	–
Endogenous agonists	–	–	–	–	12S-HETE [901] – Mouse	resolvin D1 [1359], LXA_4_ [1359]	–
Labelled ligands	–	–	–	–	–	[^3^H]resolvin D1 (Agonist) [1359]	–
Comments	Gene disruption results in increased severity of functional decompensation following aortic banding [13]. Identified as a susceptibility locus for osteoarthritis [668, 1253, 2648].	–	Has been reported to activate adenylyl cyclase constitutively through G_s_ [1186]. Gpr26 knockout mice show increased levels of anxiety and depression-like behaviours [2927].	Knockdown of Gpr27 reduces endogenous mouse insulin promotor activity and glucose-stimulated insulin secretion [1365].	See [532] for discussion of pairing.	Resolvin D1 has been demonstrated to activate GPR32 in two publications [427, 1359]. The pairing was not replicated in a recent study based on arrestin recruitment [2436]. GPR32 is a pseudogene in mice and rats. See reviews [110] and [532].	GPR33 is a pseudogene in most individuals, containing a premature stop codon within the coding sequence of the second intracellular loop [2204].

**Table:** 

Nomenclature	*GPR34*	*GPR35*
HGNC, UniProt	*GPR34*, Q9UPC5	*GPR35*, Q9HC97
Endogenous agonists	lysophosphatidylserine [1297, 2483]	2-oleoyl-LPA [1941], kynurenic acid [2436, 2725]
Comments	Lysophosphatidylserine has been reported to be a ligand of GPR34 in several publications, but the pairing was not replicated in a recent study based on arrestin recruitment [2436]. Fails to respond to a variety of lipid-derived agents [2885]. Gene disruption results in an enhanced immune response [1512]. Characterization of agonists at this receptor is discussed in [1102] and [532].	Several studies have shown that kynurenic acid is an agonist of GPR35 but it remains controversial whether the proposed endogenous ligand reaches sufficient tissue concentrations to activate the receptor [1366]. 2-oleoyl-LPA has also been proposed as an endogenous ligand [1941] but these results were not replicated in an arrestin assay [2436]. The phosphodiesterase inhibitor zaprinast [2550] has become widely used as a surrogate agonist to investigate GPR35 pharmacology and signalling [2550]. GPR35 is also activated by the pharmaceutical adjunct pamoic acid [2943]. See reviews [532] and [590].

**Table:** 

Nomenclature	*GPR37*	*GPR37L1*	*GPR39*
HGNC, UniProt	*GPR37*, O15354	*GPR37L1*, O60883	*GPR39*,O43194
Endogenous agonists	–	–	Zn^2+^ [1043]
Agonists	neuropeptide head activator [2170]	–	–
Comments	Reported to associate and regulate the dopamine transporter [1649] and to be a substrate for parkin [1647]. Gene disruption results in altered striatal signalling [1648]. The peptides prosaptide and prosaposin are proposed as endogenous ligands for GPR37 and GPR37L1 [1715].	The peptides prosaptide and prosaposin are proposed as endogenous ligands for GPR37 and GPR37L1 [1715].	Zn^2+^ has been reported to be a potent and efficacious agonist of human, mouse and rat GPR39 [2877]. Obestatin (GHRL, Q9UBU3), a fragment from the ghrelin precursor, was reported initially as an endogenous ligand, but subsequent studies failed to reproduce these findings. GPR39 has been reported to be down-regulated in adipose tissue in obesity-related diabetes [360]. Gene disruption results in obesity and altered adipocyte metabolism [2034]. Reviewed in [532].

**Table:** 

Nomenclature	*GPR45*	*GPR50*	*GPR52*	*GPR61*	*GPR62*	*GPR63*
HGNC, UniProt	*GPR45*, Q9Y5Y3	*GPR50*, Q13585	*GPR52*,Q9Y2T5	*GPR61*, Q9BZJ8	*GPR62*,Q9BZJ7	*GPR63*,Q9BZJ6
Comments	–	GPR50 is structurally related to MT_1_ and MT_2_ melatonin receptors, with which it heterodimerises constitutively and specifically [1490]. Gpr50 knockout mice display abnormal thermoregulation and are much more likely than wildtype mice to enter fasting-induced torpor [153].	First small molecule agonist reported [2343].	GPR61 deficient mice exhibit obesity associated with hyperphagia [1852]. Although no endogenous ligands have been identified, 5-(nonyloxy)tryptamine has been reported to be a low affinity inverse agonist [2529].	–	Sphingosine 1-phosphate and dioleoylphosphatidic acid have been reported to be low affinity agonists for GPR63 [1894] but this finding was not replicated in an arrestin-based assay [2885].

**Table:** 

Nomenclature	*GPR65*	*GPR68*	*GPR75*	*GPR78*	*GPR79*
HGNC, UniProt	*GPR65*, Q8IYL9	*GPR68*, Q15743	*GPR75*, O95800	*GPR78*, Q96P69	*GPR79*, –
Endogenous ligands	Protons	Protons	–	–	–
Allosteric modulators (Positive)	–	ogerin (pK_B_ 5) [1078], lorazepam (lorazepam characterised as a non-selective GPR68 positive allosteric modulator for the agonist proton in cAMP production) [1078]	–	–	–
Comments	GPR4, GPR65, GPR68 and GPR132 are now thought to function as proton-sensing receptors detecting acidic pH [532, 2344]. Reported to activate adenylyl cyclase; gene disruption leads to reduced eosinophilia in models of allergic airway disease [1345].	GPR68 was previously identified as a receptor for sphingosylphosphorylcholine (SPC) [2844], but the original publication has been retracted [1]. GPR4, GPR65, GPR68 and GPR132 are now thought to function as proton-sensing receptors detecting acidic pH [532, 2344]. A family of 3,5-disubstituted isoxazoles were identified as agonists of GPR68 [2231].	CCL5 (*CCL5*, P13501) was reported to be an agonist of GPR75 [1098], but the pairing could not be repeated in an arrestin assay [2436].	GPR78 has been reported to be constitutively active, coupled to elevated cAMP production [1186].	–

**Table:** 

Nomenclature	*GPR82*	*GPR83*	*GPR84*	*GPR85*	*GPR87*
HGNC, UniProt	*GPR82*, Q96P67	*GPR83*, Q9NYM4	*GPR84*, Q9NQS5	*GPR85*, P60893	*GPR87*, Q9BY21
Endogenous agonists	–	–	–	–	LPA [1832, 2510]
Agonists	–	PEN {Mouse} [838] – Mouse, Zn^2+^ [1823] – Mouse	decanoic acid [2436, 2727], undecanoic acid [2727], lauric acid [2727], 6-nonylpyridine-2,4-diol (orthosteric) [1655], DL-175 (orthosteric) [1655], Embelin (orthosteric) [1655], PSB-16434 (orthosteric) [1655], ZQ-16 (orthosteric) [1655]	–	–
Allosteric modulators	–	–	DIM (Agonist) [1655]	–	–
Comments	Mice with Gpr82 knockout have a lower body weight and body fat content associated with reduced food intake, decreased serum triglyceride levels, as well as higher insulin sensitivity and glucose tolerance [652].	One isoform has been implicated in the induction of CD4(+) CD25(+) regulatory T cells (Tregs) during inflammatory immune responses [939]. The extracellular N-terminal domain is reported as an intramolecular inverse agonist [1824].	Medium chain free fatty acids with carbon chain lengths of 9–14 activate GPR84 [2498, 2727]. A surrogate ligand for GPR84, 6-n-octylaminouracil has also been proposed [2498]. See review [532] for discussion of classification. Mutational analysis and molecular modelling of GPR84 has been reported [1897].	Proposed to regulate hippocampal neurogenesis in the adult, as well as neurogenesis-dependent learning and memory [406].	–

**Table:** 

Nomenclature	*GPR88*	*GPR101*	*GPR132*	*GPR135*	*GPR139*	*GPR141*	*GPR142*
HGNC, UniProt	*GPR88*, Q9GZN0	*GPR101*, Q96P66	*GPR132*, Q9UNW8	*GPR135*, Q8IZ08	*GPR139*, Q6DWJ6	*GPR141*, Q7Z602	*GPR142*, Q7Z601
Endogenous ligands	–	–	Protons	–	–	–	–
Comments	Gene disruption results in altered striatal signalling [1560]. Small molecule agonists have been reported [198].	Mutations in GPR101 have been linked to gigantism and acromegaly [2613].	GPR4, GPR65, GPR68 and GPR132 are now thought to function as protonsensing receptors detecting acidic pH [532, 2344]. Reported to respond to lysophosphatidylcholine [1197], but later retracted [2800].	–	Peptide agonists have been reported [1115].	–	Small molecule agonists have been reported [2588, 2904].

**Table:** 

Nomenclature	*GPR146*	*GPR148*	*GPR149*	*GPR150*	*GPR151*	*GPR152*	*GPR153*
HGNC, UniProt	*GPR146*, Q96CH1	*GPR148*,Q8TDV2	*GPR149*, Q86SP6	*GPR150*, Q8NGU9	*GPR151*,Q8TDV0	*GPR152* *,* Q8TDT2	*GPR153*, Q6NV75
Comments	Yosten et al. demonstrated inhibition of proinsulin C-peptide (INS, P01308)-induced stimulation of cFos expression folllowing knockdown of GPR146 in KATO III cells, suggesting proinsulin C-peptide as an endogenous ligand of the receptor [2900]. Reviewed iin [1531].	–	Gpr149 knockout mice displayed increased fertility and enhanced ovulation, with increased levels of FSH receptor and cyclin D2 mRNA levels [635].	–	GPR151 responded to galanin with an EC_50_ value of 2 μM, suggesting that the endogenous ligand shares structural features with galanin (GAL, P22466) [1095].	–	–

**Table:** 

Nomenclature	*GPR160*	*GPR161*	*GPR162*	*GPR171*	*GPR173*	*GPR174*
HGNC, UniProt	*GPR160*, Q9UJ42	*GPR161*, Q8N6U8	*GPR162*, Q16538	*GPR171*, O14626	*GPR173*, Q9NS66	*GPR174*, Q9BXC1
Endogenous agonists	–	–	–	–	–	lysophosphatidylserine [1108]
Comments	–	A C-terminal truncation (deletion) mutation in Gpr161 causes congenital cataracts and neural tube defects in the vacuolated lens (vl) mouse mutant [1677]. The mutated receptor is associated with cataract, spina bifida and white belly spot phenotypes in mice [1340]. Gene disruption is associated with a failure of asymmetric embryonic development in zebrafish [1485].	–	GPR171 has been shown to be activated by the endogenous peptide BigLEN {Mouse}. This receptor-peptide interaction is believed to be involved in regulating feeding and metabolism responses [837].	–	See [1102] which discusses characterization of agonists at this receptor.

**Table:** 

Nomenclature	*GPR176*	*GPR182*	*GPR183*
HGNC, UniProt	*GPR176*, Q14439	*GPR182*, O15218	*GPR183*, P32249
Endogenous agonists	–	–	7α,25-dihydroxycholesterol [935, 1545], 7α,27-dihydroxycholesterol [1545], 7β, 25-dihydroxycholesterol [1545], 7β, 27-dihydroxycholesterol [1545]
Comments	–	Rat GPR182 was first proposed as the adrenomedullin receptor [1215]. However, it was later reported that rat and human GPR182 did not respond to adrenomedullin [1249] and GPR182 is not currently considered to be a genuine adrenomedullin receptor [971].	Two independent publications have shown that 7α,25-dihydroxycholesterol is an agonist of GPR183 and have demonstrated by mass spectrometry that this oxysterol is present endogenously in tissues [935, 1545]. Gpr183-deficient mice show a reduction in the early antibody response to a T-dependent antigen. GPR183-deficient B cells fail to migrate to the outer follicle and instead stay in the follicle centre [1240, 2021].

**Table:** 

Nomenclature	*LGR4*	*LGR5*
HGNC, UniProt	*LGR4*, Q9BXB1	*LGR5*, O75473
Endogenous agonists	R-spondin-2 (RSPO2,Q6UXX9) [349], R-spondin-1 (RSPO1, Q2MKA7) [349], R-spondin-3 (RSPO3, Q9BXY4) [349], R-spondin-4 (RSPO4, Q2I0M5) [349]	R-spondin-2 (RSPO2, Q6UXX9) [349], R-spondin-1 (RSPO1, Q2MKA7) [349], R-spondin-3 (RSPO3, Q9BXY4) [349], R-spondin-4 (RSPO4, Q2I0M5) [349]
Comments	LGR4 does not couple to heterotrimeric G proteins or recruit arrestins when stimulated by the R-spondins, indicating a unique mechanism of action. R-spondins bind to LGR4, which specifically associates with Frizzled and LDL receptor-related proteins (LRPs) that are activated by the extracellular Wnt molecules and then trigger canonical Wnt signalling to increase gene expression [349, 546, 2225]. Gene disruption leads to multiple developmental disorders [1172, 1580, 2432, 2763].	The four R-spondins can bind to LGR4, LGR5, and LGR6, which specifically associate with Frizzled and LDL receptor-related proteins (LRPs), proteins that are activated by extracellular Wnt molecules and which then trigger canonical Wnt signalling to increase gene expression [349, 546].

**Table:** 

Nomenclature	*LGR6*	*MAS1*	*MAS1L*	*MRGPRD*	*MRGPRE*	*MRGPRF*
HGNC, UniProt	*LGR6*,Q9HBX8	*MAS1*, P04201	*MAS1L*,P35410	*MRGPRD*, Q8TDS7	*MRGPRE*, Q86SM8	*MRGPRF**,* Q96AM1
Endogenous agonists	R-spondin-1 (RSPO1, Q2M-KA7) [349, 546], R-spondin-2 (RSPO2, Q6UXX9) [349, 546], R-spondin-3 (RSPO3, Q9BXY4) [349, 546], R-spondin-4 (RSPO4, Q2I0M5) [349, 546]	–	–	β-alanine [2373, 2436]	–	–
Agonists	–	angiotensin-(1-7) (AGT, P01019) [825] – Mouse	–	–	–	–
Comments	–	–	–	An endogenous peptide with a high degree of sequence similarity to angiotensin-(1-7) (AGT, P01019), alamandine (AGT), was shown to promote NO release in MRGPRD-transfected cells. The binding of alamandine to MRGPRD to was shown to be blocked by D-Pro^7^angiotensin-(1–7), β-alanine and PD123319 [1420]. Genetic ablation of MRGPRD+ neurons of adult mice decreased behavioural sensitivity to mechanical stimuli but not to thermal stimuli [368]. See reviews [532] and [2430].	See reviews [532] and [2430].	MRGPRF has been reported to respond to stimulation by angiotensin metabolites [791]. See reviews [532] and [2430].

**Table:** 

Nomenclature	*MRGPRG*	*MRGPRX1*	*MRGPRX2*	*MRGPRX3*	*MRGPRX4*	*P2RY8*	*P2RY10*
HGNC, UniProt	*MRGPRG*, Q86SM5	*MRGPRX1*, Q96LB2	*MRGPRX2*, Q96LB1	*MRGPRX3*, Q96LB0	*MRGPRX4*, Q96LA9	*P2RY8*, Q86VZ1	*P2RY10*, O00398
Endogenous agonists	–	bovine adrenal medulla peptide 8-22 (*PENK*, P01210) [400, 1472, 2436]	PAMP-20 (*ADM*, P35318) [1207]	–	–	–	sphingosine 1-phosphate[1832], LPA [1832]
Agonists	–	–	cortistatin-14 {Mouse, Rat} [1207, 1413, 2193, 2436]	–	–	–	–
Selective agonists	–	–	PAMP-12 (human) [1207]	–	–	–	–
Comments	See reviews [532] and [2430].	Reported to mediate the sensation of itch [1550, 2384]. Reports that bovine adrenal medulla peptide 8-22 was the most potent of a series of proenkephalin A-derived peptides as an agonist of MRGPRX1 in assays of calcium mobilisation and radioligand binding [1472] were replicated in an independent study using an arrestin recruitment assay [2436]. See reviews [532] and [2430].	A diverse range of substances has been reported to be agonists of MRGPRX2, with cortistatin 14 the highest potency agonist in assays of calcium mobilisation [2193], also confirmed in an independent study using an arrestin recruitment assay [2436]. See reviews [532] and [2430].	–	See reviews [532] and [2430].	–	–

**Table:** 

Nomenclature	*TAAR2*	*TAAR3*	*TAAR4P*	*TAAR5*	*TAAR6*	*TAAR8*	*TAAR9*
HGNC, UniProt	*TAAR2*, Q9P1P5	*TAAR3P*, Q9P1P4	*TAAR4P*, –	*TAAR5*, O14804	*TAAR6*, Q96RI8	*TAAR8*, Q969N4	*TAAR9*, Q96RI9
Potency order of endogenous ligands	β-phenylethylamine > tryptamine [244]	–	–	–	–	–	–
Comments	Probable pseudogene in 10–15% of Asians due to a polymorphism (rs8192646) producing a premature stop codon at amino acid 168 [532].	TAAR3 is thought to be a pseudogene in man though functional in rodents [532].	Pseudogene in man but functional in rodents [532].	Trimethylamine is reported as an agonist [2711] and 3-iodothyronamine an inverse agonist [587].	–	–	TAAR9 appears to be functional in most individuals but has a polymorphic premature stop codon at amino acid 61 (rs2842899) with an allele frequency of 10–30% in different populations [2668].

Further reading on Class A OrphansAkbariP
 (2021) Sequencing of 640,000 exomes identifies *GPR75* variants associated with protection from obesity. Science
373:10.1126/science.abf8683PMC1027539634210852McNeilBD
 (2015) Identification of a mast-cell-specific receptor crucial for pseudo-allergic drug reactions. Nature
519: 237–4125517090
10.1038/nature14022PMC4359082WirthgenE
 (2017) Kynurenic Acid: The Janus-Faced Role of an Immunomodulatory Tryptophan Metabolite and Its Link to Pathological Conditions. Front Immunol
8: 195729379504
10.3389/fimmu.2017.01957PMC5770815

### Class C Orphans

G protein-coupled receptors → Orphan and other 7TM receptors → Class C Orphans

**Overview**: This set contains class C’orphan’ G protein coupled receptors where the endogenous ligand(s) is not known.

**Table:** 

Nomenclature	*GPR156*	*GPR158*	*GPR179*	*GPRC5A*	*GPRC5B*	*GPRC5C*	*GPRC5D*	*GPRC6* receptor
HGNC, UniProt	*GPR156*, Q8NFN8	*GPR158*, Q5T848	*GPR179*, Q6PRD1	*GPRC5A*, Q8NFJ5	*GPRC5B*, Q9NZH0	*GPRC5C*, Q9NQ84	*GPRC5D*, Q9NZD1	*GPRC6A*, Q5T6X5
Comments	–	–	–	–	–	–	–	GPRC6 is a related G_q_-coupled receptor which responds to basic amino acids [2760].

Further reading on Class C OrphansHarpsøeK
 (2017) Structural insight to mutation effects uncover a common allosteric site in class C GPCRs. Bioinformatics
33: 1116–112028011766
10.1093/bioinformatics/btw784PMC5408886

### Opsin receptors

G protein-coupled receptors → Orphan and other 7TM receptors → Opsin receptors

**Table:** 

Nomenclature	*OPN3*	*OPN4*	*OPN5*
HGNC, UniProt	*OPN3*, Q9H1Y3	*OPN4*, Q9UHM6	*OPN5*, Q6U736
Comments	–	–	Evidence indicates that UV light triggers OPN5 to activate G_i_-mediated signalling in mammalian tissues [1327].

### Taste 1 receptors

G protein-coupled receptors → Orphan and other 7TM receptors → Taste 1 receptors

**Overview**: Whilst the taste of acid and salty foods appear to be sensed by regulation of ion channel activity, bitter, sweet and umami tastes are sensed by specialised GPCR. Two classes of taste GPCR have been identified, T1R and T2R, which are similar in sequence and structure to Class C and Class A GPCR, respectively. Activation of taste receptors appears to involve gustducin- (Gαt3) and Gα14-mediated signalling, although the precise mechanisms remain obscure. Gene disruption studies suggest the involvement of PLCβ2 [2938], TRPM5 [2938] and IP3 [1025] receptors in post-receptor signalling of taste receptors. Although predominantly associated with the oral cavity, taste receptors are also located elsewhere, including further down the gastrointestinal system, in the lungs and in the brain.

**Sweet/Umami**: T1R3 acts as an obligate partner in T1R1/T1R3 and T1R2/T1R3 heterodimers, which sense umami or sweet, respectively. T1R1/T1R3 heterodimers respond to L-glutamic acid and may be positively allosterically modulated by 5’-nucleoside monophosphates, such as 5’-GMP [1501]. T1R2/T1R3 heterodimers respond to sugars, such as sucrose, and artificial sweeteners, such as saccharin [1874].

**Table:** 

Nomenclature	*TAS1R1*	*TAS1R2*	*TAS1R3*
HGNC, UniProt	*TAS1R1*, Q7RTX1	*TAS1R2*, Q8TE23	*TAS1R3*, Q7RTX0

**Comments**: Positive allosteric modulators of T1R2/T1R3 have been reported [2848]. Such compounds enhance the sweet taste of sucrose mediated by these receptors, but are tasteless on their own.

Further reading on Taste 1 receptorsBehrensM
 (2020) Structure-Function Analyses of Human Bitter Taste Receptors-Where Do We Stand?
Molecules
25: 442332993119
10.3390/molecules25194423PMC7582848PalmerRK. (2019) A Pharmacological Perspective on the Study of Taste. Pharmacol Rev
71: 20–4830559245
10.1124/pr.118.015974

### Taste 2 receptors

G protein-coupled receptors → Orphan and other 7TM receptors → Taste 2 receptors

**Overview**: Taste 2 receptors or Bitter taste receptors (TAS2Rs) are G protein-coupled receptors expressed in oral sensory cells and a variety of non-gustatory tissues. The ~25 human TAS2Rs share low amino acid sequence identities with other GPCR families and are classified as broadly tuned “generalist” receptors with numerous, chemically diverse bitter agonists, as narrowly tuned “specialist” receptors with very few activators, as intermediately tuned receptors with an average number of agonists, or receptors specialized to interact with chemically defined activators [1717]. The number of functional bitter taste receptor genes varies among species and orthologues might not be functionally conserved. Due to their expression in various tissues, the signal transduction of TAS2Rs is complex. Some TAS2Rs interact with drugs such as analgesic, anti-inflammatory, and antibacterial compounds. The specialist database BitterDB contains additional information on bitter compounds and receptors [516].

**Table:** 

Nomenclature	*TAS2R1*	*TAS2R3*	*TAS2R4*	*TAS2R5*	*TAS2R7*	*TAS2R8*	*TAS2R9*
HGNC, UniProt	*TAS2R1*,Q9NYW7	*TAS2R3*, Q9NYW6	*TAS2R4*, Q9NYW5	*TAS2R5*, Q9NYW4	*TAS2R7*, Q9NYW3	*TAS2R8*, Q9NYW2	*TAS2R9*, Q9NYW1
Agonists	cohumulone [1110], L-Phe-Phe-Phe [2638], L-Trp-Trp-Trp [1323], dextromethorphan [1717]	chloroquine[1717]	L-Trp-Trp-Trp [1323], azithromycin [1141], stevioside [990], colchicine [1717]	epigallocatechin-3-gallate [2421], Procyanidin C2 [2420], 1,10-Phenanthroline [1717]	grandinin [2421], malvidin-3-glucoside [2420], cromoglicic acid [1717]	oleuropein [511], andrographolide [718], chloramphenicol [1717], parthenolide [1717]	ofloxacin [607], pirenzepine [607], procainamide [607]
Antagonists	–	–	abscisic acid (pIC_50_ 4.5) [2103]	–	–	S6821 (pIC_50_ 7.7) [718], S7958 (pIC_50_ 7.2) [718]	–
Comments	–	–	–	–	Aluminum sulfate and magnesium sulfate act as TAS2R7 agonists; EC_50_ values are 29 μM [2739] and 14900 μM [166] respectively.	–	–

**Table:** 

Nomenclature	*TAS2R10*	*TAS2R13*	*TAS2R14*	*TAS2R16*
HGNC, UniProt	*TAS2R10*,Q9NYW0	*TAS2R13*,Q9NYV9	*TAS2R14*,Q9NYV8	*TAS2R16*,Q9NYV7
Agonists	bergapten [1633], cucurbitacin B [243], strychnine [1717], denatonium [243], haloperidol [1717]	denatonium [1717], diphenidol [1717]	flufenamic acid [576], aristolochic acid [1917], lupulone [1110], nobiletin [164], luteolin [2201], santonin [1917], datiscetin [2201], parthenolide [1917], (−)-α-thujone [163], picrotoxinin [163], N-octanoyl-L-homoserine lactone [1142], phloretin [2201], resveratrol [2201], tributyrin [1489], eriodictyol chalcone [2201], (±)Equol [2202], silibinin [2201], (+/−)-Eriodictyol [2201], genistein [1917], homoeriodictyol [2201], coumestrol [2202], vanillin [1801], L-Trp-Trp-Trp [1323], quinine [1717]	4-Nitrophenyl-β-D-mannopyranoside [2567], Phenyl-β-D-glucopyranoside [313], salicin [313], beta-gentiobiose [2250], D-(−)-Amygdalin [313], sinigrin [1717]
Antagonists	–	–	–	probenecid (pIC_50_ 3.5) [869]

**Table:** 

Nomenclature	*TAS2R19*	*TAS2R20*	*TAS2R30*	*TAS2R31*	*TAS2R38*	*TAS2R39*
HGNC, UniProt	*TAS2R19*, P59542	*TAS2R20*, P59543	*TAS2R30*, P59541	*TAS2R31*, P59538	*TAS2R38*, P59533	*TAS2R39*, P59534
Agonists	–	ritanserin [1633], methoxsalen [1633], cromoglicic acid [1717], tobramycin [1141], vanillin [1801], diphenidol [1717]	denatonium [1717], absinthin [1717], amarogentin [2215]	aristolochic acid [1370], saccharin [1370], acesulfame [1370], famotidine [1717]	phenylthiocarbamide [312], propylthiouracil [312], goitrin [2808], methimazole [165], sinigrin [1717]	theaflavin-3’-O-gallate [2857], theaflavin [2857], luteolin [2201], epicatechin gallate [2857], naringenin [2201], scutellarein [2201], datiscetin [2201], phloretin [2201], genistein [2202], (±)-Equol [2202], epigallocatechin [1857], (−)-Epicatechin [1857], vanillin [1801], L-Trp-Trp-Trp [1323]
Antagonists	–	–	–	GIV3727 (pIC_50_ 5.5) [2401], sakuranetin (pIC_50_ 5.3) [710], cyclamate (pIC_50_ 1.8) [161]	probenecid (pIC_50_ 3.7) [869]	6-Methylflavone (pIC_50_ 4.7) [2200]
Comments	–	–	–	–	Of the two main variants of TAS2R38, only the taster-variant (TAS2R38-PAV) is exquisitely sensitive to the listed agonists as well as to structurally related bitter substances from cruciferous vegetables. The non-taster variant (TAS2R38-AVI) is non-functional [312, 1278].	–

**Table:** 

Nomenclature	*TAS2R40*	*TAS2R41*	*TAS2R42*	*TAS2R43*
HGNC, UniProt	*TAS2R40*,P59535	*TAS2R41*,P59536	*TAS2R42*,Q7RTR8	*TAS2R43*,P59537
Agonists	cohumulone [1110], dapsone (Threshold=30 μM) [1717], quinine [1717]	chloramphenicol [2560]	–	aristolochic acid [1370], lactucopicrin [1406], aloin [2090], Cyclolinopeptide 1-Mso,3-Met-CL6 [1407], bengalensol [1408], grosheimin [2215], amarogentin [1717], saccharin [1370], acesulfame [1370]
Antagonists	GIV3727 (pIC_50_ 5.2) [2401]	–	–	GIV3727 (pIC_50_ 4.9) [2401], 3-methylhexanal (pIC_50_ 4.1) [2479], citronellal (pIC_50_ 4.1) [2479], cyclamate (pIC_50_ 2.3) [161]

**Table:** 

Nomenclature	*TAS2R45*	*TAS2R46*	*TAS2R50*	*TAS2R60*
HGNC, UniProt	*TAS2R45*,P59539	*TAS2R46*,P59540	*TAS2R50*,P59544	*TAS2R60*,P59551
Agonists	–	lactucopicrin [1406], strychnine [288], grosheimin [2215], absinthin [288], bengalensol [1408], andrographolide [2215], amarogentin [2215], picrotoxinin [288], denatonium [1717], colchicine [1717], L-Trp-Trp-Trp [1323]	andrographolide [162], amarogentin [162]	–
Antagonists	–	3β-hydroxydihydrocostunolide (pIC_50_ 5.3) [289]	–	–

Further reading on Taste 2 receptorsPalmerRK. (2019) A Pharmacological Perspective on the Study of Taste. Pharmacol Rev
71: 20–4830559245
10.1124/pr.118.015974

### Other 7TM proteins

G protein-coupled receptors → Orphan and other 7TM receptors → Other 7TM proteins

**Overview**: These proteins are predicted to have 7TM domains, but functional studies have yet to confirm them as G protein-coupled receptors.

**Table:** 

Nomenclature	*GPR107*	*GPR137*	*TPRA1*	*GPR143*	*GPR157*
HGNC, UniProt	*GPR107*,Q5VW38	*GPR137*, Q96N19	*TPRA1*, Q86W33	*GPR143*, P51810	*GPR157*, Q5UAW9
Endogenous agonists	–	–	–	levodopa [1565]	–
Comments	GPR107 is a member of the LUSTR family of proteins found in both plants and animals, having similar topology to G protein-coupled receptors [632]	–	TPRA1 shows no homology to known G protein-coupled receptors.	Loss-of-function mutations underlie ocular albinism type 1 [143].	GPR157 has ambiguous sequence similarities to several different GPCR families (class A, class B and the slime mould cyclic AMP receptor). Because of its distant relationship to other GPCRs, it cannot be readily classified.

Further reading on Orphan and other 7TM receptorsDavenportAP
 (2013) International Union of Basic and Clinical Pharmacology. LXXXVIII. G protein-coupled receptor list: recommendations for new pairings with cognate ligands. Pharmacol Rev
65: 967–8623686350
10.1124/pr.112.007179PMC3698937GilissenJ
 (2016) Insight into SUCNR1 (GPR91) structure and function. Pharmacol Ther
159: 56–6526808164
10.1016/j.pharmthera.2016.01.008InselPA
 (2015) G Protein-Coupled Receptor (GPCR) Expression in Native Cells: “Novel” endoGPCRs as Physiologic Regulators and Therapeutic Targets. Mol Pharmacol
88: 181–725737495
10.1124/mol.115.098129PMC4468643KhanMZ
 (2017) Neuro-psychopharmacological perspective of Orphan receptors of Rhodopsin (class A) family of G protein-coupled receptors. Psychopharmacology (Berl.)
234: 1181–120728289782
10.1007/s00213-017-4586-9MackenzieAE
 (2017) The emerging pharmacology and function of GPR35 in the nervous system. Neuropharmacology
113: 661–67126232640
10.1016/j.neuropharm.2015.07.035NgoT
 (2016) Identifying ligands at orphan GPCRs: current status using structure-based approaches. Br J Pharmacol
173: 2934–5126837045
10.1111/bph.13452PMC5341249

## 5-Hydroxytryptamine receptors

G protein-coupled receptors → 5-Hydroxytryptamine receptors

**Overview**: 5-HT receptors (**nomenclature as agreed by the NC-IUPHAR Subcommittee on 5-HT receptors** [1063] **and subsequently revised** [954]) are, with the exception of the ionotropic 5-HT_3_ class, GPCRs where the endogenous agonist is 5-hydroxytryptamine. The diversity of metabotropic 5-HT receptors is increased by alternative splicing that produces isoforms of the 5-HT_2A_ (non-functional), 5-HT_2C_ (non-functional), 5-HT_4_, 5-HT_6_ (non-functional) and 5-HT_7_ receptors. Unique amongst the GPCRs, RNA editing produces 5-HT_2C_ receptor isoforms that differ in function, such as efficiency and specificity of coupling to G_q/11_ and also pharmacology [218, 2770]. Most 5-HT receptors (except 5-ht_1e_ and 5-ht_5b_) play specific roles mediating functional responses in different tissues (reviewed by [2129, 2686]).

**Table:** 

Nomenclature	5-HT_1A_ receptor	5-HT_1B_ receptor	5-HT_1D_ receptor	5-ht_1e_ receptor	5-HT_1F_ receptor
HGNC, UniProt	*HTR1A*, P08908	*HTR1B*, P28222	*HTR1D*, P28221	*HTR1E*, P28566	*HTR1F*, P30939
Agonists	U92016A [1690], vilazodone (Partial agonist) [541], vortioxetine (Partial agonist) [127]	L-694,247 [863], naratriptan (Partial agonist) [1856], eletriptan [1856], frovatriptan [2845], zolmitriptan (Partial agonist) [1856], vortioxetine (Partial agonist) [127], rizatriptan (Partial agonist) [1856]	dihydroergotamine [924, 1482, 1492], ergotamine [829], L-694,247 [2824], naratriptan [595, 1856, 2169], zolmitriptan [1856], frovatriptan [2845], rizatriptan [1856]	BRL-54443 [296]	BRL-54443 [296], eletriptan [1856], sumatriptan [15, 16, 1856, 2705]
Selective agonists	8-OH-DPAT [553, 925, 1204, 1470, 1736, 1885, 1887, 1888], NLX-101 [1886]	CP94253 [1316]	PNU109291 [658] – Gorilla, eletriptan [1856]	–	lasmiditan [1873], LY334370 [2705], 5-BODMT [1306], LY344864 [2042]
Antagonists	(S)-UH 301 (p*K*_i_ 7.9) [1885]	–	–	–	–
Selective antagonists	WAY-100635 (p*K*_i_ 7.9–9.2) [1885, 1887], robalzotan (p*K*_i_ 9.2) [1177]	SB 224289 (Inverse agonist) (p*K*_i_ 8.2–8.6) [782, 1883, 2336], SB236057 (Inverse agonist) (p*K*_i_ 8.2) [1728], GR-55562 (p*K*_B_ 7.4) [1064]	SB 714786 (p*K*_i_ 9.1) [2742]	–	–
Labelled ligands	[^3^H]robalzotan (Antagonist) (p*K*_d_ 9.8) [1163], [^3^H]WAY100635 (Antagonist) (p*K*_d_ 9.5) [1257], [^3^H]8-OH-DPAT (Agonist) [211, 1204, 1884, 1887], [^3^H]NLX-112 (Agonist) [1007], [^11^C]WAY100635 (Antagonist) [2620], p-[^18^F]MPPF (Antagonist) [490]	[^3^H]N-methyl-AZ10419369 (Agonist, Partial agonist) [1617], [^3^H]GR 125,743 (Selective Antagonist) (p*K*_d_ 8.6–9.2) [863, 2834], [^3^H]alniditan (Agonist) [1482], [^125^I]GTI (Agonist) [253, 303] – Rat, [^3^H]eletriptan (Agonist, Partial agonist) [1856], [^3^H]sumatriptan (Agonist, Partial agonist) [1856], [^11^C]AZ10419369 (Agonist, Partial agonist) [2673]	[^3^H]eletriptan (Agonist) [1856], [^3^H] alniditan (Agonist) [1482], [^125^I]GTI (Selective Agonist) [253, 303] – Rat, [^3^H] GR 125,743 (Selective Antagonist) (p*K*_d_ 8.6) [2834], [^3^H]sumatriptan (Agonist) [1856]	[^3^H]5-HT (Agonist) [1686, 1988]	[^3^H]LY334370 (Agonist) [2705], [^125^I]LSD (Agonist) [55] – Mouse

**Table:** 

Nomenclature	5-HT_2A_ receptor	5-HT_2B_ receptor	5-HT_2C_ receptor
HGNC, UniProt	*HTR2A*,P28223	*HTR2B*,P41595	*HTR2C*,P28335
Agonists	DOI [268, 1872, 2403]	methysergide (Partial agonist) [1311, 2214, 2706], DOI [1386, 1872, 2287]	DOI [637, 1872, 2287], Ro 60-0175 [1286, 1311]
Selective agonists	–	BW723C86 [151, 1311, 2287], Ro 60-0175 [1311]	WAY-163909 [627], lorcaserin [2576]
Antagonists	risperidone (Inverse agonist) (p*K*_i_ 9.3–10) [1331, 1362, 2307], mianserin (p*K*_i_ 7.7–9.6) [1311, 1346, 1736], ziprasidone (p*K*_i_ 8.8–9.5) [1331, 1362, 2307, 2350], volinanserin (pIC_50_ 6.5–9.3) [1311, 1566, 2155], blonanserin (p*K*_i_ 9.1) [1925], clozapine (Inverse agonist) (p*K*_i_ 7.6–9) [1311, 1362, 1733, 2307, 2667], H05 (pIC_50_ 7.2) [2843]	mianserin (p*K*_i_ 7.9–8.8) [238, 1311, 2706]	mianserin (Inverse agonist) (p*K*_i_ 8.3–9.2) [707, 1311, 1736], methysergide (p*K*_i_ 8.6–9.1) [637, 1311], ziprasidone (Inverse agonist) (p*K*_i_ 7.9–9) [1000, 1362, 2350], olanzapine (Inverse agonist) (p*K*_i_ 8.1–8.4) [1000, 1362, 2350], loxapine (Inverse agonist) (p*K*_i_ 7.8–8) [1000, 1362]
Selective antagonists	compound 3b (p*K*_i_ 10.6) [703], ketanserin (p*K*_i_ 8.1–9.7) [308, 1311, 2140], pimavanserin (Inverse agonist) (p*K*_i_ 9.3) [769, 2667]	BF-1 (p*K*_i_ 10.1) [2300], RS-127445 (p*K*_i _9–9.5) [238, 1311], EGIS-7625 (p*K*_i_ 9) [1346]	FR260010 (p*K*_i_ 9) [944], SB 242084 (p*K*_i_ 8.2–9) [1250, 1311], RS-102221 (p*K*_i_ 8.3–8.4) [239, 1311]
Labelled ligands	[^3^H]fananserin (Antagonist) (p*K*_d_ 9.9) [1623] – Rat, [³H]ketanserin (Antagonist) (p*K*_d_ 8.6–9.7) [1311, 2140], [^11^C]volinanserin (Antagonist) [918], [^18^F]altanserin (Antagonist) [2210]	[^3^H]LSD (Agonist) [2140], [^3^H]5-HT (Agonist) [2704] – Rat, [^3^H]mesulergine (Antagonist, Inverse agonist) (p*K*_d_ 7.9) [1311], [^125^I]DOI (Agonist)	[^3^H]mesulergine (Antagonist, Inverse agonist) (p*K*_d_ 8.7–9.3) [707, 2140], [^125^I]DOI (Agonist) [707], [^3^H]LSD (Agonist)

**Table:** 

Nomenclature	5-HT_4_ receptor	5-HT_5A_ receptor	5-ht_5b_ receptor
HGNC, UniProt	*HTR4*,Q13639	*HTR5A*,P47898	*HTR5BP*, –
Agonists	cisapride (Partial agonist) [106, 175, 803, 1719, 1720, 2653]	–	–
Selective agonists	TD-8954 [1700], ML 10302 (Partial agonist) [187, 215, 1719, 1720, 1721], RS67506 [983] – Rat, relenopride (Partial agonist) [816], velusetrag [1562, 2411], BIMU 8 [465]	–	–
Selective antagonists	RS 100235 (p*K*_i_ 8.7–12.2) [465, 2186], SB 204070 (p*K*_i_ 9.8–10.4) [175, 1719, 1720, 2653], GR 113808 (p*K*_i_ 9.3–10.3) [106, 175, 215, 465, 1720, 2186, 2653]	SB 699551 (p*K*_i_ 8.2) [487]	–
Labelled ligands	[^3^H]GR 113808 (Antagonist) (p*K*_d_ 9.7–10.3) [106, 175, 1721, 2653], [^123^I]SB 207710 (Antagonist) (p*K*_d_ 10.1) [297] – Pig, [^3^H]RS 57639 (Selective Antagonist) (p*K*_d_ 9.7) [237] – Guinea pig, [^11^C]SB207145 (Antagonist) (p*K*_d_ 8.6) [1604]	[^125^I]LSD (Agonist) [862], [^3^H]5-CT (Agonist) [862]	[^125^I]LSD (Agonist) [1678] – Mouse, [^3^H]5-CT (Agonist) [2703] – Mouse

**Table:** 

Nomenclature	5-HT_6_ receptor	5-HT_7_ receptor
HGNC, UniProt	*HTR6*,P50406	*HTR7*,P34969
Selective agonists	WAY-181187 [2291], E6801 (Partial agonist) [1036], WAY-208466 [186], EMD-386088 [1679]	LP-12 [1476], LP-44 [1476], LP-211 [1477] – Rat, AS-19 [1279], E55888 [272]
Antagonists	–	lurasidone (p*K*_i_ 9.3) [1116], pimozide (p*K*_i_ 9.3) [2213] – Rat, vortioxetine (p*K*_i_ 6.3) [127]
Selective antagonists	SB399885 (p*K*_i_ 9) [1024], SB 271046 (p*K*_i_ 8.9) [293], cerlapirdine (p*K*_i_ 8.9) [476], SB357134 (p*K*_i_ 8.5) [294], Ro 63-0563 (p*K*_i_ 7.9–8.4) [221, 2402]	SB269970 (pKi 8.6–8.9) [2569], SB656104 (p*K*_i_ 8.7) [713], DR-4004 (p*K*_i_ 8.7) [828, 1266], JNJ-18038683 (p*K*_i_ 8.2) [234], SB 258719 (Inverse agonist) (p*K*_i_ 7.5) [2570]
Labelled ligands	[^11^C]GSK215083 (Antagonist) (p*K*_i_ 9.8) [1987], [^125^I]SB258585 (Selective Antagonist) (p*K*_d_ 9) [1024], [^3^H]LSD (Agonist) [220], [³H]Ro 63-0563 (Antagonist) (p*K*_d_ 8.3) [221], [³H]5-CT (Agonist)	[^3^H]5-CT (Agonist) [2569], [^3^H]5-HT (Agonist) [130, 2450], [^3^H] SB269970 (Selective Antagonist) (p*K*_d_ 8.9) [2569], [³H]LSD (Agonist) [2450]

**Comments**: Tabulated *pK*_i_ and *K*_D_ values refer to binding to human 5-HT receptors unless indicated otherwise. The nomenclature of 5-HT_1B_/5-HT_1D_ receptors has been revised [954]. Only the non-rodent form of the receptor was previously called 5-HT_1D_: the human 5-HT_1B_ receptor (tabulated) displays a different pharmacology to the rodent forms of the receptor due to Thr335 of the human sequence being replaced by Asn in rodent receptors [936]. Wang *et al*. (2013) report X-ray structures which reveal the binding modality of ergotamine and dihydroergotamine (DHE) to the 5-HT_1B_ receptor in comparison with the structure of the 5-HT_2B_ receptor [2719]; some of these drugs adopt rather different conformations depending on the target receptor [2017]. Various 5-HT receptors have multiple partners in addition to G proteins, which may affect function and pharmacology [1652]. NAS181 is a selective antagonist of the rodent 5-HT_1B_ receptor. Fananserin (LSD) and ketanserin bind with high affinity to dopamine D4 and histamine H_1_ receptors respectively, and ketanserin is a potent α1 adrenoceptor antagonist, in addition to blocking 5-HT_2A_ receptors. Lysergic acid (LSD) and ergotamine show a strong preference for arrestin recruitment over G protein coupling at the 5-HT_2B_ receptor, with no such preference evident at 5-HT_1B_ receptors, and they also antagonise 5-HT_7A_ receptors [2701]. DHE (dihydroergocryptine), pergolide and cabergoline also show significant preference for arrestin recruitment over G protein coupling at 5-HT_2B_ receptors [2701]. The 5-HT_2B_ (and other 5-HT) receptors interact with immunocompetent cells [1972]. The serotonin antagonist mesulergine was key to the discovery of the 5-HT_2C_ receptor [2006], initially known as 5-HT_1C_ [100]. The human5-HT_5A_ receptor may couple to several signal transduction pathways when stably expressed in C6 glioma cells [1910] and rodent prefrontal cortex (layer V pyramidal neurons) [845]. The human orthologue of the mouse 5-ht_5b_ receptor is non-functional (stop codons); the 5-ht_1e_ receptor has not been cloned from mouse, or rat, impeding definition of its function [936]. In addition to accepted receptors, an ’orphan’ receptor, unofficially termed 5-HT_1P_, has been described [808].

Further reading on 5-Hydroxytryptamine receptorsBockaertJ
 (2011) 5-HT(4) receptors, a place in the sun: act two. Curr Opin Pharmacol
11: 87–9321342787
10.1016/j.coph.2011.01.012HayesDJ
 (2011) 5-HT receptors and reward-related behaviour: a review. Neurosci Biobehav Rev
35: 1419–4921402098
10.1016/j.neubiorev.2011.03.005HoyerD
 (1994) International Union of Pharmacology classification of receptors for 5-hydroxytryptamine (Serotonin). Pharmacol Rev
46: 157–2037938165
LeopoldoM
 (2011) Serotonin 5-HT7 receptor agents: Structure-activity relationships and potential therapeutic applications in central nervous system disorders. Pharmacol Ther
129: 120–4820923682
10.1016/j.pharmthera.2010.08.013PMC3031120MeltzerHY
 (2011) The role of serotonin receptors in the action of atypical antipsychotic drugs. Curr Opin Pharmacol
11: 59–6721420906
10.1016/j.coph.2011.02.007RobertsAJ
 (2012) The 5-HT(7) receptor in learning and memory. Hippocampus
22: 762–7121484935
10.1002/hipo.20938PMC3310936

## Acetylcholine receptors (muscarinic)

G protein-coupled receptors → Acetylcholine receptors (muscarinic)

**Overview**: Muscarinic acetylcholine receptors (mAChRs) (**nomenclature as agreed by the NC-IUPHAR Subcommittee on Muscarinic Acetylcholine Receptors** [364]) are activated by the endogenous agonist acetylcholine. All five (M1-M5) mAChRs are ubiquitously expressed in the human body and are therefore attractive targets for many disorders. Functionally, M_1_, M_3_, and M_5_ mAChRs preferentially couple to G_q/11_ proteins, whilst M_2_ and M_4_ mAChRs predominantly couple to G_i/o_ proteins. Both agonists and antagonists of mAChRs are clinically approved drugs, including pilocarpine for the treatment of elevated intra-ocular pressure and glaucoma, and atropine for the treatment of bradycardia and poisoning by muscarinic agents such as organophosphates. Of note, it has been observed that mAChRs dimerise reversibly [998] and that dimerisation/oligomerisation can be affected by ligands [1506, 1656].

**Table:** 

Nomenclature	M_1_ receptor	M_2_ receptor
HGNC, UniProt	*CHRM1*, P11229	*CHRM2*, P08172
Endogenous agonists	acetylcholine [1143, 1252]	acetylcholine [419, 1143, 1252]
Agonists	xanomeline (Partial agonist) [452, 2077, 2751, 2806], methacholine [2008, 2178] – Rat, arecoline [1143, 1969, 2178], oxotremorine (Partial agonist) [1143, 2178], carbachol [452, 1143, 2806], pilocarpine (Partial agonist) [1143, 2178], bethanechol [1143, 2178], iperoxo [2308]	iperoxo [2308, 2309], xanomeline [2077, 2751, 2806], methacholine [2008, 2178] – Rat, oxotremorine [1143, 2178], arecoline [1143, 1969, 2178], pilocarpine (Partial agonist) [1143, 2178], bethanechol [1143, 2178]
Antagonists	tiotropium (p*K*_i_ 9.6–10.7) [589, 2080, 2508, 2551], aclidinium (pIC_50_ 10.1–10.2) [2080, 2551], glycopyrrolate (pIC_50_ 9.6–10.1) [2462, 2508], ipratropium (p*K*_i _9.3–9.8) [1021, 2080], atropine (p*K*_i_ 8.5–9.6) [452, 742, 1021, 1075, 2019, 2410], biperiden (p*K*_d_ 9.3) [227], 4-DAMP (p*K*_i_ 9.3) [606], darifenacin (p*K*_i_ 8.9–9.1) [819, 1021, 2396], scopolamine (p*K*_i_ 9) [509, 1075], oxybutynin (p*K*_i_ 8.6) [559, 2396], tolterodine (p*K*_i_ 8.4–8.5) [819, 2396], droxidopa (p*K*_i_ 7.1) [509]	tiotropium (p*K*_i_ 9.9–10.7) [589, 2080, 2508, 2551], aclidinium (pIC_50_ 10.1) [2080, 2551], ipratropium (p*K*_i_ 9.3–9.8) [1021, 2080], glycopyrrolate (pIC_50_ 8.7–9.5) [2462, 2508], atropine (p*K*_i_ 7.8– 9.2) [509, 1021, 1075, 2019], scopolamine (p*K*_i_ 8.7) [227, 1075], tolterodine (Inverse agonist) (p*K*_i_ 8.4–8.5) [819, 2396], 4-DAMP (p*K*_i_ 8.4) [606], biperiden (p*K*_d_ 8.2) [227], oxybutynin (p*K*_i_ 7.9–8.1) [559, 2396], darifenacin (Inverse agonist) (p*K*_i_ 7.2–7.3) [819, 1021, 2396], tropicamide (p*K*_i_ 7.2) [509]
Selective antagonists	pirenzepine (p*K*_i_ 7.6–8.3) [311, 606, 982, 1075, 1181, 2771], VU0255035 (p*K*_i_ 7.8) [2356]	AFDX384 (p*K*_i_ 8.1–8.2) [509, 606]
Allosteric modulators (Positive)	benzoquinazolinone 12 (p*K*_B_ 6.6) [6], KT 5720 (p*K*_d_ 6.4) [1427], brucine (p*K*_d_ 4.5–5.8) [203, 1143, 1426], BQCA (p*K*_B_ 4–4.8) [6, 7, 341, 1590], VU0029767 [1654], VU0090157 [1654]	LY2119620 (p*K*_d_ 5.5–5.7) [510, 1364], LY2033298 (pKd 4.4) [2646]
Allosteric modulators (Negative)	muscarinic toxin 7 (p*K*_i_ 11–11.1) [742, 1858, 1953]	gallamine (p*K*_i_ 5.8–7.6) [1347, 1780, 2609], W-84 (p*K*_d_ 6–7.5) [1762, 2609], C7/3-phth (p*K*_d_ 7.1) [98, 453], alcuronium (p*K*_d_ 6.1–6.9) [98, 1143, 2609], gallamine (p*K*_d_ 5.9–6.3) [466, 1424]
Labelled ligands	[^3^H]QNB (Antagonist) (p*K*_d_ 10.6–10.8) [1144, 2019], [^3^H]N-methyl scopolamine (Antagonist) (p*K*_d_ 9.4–10.3) [370, 452, 454, 1021, 1143, 1145, 1181, 1256, 1424], [^3^H]darifenacin (Selective Antagonist) (p*K*_d_ 8.8) [2410], [^3^H]iperoxo (Agonist) [2308], [^3^H]pirenzepine (Selective Antagonist) (p*K*_d_ 7.9) [374, 2284, 2651, 2752], [^3^H]acetylcholine (Agonist)	[^3^H]QNB (Antagonist) (p*K*_d _10.1–10.6) [1144, 2019], [^3^H]iperoxo (Agonist) [2308], [^3^H]N-methyl scopolamine (Antagonist) (p*K*_d_ 9.3–9.9) [370, 1021, 1144, 1145, 1256, 1424, 2736], [³H]AF DX-384 (Selective Antagonist) (p*K*_d_ 9) [374, 1742, 2651], [³H] acetylcholine (Agonist) [1425]
Comments	Atypical agonists: AC-42 [99, 1409, 1410, 2256, 2439, 2440], 77-LH-28-1 [99, 1409], N-desmethylclozapine [2256, 2439, 2495], TBPB [1184, 1252, 2256], McN-A-343 [1143, 2178]	Atypical agonists: AC-42 [1409, 1682], 77-LH-28-1 [1409, 1682], N-desmethylclozapine [2495], McN-A-343 [1143, 1682, 2178]

**Table:** 

Nomenclature	M_3_ receptor	M_4_ receptor	M_5_ receptor
HGNC, UniProt	*CHRM3*, P20309	*CHRM4*, P08173	*CHRM5*, P08912
Endogenous agonists	acetylcholine [419, 1143, 1252]	acetylcholine [1143, 1252]	acetylcholine [419]
Agonists	xanomeline (Partial agonist) [2077, 2751, 2806], methacholine [2008, 2178] – Rat, arecoline [1143, 1969, 2178], oxotremorine [1143, 2178], pilocarpine (Partial agonist) [1143, 2178], carbachol [419, 1143, 2806], bethanechol [1143, 2178], iperoxo [2308]	xanomeline (Partial agonist) [1694, 2077, 2751, 2806], methacholine [2008, 2178] – Rat, arecoline [1143, 1969, 2178], oxotremorine [1143, 2178], pilocarpine (Partial agonist) [1143, 2178], carbachol [1143, 2806], bethanechol [1143, 2178], iperoxo [2308]	xanomeline (Partial agonist) [866, 2077, 2751, 2806], pilocarpine (Partial agonist) [184, 599, 866], carbachol [184, 866, 2806], arecoline [1969, 2178], bethanechol [2178], iperoxo [2308], methacholine [2178]
Antagonists	tiotropium (pK_i_ 9.5–11.1) [589, 610, 2080, 2508, 2551], aclidinium (pK_i_ 10.1–10.2) [2080, 2551], atropine (pK_i_ 8.5–9.8) [509, 1021, 1075, 2019], glycopyrrolate (pIC_50_ 9.6–9.8) [2462, 2508], ipratropium (pK_i_ 9.3–9.8) [610, 1021, 2080], scopolamine (pK_i_ 9.4) [227, 1075], 4-DAMP (pK_i_ 9.3) [606], darifenacin (pK_i_ 8.9–9.1) [819, 1021, 2396], oxybutynin (pK_i_ 8.8) [559, 2396], tolterodine (pK_i_ 8.4–8.5) [819, 2396], biperiden (pK_d_ 8.4) [227], tropicamide (pK_i_ 7) [509]	tiotropium (pK_i_ 10.2–10.6) [2508, 2551], aclidinium (pK_i_ 10) [2551], glycopyrrolate (pK_i_ 9.1–10) [2462, 2508], atropine (pK_i_ 8.7–9.5) [509, 1021, 1075, 2019], scopolamine (pK_i_ 9.1–9.5) [227, 1075], ipratropium (pK_i_ 9.2) [1021], 4-DAMP (pK_i_ 8.9) [606], oxybutynin (pK_i_ 8.4–8.7) [559, 2396], biperiden (pK_d_ 8.6) [227], tolterodine (pK_i_ 8.3–8.4) [819, 2396], darifenacin (pK_i_ 7.3–8.1) [819, 1021, 2396], tropicamide (pK_i_ 6.9) [352]	tiotropium (pK_i_ 9.8–10.2) [2508, 2551], aclidinium (pK_i_ 9.9) [2551], glycopyrrolate (pK_i_ 8.9–9.9) [2462, 2508], atropine (pK_i_ 8.3–9.3) [509, 1021, 1803], 4-DAMP (pK_i_ 9) [606], ipratropium (pK_i_ 8.8) [1021], tolterodine (pK_i_ 8.5–8.8) [819, 2396], scopolamine (pK_i_ 8.7) [227], darifenacin (pK_i_ 7.9–8.6) [819, 1021, 2396], biperiden (pK_d_ 8.2) [227], oxybutynin (pK_i_ 7.9) [559, 2396], tropicamide (pK_i_ 6.4) [509]
Selective antagonists	–	PCS1055 (pK_i_ 8.2) [509], AFDX384 (pK_i_ 7.3–8) [509, 606], PD 102807 (pKi 7.4–7.6) [509, 1954]	ML381 (pK_i_ 6.3) [797]
Allosteric modulators (Positive)	WIN 62,577 (pK_d_ 5.1) [1428], N-chloromethyl-brucine (pK_d_ 3.3) [1426]	VU0152100 (pEC_50_ 6.4) [265] – Rat, VU0152099 (pEC_50_ 6.4) [265] – Rat, LY2033298 (pK_B_ 4.9–5.5) [381, 2495], LY2119620 (pKd 5.5) [510], thiochrome (pKd 4) [1425]	amiodarone (pK_B_ 7.2) [2448], ML380 (pK_B_ 4.8) [184, 799]
Allosteric modulators (Negative)	–	muscarinic toxin 3 (pK_i_ 8.7) [1181, 1952]	–
Selective allosteric modulators	–	–	ML375 (Negative) (pKB 6.2–6.6) [184, 319, 798]
Labelled ligands	[^3^H]QNB (Antagonist) (pK_d_ 10.4) [1144, 2019], [^3^H]N-methyl scopolamine (Antagonist) (pK_d_ 9.7–10.2) [370, 1021, 1143, 1144, 1256, 1424], [^3^H]darifenacin (Selective Antagonist) (pK_d_ 9.5) [2410], [^3^H]4-DAMP (Selective Antagonist) (pK_i_ 8.8–9.4) [374, 1162], [^3^H]iperoxo (Agonist) [2308], [^3^H]acetylcholine (Agonist)	[^3^H]QNB (Antagonist) [^3^H]QNB (Antagonist) (pK_d_ 9.7–10.5) [1144, 2018], [^3^H]N-methyl scopolamine (Antagonist) (pK_d_ 9.9–10.2) [370, 1143, 1144, 1256, 1424, 2736], [^3^H]iperoxo (Agonist) [2308], [^3^H]AF DX-384 (Selective Antagonist) (pK_d_ 8.7) [374, 1742, 2651], [^3^H]acetylcholine (Agonist) [1425]	[^3^H]QNB (Antagonist) (pK_d_ 10.2–10.7) [1144], [^3^H]N-methyl scopolamine (Antagonist) (pK_d_ 9.3–9.7) [370, 419, 1021, 1144, 1256, 2699, 2736], [^3^H]iperoxo (Agonist) [2308], [^3^H] acetylcholine (Agonist)
Comments	Atypical agonists: AC-42 [1409], 77-LH-28-1 [1409], N-desmethylclozapine [2495], McN-A-343 [1143, 2178]	Atypical agonists: AC-42 [1409], 77-LH-28-1 [1409], N-desmethylclozapine [2495], McN-A-343 [1143, 2178]	Atypical agonists: AC-42 [1409], 77-LH-28-1 [1409], McN-A-343 [2178]

**Comments**: Atomic structures for all five mAChRs bound to antagonists have been determined [910, 2494, 2559, 2699, 2876]. Structures of agonist-bound M_1_, M_2_, M_3_, and M_4_ mAChRs [1364, 2726, 2840, 2929] and β_2_-arrestin-bound M_2_ mAChR have been reported [2451]. These structures show that the orthosteric binding site of this family of receptor is absolutely conserved and, as a consequence, explain why highly selective orthosteric ligand binding to any specific mAChR has been notoriously difficult to achieve. As such, it is common to assess the rank order of affinity for a range of antagonists with limited selectivity (*e.g*., 4-DAMP, darifenacin, pirenzepine, AFDX384) to identify the involvement of particular subtypes- although caution should be used in the design and interpretation of such experiments due to the lack of absolute ligand subtype selectivity [1839]. Some ligands may display selectivity at the level of function (*e.g*., xanomeline) or binding kinetics (*e.g*., tiotropium) [2077, 2351, 2553]. In addition, structures of the M_3_ and M_4_ mAChR DREADDS (designer receptors exclusively activated by designer drugs) have been reported providing insights into orthosteric ligand selectivity for these chemogenetic tools [2929].

Structures of the M_1_ and M_2_ and M_4_ mAChRs in complex with allosteric modulators [1364, 1611, 2726] have validated numerous pharmacological studies that indicated the presence of a common mAChR allosteric site located at the extracellular entrance to these receptors. In addition, a structure of the M_1_ mAChR with muscarinic toxin 7 (MT7) bound to the common allosteric site has provided insight into the extreme subtype selectivity of MT7 [1611]. Allosteric ligands proposed to bind to this common allosteric site include gallamine, strychnine, C_7_/3-phth, brucine and LY2033298. Additionally, a second allosteric site has been proposed on the mAChRs based on pharmacological analyses of the actions of compounds such as KT 5720, WIN 62,577, WIN 51,708, staurosporine and amiodarone [319, 1427, 1428, 2448]. In the presence of the orthosteric ligand, allosteric modulators can exert positive, negative, or neutral cooperativity with that ligand. Direct receptor activation *via* an allosteric site has been reported for a number of allosteric ligands of the mAChRs [540, 1438, 1441, 1590, 1861, 1862]. ‘Atypical agonists’ are ligands that have been suggested to have bitopic binding modes for at least one subtype whereby the agonist occupies both the orthosteric and allosteric sites [99, 1251, 2647]. Several mAChR PET radioligands have been reported, but their utility for subtype selectivity measurements in the human brain has yet to be confirmed [1968].

Further reading on Acetylcholine receptors (muscarinic)BurgerWAC
 (2018) Toward an understanding of the structural basis of allostery in muscarinic acetylcholine receptors. J Gen Physiol
150: 1360–137230190312
10.1085/jgp.201711979PMC6168235CaulfieldMP
 (1998) International Union of Pharmacology. XVII. Classification of muscarinic acetylcholine receptors. Pharmacol Rev
50: 279–909647869
EglenRM. (2012) Overview of muscarinic receptor subtypes. Handb Exp Pharmacol
3–2810.1007/978-3-642-23274-9_122222692KruseAC
 (2014) Muscarinic acetylcholine receptors: novel opportunities for drug development. Nat Rev Drug Discov
13: 549–6024903776
10.1038/nrd4295PMC5818261LeachK
 (2012) Structure-function studies of muscarinic acetylcholine receptors. Handb Exp Pharmacol
29–4822222693
10.1007/978-3-642-23274-9_2ValantC
 (2012) The best of both worlds? Bitopic orthosteric/allosteric ligands of g protein-coupled receptors. Annu Rev Pharmacol Toxicol
52: 153–7821910627
10.1146/annurev-pharmtox-010611-134514

## Adenosine receptors

G protein-coupled receptors → Adenosine receptors

**Overview**: Adenosine receptors (**nomenclature as agreed by the NC-IUPHAR Subcommittee on Adenosine Receptors** [729]) are activated by the endogenous ligand adenosine (potentially inosine also at A_3_ receptors). Crystal structures for the antagonist-bound [480, 1129, 1553, 2334], agonist-bound [1446, 1447, 2839] and G protein-bound A_2A_ adenosine receptors [351] have been described. The structures of an antagonist-bound A_1_ receptor [830] and an adenosine-bound A_1_ receptor-G_i_ complex [612] have been resolved by cryo-electronmicroscopy. Another structure of an antagonist-bound A_1_ receptor obtained with X-ray crystallography has also been reported [422]. The structure of the A_2B_ receptor has also been elucidated [413]. Caffeine is a nonselective antagonist for adenosine receptors, while istradefylline, a selective A_2A_ receptor antagonist, is on the market for the treatment of Parkinson’s disease.

**Table:** 

Nomenclature	A_1_ receptor	A_2A_ receptor	A_2B_ receptor	A_3_ receptor
HGNC, UniProt	*ADORA1*, P30542	*ADORA2A*, P29274	*ADORA2B*, P29275	*ADORA3*, P0DMS8
Endogenous agonists	adenosine [2859]	adenosine [727, 728, 2859]	adenosine [727, 728, 2859]	adenosine [727, 728, 2859]
Agonists	NECA [767, 1175, 2190, 2607, 2859]	NECA [248, 588, 767, 1274, 1378, 2859]	NECA [194, 248, 1164, 1529, 2458, 2669, 2859]	NECA [248, 767, 1137, 2255, 2670, 2859]
Selective agonists	cyclopentyladenosine [518, 548, 767, 989, 1134, 1175, 2190], 5-Cl-5-deoxy-(±)-ENBA [722], TCPA [196], CCPA [1134, 1923], MRS7469 [2602]	apadenoson [2009], UK-432,097 [900, 2839], compound 4g [480], CGS 21680 [248, 588, 767, 1134, 1274, 1308, 1378, 1923], regadenoson [1134]	BAY 60-6583 [631]	piclidenoson [691, 756, 1308, 2670], Cl-IB-MECA [266, 1137, 1271], MRS5698 [2601]
Antagonists	CGS 15943 (p*K*_i_ 8.5) [1957], xanthine amine congener (p*K*_d_ 7.5) [722]	CGS 15943 (p*K*_i_ 7.7–9.4) [588, 1274, 1308, 1957], xanthine amine congener (p*K*_i_ 8.4–9) [588, 1308]	xanthine amine congener (p*K*_i_ 6.9–8.8) [194, 1164, 1165, 1308, 1529, 2458], CGS 15943 (p*K*_i_ 6–8.1) [89, 1164, 1165, 1308, 1957, 2458]	CGS 15943 (p*K*_i_ 7–7.9) [1281, 1308, 1957, 2670], xanthine amine congener (p*K*_i_ 7–7.4) [1308, 2255, 2670]
Selective antagonists	PSB36 (p*K*_i_ 9.9) [8] – Rat, DPCPX (p*K*_i_ 7.4–9.2) [548, 1111, 1923, 2190, 2775], derenofylline (p*K*_i_ 9) [1205], WRC-0571 (p*K*_i_ 8.8) [1659], DU172 (p*K*_i_ 7.4) [830]	SCH442416 (p*K*_i_ 8.4–10.3) [2372, 2589], ZM-241385 (p*K*_i_ 8.8–9.1) [1957]	PSB-0788 (p*K*_i_ 9.4) [247], PSB603 (p*K*_i_ 9.3) [247], MRS1754 (p*K*_i_ 8.8) [1164, 1280], PSB1115 (p*K*_i_ 7.3) [972]	MRS1220 (p*K*_i_ 8.2–9.2) [1137, 1281, 2484, 2878], VUF5574 (p*K*_i_ 8.4) [2657], MRS1523 (p*K*_i_ 7.7) [1493], MRS1191 (p*K*_i_ 7.5) [1137, 1168, 1507]
Allosteric modulators (Positive)	PD81723 [305]	–	–	LUF6000 [834], LUF6096 [988], MRS8054 [676]
Labelled ligands	[^3^H]CCPA (Agonist) [1308, 2190], [^3^H]DPCPX (Antagonist) (p*K*_d_ 8.4–9.2) [518, 691, 1308, 1957, 2190, 2607]	[^3^H]ZM 241385 (Antagonist) (p*K*_d_ 8.7–9.1) [44, 765], [^3^H]CGS 21680 (Agonist) [1152, 2716]	[^3^H]MRS1754 (Antagonist) (p*K*_d_ 9.8) [1164]	[^125^I]AB-MECA (Agonist) [1957, 2670]

**Comments**: Adenosine inhibits many intracellular ATP-utilising enzymes, including adenylyl cyclase (P-site). A pseudogene exists for the A_2B_ adenosine receptor (*ADORA2BP1*) with 79% identity to the A_2B_ adenosine receptor cDNA coding sequence, but which is unable to encode a functional receptor [1138]. DPCPX also exhibits antagonism at A_2B_ receptors (p*K*_i_ ca. 7,[42, 1308]). Antagonists at A_3_ receptors exhibit marked species differences, such that only MRS1523 and MRS1191 are selective at the rat A_3_ receptor. In the absence of other adenosine receptors, [^3^H]DPCPX and [^3^H]ZM 241385 can also be used to label A_2B_ receptors (K_D_ *ca*. 30 and 60 nM respectively). [^125^I]AB-MECA also binds to A_1_ receptors [1308]. [^3^H]CGS 21680 is relatively selective for A_2A_ receptors, but may also bind to other sites in cerebral cortex [512, 1176]. [^3^H]NECA binds to other non-receptor elements, which also recognise adenosine [1567]. XAC-BY630 has been described as a fluorescent antagonist for labelling A_1_ adenosine receptors in living cells, although activity at other adenosine receptors was not examined [278].

Further reading on Adenosine receptorsBoreaPA
 (2015) The A3 adenosine receptor: history and perspectives. Pharmacol Rev
67: 74–10225387804
10.1124/pr.113.008540CronsteinBN
 (2017) Adenosine and adenosine receptors in the pathogenesis and treatment of rheumatic diseases. Nat Rev Rheumatol
13: 41–5127829671
10.1038/nrrheum.2016.178PMC5173391FredholmBB
 (2011) International Union of Basic and Clinical Pharmacology. LXXXI. Nomenclature and classification of adenosine receptors–an update. Pharmacol Rev
63: 1–3421303899
10.1124/pr.110.003285PMC3061413GuoD
 (2017) Kinetic Aspects of the Interaction between Ligand and G Protein-Coupled Receptor: The Case of the Adenosine Receptors. Chem Rev
117: 38–6627088232
10.1021/acs.chemrev.6b00025GöblyösA
 (2011) Allosteric modulation of adenosine receptors. Biochim Biophys Acta
1808: 1309–1820599682
10.1016/j.bbamem.2010.06.013IJzermanAP
 (2022) International Union of Basic and Clinical Pharmacology. CXII: Adenosine Receptors: A Further Update. Pharmacol Rev
74: 340–37235302044
10.1124/pharmrev.121.000445PMC8973513JacobsonKA
 (2020) Adenosine A_2A_ receptor antagonists: from caffeine to selective non-xanthines. Br J Pharmacol10.1111/bph.15103PMC925183132424811MundellS
 (2011) Adenosine receptor desensitization and trafficking. Biochim Biophys Acta
1808: 1319–2820550943
10.1016/j.bbamem.2010.06.007VecchioEA
 (2018) New paradigms in adenosine receptor pharmacology: allostery, oligomerization and biased agonism. Br J Pharmacol
175: 4036–404629679502
10.1111/bph.14337PMC6177620WeiCJ
 (2011) Normal and abnormal functions of adenosine receptors in the central nervous system revealed by genetic knockout studies. Biochim Biophys Acta
1808: 1358–7921185258
10.1016/j.bbamem.2010.12.018

## Adhesion Class GPCRs

G protein-coupled receptors → Adhesion Class GPCRs

**Overview**: Adhesion GPCRs are structurally identified on the basis of a large extracellular region, similar to the Class B GPCR, but which is linked to the 7TM region by a GPCR autoproteolysis-inducing (GAIN) domain [69] containing a GPCR proteolysis site (GPS). The N-terminal extracellular region often shares structural homology with adhesive domains (e.g. cadherins, immunolobulin, lectins) facilitating inter- and matricellular interactions and leading to the term adhesion GPCR [731, 2894]. Several receptors have been suggested to function as mechanosensors [259, 2035, 2305, 2789]. Cryo-EM structures of the 7-transmembrane domain of several adhesion GPCRs have been determined recently [136, 1525, 2053, 2054, 2107, 2110, 2833, 2956]. **The nomenclature of these receptors was revised in 2015 as recommended by NC-IUPHAR and the Adhesion GPCR Consortium** [922].

**Table:** 

Nomenclature	ADGRA1	ADGRA2	ADGRA3
HGNC, UniProt	*ADGRA1*, Q86SQ6	*ADGRA2*, Q96PE1	*ADGRA3*, Q8IWK6
Comments	–	Required to assemble higher-order Reck/Gpr124/Frizzled/Lrp5/6 complexes [667, 2072, 2650, 2666, 2950]. Interacts with Reck [436, 667, 2666], Syndecan-1, -2 [440], Integrin-αvβ3 [2649] and heparin [2649]. Principal signal transduction involves Dishevelled [667], β-catenin [2072] and Cdc42 [384]. Required for CNS vascularization and blood-brain barrier formation [384, 1658, 2666].	ADGRA3 is a stem and progenitor cell marker in the male reproductive tract [2326]. It controls fluid homeostasis, sperm maturation and storage in the male reproductive tract [1918]. Principal signal transduction involves Dishevelled [1500].

**Table:** 

Nomenclature	ADGRB1	ADGRB2	ADGRB3	CELSR1	CELSR2
Systematic nomenclature	–	–	–	ADGRC1	ADGRC2
HGNC, UniProt	*ADGRB1*, O14514	*ADGRB2*, O60241	*ADGRB3*, O60242	*CELSR1*, Q9NYQ6	*CELSR2*, Q9HCU4
Endogenous agonists	phosphatidylserine [1986]	–	–	–	–
Comments	Reported to mediate phagocytosis through binding of phosphatidylserine [1986] and lipopolysaccharide [524]. Suppresses medulloblastoma formation [2951] and is involved in dendrite development [625]. A recent study disputes the previously reported expression of ADGRB1 by macrophages [1068].	Principal signal transduction involves Gα_z_ [2099]. A R1465W mutation confers increased coupling to Gα_i_[2099].	Reported to bind C1q-like molecules [231]. Promotes myoblast fusion in vertebrates [927].	Principal signal transduction involves Rho kinase [1903]. Interacts with Vangl-2 [571, 1467], Frizzled-6 [571] and LRRK2 [2252].	Mutated in Joubert syndrome patients [2685]. Signal transduction is potentially mediated through Gα_q/11_ [2365]. Interacts homomerically with CELSR2/ADGRC2 [2365].

**Table:** 

Nomenclature	CELSR3	ADGRD1	ADGRD2	ADGRE1
Systematic nomenclature	ADGRC3	–	–	–
HGNC, UniProt	*CELSR3*, Q9NYQ7	*ADGRD1*, Q6QNK2	*ADGRD2*, Q7Z7M1	*ADGRE1*, Q14246
Endogenous agonists	–	Peptides derived from the Stachel sequence: THLTNFAILMQVV; PLXDC2 is an activating ligand for mouse ADGRD1. [199, 1513]	–	–
Comments	High-confidence risk gene for Tourette syndrome [2735]. Signal transduction is potentially mediated through Gα_q/11_ [2365]. Interacts with Frizzled-3 [2558], Dystroglycan [1530] and homomerically with CELSR3/ADGRC3 [2365].	Is a G_s_ protein-coupled receptor [223, 1513] and highly expressed in glioblastoma [149]. Couples also to G_i_ proteins [1514]. Strong association with body height [1275, 1283, 2596]. Associated with bone mineral density [2243]. Regulates oviductal fluid flow in mice [199]. The cryo-EM structure of the 7-helix transmembrane domain with its intramolecular agonist has been determined [2054, 2110].	–	–

**Table:** 

Nomenclature	ADGRE2	ADGRE3	ADGRE4P	ADGRE5
HGNC, UniProt	*ADGRE2*, Q9UHX3	*ADGRE3*, Q9BY15	*ADGRE4P*, Q86SQ3	*ADGRE5*, P48960
Comments	A mutation destabilizing the GAIN domain sensitizes mast cells to IgE-independent vibration-induced degranulation [259]. Reported to bind chondroitin sulfate B [2447]. Principal signal transduction involves G protein-coupling [197] and the phospholipase C pathway [1112]. Interacts with FHR1 [1112].	–	–	Reported to bind CD55 [923], chondroitin sulfate B [2447], α_5_β_1_ and α_γ_β_3_ integrins [2737], and CD90 [2718]. Expression levels on leukocytes are regulated by shear stress-dependent interaction with CD55 on red blood cells [1220]. Promotes the retention of blood-exposed dendritic cells in the spleen by interaction with CD55 on red blood cells [1546].

**Table:** 

Nomenclature	ADGRF1	ADGRF2	ADGRF3	ADGRF4
HGNC, UniProt	*ADGRF1*, Q5T601	*ADGRF2P*, Q8IZF7	*ADGRF3*, Q8IZF5	*ADGRF4*, Q8IZF3
Endogenous agonists	Peptides derived from the Stachel sequence TSFSI LMS PFV PST IFP VVKWIT [563, 2467]	–	–	Peptides derived from the ADGRF5 (GPR116) Stachel sequence: TSFSILMSPDSPD [563]
Comments	N-Docosahexaenoylethanolamine is an agonist at ADGRF1 supporting neurogenesis [1456] and couples to G_s_ and G_q_ pathways [563, 2467]. The cryo-EM structure of the 7-helix transmembrane domain with its intramolecular agonist has been determined [2110, 2956]. Furthermore, the domain structure of the N terminus (SEA, HormR, GAIN) has been solved by X-ray crystallography [2722].	ADGRF2 is highly expressed in squamous epithelia and gene deficiency did not result in detectable defects [2089].	ADGRF3 is highly expressed in gastrointestinal neuroendocrine tumors [353].	ADGRF4 couples to G_q/11_ proteins [563], is highly expressed in squamous epithelia and gene deficiency did not result in detectable defects [2089]. ADGRF4 is required for enamel mineralization mediated by ameloblasts [429].

**Table:** 

Nomenclature	ADGRF5	ADGRG1
HGNC, UniProt	*ADGRF5*, Q8IZF2	*ADGRG1*, Q9Y653
Endogenous agonists	Peptides derived from the Stachel sequence: TSFSILMSPDSPD [563]	Peptides derived from the Stachel sequence: TYFAVLM [2467]
Comments	ADGRF5 controls alveolar surfactant secretion via G_q/11_ pathway [299, 563, 1310, 2546]. ADGRF5 deficiency leads to dysregulation of lung surfactant homeostasis [279, 749, 2870].	ADGRG1 is a collagen-responsive platelet receptor sensing shear forces [2884]. Reported to bind tissue transglutaminase 2 [2841] and collagen, which activates the G_12/13_ pathway [1581]. Interacts with heparin [428]. Couples to G13 proteins [2467]. 3-α-acetoxydihydrodeoxygedunin is a partial agonist [2468], dihydromunduletone, a rotenoid derivative, is an antagonist [2466]. Negatively regulates immediate effector functions in human NK cells [383]. Deficiency leads to dysregulation of central and peripheral myelination [11, 814] and ADGRG1 deficiency in humans lead to bilateral frontoparietal polymicrogyria [2044]. The cryo-EM structure of the 7-helix transmembrane domain with its intramolecular agonist has been determined [136].

**Table:** 

Nomenclature	ADGRG2	ADGRG3	ADGRG4
HGNC, UniProt	*ADGRG2*, Q8IZP9	*ADGRG3*, Q86Y34	*ADGRG4*, Q8IZF6
Endogenous agonists	Peptides derived from the Stachel sequence: TSFGVL LD LSR TSLPP [562]	–	–
Comments	ADGRG2 is coupled to G_q_ and G_s_ pathways [562, 2922] and gene deficiency causes congenital obstructive azoospermia [1994]. The cryo-EM structure of the 7-helix transmembrane domain with its intramolecular agonist has been determined [1525, 2833].	ADGRG3 couples to G_o_ proteins [902], Gα_s_ and Gα_o/i_ signaling [1067]. Binds to exogenous ligands beclomethasone dipropionate and cortisol [902, 2053], and the 7-helix transmembrane domain structure has been determined by cryo-EM [2053].	ADGRG4 is highly expressed in enterochromaffin cells and gastrointestinal neuroendocrine tumors [1469]. The cryo-EM structure of the 7-helix transmembrane domain with its intramolecular agonist has been determined [2833].

**Table:** 

Nomenclature	ADGRG5	ADGRG6
HGNC, UniProt	*ADGRG5*, Q8IZF4	*ADGRG6*, Q86SQ4
Endogenous agonists	Peptides derived from the Stachel sequence: TYFAV LMQ LSG DPV PAEL [2466, 2789]	Peptides derived from the Stachel sequence: THFGV LMD LPR SASQL; Progesterone and 17-hydroxyprogesterone seem to activate Gi signaling via GPR126 (ADGRG6). [56, 1513]
Comments	ADGRG5 is a constitutively active G_s_ protein-coupled receptor [902, 2466, 2789]. Dihydromunduletone is an antagonist [2466]. The cryo-EM structure of the 7-helix transmembrane domain with its intramolecular agonist has been determined [2054].	ADGRG6 is a key regulator of Schwann cell-mediated myelination [1773], and couples to G_s_ and G_i/o_ pathways [1513, 1760, 2035]. Apomorphine hydrochloride is an exogenous agonist [263]. Binds to Laminin-211 [2035]. ADGRG6 is essential for normal differentiation of promyelinating Schwann cells and for normal myelination of axons [1760, 1773, 1774, 2035], normal placenta development [2598], and for proper heart development [1999, 2710]. Furthermore, conditional deletion of Adgrg6 revealed that this adhesion GPCR is involved in regulation of body length and bone mass [2486] and intervertebral disc function [1555]. Involved in arthrogryposis multiplex congenita (lethal congenital contracture syndrome-9) [2147].

**Table:** 

Nomenclature	ADGRG7	ADGRL1	ADGRL2	ADGRL3	ADGRL4	ADGRV1
HGNC, UniProt	*ADGRG7*, Q96K78	*ADGRL1*, O94910	*ADGRL2*, O95490	*ADGRL3*, Q9HAR2	*ADGRL4*, Q9HBW9	*ADGRV1*, Q8WXG9
Comments	ADGRG7 is expressed in intestine and involved in regulation of intestinal contractility [1890].	Couples to Gs and Gq pathways [1471, 1825]. Principal signal transduction involves Gα_s_ [1825], Gα_o_ [1471, 2122] and Gα_q_ [2122]. Interacts with Teneurin-2 [2386], FLRT-1, -3 [1921], Neurexin-1α, -1β, -2β, -3β [251], Contactin-6 [2963], Shank [1357] and TRIP8b [2064, 2065].	–	A LPHN3 gene variant in humans is associated with attention-deficit-hyperactivity disorder [72, 2792]. Principal signal transduction involves Gα_12/13_ [1667] and Gα_q_ [1667]. Interacts with Teneurin-3 [1921], FLRT-1, -3 [1921] and UNC5A [1131].	–	Loss-of-function mutations are associated with Usher syndrome, a sensory deficit disorder [1139]. Interacts with Harmonin [2160] and Whirlin [2662].

Further reading on Adhesion Class GPCRsBassilanaF
 (2019) Adhesion G protein-coupled receptors: opportunities for drug discovery. Nat Rev Drug Discov
18: 869–88431462748
10.1038/s41573-019-0039-yHamannJ
 (2015) International Union of Basic and Clinical Pharmacology. XCIV. Adhesion G protein-coupled receptors. Pharmacol Rev
67: 338–6725713288
10.1124/pr.114.009647PMC4394687LangenhanT
 (2013) Sticky signaling–adhesion class G protein-coupled receptors take the stage. Sci Signal
6: re323695165
10.1126/scisignal.2003825LiebscherI
 (2016) Tethered Agonism: A Common Activation Mechanism of Adhesion GPCRs. Handb Exp Pharmacol
234: 111–12527832486
10.1007/978-3-319-41523-9_6MonkKR
 (2015) Adhesion G Protein-Coupled Receptors: From In Vitro Pharmacology to In Vivo Mechanisms. Mol Pharmacol
88: 617–2325956432
10.1124/mol.115.098749PMC4551055PurcellRH
 (2018) Adhesion G Protein-Coupled Receptors as Drug Targets. Annu Rev Pharma col Toxicol
58: 429–44910.1146/annurev-pharmtox-010617-052933PMC716728528968187

## Adrenoceptors

G protein-coupled receptors → Adrenoceptors

**Overview: The nomenclature of the Adrenoceptors has been agreed by the NC-IUPHAR Subcommittee on Adrenoceptors** [328, 1012].

### Adrenoceptors, α_1_

The three α_1_-adrenoceptor subtypes α_1A_, α_1B_ and α_1D_ are activated by the endogenous agonists (−)-adrenaline and (−)-noradrenaline. -(-)phenylephrine, methoxamine and cirazoline are agonists and prazosin and doxazosin antagonists considered selective for α_1_- relative to α_2_-adrenoceptors. [^3^H]prazosin and [^125^I]HEAT (BE2254) are relatively selective radioligands. S(+)niguldipine also has high affinity for L-type Ca^2+^ channels. Fluorescent derivatives of prazosin (Bodipy FLprazosin- QAPB) are used to examine cellular localisation of α_1_-adrenoceptors. α_1_-Adrenoceptor agonists are used as nasal decongestants; antagonists to treat symptoms of benign prostatic hyperplasia (alfuzosin, doxazosin, terazosin, tamsulosin and silodosin, with the last two compounds being α1_A_-adrenoceptor selective and claiming to relax bladder neck tone with less hypotension); and to a lesser extent hypertension (doxazosin, terazosin). The α_1_- and β_2_-adrenoceptor antagonist carvedilol is used to treat congestive heart failure, although the contribution of α_1_-adrenoceptor blockade to the therapeutic effect is unclear. Several anti-depressants and anti-psychotic drugs are α_1_-adrenoceptor antagonists contributing to side effects such as orthostatic hypotension.

**Table:** 

Nomenclature	α_1A_-adrenoceptor	α_1B_-adrenoceptor	α_1D_-adrenoceptor
HGNC, UniProt	*ADRA1A*, P35348	*ADRA1B*, P35368	*ADRA1D*, P25100
Endogenous agonists	(−)-adrenaline [1052, 2095, 2360], (−)-noradrenaline [515, 669, 1052, 2095, 2360, 2549]	(−)-adrenaline [2360], (−)-noradrenaline [2360]	(−)-noradrenaline [1052, 2095, 2360], (−)-adrenaline [1052, 2095, 2360]
Agonists	phenylephrine [2549], methoxamine [2360, 2549]	phenylephrine [715, 1746]	methoxamine [2095, 2360, 2549], phenylephrine [2095, 2549]
Selective agonists	A61603 [715, 1309], oxymetazoline [1052, 1924, 2360, 2549], dabuzalgron [216]	–	–
Antagonists	prazosin (Inverse agonist) (p*K*_i_ 9–9.9) [382, 519, 715, 2096, 2360, 2791], doxazosin (p*K*_i_ 8.6–9.3) [931, 2096], terazosin (p*K*_i_ 7.9–8.7) [1713, 2096], phentolamine (p*K*_i_ 8.2–8.6) [2096, 2360], alfuzosin (p*K*_i_ 7.8–8.1) [1010, 2096]	prazosin (Inverse agonist) (p*K*_i_ 8.7–9.9) [715, 2096, 2360, 2791], tamsulosin (Inverse agonist) (p*K*_i_ 8.1–9.7) [715, 2096, 2360, 2791], doxazosin (p*K*_i_ 8.5–9.1) [931, 2096], alfuzosin (p*K*_i_ 7.6–8.6) [1011, 2096], terazosin (p*K*_i_ 8–8.6) [1713, 2096], phentolamine (p*K*_i_ 6.6–7.5) [2096, 2360]	prazosin (Inverse agonist) (p*K*_i_ 9.1–10.2) [715, 2096, 2360, 2791], tamsulosin (p*K*_i_ 9.2–10.2) [715, 2096, 2360, 2791], doxazosin (p*K*_i_ 8.3–9.1) [931, 2096], terazosin (p*K*_i_ 7.7–9.1) [1713, 2096], alfuzosin (p*K*_i_ 7.7–8.4) [1010, 2096], dapiprazole (p*K*_i_ 8.4) [93, 2096], phentolamine (Inverse agonist) (p*K*_i_ 6.8–8.2) [2096, 2360], RS-100329 (p*K*_i_ 7.6–7.9) [2096, 2791], labetalol (p*K*_i_ 6.1–6.6) [93, 2096]
Selective antagonists	tamsulosin (p*K*_i_ 9.7–10.7) [382, 519, 715, 2096, 2360, 2791], silodosin (p*K*_i_ 9.6–10.4) [2096, 2360], S(+)-niguldipine (p*K*_i_ 9.1–10) [715, 2096, 2360], RS-100329 (p*K*_i_ 9.6) [2096, 2791], SNAP5089 (p*K*_i_ 8.8–9.4) [1010, 1475, 2096, 2774], r-Da1a (p*K*_i_ 9.2–9.3) [1618, 2116], RS-17053 (p*K*_i_ 8.3–9.3) [382, 519, 714, 715, 2096]	Rec 15/2615 (p*K*_i_ 9.5) [2557], L-765314 (p*K*_i_ 7.7) [1993], AH 11110 (p*K*_i_ 7.5) [2277]	BMY-7378 (p*K*_i_ 8.6–9.1) [355, 2096, 2899]

**Comments**: The three α1-adrenoceptor subtypes are α_1A_, α_1B_ and α_1D_. The previously described α_1C_-adrenoceptor is a species homologue that corresponds to the pharmacologically defined α_1A_-adrenoceptor [1012]. Some tissues possess α_1A_-adrenoceptors (termed α_1L_-adrenoceptors [715, 1802]) that display relatively low affinity in functional and binding assays for prazosin indicative of different receptor states or locations. α_1A_-Adrenoceptor C-terminal splice variants form homo- and heterodimers, and do not generate a functional α_1L_-adrenoceptor [2135]. Recombinant α_1D_-adrenoceptors have been shown in some heterologous systems to be mainly located intracellularly but cell-surface localization is encouraged by truncation of the N-terminus, or by co-expression and formation of heterodimers of with α_1B_-α_1B_- or β_2_–β_2_-adrenoceptors [912, 2623]. In blood vessels all three α_1-_-adrenoceptor subtypes are located both at the cell surface and intracellularly [1711, 1712]. Signalling is predominantly *via* G_q/11_ but α_1_-adrenoceptors also couple to G_i/o_, G_s_ and G_12/13_. Several α_1A_-adrenoceptor agonists display ligand directed signalling bias relative to noradrenaline [669] although some bias appears to relate to off-target activity [515] . There are also differences between subtypes in coupling efficiency to different pathways. In vascular smooth muscle, the potency of agonists is related to the predominant subtype, α_1D_- conveying greater agonist sensitivity compared to α_1A_-adrenoceptors [709].

### Adrenoceptors, α_2_

The three α_2_-adrenoceptor subtypes α_2A_, α_2B_ and α_2C_ are activated by (−)-adrenaline and with lower potency by (−)-noradrenaline. Brimonidine and talipexole are agonists and rauwolscine and yohimbine antagonists selective for α_2_- relative to α_1_-adrenoceptors. [^3^H]ra.uwolscine, [^3^H]brimonidine and [^3^H]RX821002 are relatively selective radioligands. There are species variations in the pharmacology of the α_2A_-adrenoceptor. Multiple mutations of α_2_-adrenoceptors have been described, some associated with alterations in function. Presynaptic α_2_-adrenoceptors regulate many functions in the nervous system. The α_2_-adrenoceptor agonists clonidine, guanabenz and brimonidine affect central baroreflex control (hypotension and bradycardia), induce hypnotic effects and analgesia, and modulate seizure activity and platelet aggregation. Clonidine is an anti-hypertensive (relatively little used) and counteracts opioid withdrawal. Dexmedetomidine (also xylazine) is increasingly used as a sedative and analgesic in human [132] and veterinary medicine and has sympatholytic and anxiolytic properties. The α_2_-adrenoceptor antagonist mirtazapine is used as an anti-depressant. The α_2B_ subtype appears to be involved in neurotransmission in the spinal cord and α_2C_ in regulating catecholamine release from adrenal chromaffin cells. Although subtype-selective antagonists have been developed, none are used clinically and they remain experimental tools.

**Table:** 

Nomenclature	α_2A_-adrenoceptor	α_2B_-adrenoceptor	α_2C_-adrenoceptor
HGNC, UniProt	*ADRA2A*, P08913	*ADRA2B*, P18089	*ADRA2C*, P18825
Endogenous agonists	(−)-adrenaline [1154, 2045, 2093], (−)-noradrenaline [1154, 2045, 2093]	(−)-noradrenaline (Partial agonist) [1154, 2045, 2093], (−)-adrenaline [1154, 2093]	(−)-noradrenaline [1154, 1385, 2045, 2093], (−)-adrenaline [1154, 2093]
Agonists	dexmedetomidine (Partial agonist) [1154, 1598, 2013, 2045, 2093], clonidine (Partial agonist) [1154, 2013, 2045, 2093], brimonidine [1154, 1598, 2013, 2045, 2093], apraclonidine [1827], guanabenz [93, 2093], guanfacine (Partial agonist) [1154, 1602, 2093], moxonidine [2093], tizanidine [2093]	dexmedetomidine [1154, 1598, 2013, 2045, 2093], clonidine (Partial agonist) [1154, 2013, 2045, 2093], brimonidine (Partial agonist) [1154, 2013, 2045, 2093], guanabenz [93, 2093], guanfacine [1154, 2093], oxymetazoline (Partial agonist) [1154, 2093, 2628], moxonidine [2093], tizanidine [2093]	dexmedetomidine [1154, 2013, 2045, 2093], brimonidine (Partial agonist) [1154, 1598, 2013, 2045, 2093], apraclonidine [1827, 2093], oxymetazoline (Partial agonist) [1154, 1385, 2093, 2628], guanfacine (Partial agonist) [1154], guanabenz [93, 2093], moxonidine [2093]
Selective agonists	oxymetazoline (Partial agonist) [1154, 1598, 2093, 2628]	–	–
Antagonists	RX821002 (p*K*_i_ 8.1–9.2) [2094, 2628], yohimbine (p*K*_i_ 8.4–9.2) [327, 570, 2094, 2628], atipamezole (p*K*_i_ 8.5) [2094], idazoxan (p*K*_i_ 7.2) [2094]	lisuride (p*K*_i_ 8.5–9.9) [1735, 2094], yohimbine (p*K*_i_ 7.9–8.9) [327, 570, 2094, 2628], phenoxybenzamine (p*K*_i_ 8.5) [2758], RX821002 (p*K*_i_ 7.5–8.4) [2094, 2628], atipamezole (p*K*_i_ 7.9) [2094], idazoxan (p*K*_i_ 6.4) [2094], tolazoline (p*K*_i_ 5.5) [1154]	MK-912 (p*K*_i_ 9.8–10) [2094], lisuride (p*K*_i_ 9.3– 9.9) [1598, 1735, 2094], yohimbine (p*K*_i_ 8.5–9.5) [327, 570, 2094, 2628], WB 4101 (p*K*_i_ 8.2–9.4) [327, 570, 2094, 2628], spiroxatrine (p*K*_i_ 8.7–9) [2094, 2628], RX821002 (p*K*_i_ 8.1–8.7) [2094, 2253, 2628], atipamezole (p*K*_i_ 8.5) [2094], mirtazapine (p*K*_i_ 7.7) [692], idazoxan (p*K*_i_ 7.2) [2094], tolazoline (p*K*_i_ 5.4) [1154]
Selective antagonists	BRL 44408 (p*K*_i_ 8.2–8.8) [2628, 2901]	ARC-239 (p*K*i 6.8–8.6) [327, 570, 2094, 2628], imiloxan (p*K*_i_ 7.3) [1724] – Rat	JP1302 (p*K*_B_ 6.9–7.8) [2094, 2253]
Labelled ligands	–	–	[^3^H]MK-912 (Antagonist) (p*K*_d_ 10.1) [2628]

**Comments**: The three α_2_-adrenoceptor subtypes are termed α_2A_, α_2B_ and α_2C_. ARC-239 and prazosin show some selectivity for α_2B_- and α_2C_-adrenoceptors over α_2A_-adrenoceptors. Oxymetazoline is an imidazoline partial agonist that also binds to non-GPCR binding sites for imidazolines, classified as I_1_, I_2_ and I_3_ [521] at which catecholamines have a low affinity, while rilmenidine and moxonidine are selective ligands with hypotensive effects *in vivo*. I_1_-imidazoline recognition sites cause central inhibition of sympathetic tone, I_2_-imidazoline sites are an allosteric binding site on monoamine oxidase B, and I_3_-imidazoline sites regulate insulin secretion from pancreatic β-cells. α_2A_-adrenoceptor stimulation reduces insulin secretion from β-islets [2867], with a polymorphism in the 5’-UTR of the *ADRA2A* gene being associated with increased receptor expression in β-islets and heightened susceptibility to diabetes [2208]. The α_2A_- and α_2C_-adrenoceptors form homodimers [2407]. Heterodimers between α_2A_- and either the α_2c_-adrenoceptor or μ opioid peptide receptor exhibit altered signalling and trafficking properties compared to the individual receptors [2407, 2537, 2684]. Signalling by α_2_-adrenoceptors is primarily via Gi/o, although the α2A-adrenoceptor also couples to Gs [630]. Imidazoline compounds display bias relative to each other at the α_2A_-adrenoceptor [2003]. The noradrenaline reuptake inhibitor desipramine acts directly on α_2A_-adrenoceptors to promote internalisation *via* recruitment of β-arrestin [493]. The structure of the α_2B_-adrenoceptor has recently been determined by cryo-EM in complex with dexmedetomidine and Gα_o_ at a resolution of 2.9 Å providing insights into the structural requirements required for interactions with α_2_-adrenoceptor agonists [2905].

### Adrenoceptors, β

The three β-adrenoceptor subtypes β_1_, β_2_ and β_3_ are activated by the endogenous agonists (−)-adrenaline and (−)-noradrenaline. Isoprenaline is selective for β-adrenoceptors relative to α_1_- and α_2_-adrenoceptors, while propranolol (p*K*_i_ 8.2–9.2) and cyanopindolol (p*K*_i_ 10.0–11.0) are relatively selective antagonists for β_1_- and β_2_- relative to β_3_-adrenoceptors. (−)-noradrenaline, xamoterol and (−)-Ro 363 show selectivity for β_1_- relative to β_2_-adrenoceptors. Pharmacological differences exist between human and mouse β_3_-adrenoceptors, and the’rodent selective’ agonists BRL 37344 and CL316243 have low efficacy at the human β_3_-adrenoceptor whereas CGP 12177 (low potency) and L 755507 activate human β_3_-adrenoceptors [88]. β_3_-Adrenoceptors are resistant to blockade by propranolol, but can be blocked by high concentrations of bupranolol. SR59230A has reasonably high affinity at β_3_-adrenoceptors, but does not discriminate between the three β- subtypes [1727] whereas L-748337 is more selective. [^125^I]-cyanopindolol, [^125^I]-hydroxy benzylpindolol and [^3^H]-alprenolol are high affinity radioligands that label β_1_- and β_2_- adrenoceptors and β_3_-adrenoceptors can be labelled with higher concentrations (nM) of [^125^I]-cyanopindolol together with β_1_- and β_2_-adrenoceptor antagonists. Fluorescent ligands such as BODIPY-TMR-CGP12177 can be used to track β-adrenoceptors at the cellular level [8]. Somewhat selective β_1_-adrenoceptor agonists (denopamine, dobutamine) are used short term to treat cardiogenic shock but, chronically, reduce survival. β_1_-Adrenoceptor-preferring antagonists are used to treat cardiac arrhythmias (atenolol, bisoprolol, esmolol) and cardiac failure (metoprolol, nebivolol) but also in combination with other treatments to treat hypertension (atenolol, betaxolol, bisoprolol, metoprolol and nebivolol) [2802]. Cardiac failure is also treated with carvedilol that blocks β_1_- and β_2_-adrenoceptors, as well as α_1_-adrenoceptors. Short (salbutamol, terbutaline) and long (formoterol, salmeterol) acting β_2_-adrenoceptor-selective agonists are powerful bronchodilators used to treat respiratory disorders. Many first generation β-adrenoceptor antagonists (propranolol) block both β_1_- and β_2_-adrenoceptors and there are no β_2_-adrenoceptor-selective antagonists used therapeutically. The β_3_-adrenoceptor agonist mirabegron is used to control overactive bladder syndrome. There is evidence to suggest that β-adrenoceptor antagonists can reduce metastasis in certain types of cancer [1016].

**Table:** 

Nomenclature	β_1_-adrenoceptor	β_2_-adrenoceptor
HGNC, UniProt	*ADRB1*, P08588	*ADRB2*, P07550
Potency order of endogenous ligands	(−)-noradrenaline > (−)-adrenaline	(−)-adrenaline > (−)-noradrenaline
Endogenous agonists	(−)-adrenaline [739, 1033], (−)-noradrenaline [739, 1033], noradrenaline [739]	(−)-adrenaline [739, 1033, 1150], (−)-noradrenaline [739, 1033]
Agonists	isoprenaline [739, 2276], dobutamine (Partial agonist) [1118], cimaterol [114], fenoterol [114]	arformoterol [45], indacaterol [147, 150], fenoterol [75, 121, 549], isoprenaline [2276], cimaterol [114]
Selective agonists	(−)-Ro 363 [1764], xamoterol (Partial agonist) [1118], denopamine (Partial agonist) [1118, 2500]	formoterol [114], olodaterol [254], salmeterol [114], zinterol [114], vilanterol [2085], abediterol [68], procaterol [114], clenbuterol [114, 121, 1254], salbutamol (Partial agonist) [115, 1118], terbutaline (Partial agonist) [115], orciprenaline [2435]
Antagonists	carvedilol (p*K*_i_ 8.8–9.5) [115, 342], bupranolol (p*K*_i_ 7.3–9) [115, 342, 1568], (−)-propranolol (p*K*_i_ 7.9–8.9) [115, 1191, 1568, 2422], SR59230A (p*K*_i_ 7.5–8.6) [115, 342], levobunolol (p*K*_i_ 8.4) [93], labetalol (Partial agonist) (p*K*_i_ 7.6–8.2) [93, 115, 118], metoprolol (p*K*_i_ 7–7.9) [115, 118, 342, 1033, 1568], esmolol (p*K*_i_ 6.7–6.9) [93, 1859], nadolol (p*K*_i_ 6.9) [342], practolol (p*K*_i_ 6.1–6.8) [115, 1568], propafenone (p*K*_i_ 6.7) [93], sotalol (p*K*_i_ 6.1) [93]	carvedilol (p*K*_i_ 9.4–9.9) [115, 342], timolol (p*K*_i_ 9.7) [115], propranolol (p*K*_i_ 9.1–9.5) [115, 119, 1118, 1568], SR59230A (p*K*_i_ 8.5–9.3) [115, 342], levobunolol (p*K*_i_ 9.3) [93], bupranolol (p*K*_i_ 8.3–9.1) [115, 342, 1568], alprenolol (Partial agonist) (p*K*_i_ 9) [115], nadolol (p*K*_i_ 7–8.6) [115, 342], labetalol (Partial agonist) (p*K*_i_ 8) [93], propafenone (p*K*_i_ 7.4) [93], sotalol (p*K*_i_ 6.3–6.5) [93, 115]
Selective antagonists	CGP 20712A (p*K*_i_ 7.9–9.2) [115, 342, 1568, 2275, 2422], nebivolol (p*K*_i_ 9.2) [114, 726], levobetaxolol (p*K*_i_ 8.2–9.1) [2354], NDD-825 (p*K*_i_ 8.3–9) [117], ICI-89406 (Partial agonist) (p*K*_i_ 8.8) [1749], betaxolol (p*K*_i_ 8.8) [1568], nebivolol (pIC_50_ 8.1–8.7) [2002] – Rabbit, NDD-713 (p*K*_i_ 7.8–8.5) [117], bisoprolol (p*K*_i_ 8) [117]	ICI 118551 (Inverse agonist) (pKi 9.2–9.5) [115, 119, 1568]
Allosteric modulators	–	AS408 [1554]
Labelled ligands	[^125^I]ICYP (Antagonist) (p*K*_d_ 10.4–11.3) [1118, 1568, 2276]	[^125^I]ICYP (Antagonist) (p*K*_d_ 11.1) [1568, 2276]
Comments	The agonists indicated have less than two orders of magnitude selectivity [114].	–

**Table:** 

Nomenclature	β_3_-adrenoceptor
HGNC, UniProt	*ADRB3*, P13945
Potency order of endogenous ligands	(−)-noradrenaline = (−)-adrenaline
Endogenous agonists	(−)-noradrenaline [1033, 2066, 2471], (−)-adrenaline [1033]
Agonists	carazolol (Partial agonist) [114, 1707], isoprenaline [114, 1033, 1707, 1764, 2066, 2276, 2471], fenoterol [114]
Selective agonists	L 755507 [114], vibegron [302, 578, 634], L742791 [2755], solabegron [1093, 1726, 2626], mirabegron [2523], SB251023 [1090] – Mouse, rodent selective BRL 37344 [214, 594, 1033, 1707], rodent selective CL316243 [2864]
Antagonists	SR59230A (p*K*_i_ 6.9–8.4) [114, 342, 550, 1033], bupranolol (p*K*_i_ 6.8–7.3) [214, 342, 1568, 1707], propranolol (p*K*_i_ 6.3–7.2) [1568, 2066], levobunolol (p*K*_i_ 6.8) [2066]
Selective antagonists	L748328 (p*K*_i_ 8.4–8.6) [114, 342], L-748337 (p*K*_i_ 8–8.4) [114, 342]
Labelled ligands	[^125^I]ICYP (Agonist, Partial agonist) [1568, 1764, 2066, 2276, 2471], [^3^H]CGP12177 (Agonist, Partial agonist) [115]
Comments	Agonist SB251023 has a pEC_50_ of 6.9 for the splice variant of the mouse β_3_ receptor, β_3b_ [1090]. [^3^H] L-748337 is a selective antagonist that is used to label β_3_-AR [2661].

**Comments**: The three β-adrenoceptors are termed β_1_, β_2_ and β_3_. [^125^I]ICYP can be used to define either β_1_- or β_2_-adrenoceptors when conducted in the presence of a β_1_- or a β_2_-adrenoceptor-selective antagonist. A fluorescent analogue of CGP 12177 is used to study β-adrenoceptors in living cells [120]. [^125^I]ICYP at higher (nM) concentrations has been used to label β_3_-adrenoceptors in systems with few if any other β-adrenoceptor subtypes. The β_3_-adrenoceptor has an intron in the coding region, but splice variants have only been described for the mouse [670], where the isoforms display different signalling characteristics [1090]. There are three β-adrenoceptors in turkey (termed the tβ, tβ3c and tβ4c) with pharmacology that differs from the human β-adrenoceptors [116]. Numerous polymorphisms have been described for the β-adrenoceptors; some are associated with altered signalling and trafficking, susceptibility to disease and/or altered responses to pharmacotherapy [1515]. All β-adrenoceptors couple to G_s_ (activating adenylyl cyclase and elevating cAMP levels), but the β_2_- and β_3_-adrenoceptors in particular can also activate G_i_ and the β_2_-adrenoceptor activates β-arrestin-mediated signalling. Many β_1_- and β_2_-adrenoceptor antagonists are agonists at β_3_-adrenoceptors (CL316243, CGP 12177 and carazolol). Many ‘antagonists’ of cAMP accumulation, for example carvedilol and bucindolol, weakly activate MAP kinase pathways [118, 671, 753, 754, 2273, 2274] and thus display biased agonism. Bupranolol acts as a neutral antagonist in most systems so far examined. Agonists also display biased signalling at the β_2_-adrenoceptor *via* G_s_ or arrestins [611]. X-ray crystal structures have been described of the agonist bound [2743] and antagonist bound forms of the β_1_- [2744], agonist-bound [424] and antagonist-bound forms of the β_2_-adrenoceptor [2142, 2207], as well as a fully active agonist-bound, G_s_ protein-coupled β_2_-adrenoceptor [2143], as well as providing insights into the structural requirements for agonist, partial agonist, antagonist, G protein and β-arrestin coupling [2759]. Structures have also been described for negative allosteric modulators of the β_2_-adrenoceptor [1554]. Cryo-EM studies have also been recently described that provide a structural framework for agonist mediated signal transduction [2474]. The agonists carvedilol and bucindolol bind to a site on the β_1_-adrenoceptor involving contacts in TM2, 3, and 7 and extracellular loop 2 that may facilitate coupling to arrestins [2744]. Compounds displaying β-arrestin-biased signalling at the β_2_-adrenoceptor have a greater effect on the conformation of TM7, whereas full agonists for G_s_ coupling promote movement of TM5 and TM6 [1547]. Recent studies using NMR spectroscopy demonstrate significant conformational flexibility in the β_2_-adrenoceptor that is stabilized by both agonist and G proteins highlighting the dynamic nature of interactions with both ligand and downstream signalling partners [1277, 1635, 1919]. Such flexibility likely has consequences for our understanding of allosterism and biased agonism, and for the future therapeutic exploitation of these phenomena.

Further reading on AdrenoceptorsBakerJG
 (2011) Evolution of β-blockers: from anti-anginal drugs to ligand-directed signalling. Trends Pharmacol Sci
32: 227–3421429598
10.1016/j.tips.2011.02.010PMC3081074BylundDB
 (1994) International Union of Pharmacology nomenclature of adrenoceptors. Pharmacol Rev
46: 121–367938162
EvansBA
 (2010) Ligand-directed signalling at beta-adrenoceptors. Br J Pharmacol
159: 1022–3820132209
10.1111/j.1476-5381.2009.00602.xPMC2839261JensenBC
 (2011) Alpha-1-adrenergic receptors: targets for agonist drugs to treat heart failure. J Mol Cell Cardiol
51: 518–2821118696
10.1016/j.yjmcc.2010.11.014PMC3085055KobilkaBK. (2011) Structural insights into adrenergic receptor function and pharmacology. Trends Pharmacol Sci
32: 213–821414670
10.1016/j.tips.2011.02.005PMC3090711LangerSZ. (2015) α2-Adrenoceptors in the treatment of major neuropsychiatric disorders. Trends Pharmacol Sci
36: 196–20225771972
10.1016/j.tips.2015.02.006MichelMC
 (2015) Selectivity of pharmacological tools: implications for use in cell physiology. A review in the theme: Cell signaling: proteins, pathways and mechanisms. Am J Physiol, Cell Physiol
308: C505–2025631871
10.1152/ajpcell.00389.2014

## Angiotensin receptors

G protein-coupled receptors → Angiotensin receptors

**Overview**: The actions of angiotensin II (*AGT*, P01019) (Ang II) are mediated by AT_1_ and AT_2_ receptors (**nomenclature as agreed by the NC-IUPHAR Subcommittee on Angiotensin receptors** [544, 1219]), which have around 30% sequence similarity. The decapeptide angiotensin I (*AGT*, P01019), the octapeptide angiotensin II (*AGT*, P01019) and the heptapeptide angiotensin III (*AGT*, P01019) are endogenous ligands. Losartan, candesartan, olmesartan, telmisartan, *etc*. are clinically used AT_1_ receptor blockers.

**Table:** 

Nomenclature	AT_1_ receptor	AT_2_ receptor
HGNC, UniProt	*AGTR1*,P30556	*AGTR2*,P50052
Endogenous agonists	angiotensin II (*AGT*,P01019) [545, 2664], angiotensin III (*AGT*, P01019) [545], angiotensin IV (*AGT*, P01019) (Partial agonist) [1435]	angiotensin III (*AGT*, P01019) [504, 545, 2778], angiotensin II (*AGT*, P01019) [545, 2418, 2778], angiotensin-(1-7) (*AGT*, P01019) [249]
Agonists	[Sar^1^, Cha^4^]Ang-II [1041, 1753] – Rat	–
Selective agonists	L-162,313 [2024], L-163,101 [2635]	CGP42112 [249], [p-aminoPhe6]ang II [545, 2443] – Rat, compound 21 [2681]
Antagonists	saprisartan (p*K*_i_ 9.1) [1013] – Rat, 5-oxo-1-2-4-oxadiazol biphenyl (pIC_50_ 8.8) [1893] – Rat, 5-butyl-methyl immidazole carboxylate 30 (pIC_50_ 8.5) [19], LY303336 (pIC_50_ 8.3) [2665], TRV027 (pKd 7.7) [2688]	saralasin (pIC_50_ 9) [434] – Rat
Selective antagonists	candesartan (pIC_50_ 9.5–9.7) [2664], eprosartan (pIC_50_ 8.4–8.8) [636], losartan (pIC_50_ 7.4–8.7) [545, 2585], telmisartan (pIC_50_ 8.4) [1691], olmesartan (pIC_50_ 8.1) [1326]	PD123177 (pIC_50_ 8.5–9.5) [386, 434, 622] – Rat, olodanrigan (pIC_50_ 8.5–9.3) [699, 2175, 2416], PD123319 (p*K*_d_ 8.7–9.2) [545, 621, 2787]
Labelled ligands	[^3^H]candesartan (Antagonist) (p*K*_d_ 10.3) [694], [^125^I][Sar1]Ang-II (Agonist) [690] – Rat, [^125^I][Sar1, Ile8]Ang-II (Agonist, Partial agonist) [690] – Rat, [^3^H]eprosartan (Antagonist) (p*K*_d_ 9.1) [28] – Rat, [^3^H]losartan (Antagonist) (p*K*_d_ 8.2) [390] – Rat	[^125^I]CGP42112 (Agonist) [545, 2778, 2779], [^125^I][Sar^1^, Ile^8^]Ang-II (Agonist) [2535] – Rat
Comments	Telmisartan and candesartan are also reported to be agonists of PPARγ [2465].	Compounds have been generated with enhanced AT_2_ receptor selectivity and proteolytic stability by imposing conformational constraints at position 6 of Angiotensin II [2605].

**Comments**: AT_1_ receptors are predominantly coupled to G_q/11_ [544, 1219], however they also recruit β-arrestins and stimulate G protein-independent β-arrestin signaling [1151, 1585, 2912]. Most species express a single *AGTR1* gene located on chromosome 3, but two related *Agtr1a* and *Agtr1b* receptor genes are expressed in rodents. Expression of the X chromosome-linked *AGTR2* gene is higher in females than males. AT_1_ receptor antagonists bearing substituted biphenyl tetrazolium moieties are clinically used to treat hypertension and other cardiovascular disorders. They bind to AT_1_ receptors with nanomolar affinity and are more potent than losartan in functional studies [1219]. High-resolution crystal structures of AT_1_ receptor bound to nonpeptide antagonists (PDB id: 4ZUD, 4YAY) and peptide agonists (PDB id: 6DO1, 6OS0, 6OS1, 6OS2) are deposited in the protein structure database [2393]. The AT_1_ and bradykinin B2 receptors have been proposed to form a heterodimeric complex [5]. β-arrestin1 prevents AT_1_-B2 receptor heteromerization [2117]. The AT_2_ receptor counteracts several of the growth responses initiated by AT_1_ receptors. The AT_2_ receptor is much less abundant than the AT_1_ receptor in adult tissues and is upregulated in pathological conditions. Agonist activation of AT_2_ receptors promotes anti-fibrotic tissue protection in cardiovascular and renal diseases [2738]. AT_2_ receptors are involved in pain modulation [57, 2175] and AT_2_ receptor antagonists relieve peripheral neuropathic pain in chronic diseases such as diabetes [2175, 2415]. High-resolution structures of the AT_2_ receptor bound to non-peptide antagonists (PDB id: 5UNF, 7JNI) and peptide agonists (PDB id: 5XJM, 6JOD) are available in the protein structure database [2393]. An AT_3_ receptor was proposed based on cDNA isolated from a neuroblastoma cell line, but existence of a genuine *AGTR3* gene and AT_3_ receptor are not confirmed at this time. However, there is evidence for an AT_4_ receptor that specifically binds angiotensin IV (*AGT*, P01019) (*AGT*; P01019) and is located in the brain and kidney. An additional putative endogenous ligand for the AT_4_ receptor has been described (LVV-hemorphin (*HBB*, P68871) [*HBB*, P68871], a globin decapeptide) [1759].

Further reading on Angiotensin receptorsAsadaH
 (2020) The Crystal Structure of Angiotensin II Type 2 Receptor with Endogenous Peptide Hormone. Structure
28: 418–425.e431899086
10.1016/j.str.2019.12.003BaloghM
 (2021) Angiotensin receptors and neuropathic pain. Pain Rep
6: e86933981922
10.1097/PR9.0000000000000869PMC8108581KarnikSS
 (2015) International Union of Basic and Clinical Pharmacology. XCIX. Angiotensin Receptors: Interpreters of Pathophysiological Angiotensinergic Stimuli [corrected]. Pharmacol Rev
67: 754–81926315714
10.1124/pr.114.010454PMC4630565SinghKD
 (2021) Novel allosteric ligands of the angiotensin receptor AT1R as autoantibody blockers. Proc Natl Acad Sci U S A
118:10.1073/pnas.2019126118PMC837997834380734SinghKD
 (2022) Structural Perspective on the Mechanism of Signal Activation, Ligand Selectivity and Allosteric Modulation in Angiotensin Receptors: IUPHAR Review 34. Br J Pharmacol10.1111/bph.15840PMC939892535318654WinglerLM
 (2020) Angiotensin and biased analogs induce structurally distinct active conformations within a GPCR. Science
367: 888–89232079768
10.1126/science.aay9813PMC7171558

## Apelin receptor

G protein-coupled receptors → Apelin receptor

**Overview**: The apelin receptor (**nomenclature as agreed by the NC-IUPHAR Subcommittee on the apelin receptor** [2056] **and subsequently updated** [2154]) responds to apelin, a 36 amino-acid peptide derived initially from bovine stomach. Apelin-36 (*APLN*, Q9ULZ1), apelin-13 (*APLN*, Q9ULZ1) and [Pyr^1^]apelin-13 (*APLN*, Q9ULZ1) are the predominant endogenous ligands which are cleaved from a 77 amino-acid precursor peptide (*APLN*, Q9ULZ1) [2552]. A second family of peptides discovered independently and named Elabela [435] or Toddler, that has little sequence similarity to apelin, is present, and functional at the apelin receptor in the adult cardiovascular system [2001, 2872]. The enzymatic pathways generating biologically active apelin and Elabela isoforms have not been determined but both propeptides include sites for potential proprotein convertase processing [2370]. Structure-activity relationship Elabela analogues have been described [1836, 2608]. The stoichiometry of apelin receptor-heterotrimeric G protein complexes has been studied using cryogenic-electron microscopy [2907].

**Table:** 

Nomenclature	apelin receptor
HGNC, UniProt	*APLNR*, P35414
Potency order of endogenous ligands	[Pyr^1^]apelin-13 (*APLN*, Q9ULZ1) ≥ apelin-13 (*APLN*, Q9ULZ1) > apelin-36 (*APLN*, Q9ULZ1) [678, 2552]
Endogenous agonists	apelin-13 (*APLN*, Q9ULZ1) [678, 1057, 1704], apelin receptor early endogenous ligand (*APELA*, P0DMC3) [564], apelin-17 (*APLN*, Q9ULZ1) [641, 1704], [Pyr^1^] apelin-13 (*APLN*, Q9ULZ1) [1231, 1704], Elabela/Toddler-21 (*APELA*, P0DMC3) [2871], Elabela/Toddler-32 (*APELA*, P0DMC3) [2871], apelin-36 (*APLN*, Q9ULZ1) [678, 1057, 1231, 1704], Elabela/Toddler-11 (*APELA*, P0DMC3) [2871]
Selective agonists	CMF-019 (Biased agonist) [2153], MM07 (Biased agonist) [267], azelaprag [87, 405]
Antagonists	MM54 (pK_i_ 8.2) [1597]
Labelled ligands	[^125^I][Nle^75^, Tyr^77^]apelin-36 (human) (Agonist) [1231], [^125^I][Glp^65^Nle^75^, Tyr^77^]apelin-13 (Agonist) [1057], [^125^I](Pyr^1^)apelin-13 (Agonist) [1225], [^125^I]apelin-13 (Agonist) [678], [^3^H](Pyr^1^)[Met(0)11]-apelin-13 (Agonist) [1704]

**Comments**: Potency order determined for heterologously expressed human apelin receptor (p*D*_2_ values range from 9.5 to 8.6). The apelin receptor may also act as a co-receptor with CD4 for isolates of human immunodeficiency virus, with apelin blocking this function [369]. A modified apelin-13 peptide, apelin-13(F13A) was reported to block the hypotensive response to apelin in rat *in vivo* [1453], however, this peptide exhibits agonist activity in HEK293 cells stably expressing the recombinant apelin receptor [678]. The apelin receptor antagonist, MM54, was reported to suppress tumour growth and increase survival in an intracranial xenograft mouse model of glioblastoma [946]. 

Further reading on Apelin receptorChapmanFA
 (2021) The therapeutic potential of apelin in kidney disease. Nat Rev Nephrol
17: 840–85334389827
10.1038/s41581-021-00461-zPMC8361827ChenJ
 (2022) Roles of apelin/APJ system in cancer: Biomarker, predictor, and emerging therapeutic target. J Cell Physiol
237: 3734–375135933701
10.1002/jcp.30845MarsaultE
 (2019) The apelinergic system: a perspective on challenges and opportunities in cardiovascular and metabolic disorders. Ann N Y Acad Sci
1455: 12–3331236974
10.1111/nyas.14123PMC6834863PitkinSL
 (2010) International Union of Basic and Clinical Pharmacology. LXXIV. Apelin receptor nomenclature, distribution, pharmacology, and function. Pharmacol Rev
62: 331–4220605969
10.1124/pr.110.002949ReadC
 (2019) International Union of Basic and Clinical Pharmacology. CVII. Structure and Pharmacology of the Apelin Receptor with a Recommendation that Elabela/Toddler Is a Second Endogenous Peptide Ligand. Pharmacol Rev
71: 467–50231492821
10.1124/pr.119.017533PMC6731456YangP
 (2015) Apelin, Elabela/Toddler, and biased agonists as novel therapeutic agents in the cardiovascular system. Trends Pharmacol Sci
36: 560–726143239
10.1016/j.tips.2015.06.002PMC4577653ZhangY
 (2023) Neuroprotective Roles of Apelin-13 in Neurological Diseases. Neurochem Res10.1007/s11064-023-03869-036745269

## Bile acid receptor

G protein-coupled receptors → Bile acid receptor

**Overview**: The bile acid receptor (GPBA) responds to bile acids produced during the liver metabolism of cholesterol. Selective agonists are promising drugs for the treatment of metabolic disorders, such as type II diabetes, obesity and atherosclerosis.

**Table:** 

Nomenclature	GPBA receptor
HGNC, UniProt	*GPBAR1*, Q8TDU6
Potency order of endogenous ligands	lithocholic acid > deoxycholic acid > chenodeoxycholic acid,cholic acid [1230, 1664]
Selective agonists	S-EMCA [2011] – Mouse, betulinic acid [793], oleanolic acid [2271]

**Comments**: The triterpenoid natural product betulinic acid has also been reported to inhibit inflammatory signalling through the NFκB pathway [2516]. Disruption of GPBA expression is reported to protect from cholesterol gallstone formation [2675]. A new series of 5-phenoxy-1,3-dimethyl-1H-pyrazole-4-carboxamides have been reported as highly potent agonists [1561].

Further reading on Bile acid receptorDubocH
 (2014) The bile acid TGR5 membrane receptor: from basic research to clinical application. Dig Liver Dis
46: 302–1224411485
10.1016/j.dld.2013.10.021PMC5953190LefebvreP
 (2009) Role of bile acids and bile acid receptors in metabolic regulation. Physiol Rev
89: 147–9119126757
10.1152/physrev.00010.2008LieuT
 (2014) GPBA: a GPCR for bile acids and an emerging therapeutic target for disorders of digestion and sensation. Br J Pharmacol
171: 1156–6624111923
10.1111/bph.12426PMC3952795van NieropFS
 (2017) Clinical relevance of the bile acid receptor TGR5 in metabolism. Lancet Diabetes Endocrinol
5: 224–23327639537
10.1016/S2213-8587(16)30155-3

## Bombesin receptors

G protein-coupled receptors → Bombesin receptors

**Overview**: Mammalian bombesin (Bn) receptors comprise 3 subtypes: BB_1_, BB_2_, BB_3_ (**nomenclature recommended by the NC-IUPHAR Subcommittee on bombesin receptors,** [41, 1160]). BB_1_ and BB_2_ are activated by the endogenous ligands neuromedin B (*NMB*, P08949) (NMB), gastrin-releasing peptide (*GRP*, P07492) (GRP), and GRP-(18-27) (*GRP*, P07492). Bombesin is a tetra-decapeptide, originally derived from amphibians and structurally closely related to GRP. The three Bn receptor subtypes couple primarily to the G_q/11_ and G_12/13_ family of G proteins [1160]. Each of these receptors is widely distributed in the CNS and peripheral tissues [842, 1159, 1160, 1595, 1596, 2067, 2133, 2265, 2926]. Activation of BB_1_ and BB_2_ receptors causes a wide range of physiological/pathophysiogical actions, including the stimulation of normal and neoplastic tissue growth, smooth-muscle contraction, respiration, gastrointestinal motility, feeding behavior, secretion and many central nervous system effects including regulation of circadian rhythm, body temperature control, sighing, behavioral disorders and mediation of pruritus [411, 417, 752, 1160, 1497, 1784, 1793, 1795, 2111, 2133, 2490, 2715]. BB_3_ is an orphan receptor, although some propose it is constitutively active [2541]. BB_3_ receptor knockout studies show it has important roles in glucose and insulin regulation, metabolic homeostasis, feeding, regulation of body temperature, obesity, diabetes mellitus and growth of normal/neoplastic tissues [842, 1496, 1622, 1791, 1934, 2829]. Bn receptors are one of the most frequently overexpressed receptors in cancers and are receiving increased attention for their roles in tumor growth, as well as for tumour imaging and for receptor-targeted cytotoxicity [128, 1387, 1619, 1640, 1641, 1782, 1793, 2257]. Bn receptors are also receiving attention because they are one of the primary neurotransmitters for pruritus [411, 417, 1263, 2490].

**Table:** 

Nomenclature	BB_1_ receptor	BB_2_ receptor	BB_3_ receptor
HGNC, UniProt	*NMBR*, P28336	*GRPR*, P30550	*BRS3*, P32247
Endogenous agonists	neuromedin B (*NMB*, P08949) [1160, 2133, 2625]	neuromedin C [2625], gastrin releasing peptide(14-27) (human) [2625], gastrin-releasing peptide (*GRP*, P07492) [178, 2237, 2625]	–
Selective agonists	–	[D-Tyr^6^,β-Ala^11^, N-Me-Ala^13^, Nle^14^]bombesin-(6-14) [1050]	compound 9g [1672, 2131, 2134], MK-7725 [438], MK-5046 [1792, 2327], [D-Tyr^6^, A-pa-4Cl^11^, Phe^13^, Nle^14^]bombesin-(6-14) [1643], compound 17c [1671], bag-1 [888], compound 22e [977]
Antagonists	D-Nal-Cys-Tyr-D-Trp-Lys-Val-Cys-Nal-NH_2_ (pIC_50_ 6.2–6.6) [841]	–	–
Selective antagonists	PD 176252 (pIC_50_ 9.3–9.8) [841], PD 168368 (pIC_50_ 9.3–9.6) [841], dNal-cyc(Cys-Tyr-dTrp-Orn-Val)-Nal-NH_2_	[D-Phe^6^, Leu^13^, Cpa^14^,ψ13-14]bombesin-(6-14) (p*K*_i_ 9.8) [841], JMV641 (pIC_50_ 9.3) [2590] – Mouse, [(3-Ph-Pr^6^), His^7^, D-Ala^11^, D-Pro^13^, y13-14), Phe^14^]bombesin-(6-14) (pIC_50_ 9.2) [841, 1445], JMV594 (pIC_50_ 8.9) [1556, 2590] – Mouse, [D-Tpi^6^, Leu^13^ y(CH_2_NH)-Leu^14^]bombesin-(6-14) (pIC_50_ 8.9) [841], [D-Phe^6^, Stat^13^, Leu^14^Bn(6-14) (pIC_50_ 8.1) [1641]	bantag-1 (pIC_50_ 8.6–8.7) [888, 1792, 2132], ML-18 (pIC_50_ 5.3) [1783]
Labelled ligands	[^125^I]BH-NMB (human, mouse, rat) (Agonist), [^125^I] [Tyr^4^]bombesin (Agonist)	[^125^I][D-Tyr^6^]bombesin-(6-13)-methyl ester (Selective Antagonist) (p*K*_d_ 9.3) [1642] – Mouse, BAY86-7548 (Antagonist) (pIC_50_ 8.6) [1199, 2625], [^125^I][Tyr^4^]bombesin (Agonist) [178], BAY86-7548 (Selective Antagonist) (pIC_50_ 8.1) [1387, 1640, 1641], [^125^I]GRP (human) (Agonist)	[^125^I]bantag-1 (Selective Antagonist) (p*K*_i_ 9.6) [2132], [^3^H]bag-2 (Agonist) [888] – Mouse, [^125^I][D-Tyr^6^, b-Ala^11^, Phe^13^, Nle^14^]bombesin-(6-14) (Agonist) [1644, 1792]

**Comments**: All three human subtypes may be activated by [D-Phe^6^,β-Ala^11^, Phe^13^, Nle^14^]bombesin-(6-14) [1644]. The Agonist [D-Tyr^6^, Apa-4Cl11, Phe^13^, Nle1^4^]bombesin-(6–14) has more than 200-fold selectivity for BB_3_ receptors over BB_1_ and BB_2_ [1643, 1644, 2133, 2133, 2134]. A recent study [2131] shows MK-5046 is functioning as an allosteric agonist for hBRS-3 (the first for any BnR). A further recent study reports for the first time, the inactive crystal structure of hGRPR (BB_2_) as well as two active state GRPR structures bound to GRP or [D-Phe6,β-Ala11, Phe13, Nle14] bombesin-(6–14) [2016].

Further reading on Bombesin receptorsChenZF. (2021) A neuropeptide code for itch. Nat Rev Neurosci
22: 758–77634663954
10.1038/s41583-021-00526-9PMC9437842JensenRT
 (2008) International Union of Pharmacology. LXVIII. Mammalian bombesin receptors: nomenclature, distribution, pharmacology, signaling, and functions in normal and disease states. Pharmacol Rev
60: 1–4218055507
10.1124/pr.107.07108PMC2517428KurthJ
 (2022) GRPr Theranostics: Current Status of Imaging and Therapy using GRPr Targeting Radiopharmaceuticals. Nuklearmedizin
61: 247–26135668669
10.1055/a-1759-4189LiM
 (2019) Bombesin Receptor Subtype-3 in Human Diseases. Arch Med Res
50: 463–46731911345
10.1016/j.arcmed.2019.11.004MansiR
 (2021) Radiolabeled Bombesin Analogs. Cancers (Basel)
13:10.3390/cancers13225766PMC861622034830920MoodyTW
 (2021) Bombesin Receptor Family Activation and CNS/Neural Tumors: Review of Evidence Supporting Possible Role for Novel Targeted Therapy. Front Endocrinol (Lausanne)
12: 72808834539578
10.3389/fendo.2021.728088PMC8441013QinX
 (2021) Recent advances in the biology of bombesin-like peptides and their receptors. Curr Opin Endocrinol Diabetes Obes
28: 232–23733394718
10.1097/MED.0000000000000606Ramos-ÁlvarezI
 (2015) Insights into bombesin receptors and ligands: Highlighting recent advances. Peptides
72: 128–4425976083
10.1016/j.peptides.2015.04.026PMC4641779

## Bradykinin receptors

G protein-coupled receptors → Bradykinin receptors

**Overview**: Bradykinin (or kinin) receptors (**nomenclature as agreed by the NC-IUPHAR subcommittee on Bradykinin (kinin) Receptors** [1460]) are activated by the endogenous peptides bradykinin (*KNG1*, P01042) (BK), [des-Arg^9^] bradykinin (*KNG1*, P01042), Lys-BK (kallidin (*KNG1*, P01042)), [des-Arg^10^]kallidin (*KNG1*, P01042), [Phospho-Ser^6^]-Bradykinin, T-kinin (*KNG1*, P01042) (Ile-Ser-BK), [Hyp^3^]bradykinin (*KNG1*, P01042) and Lys-[Hyp^3^]-bradykinin (*KNG1*, P01042). Variation in pharmacology and activity of B_1_ and B_2_ receptor antagonists at species orthologs has been documented. Icatibant (Hoe140, Firazir) is approved in North America and Europe for the treatment of acute attacks of hereditary angioedema. Inhibition of bradykinin with icatibant in COVID-19 infection is under clinical evaluation, with trial NCT05407597 expected to complete in mid 2023.

**Table:** 

Nomenclature	B_1_ receptor	B_2_ receptor
HGNC, UniProt	*BDKRB1*, P46663	BDKRB2, P30411
Potency order of endogenous ligands	[des-Arg^10^]kallidin (*KNG1*, P01042) > [des-Arg^9^]bradykinin (*KNG1*, P01042) = kallidin (*KNG1*, P01042) > bradykinin (*KNG1*, P01042)	kallidin (*KNG1*, P01042) > bradykinin (*KNG1*, P01042) ≫ [des-Arg^9^]bradykinin (*KNG1*, P01042), [des-Arg^10^]kallidin (*KNG1*, P01042)
Endogenous agonists	[des-Arg^10^]kallidin (*KNG1*, P01042) [95, 144, 832, 1182]	bradykinin (*KNG1*, P01042) [70, 1003]
Selective agonists	NG29 [2281], [Sar, D-Phe^8^, des-Arg^9^]bradykinin [90, 1182]	NG291 [168], labradimil [2282], [Hyp^3^, Tyr(Me)^8^]BK, [Phe^8^,ψ(CH_2_-NH)Arg^9^] BK
Selective antagonists	B-9958 (pK_i_ 9.2–10.3) [802, 2157], deucrictibant (p*K*_i_ 9.3) [1480, 1481], [Leu^9^, des-Arg^10^]kallidin (p*K*_i_ 9.1–9.3) [95, 144], SSR240612 (p*K*_i_ 9.1–9.2) [857], R-954 (pA_2_ 8.6) [833], R-715 (pA_2_ 8.5) [831]	icatibant (p*K*_i_ 10.2) [49], deucrictibant (p*K*_i_ 9.3) [1480, 1481], FR173657 (pA_2_ 8.2) [2191], anatibant (p*K*_i_ 8.2) [2097]
Labelled ligands	[^125^I]Hpp-desArg^10^HOE140 (Antagonist) (p*K*_d_ 10), [^125^I]Hpp-desArg10HOE140 (Antagonist) (p*K*_d_ 10) [551, 1956], [^3^H]Lys-[des-Arg^9^]BK (Agonist), [^3^H]Lys-[Leu^8^][des-Arg^9^]BK (Antagonist)	[^3^H]BK (human, mouse, rat) (Agonist) [2797] – Mouse, [^3^H]NPC17731 (Antagonist) (p*K*_d_ 9.1–9.4) [2933, 2934], [125I]HPP-HOE140 (Antagonist) [551, 1956], [125I][Tyr8]bradykinin (Agonist) [1564]

Further reading on Bradykinin receptorsBlaesN
 (2013) Targeting the’Janus face’ of the B2-bradykinin receptor. Expert Opin Ther Targets
17: 1145–6623957374
10.1517/14728222.2013.827664BruscoI
 (2023) Kinins and their B_1_ and B_2_ receptors as potential therapeutic targets for pain relief. Life Sci
314: 12130236535404
10.1016/j.lfs.2022.121302CoutureR
 (2014) Kinin receptors in vascular biology and pathology. Curr Vasc Pharmacol
12: 223–4824568157
10.2174/1570161112666140226121627MarceauF
 (2020) Bradykinin receptors: Agonists, antagonists, expression, signaling, and adaptation to sustained stimulation. Int Immunopharmacol
82: 10630532106060
10.1016/j.intimp.2020.106305PaquetJL
 (1999) Pharmacological characterization of the bradykinin B2 receptor: interspecies variability and dissociation between binding and functional responses. Br J Pharmacol
126: 1083–9010204994
10.1038/sj.bjp.0702403PMC1565879ThorntonE
 (2010) Kinin receptor antagonists as potential neuroprotective agents in central nervous system injury. Molecules
15: 6598–61820877247
10.3390/molecules15096598PMC6257767

## Calcitonin receptors

G protein-coupled receptors → Calcitonin receptors

**Overview**: This receptor family comprises a group of receptors for the calcitonin/CGRP family of peptides. The calcitonin (CT), amylin (AMY), calcitonin gene-related peptide (CGRP) and adrenomedullin (AM) receptors (**nomenclature as agreed by the NC-IUPHAR Subcommittee on CGRP, AM, AMY, and CT receptors** [968, 970, 2079]) are generated by the genes *CALCR* (which codes for the CT receptor, CTR) and *CALCRL* (which codes for the calcitonin receptor-like receptor, CLR, previously known as CRLR). Their function and pharmacology are altered in the presence of RAMPs (receptor activity-modifying proteins), which are single TM domain proteins of *ca*. 150 amino acids, identified as a family of three members; RAMP1, RAMP2 and RAMP3. There are splice variants of the CTR; these in turn produce variants of AMY receptors [2079], some of which can be potently activated by CGRP. The endogenous agonists are the peptides calcitonin (*CALCA*, P01258), α-CGRP (*CALCA*, P06881) (formerly known as CGRP-I), β-CGRP (*CALCB*, P10092) (formerly known as CGRP-II), amylin (*IAPP*, P10997) (occasionally called islet-amyloid polypeptide, diabetes-associated polypeptide), adrenomedullin (*ADM*, P35318) and adrenomedullin 2/intermedin (*ADM2*, Q7Z4H4). There are species differences in peptide sequences, particularly for the CTs. CTR-stimulating peptide {Pig} (CRSP) is another member of the family with selectivity for the CTR but it is not expressed in humans [1222]. CLR (calcitonin receptor-like receptor) by itself binds no known endogenous ligand, but in the presence of RAMPs it gives receptors for CGRP, adrenomedullin and adrenomedullin 2/intermedin. There are several approved drugs that target this receptor family, such as pramlintide, erenumab, and the “gepant” class of CGRP receptor antagonists. There are also species differences in agonist pharmacology; for example, CGRP displays potent activity at multiple rat and mouse receptors [113, 774]. The summary table only reflects human receptor pharmacology.

**Table:** Complexes

Nomenclature	AMY_1_ receptor	AMY_2_ receptor	AMY_3_ receptor	CGRP receptor	AM_1_ receptor	AM_2_ receptor
Subunits	CT receptor, RAMP1 (Accessory protein)	CT receptor, RAMP2 (Accessory protein)	CT receptor, RAMP3 (Accessory protein)	calcitonin receptor-like receptor, RAMP1 (Accessory protein)	calcitonin receptor-like receptor, RAMP2 (Accessory protein)	calcitonin receptor-like receptor,RAMP3 (Accessory protein)
Potency order of endogenous ligands	amylin (*IAPP*,P10997) ≥ α-CGRP (*CALCA*, P06881), β-CGRP (*CALCB*, P10092) > adrenomedullin 2/intermedin (*ADM2*, Q7Z4H4) ≥ calcitonin (*CALCA*, P01258) > adrenomedullin (*ADM*, P35318)	Poorly defined	amylin (*IAPP*,P10997) > α-CGRP (*CALCA*, P06881), β-CGRP (*CALCB*, P10092) ≥ adrenomedullin 2/intermedin (*ADM2*, Q7Z4H4) ≥ calcitonin (*CALCA*, P01258) > adrenomedullin (*ADM*, P35318)	α-CGRP (*CALCA*, P06881), β-CGRP (*CALCB*, P10092) > adrenomedullin (*ADM*,P35318) ≥ adrenomedullin 2/intermedin (*ADM2*, Q7Z4H4) > amylin (*IAPP*, P10997)	adrenomedullin (*ADM*,P35318) > adrenomedullin 2/ intermedin (*ADM2*, Q7Z4H4) > α-CGRP (*CALCA*,P06881), β-CGRP (*CALCB*, P10092), amylin (*IAPP*,P10997)	adrenomedullin (*ADM*, P35318) ≥ adrenomedullin 2/intermedin (*ADM2*, Q7Z4H4) ≥ α-CGRP (*CALCA*, P06881), β-CGRP (*CALCB*, P10092) > amylin (*IAPP*, P10997)
Endogenous agonists	α-CGRP (*CALCA*,P06881) [966 ,1390, 1391, 1487, 2709], amylin (*IAPP*,P10997) [823], β-CGRP (*CALCB*, P10092)	amylin (*IAPP*, P10997) [823]	amylin (*IAPP*,P10997) [823]	β-CGRP (*CALCB*,P10092) [27, 1701], α-CGRP (*CALCA*, P06881) [27, 1701]	adrenomedullin (*ADM*,P35318) [27, 1701], adrenomedullin 2/intermedin (*ADM2*, Q7Z4H4) [968]	adrenomedullin 2/intermedin (*ADM2*,Q7Z4H4) [968], adrenomedullin (*ADM*, P35318) [27, 725]
Agonists	pramlintide [823], calcitonin (salmon)	–	pramlintide [823], calcitonin (salmon)	–	–	–
Antagonists	rimegepant (p*K*_B_ 8.1) [1976], AC187 (p*K*_B_ 8) [966], CT-(8-32) (salmon) (p*K*_i_ 7.8) [966], olcegepant (p*K*_B_ 7.5) [967]	–	CT-(8-32) (salmon) (p*K*_B_ 7.9) [966], AC187 (p*K*_B_ 7.7) [966]	olcegepant (p*K*_i_ 10.7–11) [602, 967, 969, 1192, 1630], ubrogepant (p*K*_B_ 10.8) [1785], rimegepant (p*K*_B_ 9.6) [1976], telcagepant (pKi 9.1) [2254]	AM-(22-52) (human) (p*K*_i_ 7–7.8) [969]	AM-(22-52) (human)
Labelled ligands	[^125^I]αCGRP (human) (Agonist), [^125^I]BH-AMY (rat, mouse) (Agonist)	[^125^I]BH-AMY (rat, mouse) (Agonist)	[^125^I]BH-AMY (rat, mouse) (Agonist)	[^125^I]αCGRP (human) (Agonist), [^125^I]αCGRP (mouse, rat) (Agonist)	[^125^I]AM (rat) (Agonist)	[^125^I]AM (rat) (Agonist)

**Table:** Receptors and Subunits

Nomenclature	CT receptor	calcitonin receptor-like receptor
HGNC, UniProt	*CALCR*, P30988	*CALCRL*, Q16602
Potency order of endogenous ligands	calcitonin (*CALCA*, P01258) ≥ amylin (*IAPP*, P10997), α-CGRP (*CALCA*, P06881), β-CGRP (*CALCB*, P10092) > adrenomedullin (*ADM*, P35318), adrenomedullin 2/ intermedin (*ADM2*, Q7Z4H4)	–
Endogenous agonists	calcitonin (*CALCA*, P01258) [39, 78, 966, 1391, 1487, 1820]	–
Agonists	calcitonin (salmon) [39, 473, 850, 2041], pramlintide [823]	–
Antagonists	CT-(8-32) (salmon) (p*K*_B_ 8.2) [966], AC187 (p*K*_B_ 7.2) [966]	–
Labelled ligands	[^125^I]CT (human) (Agonist), [^125^I]CT (salmon) (Agonist)	–

**Comments**: It is important to note that a complication with the interpretation of pharmacological studies with AMY receptors in transfected cells is that most of this work has likely used a mixed population of receptors, encompassing RAMP-coupled CTR as well as CTR alone. This means that although in binding assays human calcitonin (*CALCA*, P01258) has low affinity for ^125^I-AMY binding sites, cells transfected with CTR and RAMPs can display potent CT functional responses. Transfection of human CTR with any RAMP can generate receptors with a high affinity for both salmon CT and AMY and varying affinity for different antagonists [455, 966, 967]. The major human CTR splice variant (hCT_(a)_, which does not contain an insert) with RAMP1 (*i.e*. the AMY_1(a)_ receptor) has a high affinity for CGRP [2709], unlike hCT_(a)_-RAMP3 (*i.e*. AMY_3(a)_ receptor) [455, 966]. However, the AMY receptor phenotype is RAMP-type, splice variant and cell-line-dependent [1794, 2104, 2584]. Emerging data suggests that AMY_1_ could be a second CGRP receptor [2232]. The ligands described have limited selectivity. Adrenomedullin has appreciable affinity for CGRP receptors. CGRP can show significant cross-reactivity at AMY receptors and AM_2_ receptors. Adrenomedullin 2/intermedin also has high affinity for the AM_2_ receptor [968]. CGRP-(8–37) acts as an antagonist of CGRP (*p*K_i_ ~8) and inhibits some AM and AMY responses (*p*K_i_ ~6–7). It is weak at CT receptors. Human AM-(22–52) has some selectivity towards AM receptors, but with modest potency (*p*K_i_ ~7), limiting its use [969]. Olcegepant (also known as BIBN4096BS, pK_i_~10.5) and telcagepant (also known as MK0974, pK_i_~9) are examples of the “gepant” class of small molecule antagonists. These are selective for the CGRP receptor over the AM receptors but depending on the compound, have variable affinity for the AMY_1_ receptor [775]. These antagonists tend to have higher affinity at primate receptors, compared to rodent receptors [1786, 2709].

G_s_ is a prominent route for effector coupling for CLR and CTR but other pathways (*e.g*. Ca^2+^, ERK, Akt), and G proteins can be activated [2232]. There is evidence that CGRP-RCP (a 148 amino-acid hydrophilic protein, *ASL* (P04424) is important for the coupling of CLR to adenylyl cyclase [672].

[^125^I]-Salmon CT is the most common radioligand for CTR but it has high affinity for AMY receptors and is also poorly reversible.

Further reading on Calcitonin receptorsHayDL
 (2018) Update on the pharmacology of calcitonin/CGRP family of peptides: IUPHAR Review 25. Br J Pharmacol
175: 3–1729059473
10.1111/bph.14075PMC5740251KatoJ
 (2015) Bench-to-bedside pharmacology of adrenomedullin. Eur J Pharmacol
764: 140–826144371
10.1016/j.ejphar.2015.06.061KotliarIB
 (2023) Elucidating the Interactome of G Protein-Coupled Receptors and Receptor Activity-Modifying Proteins. Pharmacol Rev
75: 1–3436757898
10.1124/pharmrev.120.000180PMC9832379RussellFA
 (2014) Calcitonin gene-related peptide: physiology and pathophysiology. Physiol Rev
94: 1099–14225287861
10.1152/physrev.00034.2013PMC4187032RussoAF
 (2023) CGRP physiology, pharmacology, and therapeutic targets: migraine and beyond. Physiol Rev
103: 1565–164436454715
10.1152/physrev.00059.2021PMC9988538

## Calcium-sensing receptor

G protein-coupled receptors → Calcium-sensing receptor

**Overview**: The calcium-sensing receptor (CaS, **provisional nomenclature as recommended by NC-IUPHAR** [712] **and subsequently updated** [1440]) responds to multiple endogenous ligands, including extracellular calcium and other divalent/trivalent cations, polyamines and polycationic peptides, L-amino acids (particularly L-Trp and L-Phe), glutathione and various peptide analogues, ionic strength and extracellular pH (reviewed in [1442]). While divalent/trivalent cations, polyamines and polycations are CaS receptor agonists [298, 2115], L-amino acids, glutamyl peptides, ionic strength and pH are allosteric modulators of agonist function [482, 712, 1027, 2113, 2114]. Indeed, L-amino acids have been identified as “co-agonists”, with both concomitant calcium and L-amino acid binding required for full receptor activation [795, 2918]. The sensitivity of the CaS receptor to primary agonists is increased by elevated extracellular pH [340] or decreased extracellular ionic strength [2114] while sensitivity is decreased by pathophysiological phosphate concentrations [372]. This receptor bears no sequence or structural relation to the plant calcium receptor, also called CaS.

**Table:** 

Nomenclature	CaS receptor
HGNC, UniProt	*CASR*, P41180
Amino-acid rank order of potency	L-phenylalanine, L-tryptophan, L-histidine > L-alanine > L-serine, L-proline, L-glutamic acid > L-aspartic acid (not L-lysine, L-arginine, L-leucine and L-isoleucine) [482]
Cation rank order of potency	Gd^3+^ > Ca^2+^ > Mg^2+^ [298]
Glutamyl peptide rank order of potency	S-methylglutathione ≈ γGlu-Val-Gly > glutathione > γGlu-Cys [287, 1936, 2730]
Polyamine rank order of potency	spermine > spermidine > putrescine [2115]
Allosteric modulators (Positive)	upacicalcet (pIC_50_ 8.1) [2272], evocalcet (pEC_50_ 7) [1755], cinacalcet (p*K*_B_ 5.9–6.6) [485, 536, 1439, 1443], tecalcet (p*K*_B_ 6.2–6.6) [485, 536], AC265347 (p*K*_B_ 6.3–6.4) [485, 1439], calindol (p*K*_B_ 6.3) [485], etelcalcetide (pEC_50_ 4.6) [2712]
Allosteric modulators (Negative)	ATF936 (pIC_50_ 8.9) [2782], encaleret (pIC_50_ 7.9) [2371], SB-423562 (pIC_50_ 7.1) [1382], ronacaleret (pIC_50_ 6.5–6.8) [123], NPS 2143 (p*K*_B_ 6.2–6.7) [536, 1439, 1443], calhex 231 (pIC_50_ 6.4) [2037]

**Comments**: The CaS receptor has a number of physiological functions, but it is best known for its central role in parathyroid and renal regulation of extracellular calcium homeostasis [934]. This is seen most clearly in patients with loss-of-function CaS receptor mutations who develop familial hypocalciuric hypercalcaemia (heterozygous mutations) or neonatal severe hyperparathyroidism (heterozygous, compound heterozygous or homozygous mutations) [934] and in *Casr* null mice [388, 1027], which exhibit similar increases in PTH secretion and blood calcium levels. Gain-of-function CaS mutations are associated with autosomal dominant hypocalcaemia and Bartter syndrome type V [934].

The CaS receptor primarily couples to G_q/11_, G_12/13_ and G_i/o_ [536, 806, 1074, 2575], but in some cell types can couple to G_s_ [1632]. The CaS receptor acts as a homodimer [2918]. However, the CaS receptor can also form heteromers with Class C GABA_B_ [389, 423] and mGlu1/5 receptors [760], which may introduce further complexity in its signalling capabilities.

Multiple other small molecule chemotypes are positive and negative allosteric modulators of the CaS receptor [1261, 1876]. Further, etelcalcetide is a novel peptide positive allosteric modulator of the receptor, that also displays weak agonist activity [2712]. Agonists and positive allosteric modulators of the CaS receptor are termed Type I and II calcimimetics, respectively, and can suppress parathyroid hormone (PTH (*PTH*, P01270)) secretion [1878]. Negative allosteric modulators are called calcilytics and can act to increase PTH (*PTH*, P01270) secretion [1877].

Where functional pK_B_ values are provided for allosteric modulators, this refers to ligand affinity determined in an assay that measures a functional readout of receptor activity (*i.e*. a receptor signalling assay), as opposed to affinity determined in a radioligand binding assay. The functional pK_B_ may differ depending on the signalling pathway studied. Consult the ’More detailed page’ for the assay description, as well as other functional readouts.

Further reading on Calcium-sensing receptorBrownEM. (2013) Role of the calcium-sensing receptor in extracellular calcium homeostasis. Best Pract Res Clin Endocrinol Metab
27: 333–4323856263
10.1016/j.beem.2013.02.006ConigraveAD
 (2013) Calcium-sensing receptor (CaSR): pharmacological properties and signaling pathways. Best Pract Res Clin Endocrinol Metab
27: 315–3123856262
10.1016/j.beem.2013.05.010HannanFM
 (2018) The calcium-sensing receptor in physiology and in calcitropic and non-calcitropic diseases. Nat Rev Endocrinol
15: 33–5130443043
10.1038/s41574-018-0115-0PMC6535143LeachK
 (2020) International Union of Basic and Clinical Pharmacology. CVIII. Calcium-Sensing Receptor Nomenclature, Pharmacology, and Function. Pharmacol Rev
72: 558–60432467152
10.1124/pr.119.018531PMC7116503NemethEF
 (2018) Discovery and Development of Calcimimetic and Calcilytic Compounds. Prog Med Chem
57: 1–8629680147
10.1016/bs.pmch.2017.12.001

## Cannabinoid receptors

G protein-coupled receptors → Cannabinoid receptors

**Overview**: Cannabinoid receptors (**nomenclature as agreed by the NC-IUPHAR Subcommittee on Cannabinoid Receptors** [2029]) are activated by endogenous ligands that include N-arachidonoylethanolamine (anandamide), N-homo-γ-linolenoylethanolamine, N-docosatetra-7,10,13,16-enoylethanolamine and 2-arachidonoylglycerol. Potency determinations of endogenous agonists at these receptors are complicated by the possibility of differential susceptibility of endogenous ligands to enzymatic conversion [43].

There are currently three licenced cannabinoid medicines each of which contains a compound that can activate CB_1_ and CB_2_ receptors [2027]. Two of these medicines were developed to suppress nausea and vomiting produced by chemotherapy. These are nabilone (Cesamet^®^), a synthetic CB_1_/CB_2_ receptor agonist, and synthetic Δ^9^-tetrahydrocannabinol (Marinol^®^; dronabinol), which can also be used as an appetite stimulant. The third medicine, Sativex^®^, contains mainly Δ^9^-tetrahydrocannabinol and cannabidiol, both extracted from cannabis, and is used to treat multiple sclerosis and cancer pain.

**Table:** 

Nomenclature	CB_1_ receptor	CB_2_ receptor
HGNC, UniProt	*CNR1*, P21554	*CNR2*, P34972
Agonists	HU-210 [685, 2377], CP55940 [685, 2211, 2377], WIN55212-2 [685, 2374, 2377], Δ^9^-tetrahydrocannabinol (Partial agonist) [685, 2377], cannabinol (Partial agonist) [685, 2377]	HU-210 [685, 2171, 2377], WIN55212-2 [685, 2374, 2377], CP55940 [685, 2211, 2377], Δ^9^-tetrahydrocannabinol (Partial agonist) [148, 685, 2171, 2377]
Selective agonists	arachidonyl-2-chloroethylamide [1015] – Rat, arachidonylcyclopropylamide [1015] – Rat, O-1812 [574] – Rat, R-(+)-methanandamide [1255] – Rat	JWH-133 [1083, 2028], L-759,633 [773, 2211], AM1241 [2875], L-759,656 [773, 2211], onternabez [942], GW405833 (Partial agonist) [1653]
Selective antagonists	JD5037 (pKi 9.5) [2533], rimonabant (p*K*_i_ 7.9–8.7) [684, 685, 2181, 2227, 2377], AM6545 (p*K*_i_ 8.5) [257], AM251 (p*K*_i_ 8.1) [1404] – Rat, AM281 (p*K*_i_ 7.9) [1403] – Rat, LY320135 (p*K*_i_ 6.9) [684]	SR144528 (p*K*_i_ 8.3–9.2) [2182, 2211], AM-630 (p*K*_i_ 7.5) [2211]
Allosteric modulators (Positive)	ZCZ011 (pEC_50_ 6.3) [1099] – Mouse, GAT211 [1417]	pepcan-12 (p*K*_i_ ~7.3) [2040], compound C2 [751]
Allosteric modulators (Negative)	GAT100 (pEC_50_ 7.7) [1377], cannabidiol [1416]	–
Labelled ligands	[^3^H]rimonabant (Antagonist) (p*K*_d_ 8.9–10) [271, 1022, 1195, 2036, 2183, 2388, 2568] – Rat	–

**Comments**: Both CB_1_ and CB_2_ receptors may be labelled with [^3^H]CP55940 (0.5 nM; [2377]) and [^3^H]WIN55212-2 (2–2.4 nM; [2405, 2434]). Anandamide is also an agonist at vanilloid receptors (TRPV1) and PPARs [1922, 2964]. There is evidence for an allosteric site on the CB_1_ receptor [2082]. All of the compounds listed as antagonists behave as inverse agonists in some bioassay systems [2029]. For some cannabinoid receptor ligands, additional pharmacological targets that include GPR55 and GPR119 have been identified [2029]. Moreover, GPR18, GPR55 and GPR119, although showing little structural similarity to CB_1_ and CB_2_ receptors, respond to endogenous agents that are structural ly similar to the endogenous cannabinoid ligands [2029].

Further reading on Cannabinoid receptorsHowlettAC
 (2002) International Union of Pharmacology. XXVII. Classification of cannabinoid receptors. Pharmacol Rev
54: 161–20212037135
10.1124/pr.54.2.161PertweeRG. (2010) Receptors and channels targeted by synthetic cannabinoid receptor agonists and antagonists. Curr Med Chem
17: 1360–8120166927
10.2174/092986710790980050PMC3013229PertweeRG
 (2010) International Union of Basic and Clinical Pharmacology. LXXIX. Cannabinoid receptors and their ligands: beyond CB_1_ and CB_2_. Pharmacol Rev
62: 588–63121079038
10.1124/pr.110.003004PMC2993256

## Chemerin receptors

G protein-coupled receptors → Chemerin receptors

**Overview**: Nomenclature for the chemerin receptors is presented as **recommended by NC-IUPHAR** [532, 1243]). The chemoattractant protein and adipokine, chemerin (*RARRES2*, Q99969), has been shown to be the endogenous ligand for both chemerin family receptors. Chemerin_1_ was the founding family member, and when *GPR1* was de-orphanised it was re-named Chermerin_2_ [1243]. Chemerin_1_ is also activated by the lipid-derived, anti-inflammatory ligand resolvin E1 (RvE1), which is formed *via* the sequential metabolism of EPA by aspirin-modified cyclooxygenase and lipoxygenase [76, 77]. In addition, two GPCRs for resolvin D1 (RvD1) have been identified: FPR2/ALX, the lipoxin A_4_ receptor, and GPR32, an orphan receptor [1359].

**Table:** 

Nomenclature	chemerin receptor 1	chemerin receptor 2
Common abbreviation	Chemerin_1_	Chemerin_2_
HGNC, UniProt	*CMKLR1*, Q99788	*CMKLR2*, P46091
Potency order of endogenous ligands	resolvin E1 > chemerin C-terminal peptide > 18R-HEPE > EPA [76]	–
Endogenous agonists	–	chemerin (RARRES2, Q99969) [133]
Selective agonists	resolvin E1	–
Labelled ligands	[^3^H]resolvin E1 (Agonist) [76, 77]	–
Comments	–	Reported to act as a co-receptor for HIV [2366]. See review [532] for discussion of pairing with chemerin.

**Comments**: CCX832 (structure not disclosed) is a selective antagonist, p*K*_i_=9.2 [1245].

Further reading on Chemerin receptorsKennedyAJ
 (2018) International Union of Basic and Clinical Pharmacology CIII: Chemerin Receptors CMKLR1 (Chemerin1) and GPR1 (Chemerin2) Nomenclature, Pharmacology, and Function. Pharmacol Rev
70: 174–19629279348
10.1124/pr.116.013177PMC5744648ShinWJ
 (2018) Mechanisms and Functions of Chemerin in Cancer: Potential Roles in Thera peutic Intervention. Front Immunol
9: 277230555465
10.3389/fimmu.2018.02772PMC6283908

## Chemokine receptors

G protein-coupled receptors → Chemokine receptors

**Overview**: Chemokine receptors (**nomenclature as agreed by the NC-IUPHAR Subcommittee on Chemokine Receptors** [107, 1833, 1834]) comprise a large subfamily of 7TM proteins that bind one or more chemokines, a large family of small cytokines typically possessing chemotactic activity for leukocytes. Additional hematopoietic and non-hematopoietic roles have been identified for many chemokines in the areas of embryonic development, immune cell proliferation, activation and death, viral infection, and as antibacterials, among others. Chemokine receptors can be divided by function into two main groups: G protein-coupled chemokine receptors, which mediate leukocyte trafficking, and “Atypical chemokine receptors”, which may signal through non-G protein-coupled mechanisms and act as chemokine scavengers to downregulate inflammation or shape chemokine gradients [107].

Chemokines in turn can be divided by structure into four subclasses by the number and arrangement of conserved cysteines. CC (also known as β-chemokines; *n*= 28), CXC (also known as α-chemokines; *n*= 17) and CX3C (*n*= 1) chemokines all have four conserved cysteines, with zero, one and three amino acids separating the first two cysteines respectively. C chemokines (*n*= 2) have only the second and fourth cysteines found in other chemokines. Chemokines can also be classified by function into homeostatic and inflammatory subgroups. Most chemokine receptors are able to bind multiple high-affinity chemokine ligands, but the ligands for a given receptor are almost always restricted to the same structural subclass. Most chemokines bind to more than one receptor subtype. Receptors for inflammatory chemokines are typically highly promiscuous with regard to ligand specificity, and may lack a selective endogenous ligand. G protein-coupled chemokine receptors are named acccording to the class of chemokines bound, whereas ACKR is the root acronym for atypical chemokine receptors [108]. There can be substantial cross-species differences in the sequences of both chemokines and chemokine receptors, and in the pharmacology and biology of chemokine receptors. Endogenous and microbial non-chemokine ligands have also been identified for chemokine receptors. Many chemokine receptors function as HIV co-receptors, but CCR5 is the only one demonstrated to play an essential role in HIV/AIDS pathogenesis. The tables include both standard chemokine receptor names [2960] and aliases.

**Table:** 

Nomenclature	CCR1	CCR2	CCR3
HGNC, UniProt	*CCR1*, P32246	*CCR2*, P41597	*CCR3*, P51677
Endogenous agonists	CCL3 (*CCL3*, P10147) [442, 475, 1005, 2962], CCL23 (*CCL23*, P55773) [442], CCL5 (*CCL5*, P13501) [475, 1005], CCL7 (*CCL7*, P80098) [442, 906], CCL15 (*CCL15*, Q16663) [495], CCL14 (*CCL14*, Q16627) [442], CCL13 (*CCL13*, Q99616), CCL8 (*CCL8*, P80075)	CCL2 (*CCL2*, P13500) [495, 1584, 1748, 1989, 2627], CCL13 (*CCL13*, Q99616) [1584, 2627], CCL7 (*CCL7*, P80098) [495, 1584, 2627], CCL11 (*CCL11*, P51671) (Partial agonist) [1584, 1989], CCL16 (*CCL16*, O15467)	CCL13 (*CCL13*, Q99616) [1807, 2627], CCL24 (*CCL24*, O00175) [1807, 1989], CCL5 (*CCL5*, P13501) [526], CCL7 (*CCL7*, P80098) [526], CCL11 (*CCL11*, P51671) [624, 1298, 1807, 2244, 2627], CCL26 (*CCL26*, Q9Y258) [1298, 1807, 1989], CCL15 (*CCL15*, Q16663) [495], CCL28 (*CCL28*, Q9NRJ3), CCL8 (*CCL8*, P80075)
Agonists	–	–	CCL11 {Mouse} [526]
Endogenous antagonists	CCL4 (*CCL4*, P13236) (p*K*_i_ 7.1–7.8) [442, 475]	CCL26 (*CCL26*, Q9Y258) (pIC_50_ 8.5) [1989]	CXCL10 (*CXCL10*, P02778), CXCL11 (*CXCL11*, O14625), CXCL9 (*CXCL9*, Q07325)
Selective antagonists	BX 471 (p*K*_i_ 8.2–9) [1508], compound 2b-1 (pIC_50_ 8.7) [1863], UCB35625 (pIC_50_ 8) [2244], CP-481,715 (p*K*_d_ 8) [826]	GSK Compound 34 (p*K*_i_ 7.6)	banyu (I) (Inverse agonist) (p*K*_i_ 8.5) [2717], SB328437 (p*K*_i_ 8.4), BMS compound 87b (p*K*_i_ 8.1) [2702]
Labelled ligands	[^125^I]CCL7 (human) (Agonist) [174], [^125^I]CCL3 (human) (Agonist) [174, 840, 2270], [^125^I]CCL5 (human) (Agonist) [2270]	[^125^I]CCL2 (human) (Agonist), [^125^I]CCL7 (human) (Agonist)	[^125^I]CCL11 (human) (Antagonist) (p*K*_d_ 8.3) [2717], [^125^I] CCL5 (human) (Agonist), [^125^I]CCL7 (human) (Agonist)

**Table:** 

Nomenclature	CCR4	CCR5	CCR6	CCR7	CCR8	CCR9	CCR10
HGNC, UniProt	*CCR4*, P51679	*CCR5*, P51681	*CCR6*, P51684	*CCR7**,* P32248	*CCR8*, P51685	*CCR9*, P51686	*CCR10*, P46092
Endogenous agonists	CCL22 (*CCL22*, O00626) [1105], CCL17 (*CCL17*, Q92583) [1105]	CCL5 (*CCL5*, P13501) [104, 1855, 2224], CCL4 (*CCL4*, P13236) [1855, 2224], CCL8 (*CCL8*, P80075) [2224], CCL3 (*CCL3*, P10147) [1855, 2224, 2962], CCL11 (*CCL11*, P51671) [212], CCL2 (*CCL2*, P13500) [1855], CCL14 (*CCL14*, Q16627) [1855], CCL16 (*CCL16*, O15467)	CCL20 (*CCL20*, P78556) [26, 103, 2076], beta-defensin 4A (*DEFB4A* *DEFB4B*, O15263) [2865]	CCL21 (*CCL21*, O00585) [2896], CCL19 (*CCL19*, Q99731) [1963, 2895, 2896]	CCL1 (*CCL1*, P22362) [517, 957, 1107], CCL8 (*CCL8*, P80075)	CCL25 (*CCL25*, O15444)	CCL27 (*CCL27*, Q9Y4X3) [1046], CCL28 (*CCL28*, Q9NRJ3)
Agonists	vMIP-III	R5-HIV-1 gp120	–	–	vMIP-I [517, 1107]	–	–
Endogenous antagonists	–	CCL7 (*CCL7*, P80098) (p*K*_i_ 7.5) [1855] vMIP-II (pIC_50_ 8.3) [1304]	–	–	–	–	–
Antagonists	–	vMIP-II (pIC_50_ 8.3) [1304]	–	–	vMIP-II (pIC_50_ 8.1) [517]	–	–
Selective antagonists	compound 8ic (pIC_50_ 7.7) [2893]	vicriviroc (p*K*_i_ 9.1) [2470], E913 (pIC_50_ 8.7) [1609], ancriviroc (p*K*_i_ 7.8–8.7) [1608, 1974, 2470], aplaviroc (p*K*_i_ 8.5) [1608], maraviroc (pIC_50_ 8.1) [1855], TAK-779 (p*K*_i_ 7.5) [1608], MRK-1 [1381] – Rat	–	–	–	–	–
Selective allosteric modulators	–	–	–	–	–	vercirnon (Antagonist) (pIC_50_ 8.2) [2713]	–
Antibodies	mogamulizumab (Inhibition) [67, 2375]	leronlimab (Binding) [1955]	–	–	–	–	–
Labelled ligands	[^125^I]CCL17 (human) (Agonist), [^125^I]CCL27 (human) (Agonist)	[^125^I]CCL4 (human) (Agonist) [1855], [^125^I]CCL3 (human) (Agonist), [^125^I]CCL5 (human) (Agonist), [^125^I]CCL8 (human) (Agonist)	[^125^I]CCL20 (human) (Agonist) [868]	[^125^I]CCL19 (human) (Agonist), [^125^I]CCL21 (human) (Agonist) [1158]	[^125^I]CCL1 (human) (Agonist) [1107, 2206]	[^125^I]CCL25 (human) (Agonist)	–

**Table:** 

Nomenclature	CXCR1	CXCR2	CXCR3	CXCR4	CXCR5	CXCR6	CX_3_CR1
HGNC, UniProt	*CXCR1*, P25024	*CXCR2*, P25025	*CXCR3*, P49682	*CXCR4*, P61073	*CXCR5*, P32302	*CXCR6*, O00574	*CX3CR1*, P49238
Endogenous agonists	CXCL8 (*CXCL8*, P10145) [191, 917, 1455, 2795, 2819], CXCL6 (*CXCL6*, P80162) [2825]	CXCL1 (*CXCL1*, P09341) [917, 1455, 2819], CXCL8 (*CXCL8*, P10145) [191, 917, 1455, 2795, 2819], CXCL7 (*PPBP*, P02775) [24], CXCL3 (*CXCL3*, P19876) [24], CXCL2 (*CXCL2*, P19875) [24], CXCL5 (*CXCL5*, P42830) [24], CXCL6 (*CXCL6*, P80162) [2825]	CXCL11 (*CXCL11*, O14625) [986], CXCL10 (*CXCL10*, P02778) [986, 2764], CXCL9 (*CXCL9*, Q07325) [986, 2764]	CXCL12α (*CXCL12*, P48061) [1004, 1559], CXCL12β (*CXCL12*, P48061) [1004]	CXCL13 (*CXCL13*, O43927) [137]	CXCL16 (*CXCL16*, Q9H2A7) [2788]	CX_3_CL1 (*CX3CL1*, P78423) [776]
Agonists	vCXCL1 [1583]	vCXCL1 [1583], HIV-1 matrix protein p17 [811]	–	–	–	–	–
Selective agonists	–	–	–	ALX40-4C (Partial agonist) [2936], X4-HIV-1 gp120	–	–	–
Endogenous antagonists	–	–	CCL11 (*CCL11*, P51671) (p*K*_i_ 7.2) [2764], CCL7 (*CCL7*, P80098) (p*K*_i_ 6.6) [2764]	–	–	–	–
Antagonists	–	–	–	plerixafor (p*K*_i_ 7) [2936]	–	–	–
Selective antagonists	–	navarixin (pIC_50_ 10.3) [107, 629], danirixin (pIC_50_ 7.9) [1741], SB 225002 (pIC_50_ 7.7) [2776], elubirixin (pIC_50_ 7.7) [107], SX-517 (pIC_50_ 7.2) [1607]	–	T134 (pIC_50_ 8.4) [2534], mavorixafor (pIC_50_ 7.9) [2397], HIV-Tat	–	–	–
Allosteric modulators (Negative)	–	reparixin (pIC_50_ 6.4) [191]	–	–	–	–	–
Labelled ligands	[^125^I]CXCL8 (human) (Agonist) [917, 2179]	[^125^I]CXCL8 (human) (Agonist) [917, 2179], [^125^I]CXCL1 (human) (Agonist), [^125^I]CXCL5 (human) (Agonist), [^125^I]CXCL7 (human) (Agonist)	[^125^I]CXCL10 (human) (Agonist), [^125^I] CXCL11 (human) (Agonist)	[^125^I]CXCL12α (human) (Agonist) [577, 1004]	[^125^I]CXCL13 (mouse) (Agonist) [291] – Mouse	[^125^I]CXCL16 (human) (Agonist)	[^125^I]CX_3_CL1 (human) (Agonist)

**Table:** 

Nomenclature	XCR1	ACKR1	ACKR2	ACKR3	ACKR4
HGNC, UniProt	*XCR1*,P46094	*ACKR1*, Q16570	*ACKR2*,O00590	*ACKR3*, P25106	*ACKR4*, Q9NPB9
Endogenous ligands	–	CXCL5 (*CXCL5*, P42830), CXCL6 (*CXCL6*, P80162), CXCL8 (*CXCL8*, P10145), CXCL11 (*CXCL11*, O14625), CCL2 (*CCL2*, P13500), CCL5 (*CCL5*, P13501), CCL7 (*CCL7*, P80098), CCL11 (*CCL11*, P51671), CCL14 (*CCL14*, Q16627), CCL17 (*CCL17*, Q92583)	–	–	–
Endogenous agonists	XCL1 (*XCL1*, P47992) [721], XCL2 (*XCL2*, Q9UBD3) [721]	–	CCL2 (*CCL2*, P13500), CCL3 (*CCL3*, P10147), CCL4 (*CCL4*, P13236), CCL5 (*CCL5*, P13501), CCL7 (*CCL7*, P80098), CCL8 (*CCL8*, P80075), CCL11 (*CCL11*, P51671), CCL13 (*CCL13*, Q99616), CCL14 (*CCL14*, Q16627), CCL17 (*CCL17*, Q92583), CCL22 (*CCL22*, O00626)	CXCL12*α* (*CXCL12*, P48061) [867, 2436], CXCL11 (*CXCL11*, O14625)	CCL19 (*CCL19*, Q99731) [2754], CCL25 (*CCL25*, O15444) [2754], CCL21 (*CCL21*, O00585) [2754]
Selective antagonists	–	–	–	LIH383 (pEC_50_ 9.2) [1718]	–
Comments	XCL1 cannot be iodinated, but a secreted alkaline phophatase (SEAP)-XCL1 fusion peptide can be used as a probe at XCR1.	ACKR1 is used by *Plasmodium vivax* and *Plasmodium knowlsei* for entering erythrocytes.	–	Several lines of evidence have suggested that CGRP and adrenomedullin could be ligands for ACKR3; however, classical direct binding to the receptor has not yet been convincingly demonstrated [2509].	–

**Comments**: Specific chemokine receptors facilitate cell entry by microbes, such as ACKR1 for *Plasmodium vivax*, and CCR5 and CXCR4 for HIV-1. Virally encoded chemokine receptors are known (*e.g*. US28, a homologue of CCR1 from human cytomegalovirus and ORF74, which encodes a homolog of CXCR2 in *Herpesvirus saimiri* and gamma-Herpesvirus-68), but their role in viral life cycles is not established. Viruses can exploit or subvert the chemokine system by producing chemokine antagonists and scavengers. Three chemokine receptor antagonists have now been approved by the FDA: 1) the CCR5 antagonist maraviroc (Pfizer) for treatment of HIV/AIDS in patients with CCR5-using strains; and 2) the CXCR4 antagonist plerixafor (Sanofi) for hematopoietic stem cell mobilization with G-CSF (*CSF3*, P09919) in patients undergoing transplantation in the context of chemotherapy for Hodgkins’ Disease and multiple myeloma; and 3) the CCR4 blocking antibody Poteligeo (mogamulizumab-kpkc, Kyowa Kirin, Inc.) for mycosis fungoides or Sezary syndrome.

Further reading on Chemokine receptorsBachelerieF
 (2014) International Union of Pharmacology. LXXXIX. Update on the extended family of chemokine receptors and introducing a new nomenclature for atypical chemokine receptors. Pharmacol Rev
66: 1–7924218476
10.1124/pr.113.007724PMC3880466BachelerieF
 (2015) An atypical addition to the chemokine receptor nomenclature: IUPHAR Review 15. Br J Pharmacol
172: 3945–925958743
10.1111/bph.13182PMC4543604KoelinkPJ
 (2012) Targeting chemokine receptors in chronic inflammatory diseases: an extensive review. Pharmacol Ther
133: 1–1821839114
10.1016/j.pharmthera.2011.06.008MurphyPM. (2002) International Union of Pharmacology. XXX. Update on chemokine receptor nomenclature. Pharmacol Rev
54: 227–912037138
10.1124/pr.54.2.227MurphyPM
 (2000) International union of pharmacology. XXII. Nomenclature for chemokine receptors. Pharmacol Rev
52: 145–7610699158
ScholtenDJ
 (2012) Pharmacological modulation of chemokine receptor function. Br J Pharmacol
165: 1617–4321699506
10.1111/j.1476-5381.2011.01551.xPMC3372818

## Cholecystokinin receptors

G protein-coupled receptors → Cholecystokinin receptors

**Overview**: Cholecystokinin receptors (**nomenclature as agreed by the NC-IUPHAR Subcommittee on CCK receptors** [1909]) are activated by the endogenous peptides cholecystokinin-8 (CCK-8 (*CCK*, P06307)), CCK-33 (*CCK*, P06307), CCK-58 (*CCK*, P06307) and gastrin (gastrin-17 (*GAST*, P01350)). There are only two distinct subtypes of CCK receptors, CCK_1_ and CCK_2_ receptors [1339, 2741], with some alternatively spliced forms most often identified in neoplastic cells. The CCK receptor subtypes are distinguished by their peptide selectivity, with the CCK_1_ receptor requiring the carboxyl-terminal heptapeptide-amide that includes a sulfated tyrosine for high affinity and potency, while the CCK_2_ receptor requires only the carboxyl-terminal tetrapeptide shared by each CCK and gastrin peptides. These receptors have characteristic and distinct distributions, with both present in both the central nervous system and peripheral tissues.

**Table:** 

Nomenclature	CCK_1_ receptor	CCK_2_ receptor
HGNC, UniProt	*CCKAR*, P32238	*CCKBR*, P32239
Potency order of endogenous ligands	CCK-8 (*CCK*, P06307), CCK-58 (*CCK*, P06307), CCK-39 (*CCK*), CCK-33 (*CCK*, P06307) ≫ gastrin-17 (*GAST*, P01350), desulfated cholecystokinin-8 > CCK-4 (*CCK*, P06307)	CCK-8 (*CCK*,P06307), CCK-39 (*CCK*), CCK-33 (*CCK*, P06307), CCK-58 (*CCK*, P06307) ≥ gastrin-17 (*GAST*, P01350), desulfated cholecystokinin-8, CCK-4 (*CCK*, P06307)
Endogenous agonists	CCK-33 (*CCK*,P06307), CCK-39 (*CCK*), CCK-58 (*CCK*,P06307), CCK-8 (*CCK*,P06307)	desulfated cholecystokinin-8 [1458], gastrin-17 (*GAST*,P01350) [1085] – Mouse, CCK-4 (*CCK*, P06307) [1120], desulfated gastrin-14 (*GAST*, P01350), desulfated gastrin-17 (*GAST*, P01350), desulfated gastrin-34 (*GAST*, P01350), desulfated gastrin-71 (*GAST*, P01350), gastrin-14 (*GAST*, P01350), gastrin-34 (*GAST*, P01350), gastrin-71 (*GAST*, P01350)
Selective agonists	A-71623 [86] – Rat, JMV180 [1247], GW-5823 [994]	RB-400 [172] – Rat, PBC-264 [1140] – Rat
Antagonists	lintitript (pIC_50_ 8.3) [858]	–
Selective antagonists	devazepide (pIC_50_ 9.7) [1085] – Rat, T-0632 (pIC_50_ 9.6) [2547] – Rat, PD-140548 (pIC_50_ 8.6) [2394] – Rat, lorglumide (pIC_50_ 6.7–8.2) [1085, 1126] – Rat	YF-476 (pIC_50_ 9.7) [258, 2531], GV150013 (pIC_50_ 9.4) [2641], L-740093 (pIC_50_ 9.2) [1899], YM-022 (pIC_50_ 9.2) [1899], JNJ-26070109 (pIC_50_ 8.5) [1813], L-365260 (pIC_50_ 8.4) [1458], RP73870 (pIC_50_ 8) [1533] – Rat, LY262691 (pIC_50_ 7.5) [2142] – Rat
Labelled ligands	[^3^H]devazepide (Antagonist) (p*K*_d_ 9.7) [387], [^125^I]DTyr-Gly-[(Nle28,31) CCK-26-33 (Agonist) [2078]	[^3^H]PD140376 (Antagonist) (p*K*_i_ 9.7–10) [1089] – Guinea pig, [^125^I] PD142308 (Antagonist) (p*K*_d_ 9.6) [1053] – Guinea pig, [^125^I]DTyr-Gly-[(Nle28,31)CCK-26-33 (Agonist) [2078], [^125^I]gastrin (Agonist), [^3^H] gastrin (Agonist), [^3^H]L365260 (Antagonist) (p*K*_d_ 8.2–8.5) [1899], [^125^I]-BDZ2 (Antagonist) (p*K*_i_ 8.4) [31]

**Comments**: While a cancer-specific CCK receptor has been postulated to exist, which also might be responsive to incompletely processed forms of CCK (Gly-extended forms), this has never been isolated. An alternatively spliced form of the CCK_2_ receptor in which intron 4 is retained, adding 69 amino acids to the intracellular loop 3 (ICL3) region, has been described to be present particularly in certain neoplasms where mRNA mis-splicing has been commonly observed [2412], but it is not clear that this receptor splice form plays a special role in carcinogenesis. Another alternative splicing event for the CCK_2_ receptor was reported [2433], with alternative donor sites in exon 4 resulting in long (452 amino acids) and short (447 amino acids) forms of the receptor differing by five residues in ICL3, however, no clear functional differences have been observed.

Further reading on Cholecystokinin receptorsCawstonEE
 (2010) Therapeutic potential for novel drugs targeting the type 1 cholecystokinin receptor. Br J Pharmacol
159: 1009–2119922535
10.1111/j.1476-5381.2009.00489.xPMC2839260DockrayGJ. (2009) Cholecystokinin and gut-brain signalling. Regul Pept
155: 6–1019345244
10.1016/j.regpep.2009.03.015DufresneM
 (2006) Cholecystokinin and gastrin receptors. Physiol Rev
86: 805–4716816139
10.1152/physrev.00014.2005HerranzR. (2003) Cholecystokinin antagonists: pharmacological and therapeutic potential. Med Res Rev
23: 559–60512789687
10.1002/med.10042MillerLJ
 (2008) Structural basis of cholecystokinin receptor binding and regulation. Pharmacol Ther
119: 83–9518558433
10.1016/j.pharmthera.2008.05.001PMC2570585NobleF
 (1999) International Union of Pharmacology. XXI. Structure, distribution, and functions of cholecystokinin receptors. Pharmacol Rev
51: 745–78110581329


## Class Frizzled GPCRs

G protein-coupled receptors → Class Frizzled GPCRs

**Overview**: Receptors of the Class Frizzled (FZD, **nomenclature as agreed by the NC-IUPHAR subcommittee on the Class Frizzled GPCRs** [2312]), are GPCRs originally identified in *Drosophila* [380], which are highly conserved across species. While SMO shows structural resemblance to the 10 FZDs, it is functionally separated as it is involved in the Hedgehog signaling pathway [2312]. SMO exerts its effects by activating heterotrimeric G proteins or stabilization of GLI by sequestering catalytic PKA subunits [83, 943, 2358]. While SMO itself is bound by sterols and oxysterols [488, 1288], FZDs are activated by WNTs, which are cysteine-rich lipoglycoproteins with fundamental functions in ontogeny and tissue homeostasis. FZD signalling was initially divided into two pathways, being either dependent on the accumulation of the transcription regulator β-catenin (*CTNNB1*, P35222) or being β-catenin-independent (often referred to as canonical *vs*. non-canonical WNT/FZD signalling, respectively). WNT stimulation of FZDs can, in cooperation with the low density lipoprotein receptors *LRP5* (O75197) and *LRP6* (O75581), lead to the inhibition of a constitutively active destruction complex, which results in the accumulation of β-catenin and subsequently its translocation to the nucleus. β-catenin, in turn, modifies gene transcription by interacting with TCF/LEF transcription factors. WNT/β-catenin-dependent signalling can also be activated by FZD subtype-specific WNT surrogates [1722]. β-catenin-independent FZD signalling is far more complex with regard to the diversity of the activated pathways. WNT/FZD signalling can lead to the activation of heterotrimeric G proteins [584, 2032, 2313], the elevation of intracellular calcium [2406], activation of cGMP-specific PDE6 [25] and elevation of cAMP as well as RAC-1, JNK, Rho and Rho kinase signalling [938]. Novel resonance energy transfer-based tools have allowed the study of the GPCR-like nature of FZDs in greater detail. Upon ligand stimulation, FZDs undergo conformational changes and signal *via* heterotrimeric G proteins [1349, 1353, 2292, 2813, 2814]. Furthermore, the phosphoprotein Dishevelled constitutes a key player in WNT/FZD signalling towards planar-cell-polarity-like pathways. Importantly, FZDs exist in at least two distinct conformational states that regulate pathway selection [2814]. As with other GPCRs, members of the Frizzled family are functionally dependent on the arrestin scaffolding protein for internalization [410], as well as for β-catenin-dependent [309] and -independent [310, 1270] signalling. The pattern of cell signalling is complicated by the presence of additional ligands, which can enhance or inhibit FZD signalling (secreted Frizzled-related proteins (sFRP), Wnt-inhibitory factor (*WIF1*, Q9Y5W5) (WIF), sclerostin (*SOST*, Q9BQB4) or Dickkopf (DKK)), as well as modulatory (co)-receptors with Ryk, ROR1, ROR2 and Kremen, which may also function as independent signalling proteins.

**Table:** 

Nomenclature	FZD_1_	FZD_2_	FZD_3_	FZD_4_	FZD_5_
HGNC, UniProt	*FZD1*, Q9UP38	*FZD2*, Q14332	*FZD3*, Q9NPG1	*FZD4*, Q9ULV1	*FZD5*, Q13467
Allosteric modulators (Positive)	–	–	–	FzM1.8 (pEC_50_ 6.4) [2174]	–
Allosteric modulators (Negative)	–	–	–	FzM1.8 (pIC_50_ 5.5–7.8) [792], FzM1 (pIC_50_ 6.2) [792, 2174]	–
Antibodies	vantictumab (Antagonist) (pIC_50_ ∼ 9.1) [903]	vantictumab (Antagonist) (pIC_50_ ∼9) [903]	–	–	vantictumab (Antagonist) (pIC_50_ ∼9) [903]
Comments	–	–	–	–	IgG-2919 and IgG-2921 are FZD_5_ antibodies that have exhibited antitumour activities in vitro and in vivo (inhibiting the growth of RNF43-mutant pancreatic ductal adenocarcinoma cells/xenograft tumours), by blocking autocrine Wnt-β-catenin signalling in these mutant, FZD_5_-dependent cells [2453].

**Table:** 

Nomenclature	FZD_6_	FZD_7_	FZD_8_	FZD_9_	FZD_10_
HGNC, UniProt	*FZD6*, O60353	*FZD7*, O75084	*FZD8*, Q9H461	*FZD9*, O00144	*FZD10*, Q9ULW2
Selective antagonists	–	Fz7-21 (pIC_50_ 7) [1898]	–	–	–
Allosteric modulators (Negative)	–	–	carbamazepine (p*K*_d_ 4.8) [2945]	–	–
Antibodies	–	vantictumab (Antagonist) (pIC_50_ ∼9) [903]	vantictumab (Antagonist) (pIC_50_ ∼8) [903]	–	–
Comments	SAG1.3 and purmorphamine have been described as weak partial agonists with varying potencies depending on a read-out [1353].	–	FZD8-Fc/OMP-54F28 is a FZD_8_ antagonist [556].	–	Radio-labelled murine monoclonal antibody MAb 92–13 has been used to demonstrate the therapeutic potential of targeting FZD_10_-positive tumours [745].

**Table:** 

Nomenclature	SMO
HGNC, UniProt	*SMO*, Q99835
Agonists	SAG1.3 [403] – Mouse, purmorphamine [2395]
Antagonists	MRT-92 (pK_d_ 9.5) [1032], SANT-1 (pK_i_ 7.7) [403] – Mouse, cyclopamine-KAAD (pIC_50_ 7.7) [2514] – Mouse, cyclopamine (pIC_50_ 697) [2621] – Mouse
Selective antagonists	vismodegib (pK_i_ 7.8) [2724]
Allosteric modulators (Positive)	GSA-10 (pEC_50_ 5.9) [851]
Comments	SANT-3 and SANT-4 are SMO antagonists [403]. Cyclopamine-KAAD can act as an inverse agonist [2814].

**Comments**: There is limited knowledge about WNT/FZD specificity and which molecular entities determine the signalling outcome of a specific WNT/FZD pair. Understanding of the FZD and SMO coupling to G proteins is incomplete, but progress have been made [82, 568, 584, 1267, 1639, 2105, 2106, 2185, 2358, 2697, 2813]. There is also a scarcity of information on basic pharmacological characteristics of FZDs, such as binding constants, ligand specificity or concentration-response relationships [1265]. However, progress in understanding WNT-FZD interactions has been initiated with generation of eGFP-tagged WNT-3A and its use in BRET-based binding assays [1352, 2515, 2772]. Development of pharmacological tools [1351] for SMO has been faciliated by successful determination of several SMO structures [329, 568, 1076, 1288, 2105, 2106, 2720, 2721, 2757, 2924, 2937]. The recently solved FZD_4_ and FZD_5_ structures in apo state, as well as the structure of FZD_7_ bound to a heterotrimeric Gs protein, have provided first insights into FZD transmembrane organization [2616, 2842, 2873].

### Ligands associated with FZD signalling

Wnts: Wnt-1 (*WNT1*, P04628), Wnt-2 (*WNT2*, P09544) (also known as Int-1-related protein), Wnt-2b (*WNT2B*, Q93097) (also known as WNT-13), Wnt-3 (*WNT3*, P56703) , Wnt-3a (*WNT3A*, P56704), Wnt-4 (*WNT4*, P56705), Wnt-5a (*WNT5A*, P41221) (pEC50 7.7–8.9 [2813]), Wnt-5b (*WNT5B*, Q9H1J7), Wnt-6 (*WNT6*, Q9Y6F9), Wnt-7a (*WNT7A*, O00755), Wnt-7b (*WNT7B*, P56706), Wnt-8a (*WNT8A*, Q9H1J5), Wnt-8b (*WNT8B*, Q93098), Wnt-9a (*WNT9A*,O14904) (also known as WNT-14), Wnt-9b (*WNT9B*, O14905) (also known as WNT-15 or WNT-14b), Wnt-10a (*WNT10A*,Q9GZT5), Wnt-10b (*WNT10B*, O00744) (also known as WNT-12), Wnt-11 (*WNT11*, O96014) and Wnt-16 (*WNT16*, Q9UBV4).

***Extracellular proteins that interact with FZDs:*** norrin (*NDP*, Q00604), R-spondin-4 (*RSPO4*, Q2I0M5), sFRP-1 (*SFRP1*, Q8N474), sFRP-2 (*SFRP2*, Q96HF1), sFRP-3 (*FRZB*, Q92765), sFRP-4 (*SFRP4*, Q6FHJ7), sFRP-5 (*SFRP5*, Q6FHJ7).

***Extracellular proteins that interact with WNTs or LRPs:*** Dickkopf 1 (*DKK1*, O94907), *WIF1* (Q9Y5W5), sclerostin (*SOST*,Q9BQB4), kremen 1 (*KREMEN1*, Q96MU8) and kremen 2 (*KREMEN2*,Q8NCW0)

***Small exogenous ligands:*** Foxy-5 [2245], Box-5 [1157], UM206 [1395], and XWnt8 (P28026) also known as mini-Wnt8.

**Ligands associated with SMO signalling:** cholesterol, oxysterols [329, 1574, 2125].

Further reading on Class Frizzled GPCRsAngersS
 (2009) Proximal events in Wnt signal transduction. Nat Rev Mol Cell Biol
10: 468–7719536106
10.1038/nrm2717KozielewiczP
 (2020) Molecular Pharmacology of Class F Receptor Activation. Mol Pharmacol
97: 62–7131591260
10.1124/mol.119.117986SchulteG
 (2021) In Pharmacology of the WNT Signaling System Edited by SchulteG, KozielewiczP: Springer Cham: 422 [ISBN: 9783030854980]SchulteG
 (2018) Frizzleds as GPCRs - More Conventional Than We Thought!
Trends Pharmacol Sci
39: 828–84230049420
10.1016/j.tips.2018.07.001van AmerongenR. (2012) Alternative Wnt pathways and receptors. Cold Spring Harb Perspect Biol
4:10.1101/cshperspect.a007914PMC347517422935904WangY
 (2016) Frizzled Receptors in Development and Disease. Curr Top Dev Biol
117: 113–3926969975
10.1016/bs.ctdb.2015.11.028PMC5103317

## Complement peptide receptors

G protein-coupled receptors → Complement peptide receptors

**Overview**: Complement peptide receptors (**nomenclature as agreed by the NC-IUPHAR subcommittee on Complement peptide receptors** [1307]) are activated by the endogenous ~75 amino-acid anaphylatoxin polypeptides C3a (*C3*, P01024) and C5a (*C5*, P01031), generated upon stimulation of the complement cascade. C3a and C5a exert their functions through binding to their receptors (C3a receptor, C5a receptor 1 and C5a receptor 2), causing cell recruitment and triggering cellular degranulation that contributes to local inflammation.

**Table:** 

Nomenclature	C3a receptor
HGNC, UniProt	*C3AR1*, Q16581
Potency order of endogenous ligands	C3a (*C3*, P01024) > C5a (*C5*, P01031) [51]
Agonists	E7 [53, 648, 2325], compound 17 [2159], compound 21 [2158], casoxin C [2517, 2898], albutensin A [1933, 2898], oryzatensin [1173, 2518, 2898]
Antagonists	JR14a (pIC_50_ 8) [2220], SB290157 (SB290157 has also been reported to have agonist properties at the C3a receptor, and act as a weak C5aR2 activator) (pIC_50_ 7.6) [50, 1503]
Labelled ligands	[^125^I]C3a (human) (Agonist) [391], Eu-DTPA-hC3a (Agonist) [520]
Comments	Dual pro- and anti-inflammatory roles of C3a receptor have been reported in pathological conditions [498]. In particular, C3 and the C3a receptor have been identified as being involved in regulating the intestinal immune response during chronic colitis [2492, 2762]. Protective roles of the C3a receptor were reported for traumatic spinal cord injury [273], melanoma [1840] and systemic lupus erythematosus [1268]. C3a-C3a receptor signalling inhibits neural progenitor cell proliferation during neurodevelopment, playing a critical role in the normal development of the mammalian brain [408]. Inactivation of C3a receptor leads to decreased cytotoxic NK-cell infiltration into tumors [1854]. Moreover, a protective role for C3a receptor is described in experimental chronic pyelonephritis [2944].

**Table:** 

Nomenclature	C5a_1_ receptor	C5a_2_ receptor
HGNC, UniProt	*C5AR1*, P21730	*C5AR2*, Q9P296
Potency order of endogenous ligands	C5a (*C5*, P01031), C5a des-Arg (*C5*) > C3a (*C3*, P01024) [51]	–
Endogenous agonists	C5a des-Arg (*C5*) (Partial agonist) [334], ribosomal protein S19 (*RPS19*, P39019) [2852]	C5a (*C5*, P01031) [334], C5a des-Arg (*C5*) [334, 1947]
Agonists	BM221 [848], NDT9513727 (Inverse agonist) [290], N-methyl-Phe-Lys-Pro-D-Cha-Cha-D-Arg-CO_2_H [1229, 1336], BM213 [848], lactomedin 1 [1980, 2898]	C5a^pep^ (Partial agonist) [1980]
Selective agonists	–	P59 (Biased agonist) [506], P32 (Biased agonist) [506]
Antagonists	avacopan (pIC_50_ 9.7) [167], W54011 (p*K*_i_ 8.7) [2485], DF2593A (pIC_50_ 8.3) [1799], ACT-1014-6470 (pIC_50_ 8) [926], AcPhe-Orn-Pro-D-Cha-Trp-Arg (pIC_50_ 7.9) [2803], PMX205 (pIC_50_ 7.5) [1505, 1650], DF3016A (pIC_50_ 7.3) [269], *N*-methyl-Phe-Lys-Pro-D-Cha-Trp-D-Arg-CO2H (pIC_50_ 7.2) [1336]	A8^Δ71-3^ (pIC_50_ ∼6) [1964] – Mouse
Labelled ligands	[^125^I]C5a (human) (Agonist) [1082], Eu-DTPA-[Ser^27^, Nle^70^]hC5a (Agonist) [849]	Eu-DTPA-[Ser^27^, Nle^70^]hC5a (Agonist) [847], [^125^I]C5a (human) (Agonist)
Comments	The C5a_1_ receptor is currently referred to as C5aR1 in the literature. C5a_1_ has been an attractive target for pharmacological inhibition to treat a myriad of inflammatory and neurodegenerative diseases. Several C5a_1_ antagonists have been reported that have progressed to various stages of clinical development [965, 1505, 1775], although none are yet approved for use in humans. The non-peptide C5aR1 inhibitor CCX168 (Avacopan^®^), developed by ChemoCentryx/Amgen, is currently the most clinically advanced C5aR_1_ inhibitor [167]. The drug was approved by the FDA in October 2021, as an adjunctive treatment in adults for severe active ANCA-associated vasculitis (specifically microscopic polyangiitis (MPA) and granulomatosis with polyangiitis (GPA)) in combination with standard therapy including glucocorticoids [1451]. Considering the potential benefits of blocking the C5a-C5a_1_ axis to limit myeloid infiltration and prevent excessive lung inflammation in Coronavirus disease 2019 (COVID-19) [357], the two anti-C5a/C5a_1_ blocking antibodies, avdoralimab (IPH5401) and vilobelimab (IFX-1), were studied in patients with COVID-19 severe pneumonia (NCT04371367 and NCT04333420) [2691].	The C5a_2_ receptor is commonly referred to as C5L2 and C5aR2 in the literature. C5a_2_ was traditionally recognized as a decoy receptor for C5a, as it has no reported G protein signalling capacity. New research however, shows C5a_2_ is capable of mediating its own set of signalling events and immunomodulatory actions, not only towards C5a_1_ but also other complement, chemokine and pattern recognition receptors [1502].

**Comments**: SB290157 has also been reported to have agonist properties at the C3a receptor [1503, 1670]. The chemoattractant receptor C5a_2_ (also known as GPR77, C5L2) binds C5a and has putative roles in either opposing or promoting inflammatory responses [334, 764, 785, 1504, 1981]. Binding to this site may be displaced with the rank order C5a des-Arg (*C5*) > C5a (*C5*, P01031) [334, 1947] while there is controversy over the ability of C3a (*C3*, P01024) and C3a des Arg (*C3*, P01024) to compete [1047, 1201, 1202, 1947]. C5a_2_ appears to lack G protein signalling and has been termed a decoy receptor [2320]. However, C5a_2_ does recruit β-arrestin 2 after ligand binding, which might provide a signalling pathway for this receptor [125, 2656], and forms heteromers with C5a_1_. A recent study has identified p90RSK (90 kDa ribosomal s6 kinase) phosphorylation as a potential signalling pathway for C5a_2_ [1979]. C5a, but not C5a-des Arg, induces upregulation of heterodimer formation between complement C5a receptors C5a_1_ and C5a_2_ [505]. There are also reports of pro-inflammatory activity of C5a_2_, mediated by HMGB1, likely through AKT and MAPK signalling pathways (reviewed in [1498, 2935]). In T cells it has been shown that C5a_1_ and C5a_2_ act in opposition to each other and that altering the equilibrium between the two receptors, by differential expression or production of C5a-des Arg (which favours C5a_2_), can affect the final cellular response [71]. In human macrophages, C5a_2_ was observed to modulate multiple complement and chemokine receptor-mediated signalling and pattern recognition-induced cytokine responses, independent of C5a_1_ [1502]. In addition, C5a_2_ is reported to act as a C5a transporter on endothelial cells, and is required for the transport of C5a into the vessel lumen and the subsequent neutrophil arrest in arthritis [1754].

Further reading on Complement peptide receptorsArboreG
 (2016) A novel “complement-metabolism-inflammasome axis” as a key regulator of immune cell effector function. Eur J Immunol
46: 1563–7327184294
10.1002/eji.201546131PMC5025719CoulthardLG
 (2015) Is the complement activation product C3a a proinflammatory molecule? Re-evaluating the evidence and the myth. J Immunol
194: 3542–825848071
10.4049/jimmunol.1403068LaumonnierY
 (2017) Novel insights into the expression pattern of anaphylatoxin receptors in mice and men. Mol Immunol
89: 44–5828600003
10.1016/j.molimm.2017.05.019

## Corticotropin-releasing factor receptors

G protein-coupled receptors → Corticotropin-releasing factor receptors

**Overview**: Corticotropin-releasing factor (CRF, **nomenclature as agreed by the NC-IUPHAR subcommittee on Corticotropin-releasing Factor Receptors** [964]) receptors are activated by the endogenous peptides corticotrophin-releasing hormone (*CRH*, P06850), a 41 amino-acid peptide, urocortin 1 (*UCN*, P55089), 40 amino-acids, urocortin 2 (*UCN2*, Q96RP3), 38 amino-acids and urocortin 3 (*UCN3*, Q969E3), 38 amino-acids. CRF_1_ and CRF_2_ receptors are activated non-selectively by CRH and UCN. CRF_2_ receptors are selectively activated by UCN2 and UCN3. Binding to CRF receptors can be conducted using radioligands [^125^I]Tyr^0^-CRF or [^125^I]Tyr^0^-sauvagine with *K*_d_ values of 0.1–0.4 nM. CRF_1_ and CRF_2_ receptors are non-selectively antagonized by α-helical CRF, D-Phe-CRF-(12-41) and astressin. CRF_1_ receptors are selectively antagonized by small molecules NBI27914, R121919, antalarmin, CP 154,526, CP 376,395. CRF_2_ receptors are selectively antagonized by antisauvagine and astressin 2B.

**Table:** 

Nomenclature	CRF_1_ receptor	CRF_2_ receptor
HGNC, UniProt	*CRHR1*, P34998	*CRHR2*, Q13324
Endogenous agonists	urocortin 1 (*UCN*, P55089) [528, 530, 597], corticotrophin-releasing hormone (*CRH*, P06850) [407, 527, 530, 597, 1975, 2678]	urocortin 2 (*UCN2*, Q96RP3) [528], urocortin 3 (*UCN3*, Q969E3) [528]
Antagonists	SSR125543A (pK_i_ 8.7) [896], astressin (pK_i_ 8.7) [2189]	astressin (pIC_50_ 9.2) [2187]
Selective antagonists	CP 154,526 (pIC_50_ 9.3–10.4) [1579] – Rat, DMP696 (pK_i_ 8.3–9) [976], NBI27914 (pK_i_ 8.3–9) [397], R121919 (pK_i_ 8.3–9) [2961], antalarmin (pK_i_ 8.3–9) [2756], NBI-35965 (pK_i_ 8.4) [1744] – Rat, CP 376,395 (pIC_50_ 8.3) [415] – Rat, CRA1000 (pIC_50_ 6.4–7.1) [378]	antisauvagine (pK_d_ 8.8–9.6) [530], K41498 (pK_i_ 9.2) [1423], astressin 2B (pIC_50_ 8.9) [2187], K31440 (pK_i_ 8.7–8.8) [2226]

**Comments**: A CRF binding protein has been identified (*CRHBP*, P24387) to which both corticotrophin-releasing hormone (*CRH*, P06850) and urocortin 1 (*UCN*, P55089) bind with high affinities, which has been suggested to bind and inactivate circulating corticotrophin-releasing hormone (*CRH*, P06850) [537, 2023].

Further reading on Corticotropin-releasing factor receptorsDeussingJM
 (2018) The Corticotropin-Releasing Factor Family: Physiology of the Stress Response. Physiol Rev
98: 2225–228630109816
10.1152/physrev.00042.2017GrammatopoulosDK. (2012) Insights into mechanisms of corticotropin-releasing hormone receptor signal transduction. Br J Pharmacol
166: 85–9721883143
10.1111/j.1476-5381.2011.01631.xPMC3415640HaugerRL
 (2003) International Union of Pharmacology. XXXVI. Current status of the nomenclature for receptors for corticotropin-releasing factor and their ligands. Pharmacol Rev
55: 21–612615952
10.1124/pr.55.1.3LiapakisG
 (2011) Members of CRF family and their receptors: from past to future. Curr Med Chem
18: 2583–60021568890
10.2174/092986711795933704SlaterPG
 (2016) Corticotropin-Releasing Factor Receptors and Their Interacting Proteins: Functional Consequences. Mol Pharmacol
90: 627–63227612874
10.1124/mol.116.104927ZelenayV
 (2017) Structures of the First Extracellular Domain of CRF Receptors. Curr Mol Pharmacol
10: 318–32428103782
10.2174/1874467210666170110120301

## Dopamine receptors

G protein-coupled receptors → Dopamine receptors

**Overview**: Dopamine receptors (**nomenclature as agreed by the NC-IUPHAR Subcommittee on Dopamine Receptors** [2314]) are commonly divided into D_1_-like (D_1_ and D_5_) and D_2_-like (D_2_, D_3_ and D_4_) families, where the endogenous agonist is dopamine.

**Table:** 

Nomenclature	D_1_ receptor	D_2_ receptor
HGNC, UniProt	*DRD1*, P21728	*DRD2*, P14416
Endogenous agonists	dopamine [2491, 2582]	dopamine [320, 733, 2278]
Agonists	fenoldopam [2582]	rotigotine [585], cabergoline (Partial agonist) [1735], aripiprazole (Partial agonist) [2911], bromocriptine [733, 1735, 2278], MLS1547 (Biased agonist) [732], ropinirole [984], apomorphine (Partial agonist) [320, 733, 1735, 2278, 2427], pramipexole [1729, 2278], benzquinamide [871]
Sub/family-selective agonists	A68930 [1879], SKF-38393 (Partial agonist) [2491, 2582]	quinpirole [320, 1729, 1996, 2427, 2429, 2660]
Selective agonists	SKF-83959 (Biased agonist) [484], A77636 [2239], SKF-81297 [59] – Rat	sumanirole [1689]
Antagonists	flupentixol (p*K*_i_ 7–8.4) [2491, 2582]	blonanserin (p*K*_i_ 9.9) [1925], pipotiazine (p*K*_i_ 9.7) [2428], perphenazine (p*K*_i_ 8.9–9.6) [1362, 2330], risperidone (p*K*_i_ 9.4) [80], perospirone (p*K*_i_ 9.2) [2331], trifluoperazine (p*K*_i_ 8.9–9) [1362, 2332], quetiapine (p*K*_i_ 7.2) [80]
Sub/family-selective antagonists	SCH-23390 (p*K*_i_ 7.4–9.5) [2491, 2582], SKF-83566 (p*K*_i_ 9.5) [2491], ecopipam (p*K*_i_ 8.3) [2583]	haloperidol (p*K*_i_ 7.4–8.8) [733, 1601, 1729, 2427, 2583]
Selective antagonists	–	L-741,626 (p*K*_i_ 7.9–8.5) [885, 1376], domperidone (p*K*_i_ 7.9–8.4) [733, 2427], raclopride (p*K*_i_ 8) [1737], ML321 (pK_i_ 7) [2830, 2831]
Sub/family-selective labelled ligands	[^125^I]SCH23982 (Antagonist) (p*K*_d_ 9.5) [557], [3H]SCH-23390 (Antagonist) (p*K*_d_ 9.5) [2949]	[^3^H]spiperone (Antagonist) (pKd 10.2) [315, 1030, 2947] – Rat
Labelled ligands	–	[^3^H]raclopride (Antagonist) (p*K*_d_ 8.9) [1324] – Rat

**Table:** 

Nomenclature	D_3_ receptor	D_4_ receptor	D_5_ receptor
HGNC, UniProt	*DRD3*, P35462	*DRD4*, P21917	*DRD5*, P21918
Endogenous agonists	dopamine [320, 733, 2278, 2429]	dopamine [2660]	dopamine [2491]
Agonists	cariprazine (Partial agonist) [1295], pramipexole [1729, 2278], bromocriptine (Partial agonist) [733, 1735, 2278], ropinirole [984], apomorphine (Partial agonist) [320, 733, 1735, 2278, 2427]	apomorphine (Partial agonist) [1735]	–
Sub/family-selective agonists	quinpirole [320, 1729, 1737, 1996, 2278, 2427, 2429, 2660]	quinpirole [1735, 1996, 2660]	A68930 [1879]
Selective agonists	PD 128907 [2098, 2278]	PD168,077 (Partial agonist) [1341] – Rat, A412997 [1788] – Rat, A412997 [1788]	–
Antagonists	perospirone (pKi 9.6) [2427], sertindole (pKi 8–8.8) [80, 2307, 2330], prochlorperazine (pKi 8.4) [93], (−)-sulpiride (pKi 6.7–7.7) [733, 2427, 2542], loxapine (pKi 7.7) [2330], domperidone (pKi 7.1–7.6) [733, 2427], promazine (pKi 6.8) [321]	perospirone (pKi 10.1) [2333], sertindole (pKi 7.8–9.1) [321, 2330, 2332, 2333], sonepiprazole (pKi 8.9) [2297], loxapine (pKi 8.1) [2332]	–
Sub/family-selective antagonists	haloperidol (p*K*_i_ 7.5–8.6) [733, 2350, 2427, 2583]	haloperidol (p*K*_i_ 8.7–8.8) [1397, 2350, 2583]	SCH-23390 (p*K*_i_ 7.5–9.5) [2491], SKF-83566 (p*K*_i_ 9.4) [2491], ecopipam (p*K*_i_ 8.3) [2491]
Selective antagonists	S33084 (p*K*_i_ 9.6) [1734], nafadotride (p*K*_i_ 9.5) [2279], PG01037 (p*K*_i_ 9.2) [886], NGB 2904 (p*K*_i_ 8.8) [2827], SB 277011-A (p*K*_i_ 8) [2156], (+)-S-14297 (p*K*_i_ 6.9–7.9) [1732, 1737]	L745870 (p*K*_i_ 9.4) [1376], A-381393 (p*K*_i_ 8.8) [1850], L741742 (p*K*_i_ 8.5) [2221], ML398 (p*K*_i_ 7.4) [188]	–
Selective allosteric modulators	SB269652 (Negative) (p*K*_i_ ~9) [750]	–	–
Sub/family-selective labelled ligands	–	[^3^H]spiperone (Antagonist) (pK_d_ 9.5) [1009, 2660]	[^3^H]SCH-23390 (Antagonist) (pK_d_ 9.2) [2172]
Labelled ligands	[^3^H]spiperone (Antagonist) (p*K*_d_ 9.9) [1030, 2947] – Rat, [³H]7-OH-DPAT (Agonist) [2173], [³H] PD128907 (Agonist) [33]	[^125^I]L750667 (Antagonist) (p*K*_d_ 9.8) [1996], [³H] NGD941 (Antagonist) (p*K*_d_ 8.3) [2084]	[^125^I]SCH23982 (Antagonist) (p*K*_d_ 9.1)

**Comments**: The selectivity of many of these agents is less than two orders of magnitude. [^3^H]raclopride exhibits similar high affinity for D_2_ and D_3_ receptors (low affinity for D_4_), but has been used to label D_2_ receptors in the presence of a D_3_-selective antagonist. [^3^H]7-OH-DPAT has similar affinity for D_2_ and D_3 _ receptors, but labels only D_3_ receptors in the absence of divalent cations. The pharmacological profile of the D_5_ receptor is similar to, yet distinct from, that of the D_1_ receptor. The splice variants of the D_2_ receptor are commonly termed D_2S_ and D_2L_ (short and long). The *DRD4* gene encoding the D_4_ receptor is highly polymorphic in humans, with allelic variations of the protein from amino acid 387 to 515.

Further reading on Dopamine receptorsBeaulieuJM
 (2015) Dopamine receptors - IUPHAR Review 13. Br J Pharmacol
172: 1–2325671228
10.1111/bph.12906PMC4280963BeaulieuJM
 (2011) The physiology, signaling, and pharmacology of dopamine receptors. Pharmacol Rev
63: 182–21721303898
10.1124/pr.110.002642CummingP. (2011) Absolute abundances and affinity states of dopamine receptors in mammalian brain: A review. Synapse
65: 892–90921308799
10.1002/syn.20916MoritzAE
 (2018) Advances and challenges in the search for D_2_ and D_3_ dopamine receptor-selective compounds. Cell Signal
41: 75–8128716664
10.1016/j.cellsig.2017.07.003PMC5722689UrsNM
 (2017) New Concepts in Dopamine D_2_ Receptor Biased Signaling and Implications for Schizophrenia Therapy. Biol Psychiatry
81: 78–8527832841
10.1016/j.biopsych.2016.10.011PMC5702557ZhuangY
 (2021) Structural insights into the human D1 and D2 dopamine receptor signaling complexes. Cell
184: 931–942.e1833571431
10.1016/j.cell.2021.01.027PMC8215686

## Endothelin receptors

G protein-coupled receptors → Endothelin receptors

**Overview**: Endothelin receptors (**nomenclature as agreed by the NC-IUPHAR Subcommittee on Endothelin Receptors** [531]) are activated by the endogenous 21 amino-acid peptides endothelins 1–3 (endothelin-1 (*EDN1*, P05305), endothelin-2 (*EDN2*, P20800) and endothelin-3 (*EDN3*, P14138)).

**Table:** 

Nomenclature	ET_A_ receptor	ET_B_ receptor
HGNC, UniProt	*EDNRA*,P25101	*EDNRB*,P24530
Potency order of endogenous ligands	endothelin-1 (*EDN1*,P05305) = endothelin-2 (*EDN2*, P20800) > endothelin-3 (*EDN3*, P14138) [1614]	endothelin-1 (*EDN1*, P05305) = endothelin-2 (*EDN2*, P20800), endothelin-3 (*EDN3*, P14138) [2251]
Selective agonists	–	sarafotoxin S6c [1367, 2230], BQ 3020 [2168], [Ala^1,3,11,15^]ET-1 [1763], sovateltide [2137, 2746]
Antagonists	SB209670 (pKB 9.4) [647] – Rat, TAK 044 (pA2 8.4) [2749] – Rat, bosentan (pA2 7.2) [471] – Rat, aprocitentan (pA2 6.7) [1094]	SB209670 (pKB 9.4) [647] – Rat, TAK 044 (pA2 8.4) [2749] – Rat, bosentan (pKi 7.1) [471], aprocitentan (pA2 5.5) [1094]
Selective antagonists	clazosentan (pA_2_ 9.5) [2218], macitentan (pIC_50_ 9.3) [229], atrasentan (pA_2_ 9.2) [1959], zibotentan (pIC_50_ 8.3) [1810], sitaxsentan (pA_2_ 8) [2815], FR139317 (Inverse agonist) (pIC_50_ 7.3–7.9) [1614], BQ123 (pA_2_ 6.9–7.4) [1614], ambrisentan (pA_2_ 7.1) [230]	K-8794 (pIC_50_ 8.2) [2364], A192621 (pKd 8.1) [2695], BQ788 (pKd 7.9–8) [2230], IRL 2500 (pKd 7.2) [2230], Ro 46-8443 (pIC50 7.2) [276]
Labelled ligands	[^125^I]PD164333 (Antagonist) (pKd 9.6–9.8) [534], [^3^H]S0139 (Antagonist) (pKd 9.2) [1731], [^125^I]PD151242 (Antagonist) (pKd 9–9.1) [535], [^3^H]BQ123 (Antagonist) (pKd 8.5) [1100]	[^125^I]IRL1620 (Agonist) [1851], [^125^I]BQ3020 (Agonist) [948, 1763, 2030], [^125^I][Ala^1,3,11,15^]ET-1 (Agonist) [1763]

**Comments**: Splice variants of the ET_A_ receptor have been identified in rat pituitary cells; one of these, ET_A_R-C13, appeared to show loss of function with comparable plasma membrane expression to wild type receptor [960]. Subtypes of the ET_B_ receptor have been proposed, although gene disruption studies in mice suggest that only a single gene product exists [1756]. Cryogenic-electron microscopy structures of ET_A_ and ET_B_ bound to endothelin-1 (*EDN1*, P05305) and ET_B_ bound to sovateltide (IRL1620) [1166] and crystal structures of the ET_B_ receptor complexed with non-selective agonists endothelin-1 (*EDN1*, P05305) [2363] and sarafotoxin S6b [1128], ET_B_ selective agonists endothelin-3 (*EDN3*, P14138) and sovateltide (IRL1620) [2362], inverse agonist IRL 2500 [1844], and clinically relevant non-selective antagonist bosentan and the ET_B_ selective analogue K-8794 [2364] have been reported. Sparsentan is a combined ET_A_ and AT_1_ receptor antagonist [1348].

Further reading on Endothelin receptorsClozelM
 (2013) Endothelin receptor antagonists. Handb Exp Pharmacol
218: 199–22724092342
10.1007/978-3-642-38664-0_9DavenportAP. (2002) International Union of Pharmacology. XXIX. Update on endothelin receptor nomenclature. Pharmacol Rev
54: 219–2612037137
10.1124/pr.54.2.219DavenportAP
 (2016) Endothelin. Pharmacol Rev
68: 357–41826956245
10.1124/pr.115.011833PMC4815360DavenportAP
 (2018) New drugs and emerging therapeutic targets in the endothelin signaling pathway and prospects for personalized precision medicine. Physiol Res
67: S37–S5429947527
10.33549/physiolres.933872DhaunN
 (2019) Endothelins in cardiovascular biology and therapeutics. Nat Rev Cardiol
16: 491–50230867577
10.1038/s41569-019-0176-3KoCJ
 (2022) Endothelin 2: a key player in ovulation and fertility. Reproduction
163: R71–R8035167488
10.1530/REP-21-0313PMC8941721MaguireJJ
 (2014) Endothelin@25 - new agonists, antagonists, inhibitors and emerging research frontiers: IUPHAR Review 12. Br J Pharmacol
171: 5555–7225131455
10.1111/bph.12874PMC4290702TocciP
 (2021) YAP and endothelin-1 signaling: an emerging alliance in cancer. J Exp Clin Cancer Res
40: 2733422090
10.1186/s13046-021-01827-8PMC7797087

## G protein-coupled estrogen receptor

G protein-coupled receptors → G protein-coupled estrogen receptor

**Overview**: The G protein-coupled estrogen receptor (GPER, **nomenclature as agreed by the NC-IUPHAR Subcommittee on the G protein-coupled estrogen receptor** [2091]) was identified following observations of estrogen-evoked cyclic AMP signalling in breast cancer cells [81], which mirrored the differential expression of an orphan 7-transmembrane receptor GPR30 [348]. There are observations of both cell-surface and intracellular expression of the GPER receptor [2163, 2573]. Selective agonist/antagonists for GPER have been characterized [2091]. Antagonists of the nuclear estrogen receptor, such as fulvestrant [696], tamoxifen [2163, 2573] and raloxifene [2039], as well as the flavonoid’phytoestrogens’ genistein and quercetin [1613], are agonists of GPER. Reviews of GPER pharmacology have been published [2091]. The roles of GPER in (patho)physiological systems throughout the body (cardiovascular, metabolic, endocrine, immune, reproductive) and in cancer have also been reviewed [695, 1415, 1714, 2091, 2092]. The GPER-selective agonist G-1 is currently in Phase I/II clinical trials for cancer (NCT04130516).

**Table:** 

Nomenclature	GPER
HGNC, UniProt	*GPER1*, Q99527
Endogenous agonists	17*β*-estradiol [2163, 2573]
Agonists	fulvestrant [2573], raloxifene [2039], 4-hydroxytamoxifen [2163]
Selective agonists	G-1 [232]
Selective antagonists	G36 (pIC_50_ 6.8–6.9) [566], G15 (pIC_50_ 6.7) [565]
Labelled ligands	[^3^H]17*β*-estradiol (Agonist) [2573]

Further reading on G protein-coupled estrogen receptorArterburnJB
 (2023) G Protein-Coupled Estrogen Receptor GPER: Molecular Pharmacology and Therapeutic Applications. Annu Rev Pharmacol Toxicol
63: 295–32036662583
10.1146/annurev-pharmtox-031122-121944PMC10153636BartonM
 (2018) Twenty years of the G protein-coupled estrogen receptor GPER: Historical and personal perspectives. J Steroid Biochem Mol Biol
176: 4–1528347854
10.1016/j.jsbmb.2017.03.021PMC5716468GaudetHM
 (2015) The G-protein coupled estrogen receptor, GPER: The inside and inside-out story. Mol Cell Endocrinol
418 Pt 3: 207–1926190834
10.1016/j.mce.2015.07.016ProssnitzER
 (2015) International Union of Basic and Clinical Pharmacology. XCVII. G Protein-Coupled Estrogen Receptor and Its Pharmacologic Modulators. Pharmacol Rev
67: 505–4026023144
10.1124/pr.114.009712PMC4485017ProssnitzER
 (2023) The G-protein-coupled estrogen receptor GPER in health and disease: an update. Nat Rev Endocrinol10.1038/s41574-023-00822-7PMC1018752537193881ProssnitzER
 (2015) What have we learned about GPER function in physiology and disease from knockout mice?
J Steroid Biochem Mol Biol
153: 114–2626189910
10.1016/j.jsbmb.2015.06.014PMC4568147

## Formylpeptide receptors

G protein-coupled receptors → Formylpeptide receptors

**Overview**: The formylpeptide receptors (**nomenclature agreed by the NC-IUPHAR Subcommittee on the formylpeptide receptor family** [2882]) respond to exogenous ligands such as the bacterial product fMet-Leu-Phe (fMLP) and endogenous ligands such as lipoxin A_4_ (LXA_4_), 15-*epi*-lipoxin A_4_, annexin I (*ANXA1*, P04083) , cathepsin G (*CTSG*, P08311), amyloid *β*42, serum amyloid A and spinorphin, derived from *β*-haemoglobin (*HBB*, P68871). FPR1 also serves as a plague receptor for selective destruction of human immune cells by *Y. pestis* [1962]. The FPR1/2 agonists’compound 17b’ and ’compound 43’ have shown cardiac protective functions [768, 2108].

**Table:** 

Nomenclature	FPR1	FPR2/ALX	FPR3
HGNC, UniProt	*FPR1*, P21462	*FPR2*, P25090	*FPR3*, P25089
Potency order of endogenous and other ligands	–	LXA_4_ = aspirin triggered lipoxin A4 = ATLa2 = resolvin D1 > LTC_4_ = LTD_4_ ≫ 15-deoxy-LXA4 ≫ fMet-Leu-Phe [469, 700, 702, 879, 2520]	–
Potency order of endogenous ligands	fMet-Leu-Phe > cathepsin G (*CTSG*, P08311) > annexin I (*ANXA1*, P04083) [1437, 2488]	–	–
Endogenous agonists	–	LXA_4_ [1359], resolvin D1 [1359], aspirin-triggered resolvin D1 [1358], aspirin triggered lipoxin A4	F2L (*HEBP1*, Q9NRV9) [1730]
Agonists	fMet-Leu-Phe [735, 2378]	–	–
Selective agonists	–	ATLa2 [895]	–
Endogenous antagonists	spinorphin (pIC_50_ 4.3) [1509, 1835]	–	–
Antagonists	t-Boc-FLFLF (pK_i_ 6–6.5) [2767]	–	–
Selective antagonists	cyclosporin H (pK_i_ 6.1–7.1) [2767, 2860]	WRWWWW (pIC_50_ 6.6) [111], t-Boc-FLFLF (pIC_50_ 4.3–6) [734, 2454, 2714]	–
Labelled ligands	[^3^H]fMet-Leu-Phe (Agonist) [1337]	[^3^H]LXA_4_ (Agonist) [700, 701]	–
Comments	A FITC-conjugated fMLP analogue has been used for binding to the mouse recombinant receptor [974].	–	–

**Comments**: Note that the data for FPR2/ALX are also reproduced on the leukotriene receptor page.

FPR1 has been reported to be the plague receptor on host immune cells [1962]. By interacting with LcrV, the needle cap protein of the type III secretion system of *Y. pestis*, FPR1 serves to promote translocation of virulent factors of the bacteria. The R190W mutation of FPR1 confers resistance to this function of *Y. pestis*. Several FPR1/2 agonists including’compound 17b’ and’compound 43’ have been shown to display cardiac protective functions in mouse models of myocardial ischemia-reperfusion injury [768, 2108]. Studies have been conducted to explore the mechanisms by which FPR2 mediates both inflammatory and anti-inflammatory signaling in a ligand-dependent manner. The status of FPR2 dimerization is a determining factor for ligand-specific conformational changes leading to biased signaling [486]. There is also a report on ligand concentration-dependent dual modulation of FPR2 by lipoxin A_4_ for receptor-activation *vs*. anti-inflammatory activities [788]. Some FPR2 ligands may display allosteric modulatory effects that cause changes in FPR2 conformational states and receptor signaling [2928]. The 3-D structure of FPR2 has been solved by the use of cryo-electron microscopy [2958] and receptor protein crystallization [409]. The FPR2 structure reveals a large binding pocket that can accommodate several ligands of different shapes and sizes.

Further reading on Formylpeptide receptorsDahlgrenC
 (2016) Basic characteristics of the neutrophil receptors that recognize formylated peptides, a danger-associated molecular pattern generated by bacteria and mitochondria. Biochem Pharmacol
114: 22–3927131862
10.1016/j.bcp.2016.04.014DorwardDA
 (2015) The Role of Formylated Peptides and Formyl Peptide Receptor 1 in Governing Neutrophil Function during Acute Inflammation. Am J Pathol
185: 1172–118425791526
10.1016/j.ajpath.2015.01.020PMC4419282KrepelSA
 (2019) Chemotactic Ligands that Activate G-Protein-Coupled Formylpeptide Receptors. Int J Mol Sci
20:10.3390/ijms20143426PMC667834631336833PerrettiM
 (2020) Formyl peptide receptor type 2 agonists to kick-start resolution pharmacology. Br J Pharmacol
177: 4595–460032954491
10.1111/bph.15212PMC7520433YazidS
 (2012) Anti-inflammatory drugs, eicosanoids and the annexin A1/FPR2 anti-inflammatory system. Prostaglandins Other Lipid Mediat
98: 94–10022123264
10.1016/j.prostaglandins.2011.11.005YeRD
 (2009) International Union of Basic and Clinical Pharmacology. LXXIII. Nomenclature for the formyl peptide receptor (FPR) family. Pharmacol Rev
61: 119–6119498085
10.1124/pr.109.001578PMC2745437

## Free fatty acid receptors

G protein-coupled receptors → Free fatty acid receptors

**Overview**: Free fatty acid receptors (FFA, **nomenclature as agreed by the NC-IUPHAR Subcommittee on free fatty acid receptors** [532, 2464]) are activated by free fatty acids. Long-chain saturated and unsaturated fatty acids (including C14.0 (myristic acid), C16:0 (palmitic acid), C18:1 (oleic acid), C18:2 (linoleic acid), C18:3, (*β*-linolenic acid), C20:4 (arachidonic acid), C20:5, n-3 (EPA) and C22:6, n-3 (docosahexaenoic acid)) activate FFA1 [284, 1121, 1344] and FFA4 receptors [1019, 1092, 1931], while short chain fatty acids (C2 (acetic acid), C3 (propanoic acid), C4 (butyric acid) and C5 (pentanoic acid)) activate FFA2 [295, 1436, 1900] and FFA3 [295, 1436] receptors. The crystal structure for agonist bound FFA1 has been described [2446].

**Table:** 

Nomenclature	FFA1 receptor	FFA2 receptor
HGNC, UniProt	*FFAR1*, O14842	*FFAR2*, O15552
Endogenous agonists	docosahexaenoic acid [284, 1121], α-linolenic acid [284, 1121, 1344], oleic acid [284, 1121, 1344], myristic acid [284, 1121, 1344]	propanoic acid [295, 1436, 1900, 2299], acetic acid [295, 1436, 1900, 2299], butyric acid [295, 1436, 1900, 2299], trans-2-methylcrotonic acid [2299], 1-methylcyclopropanecarboxylic acid [2299]
Agonists	HWL-088 [412]	–
Selective agonists	AMG-837 [1522], compound 4 [448], TUG-770 [447], TUG-905 [446], GW9508 (Partial agonist) [283], fasiglifam [1200, 1869, 2446, 2615]	TUG-1375 [937]
Selective antagonists	GW1100 (pIC_50_ 6) [283, 2463]	GLPG0974 (pIC_50_ 8.1) [1853, 2058], CATPB (pIC_50_ 6.5) [1080]
Comments	A wide range of both saturated and unsaturated fatty acids containing from 6 to 22 carbons have been shown to act as agonists at FFA1 [284, 1121, 1344]. Antagonist GW1100 is also an oxytocin receptor antagonist [283]. Fasiglifam, TUG-770 and GW9508 are approximately 100 fold selective for FFA1 over FFA4 [283, 447, 1869]. AMG-837 and the related analogue AM6331 have been suggested to have an allosteric mechanism of action at FFA1, with respect to the orthosteric fatty acid binding site [1522, 2838].	–

**Table:** 

Nomenclature	FFA3 receptor	FFA4 receptor
HGNC, UniProt	*FFAR3*, O14843	*FFAR4*, Q5NUL3
Endogenous agonists	propanoic acid [295, 1436, 2299, 2837], butyric acid [295, 1436, 2299, 2837], 1-methylcyclopropanecarboxylic acid [2299]	α-linolenic acid [2369], myristic acid [2753], α-linolenic acid [2538] – Rat, oleic acid [2753]
Agonists	acetic acid [295, 1436, 2299, 2837]	–
Selective agonists	–	compound A [1930], TUG-891 [2369], NCG21 [2499]
Comments	Beta-hydroxybutyrate has been reported to antagonise FFA3 responses to short chain fatty acids [1284]. A range of FFA3 selective molecules with agonist and antagonist properties, but which bind at sites distinct from the short chain fatty acid binding site (i.e. allosteric modulators), have been described [233, 1079, 1591].	A wide range of both saturated and unsaturated fatty acids containing from 6 to 22 carbons have been shown to act as agonists at FFA4 [449] with a small subset listed above. Compound A [PMID 24997608] exhibits more than 1000 fold selectivity [1930], and TUG-891 50–1000 fold selectivity for FFA4 over FFA1 [2369], dependent on the assay. NGC21 exhibits approximately 15 fold selectivity for FFA4 over FFA1 [2487].

**Comments**: Short (361 amino acids) and long (377 amino acids) splice variants of human FFA4 have been reported [1787], which differ by a 16 amino acid insertion in intracellular loop 3, and exhibit differences in intracellular signalling properties in recombinant systems [2753]. The long FFA4 splice variant has not been identified in other primates or rodents to date [1019, 1787].

*GPR42* was originally described as a pseudogene within the family (ENSFM00250000002583), but the discovery of several polymorphisms suggests that some versions of GPR42 may be functional [1510]. *GPR84* is a structurally-unrelated G protein-coupled receptor which has been found to respond to medium chain fatty acids [2727].

Further reading on Free fatty acid receptorsBologniniD
 (2016) The Pharmacology and Function of Receptors for Short-Chain Fatty Acids. Mol Pharmacol
89: 388–9826719580
10.1124/mol.115.102301ManciniAD
 (2013) The fatty acid receptor FFA1/GPR40 a decade later: how much do we know?
Trends Endocrinol Metab
24: 398–40723631851
10.1016/j.tem.2013.03.003MilliganG
 (2017) Complex Pharmacology of Free Fatty Acid Receptors. Chem Rev
117: 67–11027299848
10.1021/acs.chemrev.6b00056MoniriNH. (2016) Free-fatty acid receptor-4 (GPR120): Cellular and molecular function and its role in metabolic disorders. Biochem Pharmacol
110-111: 1–1526827942
10.1016/j.bcp.2016.01.021PMC6415295StoddartLA
 (2008) International Union of Pharmacology. LXXI. Free fatty acid receptors FFA1, -2, and -3: pharmacology and pathophysiological functions. Pharmacol Rev
60: 405–1719047536
10.1124/pr.108.00802WattersonKR
 (2014) Treatment of type 2 diabetes by free Fatty Acid receptor agonists. Front Endocrinol (Lausanne)
5: 13725221541
10.3389/fendo.2014.00137PMC4147718

## GABA_B_ receptors B

G protein-coupled receptors → GABA_B_ receptors

**Overview**: Functional GABA_B_ receptors (**nomenclature as agreed by the NC-IUPHAR Subcommittee on GABA_B_ receptors** [255, 2051]) are formed from the heterodimeriza tion of two similar 7TM subunits termed GABA_B1_ and GABA_B2_ [255, 651, 2050, 2051, 2633]. GABA_B_ receptors are widespread in the CNS and regulate both pre- and postsynaptic activity. The GABA_B1_ subunit, when expressed alone, binds both antagonists and agonists, but the affinity of the latter is generally 10–100-fold less than for the native receptor. Co-expression of GABA_B1_ and GABA_B2_ subunits allows transport of GABA_B1_ to the cell surface and generates a functional receptor that can couple to signal transduction pathways such as high-voltage-activated Ca^2+^ channels (Ca_v_2.1, Ca_v_2.2), or inwardly rectifying potassium channels (Kir3) [193, 255, 256]. The GABA_B1_ subunit harbours the GABA (orthosteric)-binding site within an extracellular domain (ECD) venus flytrap module (VTM), whereas the GABA_B2_ subunit mediates G protein-coupled signalling [255, 794, 796, 2050]. The cryo-electron microscopy structures of the human full-length GABA_B1_-GABA_B2_ heterodimer have been solved in the inactive apo state, two intermediate agonist-bound forms and an active state in which the heterodimer is bound to an agonist and a positive allosteric modulator [2355]. The positive allosteric modulator binds to the transmembrane dimerization interface and stabilizes the active state. Recent evidence indicates that higher order assemblies of GABA_B_ receptor comprising dimers of heterodimers occur in recombinant expression systems and *in vivo* and that such complexes exhibit negative functional cooperativity between heterodimers [479, 2048]. Adding further complexity, KCTD (potassium channel tetramerization proteins) 8, 12, 12b and 16 associate as tetramers with the carboxy terminus of the GABA_B2_ subunit to impart altered signalling kinetics and agonist potency to the receptor complex [142, 2317, 2619] and are reviewed by [2052]. The molecular complexity of GABA_B_ receptors is further increased through association with trafficking and effector proteins [2318] and reviewed by [2047]. The predominant GABA_B1a_ and GABA_B1b_ isoforms, which are most prevalent in neonatal and adult brain tissue respectively, differ in their ECD sequences as a result of the use of alternative transcription initiation sites. GABA_B1a_-containing heterodimers localise to distal axons and mediate inhibition of glutamate release in the CA3-CA1 terminals, and GABA release onto the layer 5 pyramidal neurons, whereas GABA_B1b_containing receptors occur within dendritic spines and mediate slow postsynaptic inhibition [2022, 2683]. Amyloid precursor protein (APP) and soluble APP (sAPP) bind to the N- terminal sushi domain of the GABA_B1a_ isoform to regulate axonal trafficking of GABA_B_ receptors and release of neurotransmitters [2176].

**Table:** Complexes

Nomenclature	GABA_B_ receptor
Subunits	GABA_B1_,GABA_B2_, KCTD8 (Accessory protein), KCTD12 (Accessory protein), kctd12b (Accessory protein), KCTD16 (Accessory protein)
Agonists	CGP 44532 [741] – Rat, (−)-baclofen [741] – Rat, 3-APPA [1023], baclofen [1023, 2807], 3-APMPA [2807]
Antagonists	CGP 62349 (pKi 8.5–8.9) [1023, 2807], CGP 55845 (pKi 7.8) [2807], SCH 50911 (pKi 5.5–6) [1023, 2807], CGP 35348 (pKi 4.4) [2807], 2-hydroxy-saclofen (pIC50 4.1) [1227] – Rat
Allosteric modulators (Positive)	rac-BHFF (pEC_50_ 6.6) [1627], GS39783 (pKB 4.7) [992, 2643], CGP7930 [2642]
Allosteric modulators (Negative)	compound 14 (pIC_50_ 4.4) [404]
Labelled ligands	[^3^H]CGP 54626 (Antagonist) (pKi 9.1) [1185] – Rat, [^3^H]CGP 62349 (Antagonist) (pKd 9.1) [1236] – Rat, [^125^I]CGP 64213 (Antagonist) (pKd 9) [759] – Rat, [^125^I]CGP 71872 (Antagonist) (pKd 9) [1227] – Rat, [^3^H](R)-(−)-baclofen (Agonist)

**Table:** Subunits

Nomenclature	GABA_B1_	GABA_B2_
HGNC, UniProt	*GABBR1*, Q9UBS5	*GABBR2*, O75899

**Comments**: Potencies of agonists and antagonists listed in the table, quantified as IC_50_ values for the inhibition of [^3^H] CGP27492 binding to rat cerebral cortex membranes, are from [255, 740, 741]. Radioligand *K*_D_ values relate to binding to rat brain membranes. CGP 71872 is a photoaffinity ligand for the GABA_B1_ subunit [171]. CGP27492 (3-APPA), CGP35024 (3-APMPA) and CGP 44532 act as antagonists at human GABA_A_ ⍴1 receptors, with potencies in the low micromolar range [740]. In addition to the ligands listed in the table, Ca^2+^ binds to the VTM of the GABA_B1_ subunit to act as a positive allosteric modulator of GABA [759]. Synthetic positive allosteric modulators with low, or no, intrinsic activity include CGP7930, GS39783, BHF-177 [2692] and (+)-BHFF [12, 193, 202, 740]. The site of action of CGP7930 and GS39783 appears to be on the heptahelical domain of the GABA_B2_ subunit [628, 2050]. In the presence of CGP7930 or GS39783, CGP 35348 and 2-hydroxy-saclofen behave as partial agonists [740]. A negative allosteric modulator of GABA_B_ activity has been reported [404]. Knock-out of the GABA_B1_ subunit in C57B mice causes the development of severe tonic-clonic convulsions that prove fatal within a month of birth, whereas GABA_B1_^−/−^ BALB/c mice, although also displaying spontaneous epileptiform activity, are viable. The phenotype of the latter animals additionally includes hyperalgesia, hyperlocomotion (in a novel, but not familiar, environment), hyperdopaminergia, memory impairment and behaviours indicative of anxiety [657, 2645]. A similar phenotype has been found for GABA_B2_^−/−^ BALB/c mice [781].

Further reading on GABAB receptorsBoweryNG
 (2002) International Union of Pharmacology. XXXIII. Mammalian gamma-aminobutyric acid(B) receptors: structure and function. Pharmacol Rev
54: 247–6412037141
10.1124/pr.54.2.247FroestlW. (2011) An historical perspective on GABAergic drugs. Future Med Chem
3: 163–7521428811
10.4155/fmc.10.285GassmannM
 (2012) Regulation of neuronal GABA(B) receptor functions by subunit composition. Nat Rev Neurosci
13: 380–9422595784
10.1038/nrn3249PinJP
 (2016) Organization and functions of mGlu and GABAB receptor complexes. Nature
540: 60–6827905440
10.1038/nature20566

## Galanin receptors

G protein-coupled receptors → Galanin receptors

**Overview**: Galanin receptors (**provisional nomenclature as recommended by NC-IUPHAR** [712]) are activated by the endogenous peptides galanin (*GAL*, P22466) and galanin-like peptide (*GALP*, Q9UBC7). Human galanin (*GAL*, P22466) is a 30 amino-acid non-amidated peptide [673]; in other species, it is 29 amino acids long and C-terminally amidated. Amino acids 1–14 of galanin are highly conserved in mammals, birds, reptiles, amphibia and fish. Shorter peptide species (*e.g*. human galanin-1–19 [189] and porcine galanin-5-29 [2385]) and N-terminally extended forms (*e.g*. N-terminally seven and nine residue elongated forms of porcine galanin [190, 2385]) have been reported. More recently, the newly-identified peptide, spexin (SPX), has been reported to activate human GAL2 and GAL3 (but not GAL1) receptors in heterologous expression systems; and to alter GAL2/3 receptor-related behaviours in animals [1269].

**Table:** 

Nomenclature	GAL_1_ receptor	GAL_2_ receptor	GAL_3_ receptor
HGNC, UniProt	*GALR1*,P47211	*GALR2*,O43603	*GALR3*,O60755
Potency order of endogenous ligands	galanin (*GAL*,P22466) > galanin-like peptide (*GALP*, Q9UBC7) [1938]	galanin-like peptide (*GALP*, Q9UBC7) ≥ galanin (*GAL*, P22466) [1938]	galanin-like peptide (*GALP*, Q9UBC7) > galanin (*GAL*, P22466) [1405]
Endogenous agonists	–	spexin-1 (*SPX*,Q9BT56) [1269]	spexin-1 (*SPX*,Q9BT56) [1269]
Agonists	–	galanin(2-29) (rat/mouse) [1982, 2732, 2733, 2734] – Rat	–
Selective agonists	–	[D-Trp^2^]galanin-(1-29) [2413] – Rat, Qu-SPX [1459]	–
Selective antagonists	2,3-dihydro-1,4-dithiin-1,1,4,4-tetroxide (pIC_50_ 5.6) [2324]	M871 (p*K*_i_ 7.9) [2431]	SNAP 398299 (p*K*_i_ 8.3) [1332, 1333, 2503], SNAP 37889 (p*K*_i_ 7.8–7.8) [1332, 1333, 2503]
Selective allosteric modulators	–	CYM2503 (Positive) (pEC_50_ 9.2) [1571] – Rat	–
Labelled ligands	[^125^I][Tyr^26^]galanin (human) (Agonist) [708], [^125^I] [Tyr^26^]galanin (human) (Agonist) [708]	[^125^I][Tyr^26^]galanin (human) (Agonist) [2733] – Rat, [^125^I]spexin-1 (Agonist) [1269]	[^125^I][Tyr^26^]galanin (pig) (Agonist) [246, 2414], [^125^I] spexin-1 (Agonist) [1269]
Comments	–	The CYM2503 PAM potentiates the anticonvulsant activity of endogenous galanin in mouse seizure models [1571]. Activation and binding potency of spexin at human GAL_2_ receptor is less than galanin (GAL) [1269].	Activation and binding potency of spexin at human GAL_3_ receptor is higher than galanin (GAL) [1269].

**Comments**: Galanin-(1-11) is a high-affinity agonist at GAL_1_/GAL_2_ (p*K*_i_ 9), and galanin(2-11) is selective for GAL_2_ and GAL_3_ compared with GAL_1_ [1570]. [^125^I]-[Tyr^26^]galanin binds to all three subtypes with *K*_d_ values generally reported to range from 0.05 to 1 nM, depending on the assay conditions used [708, 2399, 2413, 2414, 2733]. Porcine galanin-(3-29) does not bind to cloned GAL_1_, GAL_2_ or GAL_3_ receptors, but a receptor that is functionally activated by porcine galanin-(3–29) has been reported in pituitary and gastric smooth muscle cells [887, 2826]. Additional galanin receptor subtypes are also suggested from studies with chimeric peptides (*e.g*. M15, M35 and M40), which act as antagonists in functional assays in the cardiovascular system [2631], spinal cord [2786], locus coeruleus, hippocampus [140] and hypothalamus [141, 1468], but exhibit agonist activity at some peripheral sites [141, 887]. The chimeric peptides M15, M32, M35, M40 and C7 are agonists at GAL_1_ receptors expressed endogenously in Bowes human melanoma cells [1938], and at heterologously expressed recombinant GAL_1_, GAL_2_ and GAL_3_ receptors [708, 2413, 2414]. Further studies described the synthesis of a series of novel, systemically-active, galanin analogues, with modest preferential binding at the GAL_2_ receptor. Specific chemical modifications to the galanin backbone increased brain levels of these peptides after *i.v.* injection and several of these peptides exerted a potent antidepressant-like effect in mouse models of depression [2241]. More recent studies have identified synthetic spexin (SPX)-based peptides that are selective GAL_2_ receptor agonists [1459, 2166].

Further reading on Galanin receptorsFoordSM
 (2005) International Union of Pharmacology. XLVI. G protein-coupled receptor list. Pharmacol Rev
57: 279–8815914470
10.1124/pr.57.2.5LangR
 (2015) Physiology, signaling, and pharmacology of galanin peptides and receptors: three decades of emerging diversity. Pharmacol Rev
67: 118–7525428932
10.1124/pr.112.006536LangR
 (2011) The galanin peptide family in inflammation. Neuropeptides
45: 1–821087790
10.1016/j.npep.2010.10.005LawrenceC
 (2011) Galanin-like peptide (GALP) is a hypothalamic regulator of energy homeostasis and reproduction. Front Neuroendocrinol
32: 1–920558195
10.1016/j.yfrne.2010.06.001PMC2950899WeblingKE
 (2012) Galanin receptors and ligands. Front Endocrinol (Lausanne)
3: 14623233848
10.3389/fendo.2012.00146PMC3516677

## Ghrelin receptor

G protein-coupled receptors → Ghrelin receptor

**Overview**: The ghrelin receptor (**nomenclature as agreed by the NC-IUPHAR Subcommittee for the Ghrelin receptor** [533]) is activated by a 28 amino-acid peptide originally isolated from rat stomach, where it is cleaved from a 117 amino-acid precursor (*GHRL*, Q9UBU3). The human gene encoding the precursor peptide has 83% sequence homology to rat preproghrelin, although the mature peptides from rat and human differ by only two amino acids [1673]. Alternative splicing results in the formation of a second peptide, [des-Gln^14^]ghrelin (*GHRL*, Q9UBU3) with equipotent biological activity [1055]. A unique post-translational modification (octanoylation of Ser^3^, catalysed by ghrelin Ο-acyltransferase (*MBOAT4*, Q96T53) [2866] occurs in both peptides, essential for full activity in binding to ghrelin receptors in the hypothalamus and pituitary, and for the release of growth hormone from the pituitary [1328]. Structure activity studies showed the first five N-terminal amino acids to be the minimum required for binding [158], and receptor mutagenesis has indicated overlap of the ghrelin binding site with those for small molecule agonists and allosteric modulators of ghrelin (*GHRL*, Q9UBU3) function [1044]. An endogenous antagonist and inverse agonist called Liver enriched antimicrobial peptide 2 (Leap2), expressed primarily in hepatocytes and in enterocytes of the proximal intestine [787, 1588] inhibits ghrelin receptor-induced GH secretion and food intake [787]. The secretion of Leap2 and ghrelin is inversely regulated under various metabolic conditions [1637]. In cell systems, the ghrelin receptor is constitutively active [1045], but this is abolished by a naturally occurring mutation (A204E) that results in decreased cell surface receptor expression and is associated with familial short stature [1983].

**Table:** 

Nomenclature	ghrelin receptor
HGNC, UniProt	*GHSR*, Q92847
Potency order of endogenous ligands	ghrelin (*GHRL*, Q9UBU3) = [des-Gln^14^]ghrelin (*GHRL*, Q9UBU3) [157, 1673]
Antagonists	liver enriched antimicrobial peptide 2 (*LEAP2*, Q969E1) (pIC_50_ 8.2) [787]
Selective antagonists	GSK1614343 (pIC_50_ 8.4) [2242], GSK1614343 (pK_B_ 8) [2020] – Rat
Labelled ligands	[^125^I][His^9^]ghrelin (human) (Agonist) [1226], [^125^I][Tyr^4^]ghrelin (human) (Agonist) [1818]

**Comments**: [des-octanoyl]ghrelin (*GHRL*, Q9UBU3) has been shown to bind (as [^125^I]Tyr^4^-des-octanoyl-ghrelin) and have effects in the cardiovascular system [157], which raises the possible existence of different receptor subtypes in peripheral tissues and the central nervous system. A potent inverse agonist has been identified ([D-Arg^1^, D-Phe^5^, D-Trp^7,9^, Leu^11^]substance P, p*D*_2_ 8.3; [1042]). Ulimorelin, described as a ghrelin receptor agonist (p*K*_i_ 7.8 and p*D*_2_ 7.5 at human recombinant ghrelin receptors), has been shown to stimulate ghrelin receptor mediated food intake and gastric emptying but not elicit release of growth hormone, or modify ghrelin stimulated growth hormone release, thus pharmacologically discriminating the orexigenic and gastrointestinal actions of ghrelin (GHRL, Q9UBU3) from the release of growth hormone [724]. Similar discrimination of ghrelin receptor mediated physiological functions can be obtained by activation of distinct signaling pathways [1708]. A number of selective antagonists have been reported, including peptidomimetic [1817] and non-peptide small molecules including GSK1614343 [2004, 2020, 2242].

Further reading on Ghrelin receptorAndrewsZB. (2011) The extra-hypothalamic actions of ghrelin on neuronal function. Trends Neurosci
34: 31–4021035199
10.1016/j.tins.2010.10.001AngelidisG
 (2010) Current and potential roles of ghrelin in clinical practice. J Endocrinol Invest
33: 823–3821293171
10.1007/BF03350350BriggsDI
 (2011) Metabolic status regulates ghrelin function on energy homeostasis. Neuroendocrinology
93: 48–5721124019
10.1159/000322589CallaghanB
 (2014) Novel and conventional receptors for ghrelin, desacyl-ghrelin, and pharmacologically related compounds. Pharmacol Rev
66: 984–100125107984
10.1124/pr.113.008433DavenportAP
 (2005) International Union of Pharmacology. LVI. Ghrelin receptor nomenclature, distribution, and function. Pharmacol Rev
57: 541–616382107
10.1124/pr.57.4.1

## Glucagon receptor family

G protein-coupled receptors → Glucagon receptor family

**Overview**: The glucagon family of receptors (**nomenclature as agreed by the NC-IUPHAR Subcommittee on the Glucagon receptor family** [1684]) are activated by the endogenous peptide (27–44 aa) hormones glucagon (*GCG*, P01275), glucagon-like peptide 1 (*GCG*, P01275), glucagon-like peptide 2 (*GCG*, P01275), glucose-dependent insulinotropic polypeptide (also known as gastric inhibitory polypeptide (*GIP*, P09681)), GHRH (*GHRH*, P01286) and secretin (*SCT*, P09683). One common precursor (*GCG*) generates glucagon (*GCG*, P01275), glucagon-like peptide 1 (*GCG*, P01275) and glucagon-like peptide 2 (*GCG*, P01275) peptides [1114]. For a recent review on the current understanding of the structures of GLP-1 and GLP-1R, the molecular basis of their interaction, and the associated signaling events see de Graaf *et al*., 2016 [861].

**Table:** 

Nomenclature	GHRH receptor	GIP receptor	GLP-1 receptor
HGNC, UniProt	*GHRHR*, Q02643	*GIPR*, P48546	*GLP1R*, P43220
Endogenous agonists	GHRH (*GHRH*, P01286)	gastric inhibitory polypeptide (*GIP*, P09681) [2694]	glucagon-like peptide 1-(7-36) amide (*GCG*,P01275) [1190], glucagon-like peptide 1-(7-37) (*GCG*, P01275) [586]
Agonists	JI-38 [332], sermorelin	–	liraglutide [1313], lixisenatide [2769], WB4-24 [677]
Selective agonists	BIM28011 [503], tesamorelin	–	semaglutide [1418], exendin-4 [1747], exendin-4 [1190], exendin-3 (P20394) [2145]
Selective antagonists	JV-1-36 (p*K*_i_ 10.1–10.4) [2290, 2671, 2672] – Rat, JV-1-38 (p*K*_i_ 10.1) [2290, 2671, 2672] – Rat	[Pro^3^]GIP [784] – Mouse	exendin-(9-39) (p*K*_i_ 8.1) [1190], GLP-1-(9-36) (pIC_50_ 6.9) [1779] – Rat, T-0632 (pIC_50_ 4.7) [2581]
Labelled ligands	[^125^I]GHRH (human) (Agonist) [252] – Rat	[^125^I]GIP (human) (Agonist) [757] – Rat	[^125^I]GLP-1-(7-36)-amide (Agonist) [1190], [^125^I]exendin-(9-39) (Antagonist) (p*K*_d_ 8.3) [1190], [^125^I]GLP-1-(7-37) (human) (Agonist)

**Table:** 

Nomenclature	GLP-2 receptor	glucagon receptor	secretin receptor
HGNC, UniProt	*GLP2R*, O95838	*GCGR*, P47871	*SCTR*, P47872
Endogenous agonists	glucagon-like peptide 2 (*GCG*, P01275) [2578]	glucagon (*GCG*, P01275) [2061]	secretin (*SCT*, P09683) [443]
Agonists	teduglutide [1699]	NNC1702 [2923]	–
Selective agonists	apraglutide [947, 2404]	–	–
Selective antagonists	–	L-168,049 (pIC_50_ 8.4) [358], adomeglivant (pKi 8.2) [1235, 1241], des-His1-[Glu9]glucagon-NH2 (pA2 7.2) [2636, 2637] – Rat, NNC 92-1687 (pKi 5) [1605], BAY27-9955 [2033]	[(CH_2_NH)^4,5^]secretin (pKi 5.3) [908]
Labelled ligands	–	[^125^I]glucagon (human, mouse, rat) (Agonist)	[^125^I](Tyr^10^)secretin-27 (rat) (Agonist) [2632] – Rat

**Comments**: The glucagon receptor has been reported to interact with receptor activity modifying proteins (RAMPs), specifically RAMP2, in heterologous expression systems [451], although the physiological significance of this has yet to be established.

Further reading on Glucagon receptor familyChangR
 (2020) Cryo-electron microscopy structure of the glucagon receptor with a dual-agonist peptide. J Biol Chem
295: 9313–932532371397
10.1074/jbc.RA120.013793PMC7363120DongM
 (2020) Structure and dynamics of the active Gs-coupled human secretin receptor. Nat Commun
11: 413732811827
10.1038/s41467-020-17791-4PMC7435274DruckerDJ. (2019) The Discovery of GLP-2 and Development of Teduglutide for Short Bowel Syndrome. ACS Pharmacol Transl Sci
2: 134–14232219218
10.1021/acsptsci.9b00016PMC7088900HolstJJ
 (2020) GIP as a Therapeutic Target in Diabetes and Obesity: Insight From Incretin Co-agonists. J Clin Endocrinol Metab
105:10.1210/clinem/dgaa327PMC730807832459834LiangYL
 (2020) Toward a Structural Understanding of Class B GPCR Peptide Binding and Activation. Mol Cell
77: 656–668.e532004469
10.1016/j.molcel.2020.01.012ZhangX
 (2020) Differential GLP-1R Binding and Activation by Peptide and Non-peptide Agonists. Mol Cell
80: 485–500.e733027691
10.1016/j.molcel.2020.09.020ZhouF
 (2020) Structural basis for activation of the growth hormone-releasing hormone receptor. Nat Commun
11: 520533060564
10.1038/s41467-020-18945-0PMC7567103

## Glycoprotein hormone receptors

G protein-coupled receptors → Glycoprotein hormone receptors

**Overview**: Glycoprotein hormone receptors (**provisional nomenclature** [712]) are activated by a non-covalent heterodimeric glycoprotein made up of a common α chain (glycoprotein hormone common alpha subunit (*CGA*, P01215) *CGA*, P01215), with a unique β chain that confers the biological specificity to FSH (*CGA* *FSHB*, P01215 P01225), LH (*CGA* *LHB*, P01215 P01229), hCG (*CGA* *CGB3*, P01215 P01233) or TSH (*CGA* *TSHB*, P01215 P01222). There is binding cross-reactivity across the endogenous agonists for each of the glycoprotein hormone receptors. The deglycosylated hormones appear to exhibit reduced efficacy at these receptors [537, 2246].

**Table:** 

Nomenclature	FSH receptor	LH receptor	TSH receptor
HGNC, UniProt	*FSHR*, P23945	*LHCGR*, P22888	*TSHR*, P16473
Potency order of endogenous ligands	FSH (*CGA* *FSHB*, P01215 P01225)	LH (*CGA* *LHB*, P01215 P01229), hCG (CGA *CGB3*, P01215 P01233) [1167, 1826]	TSH (CGA *TSHB*, P01215 P01222)
Labelled ligands	[^125^I]FSH (human) (Agonist)	[^125^I]LH (Agonist), [^125^I]chorionic gonadotropin (human) (Agonist)	[^125^I]TSH (human) (Agonist)

Further reading on Glycoprotein hormone receptorsJiangX
 (2012) Structure of follicle-stimulating hormone in complex with the entire ectodomain of its receptor. Proc Natl Acad Sci USA
109: 12491–622802634
10.1073/pnas.1206643109PMC3411987KleinauG

Thyroid Disease Manager. Accessed on 2017-02-23.TaoYX
 (2009) Follicle stimulating hormone receptor mutations and reproductive disorders. Prog Mol Biol Transl Sci
89: 115–3120374735
10.1016/S1877-1173(09)89005-4TroppmannB
 (2013) Structural and functional plasticity of the luteinizing hormone/choriogonadotrophin receptor. Hum Reprod Update
19: 583–60223686864
10.1093/humupd/dmt023

## Gonadotrophin-releasing hormone receptors

G protein-coupled receptors → Gonadotrophin-releasing hormone receptors

**Overview**: GnRH_1_ and GnRH_2_ receptors (**provisonal nomenclature** [712], also called Type I and Type II GnRH receptor, respectively [1740]) have been cloned from numerous species, most of which express two or three types of GnRH receptor [1739, 1740, 2387]. GnRH I (*GNRH1*, P01148) (p-Glu-His-Trp-Ser-Tyr-Gly-Leu-Arg-Pro-Gly-NH2) is a hypothalamic decapeptide also known as luteinizing hormone-releasing hormone, gonadoliberin, luliberin, gonadorelin or simply as GnRH. It is a member of a family of similar peptides found in many species [1739, 1740, 2387] including GnRH II (*GNRH2*, O43555) (pGlu-His-Trp-Ser-His-Gly-Trp-Tyr-Pro-Gly-NH_2_ (which is also known as chicken GnRH-II). Receptors for three forms of GnRH exist in some species but only GnRH I and GnRH II and their cognate receptors have been found in mammals [1739, 1740, 2387]. GnRH_1_ receptors are expressed by pituitary gonadotrophs, where they mediate the effects of GnRH on gonadotropin hormone synthesis and secretion that underpin central control of mammalian reproduction. GnRH analogues are used in assisted reproduction and to treat steroid hormone-dependent conditions [1262]. Notably, agonists cause desensitization of GnRH-stimulated gonadotropin secretion and the consequent reduction in circulating sex steroids is exploited to treat hormone-dependent cancers of the breast, ovary and prostate [1262]. GnRH_1_ receptors are selectively activated by GnRH I and all lack the COOH-terminal tails found in other GPCRs. GnRH_2_ receptors do have COOH-terminal tails and (where tested) are selective for GnRH II over GnRH I. GnRH_2_ receptors are expressed by some primates but not by humans [1796]. Phylogenetic classifications divide GnRH receptors into three [1740] or five groups [2790] and highlight examples of gene loss through evolution, with humans retaining only one ancient gene. The structure of the GnRH_1_ receptor in complex with elagolix has been elucidated [2861].

**Table:** 

Nomenclature	GnRH_1_ receptor	GnRH_2_ receptor
HGNC, UniProt	*GNRHR*, P30968	*GNRHR2*, Q96P88
Potency order of endogenous ligands	GnRH I (GNRH1, P01148) > GnRH II (GNRH2, O43555) [1740]	GnRH II (*GNRH2*, O43555) > GnRH I (*GNRH1*, P01148) (Monkey) [1738]
Endogenous agonists	GnRH I (GNRH1, P01148) [1572], GnRH II (GNRH2, O43555) [706, 1572, 2457]	GnRH II (*GNRH2*, O43555) [1738] – Monkey, GnRH I (*GNRH1*, P01148) [1738, 1740] – Monkey
Selective agonists	buserelin [1865, 1866], triptorelin [154], leuprolide [2472], goserelin, histrelin, nafarelin	–
Antagonists	iturelix (p*K*_i_ 9.5) [2188]	–
Selective antagonists	cetrorelix (p*K*_i_ 9.3–10) [155, 156, 2472], abarelix (p*K*_i_ 9.1–9.5) [2472], elagolix (p*K*_i_ 9.1) [398, 1401], degarelix (p*K*_i_ 8.8) [2658], ganirelix	trptorelix-1 [1620] – Monkey
Labelled ligands	[^125^I]cetrorelix (Antagonist) (pK_d_ 9.7) [1034], [^125^I]triptorelin (Agonist) [561] – Rat, [^125^I]buserelin (Agonist) [1384] – Rat, [^125^I]GnRH I (human, mouse, rat) (Agonist)	–

**Comments**: GnRH_1_ and GnRH_2_ receptors couple primarily to G_q/11_ [881] but coupling to G_s_ and G_i_ is evident in some systems [1363, 1384]. GnRH_2_ receptors may also mediate (heterotrimeric) G protein-independent signalling to protein kinases [365]. There is increasing evidence for expression of GnRH receptors on hormone-dependent cancer cells where they can exert antiproliferative and/or proapoptotic effects and mediate effects of cytotoxins conjugated to GnRH analogues [418, 952, 1520, 2289]. In some human cancer cell models GnRH II (*GNRH2*, O43555) is more potent than GnRH I (*GNRH1*, P01148), implying mediation by GnRH_2_ receptors [884], but GnRH_2_ receptors are not expressed by humans because the human GNRHR2 gene contains a frame shift and internal stop codon [1796]. The possibility remains that this gene generates GnRH_2_ receptor-related proteins (other than the full-length receptor) that mediate responses to GnRH II (*GNRH2*, O43555) (see [1871]). Alternatively, evidence for multiple active GnRH receptor conformations [365, 366, 697, 1681, 1740] raises the possibility that GnRH_1_ receptor-mediated proliferation inhibition in hormone-dependent cancer cells is dependent upon a conformation that couples to G_i_ rather than G_q/11_ proteins as in pituitary cells [366, 1681]. Loss-of-function mutations in the GnRH_1_ receptor and deficiency of GnRH I (*GNRH1*, P01148) are associated with hypogonadotropic hypogonadism although some’loss of function’ mutations may actually prevent trafficking of ’functional’ GnRH_1_ receptors to the cell surface, as evidenced by recovery of function by nonpeptide antagonists [1444]. Human GnRH_1_ receptors are poorly expressed at the cell surface because of failure to meet structural quality control criteria for endoplasmic reticulum exit [698, 1444], and this increases susceptibility to point mutations that further impair trafficking [698, 1444]. GnRH receptor signalling may require receptor oligomerisation [483, 1361].

Further reading on Gonadotrophin-releasing hormone receptorsDesaulniersAT
 (2017) Expression and Role of Gonadotropin-Releasing Hormone 2 and Its Receptor in Mammals. Front Endocrinol (Lausanne)
8: 26929312140
10.3389/fendo.2017.00269PMC5732264LimontaP
 (2012) GnRH receptors in cancer: from cell biology to novel targeted therapeutic strategies. Endocr Rev
33: 784–81122778172
10.1210/er.2012-1014McArdleCA and RobersonMS.. (2015) In Knobil and Neill’s Physiology of Reproduction (4th edition). Edited by PlantTM and ZeleznikAJ.: Elsevier Inc.: [ISBN: 9780123971753]MillarRP
 (2004) Gonadotropin-releasing hormone receptors. Endocr Rev
25: 235–7515082521
10.1210/er.2003-0002TaoYX
 (2014) Chaperoning G protein-coupled receptors: from cell biology to therapeutics. Endocr Rev
35: 602–4724661201
10.1210/er.2013-1121PMC4105357

## GPR18, GPR55 and GPR119

G protein-coupled receptors → GPR18, GPR55 and GPR119

**Overview**: GPR18, GPR55 and GPR119 (**provisional nomenclature**), although showing little structural similarity to CB_1_ and CB_2_ cannabinoid receptors, respond to endogenous agents analogous to the endogenous cannabinoid ligands, as well as some natural/synthetic cannabinoid receptor ligands [2029]. Although there are multiple reports to indicate that GPR18, GPR55 and GPR119 can be activated *in vitro* by N-arachidonoylglycine, lysophosphatidylinositol and N-oleoylethanolamide, respectively, there is a lack of evidence for activation by these lipid messengers *in vivo*. As such, therefore, these receptors retain their orphan status.

**Table:** 

Nomenclature	GPR18	GPR55	GPR119
HGNC, UniProt	*GPR18*, Q14330	*GPR55*, Q9Y2T6	*GPR119*, Q8TDV5
Potency order of endogenous ligands	–	–	N-oleoylethanolamide, N-palmitoylethanolamine > SEA (anandamide is ineffective) [1967]
Endogenous agonists	N-arachidonoylglycine [1325]	lysophosphatidylinositol [995, 1940, 2436], 2-arachidonoylglycerolphosphoinositol [1942]	N-oleoylethanolamide [456, 1967, 2436], N-palmitoylethanolamine, SEA
Selective agonists	–	AM251 [995, 1216, 2238]	AS1269574 [2897], PSN632408 [1967], PSN375963 [1967]
Selective antagonists	–	CID16020046 (apparent pA_2_) (pA_2_ 7.3) [1218], ML193 (pIC_50_ 6.7) [1008]	–
Comments	The pairing of N-arachidonoylglycine with GPR18 was not replicated in two studies based on arrestin assays [2436, 2885]. See [532] for discussion.	See reviews [532] and [2376].	In addition to those shown above, further small molecule agonists have been reported [929].

**Comments**: GPR18 failed to respond to a variety of lipid-derived agents in an *in vitro* screen [2885], but has been reported to be activated by Δ^9^-tetrahydrocannabinol [1698]. GPR55 responds to AM251 and rimonabant at micromolar concentrations, compared to their nanomolar affinity as CB_1_ receptor antagonists/inverse agonists [2029]. It has been reported that lysophosphatidylinositol acts at other sites in addition to GPR55 [2856]. N-Arachidonoylserine has been suggested to act as a low efficacy agonist/antagonist at GPR18 *in vitro* [1696]. It has also been suggested oleoyl-lysophosphatidylcholine acts, at least in part, through GPR119 [1901]. Although PSN375963 and PSN632408 produce GPR119-dependent responses in heterologous expression systems, comparison with N-oleoylethanolamide-mediated responses suggests additional mechanisms of action [1901].

Further reading on GPR18, GPR55 and GPR119DavenportAP
 (2013) International Union of Basic and Clinical Pharmacology. LXXXVIII. G protein-coupled receptor list: recommendations for new pairings with cognate ligands. Pharmacol Rev
65: 967–8623686350
10.1124/pr.112.007179PMC3698937HassingHA
 (2016) Biased signaling of lipids and allosteric actions of synthetic molecules for GPR119. Biochem Pharmacol
119: 66–7527569424
10.1016/j.bcp.2016.08.018LiuB
 (2015) GPR55: from orphan to metabolic regulator?
Pharmacol Ther
145: 35–4224972076
10.1016/j.pharmthera.2014.06.007PertweeRG
 (2010) International Union of Basic and Clinical Pharmacology. LXXIX. Cannabinoid receptors and their ligands: beyond CB_1_ and CB_2_. Pharmacol Rev
62: 588–63121079038
10.1124/pr.110.003004PMC2993256

## Histamine receptors

G protein-coupled receptors → Histamine receptors

**Overview**: Histamine receptors (**nomenclature as agreed by the NC-IUPHAR Subcommittee on Histamine Receptors** [1014, 1984]) are activated by the endogenous ligand histamine. Marked species differences exist between histamine receptor orthologues [1014]. The human and rat H_3_ receptor genes are subject to significant splice variance [122]. The potency order of histamine at histamine receptor subtypes is H_3_ = H_4_ > H_2_ > H_1_ [1984]. Some agonists at the human H_3_ receptor display significant ligand bias [2180]. Antagonists of all 4 histamine receptors have clinical uses: H_1_ antagonists for allergies (*e.g*. cetirizine), H_2_ antagonists for acid-reflux diseases (*e.g*. ranitidine), H_3_ antagonists for narcolepsy (*e.g*. pitolisant/WAKIX; Registered) and H_4_ antagonists for atopic dermatitis (*e.g*. adriforant; Phase IIa) [1984] and vestibular neuritis (AUV) (SENS-111 (Seliforant, previously UR-63325), entered and completed vestibular neuritis (AUV) Phase IIa efficacy and safety trials, respectively) [88, 2679].

**Table:** 

Nomenclature	H_1_ receptor	H_2_ receptor
HGNC, UniProt	*HRH1*, P35367	*HRH2*, P25021
Selective agonists	methylhistaprodifen [2335], histaprodifen [1519]	amthamine [1354]
Antagonists	cyproheptadine (p*K*_i_ 10.2) [1761], promethazine (pKi 9.6) [810], mepyramine (Inverse agonist) (p*K*_i_ 8.7–9) [242, 2144], cetirizine (Inverse agonist) (p*K*_i_ 8.2) [1761], diphenhydramine (p*K*_i_ 7.9) [242]	–
Selective antagonists	clemastine (p*K*_i_ 10.3) [93], desloratadine ((p*K*_i_ 9) [1491], triprolidine (p*K*_i_ 8.5–9) [242, 1761], azelastine (p*K*_i_ 8.9) [2086], astemizole (p*K*_i_ 8.5) [2007]	tiotidine (p*K*_i_ 7.5) [195] – Rat, ranitidine ((p*K*_i_ 7.1) [1486], cimetidine ((p*K*_i_ 6.8) [344]
Labelled ligands	[^3^H]pyrilamine (Antagonist, Inverse agonist) (pKd 8.4–9.1) [543, 1761, 2307, 2335], [^11^C]doxepin (Antagonist) (pKd 9) [1117], [^11^C]pyrilamine (Antagonist, Inverse agonist)	[^125^I]iodoaminopotentidine (Antagonist) (pK_d_ 8.7) [1369] – Rat, [^3^H]tiotidine (Antagonist) (pKd 7.7–8.7) [1772]

**Table:** 

Nomenclature	H_3_ receptor	H_4_ receptor
HGNC, UniProt	*HRH3*, Q9Y5N1	*HRH4*, Q9H3N8
Selective agonists	GSK-189254 (Inverse agonist) [1703], immethridine [1302], methimepip [1301], MK-0249 (Inverse agonist) [1842]	clobenpropit (Partial agonist) [663, 1519, 1542, 1543, 1812], 4-methylhistamine [786, 1519], ST-1006 [1984], VUF 8430 [1518]
Antagonists	iodophenpropit (pKi 8.2–8.7) [2785, 2822]	SENS-111 [2038]
Selective antagonists	pitolisant (p*K*_i_ 8.1–8.6) [1431, 1984], A331440 (p*K*_i_ 8.5) [930], conessine ((p*K*_i_ 8.3) [1984], MK-0249 (p*K*_i_ 8.2) [1984], thioperamide (Selective for H3/H4 compared to H1 and H3.) (p*K*_i_ 7.1–7.7) [472, 662, 663, 1516, 1569, 2785, 2822], ciproxifan (p*K*_i_ 6.7–7.3) [472, 662, 663, 1516, 1984, 2822]	adriforant (p*K*_i_ 8.3) [1984], INCB-38579 (p*K*_i_ 8.3) [1984], JNJ 7777120 (p*K*_i_ 7.8–8.3) [1519, 2419, 2579], JNJ-39758979 (p*K*_i_ 7.9) [1984, 2280], thioperamide (Selective for H3/H4 compared to H1 and H3.) (pKi 6.3–7.6) [662, 663, 1542, 1543, 1812, 2957]
Labelled ligands	[^123^I]iodoproxyfan (Antagonist) (pKd 10.2) [1516], [^125^I]iodophenpropit (Antagonist) (pKd 9.2) [1148] – Rat, [^3^H](*R*)-α-methylhistamine (Agonist) [1542], *N*-[^3^H]α-methylhistamine (Agonist) [402] – Mouse	[^3^H]JNJ 7777120 (Antagonist) (pK_d_ 8.4) [2579]

**Comments**: Histaprodifen and methylhistaprodifen are reduced efficacy agonists. The H_4_ receptor appears to exhibit broadly similar pharmacology to the H_3_ receptor for imidazole-containing ligands, although (*R*)-α-methylhistamine and *N*-α-methylhistamine are less potent, while clobenpropit acts as a reduced efficacy agonist at the H_4_ receptor and an antagonist at the H_3_ receptor [1542, 1849, 1889, 1926, 2957]. Moreover, 4-methylhistamine is identified as a high affinity, full agonist for the human H_4_ receptor [1519]. [^3^H]histamine has been used to label the H_4_ receptor in heterologous expression systems. 

Further reading on Histamine receptorsGbahouF
 (2012) The histamine autoreceptor is a short isoform of the H_3_ receptor. Br J Pharmacol
166: 1860–7122356432
10.1111/j.1476-5381.2012.01913.xPMC3402810Nieto-AlamillaG
 (2016) The Histamine H3 Receptor: Structure, Pharmacology, and Function. Mol Pharmacol
90: 649–67327563055
10.1124/mol.116.104752PanulaP
 (2015) International Union of Basic and Clinical Pharmacology. XCVIII. Histamine Receptors. Pharmacol Rev
67: 601–5526084539
10.1124/pr.114.010249PMC4485016van RijnRM
 (2008) Cloning and characterization of dominant negative splice variants of the human histamine H4 receptor. Biochem J
414: 121–3118452403
10.1042/BJ20071583

## Hydroxycarboxylic acid receptors

G protein-coupled receptors → Hydroxycarboxylic acid receptors

**Overview**: The hydroxycarboxylic acid family of receptors (ENSFM00500000271913, **nomenclature as agreed by the NC-IUPHAR Subcommittee on Hydroxycarboxylic acid receptors** [532, 1928]) respond to organic acids, including the endogenous hydroxy carboxylic acids 3-hydroxy butyric acid and L-lactic acid, as well as the lipid lowering agents nicotinic acid (niacin), acipimox and acifran [2425, 2618, 2799]. These receptors were provisionally described as nicotinic acid receptors, although nicotinic acid shows submicromolar potency at HCA_2_ receptors only and is unlikely to be the natural ligand [2618, 2799].

**Table:** 

Nomenclature	HCA_1_ receptor	HCA_2_ receptor	HCA_3_ receptor
HGNC, UniProt	*HCAR1*, Q9BXC0	*HCAR2*, Q8TDS4	*HCAR3*, P49019
Potency order of endogenous ligands	–	β-D-hydroxybutyric acid > butyric acid	–
Endogenous agonists	L-lactic acid [21, 333, 1544, 2436]	β-D-hydroxybutyric acid [2511], butyric acid	3-hydroxyoctanoic acid [20]
Agonists	compound 2 [2249], 3,5-dihydroxybenzoic acid [1541]	SCH 900271 [1973], GSK256073 [2445]	D-phenyllactic acid [2031]
Selective agonists	–	MK 6892 [2359], MK 1903 [217], nicotinic acid [2425, 2618, 2799], acipimox [2425, 2799], monomethyl fumarate [2540]	compound 6o [2398], IBC 293 [2337]
Labelled ligands	–	[^3^H]nicotinic acid (Agonist) [2425, 2618, 2799]	–

**Comments**: Further closely-related GPCRs include the 5-oxoeicosanoid receptor (*OXER1*, Q8TDS5) and *GPR31* (O00270). Lactate activates HCA_1_ on adipocytes in an autocrine manner. It inhibits lipolysis and thereby promotes anabolic effects. HCA_2_ and HCA_3_ regulate adipocyte lipolysis and immune functions under conditions of increased FFA formation through lipolysis (e.g., during fasting). HCA_2_ agonists acting mainly through the receptor on immune cells exert antiatherogenic and anti-inflammatory effects. HCA_2_ is also a receptor for butyrate and mediates some of the beneficial effects of short-chain fatty acids produced by gut microbiota. HCA_3_ has been shown to be activated by aromatic D-amino acids, and by D-phenyllactic acid, a metabolite of gut lactic acid bacteria [2031].

Further reading on Hydroxycarboxylic acid receptorsBoatmanPD
 (2008) Nicotinic acid receptor agonists. J Med Chem
51: 7653–6218983141
10.1021/jm800896zGraffEC
 (2016) Anti-inflammatory effects of the hydroxycarboxylic acid receptor 2. Metab Clin Exp
65: 102–1326773933
10.1016/j.metabol.2015.10.001KamannaVS
 (2013) Recent advances in niacin and lipid metabolism. Curr Opin Lipidol
24: 239–4523619367
10.1097/MOL.0b013e3283613a68KennedyL
 (2022) Lactate receptor HCAR1 regulates neurogenesis and microglia activation after neonatal hypoxia-ischemia. Elife
11:10.7554/eLife.76451PMC936311535942676OffermannsS. (2017) Hydroxy-Carboxylic Acid Receptor Actions in Metabolism. Trends Endocrinol Metab
28: 227–23628087125
10.1016/j.tem.2016.11.007OffermannsS
 (2011) International Union of Basic and Clinical Pharmacology. LXXXII: Nomenclature and Classification of Hydroxy-carboxylic Acid Receptors (GPR81, GPR109A, and GPR109B). Pharmacol Rev
63: 269–9021454438
10.1124/pr.110.003301OffermannsS
 (2015) Nutritional or pharmacological activation of HCA(2) ameliorates neuroinflammation. Trends Mol Med
21: 245–5525766751
10.1016/j.molmed.2015.02.002

## Kisspeptin receptor

G protein-coupled receptors → Kisspeptin receptor

**Overview**: The kisspeptin receptor (**nomenclature as agreed by the NC-IUPHAR Subcommittee on the kisspeptin receptor** [1291]), like neuropeptide FF (NPFF), prolactin-releasing peptide (PrP) and QRFP receptors (provisional nomen-clature) responds to endogenous peptides with an arginine-phenylalanine-amide (RFamide) motif. Kisspeptin-54 (*KISS1*, Q15726) (KP54, originally named metastin), kisspeptin-13 (*KISS1*, Q15726) (KP13) and kisspeptin-10 (*KISS1*) (KP10) are biologically-active peptides cleaved from the *KISS1* (Q15726) gene product. Kisspeptins have roles in, for example, cancer metastasis, fertility/puberty regulation and glucose homeostasis.

**Table:** 

Nomenclature	kisspeptin receptor
HGNC, UniProt	*KISS1R*, Q969F8
Endogenous agonists	kisspeptin-10 (*KISS1*) [1342, 1939], kisspeptin-54 (*KISS1*, Q15726) [1342, 1939], kisspeptin-14 (*KISS1*, Q15726) [1342], kisspeptin-13 (KISS1, Q15726) [1342]
Selective agonists	4-fluorobenzoyl-FGLRW-NH2 [2595], [dY]^1^KP-10 [513] – Mouse, TAK-448 [1904]
Selective antagonists	peptide 234 [2209]
Labelled ligands	[^125^I]Tyr^45^-kisspeptin-15 (Agonist) [1939], [^125^I]kisspeptin-13 (human) (Agonist) [1702], [^125^I]kisspeptin-10 (human) (Agonist) [1342], [^125^I]kisspeptin-14 (human) (Agonist) [1702], [d-Tyr-^14^C]TAK-448 (Agonist) [1804]

**Comments**: 2-acylamino-4,6-diphenylpyridine derivatives have been described and are the first small molecule kisspeptin receptor antagonists reported with potential for treatment of sex-hormone dependent diseases such as prostate cancer and endometriosis [537, 1314].

Further reading on Kisspeptin receptorHarterCJL
 (2018) The role of kisspeptin neurons in reproduction and metabolism. J Endocrinol
238: R173–R18330042117
10.1530/JOE-18-0108KandaS
 (2013) Structure, synthesis, and phylogeny of kisspeptin and its receptor. Adv Exp Med Biol
784: 9–2623550000
10.1007/978-1-4614-6199-9_2KirbyHR
 (2010) International Union of Basic and Clinical Pharmacology. LXXVII. Kisspeptin receptor nomenclature, distribution, and function. Pharmacol Rev
62: 565–7821079036
10.1124/pr.110.002774PMC2993257OakleyAE
 (2009) Kisspeptin signaling in the brain. Endocr Rev
30: 713–4319770291
10.1210/er.2009-0005PMC2761114PasquierJ
 (2014) Molecular evolution of GPCRs: Kisspeptin/kisspeptin receptors. J Mol Endocrinol
52: T101–1724577719
10.1530/JME-13-0224

## Leukotriene receptors

G protein-coupled receptors → Leukotriene receptors

**Overview**: The leukotriene receptors (**nomenclature as agreed by the NC-IUPHAR subcommittee on Leukotriene Receptors** [109, 110]) are activated by the endogenous ligands leukotrienes (LT), synthesized from lipoxygenase metabolism of arachidonic acid. The human BLT_1_ receptor is the high affinity LTB_4_ receptor whereas the BLT_2_ receptor in addition to being a low-affinity LTB_4_ receptor also binds several other lipoxygenase-products, such as 12S-HETE, 12S-HPETE, 15S-HETE, and the thromboxane synthase product 12-hydroxyheptadecatrienoic acid. The BLT receptors mediate chemotaxis and immunomodulation in several leukocyte populations and are in addition expressed on non-myeloid cells, such as vascular smooth muscle and endothelial cells. In addition to BLT receptors, LTB_4_ has been reported to bind to the peroxisome proliferator activated receptor (PPAR) α [1526] and the vanilloid TRPV1 ligand-gated nonselective cation channel [1697]. The crystal structure of the BLT_1_ receptor was initially determined in complex with selective antagonists [1051, 1723] and has recently been extended to the cryo-electron microscopy structure of LTB_4_-bound human BLT_1_ receptor at 2.91 Å resolution [2731]. The receptors for the cysteinyl-leukotrienes (*i.e*. LTC_4_, LTD_4_ and LTE_4_) are termed CysLT_1_ and CysLT_2_ and exhibit distinct expression patterns in human tissues, mediating for example smooth muscle cell contraction, regulation of vascular permeability, and leukocyte activation. Quite recently, the the crystal structures of both receptors have been solved, the CysLT_1_ in complex with zafirlukast and pranlukast [1575] and the CysLT_2_ in complex with three dual CysLT_1_/CysLT_2_ antagonists [904]. There is also evidence in the literature for additional CysLT receptor subtypes, derived from functional in vitro studies, radioligand binding and in mice lacking both CysLT_1_ and CysLT_2_ receptors [110]. Cysteinyl-leukotrienes have also been suggested to signal through the P2Y_12_ receptor [730, 1911, 1990], GPR17 [461] and GPR99 [1208].

**Table:** 

Nomenclature	BLT_1_ receptor	BLT_2_ receptor	CysLT_1_ receptor	CysLT_2_ receptor	OXE receptor	FPR2/ALX
HGNC, UniProt	*LTB4R*, Q15722	*LTB4R2*, Q9NPC1	*CYSLTR1*,Q9Y271	*CYSLTR2*,Q9NS75	*OXER1*, Q8TDS5	*FPR2*, P25090
Potency order of endogenous and other ligands	–	–	–	–	–	LXA_4_ = aspirin triggered lipoxin A4 = ATLa2 = resolvin D1 > LTC_4_ = LTD_4_ ≫ 15-deoxy-LXA4 ≫ fMet-Leu-Phe [469, 700, 702, 879, 2520]
Potency order of endogenous ligands	LTB_4_ >20-hydroxy-LTB_4_≫ 12R-HETE [2892]	12-hydroxyheptadecatrienoic acid > LTB_4_ > 12S-HETE = 12S-HPETE > 15S-HETE > 12R-HETE > 20-hydroxy-LTB_4_ [1949, 2892]	LTD_4_>LTC_4_ >LTE_4_ [1587, 2267]	LTC_4_ ≥ LTD_4_ *^≫^* LTE_4_ [985, 1916, 2521]	5-oxo-ETE, 5-oxo-C20:3, 5-oxo-ODE > 5-oxo-15-HETE > 5S-HPETE > 5S-HETE [865, 1056, 1183, 1920, 1995, 2075, 2319]	–
Endogenous agonists	–	–	–	–	–	LXA_4_ [1359], resolvin D1 [1359], aspirin-triggered resolvin D1 [1358], aspirin triggered lipoxin A4
Selective agonists	–	–	–	–	–	ATLa2 [895]
Endogenous antagonists	–	–	–	–	5-oxo-12-HETE (pIC_50_ 6.3) [2074]	–
Antagonists	–	–	pranlukast (pKi 7.1–8.8) [345, 2146], pobilukast (pKi 7.1) [347]	pranlukast (pA_2_ 7.1) [346], pobilukast (pA_2_ 6.2) [346]	S-Y048 (pIC_50_ 10.7) [2881]	–
Selective antagonists	BIIL 260 (pK_i_ 8.8) [204, 580], CP105696 (pIC_50_ 8.1) [2379], U75302 (pK_i_ 6.4) [226]	LY255283 (pIC_50_ 6–7.1) [1001, 2892]	ICI198615 (pKi 9.7) [755] – Guinea pig, zafirlukast (zafirlukast is only about 100-fold selective for CysLT1) (pKi 8.9) [345, 2146], montelukast (pKi 8.6) [2146], MK-571 (pIC50 8) [1587]	BayCysLT_2_ (pA_2_ 8.4) [350], BayCysLT_2_ (pA_2_ 8.3) [350], HAMI3379 (pIC_50_ 7.4) [2823]	–	WRWWWW (pIC_50_ 6.6) [111], t-Boc-FLFLF (pIC_50_ 4.3–6) [734, 2454, 2714]
Labelled ligands	[^3^H]LTB_4_ (Agonist) [2891], [^3^H] CGS23131 (Antagonist) (pK_d_ 7.9) [1130]	[^3^H]LTB_4_ (pK_d_ 7.6–9.7)	[^3^H]LTD_4_ (Agonist), [^3^H] ICI-198615 (Antagonist) (pK_d_ 10.6) [2219]	[^3^H]LTD_4_ (Agonist) [985]	[^3^H]5-oxo-ETE (Agonist) [1920]	[^3^H]LXA_4_ (Agonist) [700, 701]

**Comments**: The FPR2/ALX receptor (**nomenclature as agreed by the NC-IUPHAR subcommittee on Leukotriene and Lipoxin Receptors** [110]) is activated by the endogenous lipid-derived, anti-inflammatory ligands lipoxin A_4_ (LXA_4_) and 15-epi-LXA_4_ (aspirin triggered lipoxin A4, ATL). The FPR2/ALX receptor also interacts with endogenous peptide and protein ligands, such as MHC binding peptide [426] as well as annexin I (*ANXA1*, P04083) (ANXA1) and its *N*-terminal peptides [486, 2025]. In addition, a soluble hydrolytic product of protease action on the urokinase-type plasminogen activator receptor has been reported to activate the FPR2/ALX receptor [2162]. Furthermore, FPR2/ALX has been suggested to act as a receptor mediating the proinflammatory actions of the acute-phase reactant, serum amyloid A [2423, 2475]. FPR2/ALX has also been reported to be activated by resolvin D1 [1875]. The agonist activity of the lipid mediators described has been questioned [940, 2059], which may derive from batch-to-batch differences, partial agonism or biased agonism. Results from Cooray *et al*. (2013) [486] have addressed this issue and the role of homodimers and heterodimers in intracellular signaling. ATL-induced conformational changes of recombinant human ALX was demonstrated using FRET analysis [788]; ATL gives a bell-shaped concentration-response relationship, inducing maximal conformational changes of ALX at 0.1-1 nM. In addition, the crystal structure of ALX was reported at 2.8 Å resolution [409]. A receptor selective for LXB_4_ has been suggested from functional studies [74, 1603, 2203]. Note that the data for FPR2/ALX are also reproduced on the Formylpeptide receptor pages.

Oxoeicosanoid receptors (OXE, nomenclature agreed by the NC-IUPHAR subcommittee on Leukotriene receptors [281]) are activated by endogenous chemotactic eicosanoid ligands oxidised at the C-5 position, with 5-oxo-ETE the most potent agonist identified for this receptor. Initial characterization of the heterologously expressed OXE receptor suggested that polyunsaturated fatty acids, such as docosahexaenoic acid and EPA, acted as receptor antagonists [1056].

Further reading on Leukotriene receptorsBrinkC
 (2003) International Union of Pharmacology XXXVII. Nomenclature for leukotriene and lipoxin receptors. Pharmacol Rev
55: 195–22712615958
10.1124/pr.55.1.8BrinkC
 (2004) International Union of Pharmacology XLIV. Nomenclature for the oxoeicosanoid receptor. Pharmacol Rev
56: 149–5715001665
10.1124/pr.56.1.4BäckM
 (2011) International Union of Basic and Clinical Pharmacology. LXXXIV: leukotriene receptor nomenclature, distribution, and pathophysiological functions. Pharmacol Rev
63: 539–8421771892
10.1124/pr.110.004184BäckM
 (2014) Update on leukotriene, lipoxin and oxoeicosanoid receptors: IUPHAR Review 7. Br J Pharmacol
171: 3551–7424588652
10.1111/bph.12665PMC4128057LaidlawTM
 (2012) Cysteinyl leukotriene receptors, old and new; implications for asthma. Clin Exp Allergy
42: 1313–2022925317
10.1111/j.1365-2222.2012.03982.xPMC3773244

## Lysophospholipid (LPA) receptors

G protein-coupled receptors → Lysophospholipid (LPA) receptors

**Overview**: Lysophosphatidic acid (LPA) receptors (**nomenclature as agreed by the NC-IUPHAR Subcommittee on Lysophospholipid Receptors** [532, 1264, 1757, 2863]) are activated by the endogenous phospholipid LPA. The first receptor, LPA_1_, was identified as *ventricular zone gene-1* (*vzg-1*) [981], This discovery represented the beginning of the deorphanisation of members of the endothelial differentiation gene (edg) family, as other LPA and sphingosine 1-phosphate (S1P) receptors were found. Five additional LPA receptors (LPA_2,3,4,5,6_) have since been identified [1757] and their gene nomenclature codified for human *LPAR1, LPAR2, etc*. (HUGO Gene Nomenclature Committee, HGNC) and *Lpar1, Lpar2, etc*. for mice (Mouse Genome Informatics Database, MGI) to reflect species and receptor function of their corresponding proteins. The crystal structure of LPA_1_ [29, 445, 1551] and LPA_6_ [2548] are solved and indicate that LPA accesses the extracellular binding pocket, consistent with its proposed delivery via autotaxin [445]. These studies have also implicated cross-talk with endocannabinoids *via* phosphorylated intermediates that can also activate these receptors. The binding affinities to LPA_1_ of unlabeled, natural LPA and anandamide phosphate (AEAp) were measured using backscattering interferometry (pK_d_ = 9) [1758, 2150]. Utilization of this method indicated affinities that were 77-fold lower than when measured using radioactivity-based protocols [2862]. Targeted deletion of LPA receptors has clarified signalling pathways and identified physiological and pathophysiological roles. Multiple groups have independently published validation of all six LPA receptors described in these tables, and further validation was achieved using a distinct read-out via a novel TGFα “shedding* assay [1108]. LPA has been proposed to be a ligand for GPR35 [1941], supported by a study revealing that LPA modulates macrophage function through GPR35 [1234]. However chemokine (C-X-C motif) ligand 17 (CXCL17 (*CXCL17*, Q6UXB2)) is reported to be a ligand for GPR35/CXCR8 [1646]. Moreover, LPA has also been described as an agonist for the transient receptor potential (Trp) ion channels TRPV1 [1896] and TRPA1 [1303]. All of these proposed non-GPCR receptor identities require confirmation and are not currently recognized as *bona fide* LPA receptors.

**Table:** 

Nomenclature	LPA_1_ receptor	LPA_2_ receptor	LPA_3_ receptor	LPA_4_ receptor	LPA_5_ receptor	LPA_6_ receptor
HGNC, UniProt	*LPAR1*, Q92633	*LPAR2*, Q9HBW0	*LPAR3*, Q9UBY5	*LPAR4*, Q99677	*LPAR5*, Q9H1C0	*LPAR6*, P43657
Agonists	UCM-05194 [844]	–	–	–	–	–
Selective agonists	–	dodecylphosphate [2689], decyl dihydrogen phosphate [2689], GRI977143 [1296]	OMPT [956]	–	–	–
Antagonists	Ki16425 (pIC_50_ 6.6–6.9) [1937] – Mouse, VPC12249 (pKi 5.2–6.9) [987] – Mouse, VPC32179 [980]	–	VPC12249 (pKi 6.4) [987], VPC32179 [980]	–	compound 66 (pIC_50_ 7.5) [2919], compound 65 (pIC_50_ 7.2) [2919]	–
Sub/family-selective antagonists	–	–	Ki16425 (pKi 6.4) [1937]	–	–	–
Selective antagonists	BMS-986020 (pIC_50_ 8.9), AM966 (pIC_50_ 6.7–7.8) [2502], ONO-7300243 (pIC_50_ 6.8) [2555], AM095 (pIC_50_ 6–6.1) [2502]	H2L5186303 (pIC_50_ 8.1) [686, 687], UCM-14216 (pIC_50_ 5.7) [1258]	dioctanoylglycerol pyrophosphate (pKi 5.5–7) [704, 1937]	–	AS2717638 (pIC_50_ 7.4) [1830], TCLPA5 (pIC_50_ 6.1) [1350]	–

**Comments**: Ki16425 [1937], VPC12249 [987] and VPC32179 [980] have dual antagonist activity at LPA_1_ and LPA_3_ receptors. There is growing evidence for *in vivo* efficacy of these chemical antagonists in several disorders, including fetal hydrocephalus [2909], fetal hypoxia [999], lung fibrosis [1932], systemic sclerosis [1932] and atherosclerosis progression [1360]. LPA_2_ selective antagonist SAR100842 [1448], and LPA_1_ selective agonist UCM-05194 [844], are proposed for therapy of systemic sclerosis and neuropathic pain, respectively. The LPA_2_ selective agonist, GRI977143, shows efficacy in an animal model of multiple sclerosis [2301]. The LPA_5_ selective antagonist, AS2717638, is effective in pain models [1233]. Antidepressants, amitriptyline, clomipramine, and mianserin, are reported to show profibrotic responses *via* LPA_1_ [1951].

Further reading on Lysophospholipid (LPA) receptorsChunJ
 (2010) International Union of Basic and Clinical Pharmacology. LXXVIII. Lysophospholipid receptor nomenclature. Pharmacol Rev
62: 579–8721079037
10.1124/pr.110.003111PMC2993255KiharaY
 (2014) Lysophospholipid receptor nomenclature review: IUPHAR Review 8. Br J Pharmacol
171: 3575–9424602016
10.1111/bph.12678PMC4128058MizunoH
 (2020) Druggable Lipid GPCRs: Past, Present, and Prospects. Adv Exp Med Biol
1274: 223–25832894513
10.1007/978-3-030-50621-6_10YungYC
 (2015) Lysophosphatidic Acid signaling in the nervous system. Neuron
85: 669–8225695267
10.1016/j.neuron.2015.01.009PMC4400838

## Lysophospholipid (S1P) receptors

G protein-coupled receptors → Lysophospholipid (S1P) receptors

**Overview**: Sphingosine 1-phosphate (S1P) receptors (**nomenclature as agreed by the NC-IUPHAR Subcommittee on Lysophospholipid receptors** [1264]) are activated by the endogenous lipid sphingosine 1-phosphate (S1P). Originally cloned as orphan members of the endothelial differentiation gene (*edg*) family [208, 1757], the receptors are currently designated as S1P_1_R through S1P_5_R [208, 1026, 1757]. Their gene nomenclature has been codified as human *S1PR1, S1PR2, etc*. (HUGO Gene Nomenclature Committee, HGNC) and *S1pr1, S1pr2, etc*. for mice (Mouse Genome Informatics Database, MGI) to reflect species and receptor function. All S1P receptors (S1PRs) have been knocked-out in mice constitutively and in some cases, conditionally.

S1PRs, particularly S1P_1_, are expressed throughout all mammalian organ systems. Ligand delivery occurs *via* two known carriers (or “chaperones”): albumin and HDL-bound apolipoprotein M (ApoM), the latter of which elicits biased agonist signaling by S1P_1_ in multiple cell types [210, 758]. The five S1PRs, two chaperones, and active cellular metabolism have complicated analyses of receptor ligand binding in native systems.

Signaling pathways and physiological roles have been characterized through radioligand binding in heterologous expression systems, targeted deletion of the different S1PRs, and most recently, mouse models that report *in vivo* S1P_1_R activation [1334, 1335]. The structures of S1P_1_ [941, 1551, 2847, 2903], S1P_2_ [399], S1P_3_[1610, 2939], and S1P_5_ [1586, 2906] are solved, and confirmed aspects of ligand binding, specificity, and receptor activation, determined previously through biochemical and genetic studies [209, 941]. Fingolimod (FTY720), the first FDA-approved drug to target any of the lysophospholipid receptors, binds as a phosphorylated metabolite to four of the five S1PRs, and was the first oral therapy for multiple sclerosis (MS) [457]. Second-generation S1PR modulators siponimod, ozanimod, and ponesimod that target S1P_1_ and S1P_5_ are also FDA approved for the treatment of various MS forms [208, 1757]. In 2021, ozanimod became the first S1PR modulator to be FDA approved for the treatment of ulcerative colitis [2258]. The mechanisms of action of fingolimod and other S1PR-modulating drugs now in development include binding S1PRs in multiple organ systems, *e.g*., immune and nervous systems, although the precise nature of their receptor interactions requires clarification [474, 882, 883, 2087].

**Table:** 

Nomenclature	S1P_1_ receptor	S1P_2_ receptor	S1P_3_ receptor	S1P_4_ receptor	S1P_5_ receptor
HGNC, UniProt	*S1PR1*, P21453	*S1PR2*, O95136	*S1PR3*, Q99500	*S1PR4*, O95977	*S1PR5*, Q9H228
Potency order of endogenous ligands	sphingosine 1-phosphate > dihydrosphingosine 1-phosphate [58, 1943]	sphingosine 1-phosphate > dihydrosphingosine 1-phosphate [58, 1943]	sphingosine 1-phosphate > dihydrosphingosine 1-phosphate [1943]	sphingosine 1-phosphate > dihydrosphingosine 1-phosphate [2652]	sphingosine 1-phosphate > dihydrosphingosine 1-phosphate [1104]
Agonists	fingolimod-phosphate [282, 716], siponimod [827, 1977], BMS-986166 (Partial agonist) [822], BMS-986104 derivative 12 (Biased agonist) [821], BMS-986104 derivative 24 (Biased agonist) [821], etrasimod [325], SAR247799 (Biased agonist) [2062], ST-2191 [2455], ST-1478 [1106], ST-1505 [1106]	S1P d20:1 (Partial agonist) [2700]	fingolimod-phosphate [282, 716], fingolimod-phosphate [282, 716]	fingolimod-phosphate [282, 716, 1978, 2263, 2851], etrasimod [324, 325]	fingolimod-phosphate [282, 716, 1978], siponimod [783, 807, 2639], etrasimod [325]
Selective agonists	RP-001 [331], cenerimod [2043], CYM5442 [843], ponesimod [228], SEW2871 [2263] – Mouse	–	CYM-5541 [1174]	CYM-50308 [2640], SLB736 [1049]	A-971432 [575, 1031]
Antagonists	VPC23019 (pKi 7.9) [538], VPC03090-P (pKi 7.6–7.7) [1248], VPC44116 (pIC50 7.6) [717]	–	VPC44116 (pKi 6.5) [717], VPC23019 (pKi 5.9) [538]	–	–
Selective antagonists	NIBR-0213 (pIC_50_ 8.6) [2112], W146 (pKi 7.1) [2264]	JTE-013 (pIC_50_ 7.8) [1960]	TY-52156 (pKi 7) [1831]	CYM-50358 (pIC_50_ 7.6) [371, 890]	compound 15 (pIC_50_ 10) [1589]

**Comments**: The FDA-approved immunomodulator fingolimod (FTY720) is phosphorylated *in vivo* [37] to generate an agonist with activity at S1P_1_, S1P_3_, S1P_4_ and S1P_5_ receptors [282, 1634]. Many of the physiological consequences of fingolimod-phosphate administration, as well as those of other currently described S1P_1_ agonists, may involve functional antagonism *via* ubiquitination and subsequent degradation of S1P_1_ [208, 1958]. Additionally, receptor specificities of the different compounds may depend on the functional assay system utilized and from which species the receptor sequence originated.

Further reading on Lysophospholipid (S1P) receptorsCartierA
 (2019) Sphingosine 1-phosphate: Lipid signaling in pathology and therapy. Science
366:10.1126/science.aar5551PMC766110331624181ChewWS
 (2016) To fingolimod and beyond: The rich pipeline of drug candidates that target S1P signaling. Pharmacol Res
113: 521–53227663260
10.1016/j.phrs.2016.09.025ChunJ
 (2010) International Union of Basic and Clinical Pharmacology. LXXVIII. Lysophospholipid receptor nomenclature. Pharmacol Rev
62: 579–8721079037
10.1124/pr.110.003111PMC2993255PyneNJ
 (2017) Sphingosine 1-Phosphate Receptor 1 Signaling in Mammalian Cells. Molecules
22:10.3390/molecules22030344PMC615526328241498RosenH
 (2013) Sphingosine-1-phosphate and its receptors: structure, signaling, and influence. Annu Rev Biochem
82: 637–6223527695
10.1146/annurev-biochem-062411-130916YanagidaK
 (2017) Vascular and Immunobiology of the Circulatory Sphingosine 1-Phosphate Gradient. Annu Rev Physiol
79: 67–9127813829
10.1146/annurev-physiol-021014-071635PMC5500220

## Melanin-concentrating hormone receptors

G protein-coupled receptors → Melanin-concentrating hormone receptors

**Overview**: Melanin-concentrating hormone (MCH) receptors (**provisional nomenclature as recommended by NC-IUPHAR** [712]) are activated by an endogenous nonadecameric cyclic peptide identical in humans and rats (DFDML RCM LGR VYRP CWQV; mammalian MCH) generated from a precursor (*PMCH*, P20382), which also produces neuropeptide EI (*PMCH*, P20382) and neuropeptide GE (*PMCH*, P20382).

**Table:** 

Nomenclature	MCH_1_ receptor	MCH_2_ receptor
HGNC, UniProt	*MCHR1*, Q99705	*MCHR2*, Q969V1
Selective antagonists	GW803430 (pIC_50_ 9.3) [1002], SNAP-7941 (pA_2_ 9.2) [245], T-226296 (pIC_50_ 8.3) [2530], ATC0175 (pIC_50_ 7.9–8.1) [377]	–
Labelled ligands	[^125^I]S36057 (Antagonist) (pK_d_ 9.2–9.5) [91], [^125^I][Phe^13^, Tyr^19^]MCH (Agonist) [318], [^3^H]MCH (human, mouse, rat) (Agonist) [318]	–

**Comments**: The MCH_2_ receptor appears to be a non-functional pseudogene in rodents [2536].

Further reading on Melanin-concentrating hormone receptorsChungS
 (2011) Recent updates on the melanin-concentrating hormone (MCH) and its receptor system: lessons from MCH1R antagonists. J Mol Neurosci
43: 115–2120582487
10.1007/s12031-010-9411-4PMC3018593EberleAN
 (2010) Cellular models for the study of the pharmacology and signaling of melanin-concentrating hormone receptors. J Recept Signal Transduct Res
30: 385–40221083507
10.3109/10799893.2010.524223FoordSM
 (2005) International Union of Pharmacology. XLVI. G protein-coupled receptor list. Pharmacol Rev
57: 279–8815914470
10.1124/pr.57.2.5TakaseK
 (2014) Meta-analysis of melanin-concentrating hormone signaling-deficient mice on behavioral and metabolic phenotypes. PLoS ONE
9: e9996124924345
10.1371/journal.pone.0099961PMC4055708

## Melanocortin receptors

G protein-coupled receptors → Melanocortin receptors

**Overview**: Melanocortin receptors (**provisional nomenclature as recommended by NC-IUPHAR** [712]) are activated by members of the melanocortin family (α-MSH (*POMC*, P01189), β-MSH (*POMC*, P01189) and γ-MSH (*POMC*, P01189) forms; δ form is not found in mammals) and adrenocorticotrophin (ACTH (*POMC*, P01189)). Endogenous antagonists include agouti (*ASIP*,P42127) and agouti-related protein (*AGRP*, O00253). ACTH(1–24) was approved by the US FDA as a diagnostic agent for adrenal function test. Setmelanotide was approved by the US FDA for weight management in patients with POMC, PCSK1 or LEPR defiency, bremelanotide was approved by the US FDA for generalized hypoactive sexual desire disorder in premenopausal women, and NDP-MSH (afamelanotide) was approved by the EMA for the treatment of erythropoietic protoporphyria. Several synthetic melanocortin receptor agonists are under clinical development.

**Table:** 

Nomenclature	MC_1_ receptor	MC_2_ receptor	MC_3_ receptor	MC_4_ receptor	MC_5_ receptor
HGNC, UniProt	*MC1R*,Q01726	*MC2R*,Q01718	*MC3R*,P41968	*MC4R*, P32245	*MC5R*, P33032
Potency order of endogenous agonists	α-MSH (*POMC*, P01189) > β-MSH (*POMC*, P01189) > ACTH (*POMC*, P01189), γ-MSH (*POMC*, P01189)	ACTH (*POMC*,P01189)	γ-MSH (*POMC*,P01189), β-MSH (*POMC*,P01189) > ACTH (*POMC*, P01189), α-MSH (*POMC*,P01189)	*β*-MSH (*POMC*, P01189) > α-MSH (*POMC*, P01189), ACTH (*POMC*, P01189) > γ-MSH (*POMC*, P01189)	α-MSH (*POMC*, P01189) > β-MSH (POMC, P01189) > ACTH (*POMC*, P01189) > γ-MSH (*POMC*, P01189)
Selective agonists	–	corticotropin zinc hydroxide	[D-Trp^8^]γ-MSH [874]	THIQ [2328], setmelanotide [468, 1380]	–
Antagonists	–	–	PG-106 (pIC_50_ 6.7) [875]	–	–
Selective antagonists	–	–	–	MBP10 (pIC_50_ 10) [159], HS014 (pKi 8.5) [2296]	–
Labelled ligands	[^125^I]NDP-MSH (Agonist) [1338]	[^125^I]ACTH-(1-24) (Agonist)	[^125^I]NDP-MSH (Agonist) [1338], [^125^I]SHU9119 (Antagonist) [1891]	[^125^I]SHU9119 (Antagonist) (pK_d_ 9.2) [1891], [^125^I]NDP-MSH (Agonist) [1338, 2295]	[^125^I]NDP-MSH (Agonist) [1338]

**Comments**: Polymorphisms of the MC_1_ receptor have been linked to variations in skin pigmentation. Defects of the MC_2_ receptor underlie familial glucocorticoid deficiency. Polymorphisms of the MC_4_ receptor have been linked to obesity [376, 681].

Further reading on Melanocortin receptorsCarusoV
 (2014) Synaptic changes induced by melanocortin signalling. Nat Rev Neurosci
15: 98–11024588018
10.1038/nrn3657FoordSM
 (2005) International Union of Pharmacology. XLVI. G protein-coupled receptor list. Pharmacol Rev
57: 279–8815914470
10.1124/pr.57.2.5RenquistBJ
 (2011) Physiological roles of the melanocortin MC_3_ receptor. Eur J Pharmacol
660: 13–2021211527
10.1016/j.ejphar.2010.12.025PMC3095771

## Melatonin receptors

G protein-coupled receptors → Melatonin receptors

**Overview**: Melatonin receptors (**nomenclature as agreed by the NC-IUPHAR Subcommittee on Melatonin Receptors** [618]) are activated by the endogenous ligands melatonin and clinically used drugs like ramelteon, agomelatine and tasimelteon.

**Table:** 

Nomenclature	MT_1_ receptor	MT_2_ receptor
HGNC, UniProt	*MTNR1A*, P48039	*MTNR1B*, P49286
Endogenous agonists	melatonin [92, 617, 619]	melatonin [92, 617, 619]
Agonists	ramelteon [1224], agomelatine [92, 181], tasimelteon [2123, 2663]	agomelatine [92, 181], tasimelteon [2123, 2663], ramelteon [1224, 2148]
Selective agonists	–	UCM1014 [2437], IIK7 [682, 2480], 5-methoxy-luzindole (Partial agonist) [619]
Selective antagonists	–	4P-PDOT (pK_i_ 8.8–9.4) [92, 619, 620], K185 (pK_i_ 9.3) [682, 2480], DH97 (pK_i_ 8) [2554]
Labelled ligands	[^125^I]SD6 (Agonist) [1463], 2-[^125^I]melatonin (Agonist) [92, 619], [^3^H] melatonin (Agonist) [300]	[^125^I]SD6 (Agonist) [1463], 2-[^125^I]melatonin (Agonist) [92, 619], [^125^I]DIV880 (Agonist, Partial agonist) [1463], [^3^H]melatonin (Agonist) [300]

**Comments**: Melatonin, 2-iodo-melatonin, agomelatine, GR 196429, LY 156735 and ramelteon [1224] are nonselective agonists for MT_1_ and MT_2_ receptors. (−)-AMMTC displays an ~400-fold greater agonist potency than (+)-AMMTC at rat MT_1_ receptors (see AMMTC for structure) [2586]. Luzindole is an MT_1_/MT_2_ non-selective competitive melatonin receptor antagonist with about 15-25 fold selectivity for the MT_2_ receptor [620]. MT_1_/MT_2_ heterodimers present different pharmacological profiles from MT_1_ and MT_2_ receptors [101].

The *MT_3_* binding site of hamster brain and peripheral tissues such as kidney and testis, also termed the ML_2_ receptor, binds selectively 2-iodo-[^125^I]5MCA-NAT [1765]. Pharmacological in vestigations of *MT_3_* binding sites have primarily been conducted in hamster tissues. At this site, The endogenous ligand N-ace-tylserotonin [640, 1573, 1765, 2063] and 5MCA-NAT [2063] appear to function as agonists, while prazosin [1573] functions as an antagonist. The *MT_3_* binding site of hamster kidney was also identified as the hamster homologue of human quinone reductase 2 (*NQO2*, P16083 [1913, 1914]). The *MT_3_* binding site activated by 5MCA-NAT in eye ciliary body is positively coupled to adenylyl cyclase and regulates chloride secretion [1081]. *Xenopus* melanophores and chick brain express a distinct receptor (x420, P49219; c346, P49288, initially termed Mel_1C_) coupled to the G_i/o_ family of G proteins, for which GPR50 has recently been suggested to be a mammalian counterpart [623] although melatonin does not bind to GPR50 receptors. Several variants of the *MTNR1B* gene have been associated with increased type 2 diabetes risk [1217].

Further reading on Melatonin receptorsBoutinJA
 (2020) Melatonin receptor ligands: A pharmaco-chemical perspective. J Pineal Res
69: e1267232531076
10.1111/jpi.12672CeconE
 (2018) Melatonin receptors: molecular pharmacology and signalling in the context of system bias. Br J Pharmacol
175: 3263–328028707298
10.1111/bph.13950PMC6057902DubocovichML
 (2010) International Union of Basic and Clinical Pharmacology. LXXV. Nomenclature, classification, and pharmacology of G protein-coupled melatonin receptors. Pharmacol Rev
62: 343–8020605968
10.1124/pr.110.002832PMC2964901JockersR
 (2016) Update on melatonin receptors: IUPHAR Review 20. Br J Pharmacol
173: 2702–2527314810
10.1111/bph.13536PMC4995287KaramitriA
 (2019) Melatonin in type 2 diabetes mellitus and obesity. Nat Rev Endocrinol
15: 105–12530531911
10.1038/s41574-018-0130-1LiuJ
 (2016) MT1 and MT2 Melatonin Receptors: A Therapeutic Perspective. Annu Rev Pharmacol Toxicol
56: 361–8326514204
10.1146/annurev-pharmtox-010814-124742PMC5091650

## Metabotropic glutamate receptors

G protein-coupled receptors → Metabotropic glutamate receptors

**Overview**: Metabotropic glutamate (mGlu) receptors (**nomenclature as agreed by the NC-IUPHAR Subcommittee on Metabotropic Glutamate Receptors** [2302]) are a family of G protein-coupled receptors activated by the neurotransmitter glutamate [872]. The mGlu family is composed of eight members (named mGlu1 to mGlu_8_) which are divided in three groups based on similarities of agonist pharmacology, primary sequence and G protein coupling to effector: Group-I (mGlu_1_ and mGlu_5_), Group-II (mGlu_2_ and mGlu_3_) and Group-III (mGlu_4_, mGlu_6_, mGlu_7_ and mGlu_8_) (see Further reading).

Structurally, mGlu are composed of three juxtaposed domains: a core G protein-activating seven-transmembrane domain (TM), common to all GPCRs, is linked *via* a rigid cysteine-rich domain (CRD) to the Venus Flytrap domain (VFTD), a large bi-lobed extracellular domain where glutamate binds. mGlu form constitutive dimers, cross-linked by a disulfide bridge. The structures of the VFTD of mGlu_1_, mGlu_2_, mGlu_3_, mGlu_5_ and mGlu_7_ have been solved [1383, 1776, 1838, 2614]. The structure of the 7 transmembrane (TM) domains of both mGlu1 and mGlu5 have been solved, and confirm a general helical organisation similar to that of other GPCRs, although the helices appear more compacted [450, 605, 2817]. Recent advances in cryo-electron microscopy have provided structures of full-length mGlu receptor homodimers [1317, 1527] and heterodimers [615]. Studies have revealed the possible formation of heterodimers between either group-I receptors, or within and between group-II and -III receptors [609]. First characterised in transfected cells, co-localisation and specific pharmacological properties suggest the existence of such heterodimers in the brain [907, 1528, 1709, 1790, 1907, 2890]. Beyond heteromerisation with other mGlu receptor subtypes, increasing evidence suggests mGlu receptors form heteromers and larger order complexes with class A GPCRs (reviewed in [872]).

The endogenous ligands of mGlu are L-glutamic acid, L-serine-O-phosphate, N-acetylaspartylglutamate (NAAG) and L-cysteine sulphinic acid. Group-I mGlu receptors may be activated by 3,5-DHPG and (S)-3HPG [261] and antagonised by (S)-hexylhomoibotenic acid [1606]. Group-II mGlu receptors may be activated by LY389795 [1777], LY379268 [1777], eglumegad [2303, 2821], DCG-IV and (2R,3R)-APDC [2304], and antagonised by eGlu [1147] and LY307452 [664, 2768]. Group-III mGlu receptors may be activated by L-AP4 and (R, S)-4-PPG [778]. An example of an antagonist selective for mGlu receptors is LY341495, which blocks mGlu_2_ and mGlu_3_ at low nanomolar concentrations, mGlu_8_ at high nanomolar concentrations, and mGlu_4_, mGlu_5_, and mGlu_7_ in the micromolar range [1287]. In addition to orthosteric ligands that directly interact with the glutamate recognition site, allosteric modulators that bind within the TM domain have been described. Negative allosteric modulators are listed separately. The positive allosteric modulators most often act as ‘potentiators’ of an orthosteric agonist response, without significantly activating the receptor in the absence of agonist.

**Table:** 

Nomenclature	mGlu_1_ receptor	mGlu_2_ receptor	mGlu_3_ receptor	mGlu_4_ receptor	mGlu_5_ receptor
HGNC, UniProt	*GRM1*, Q13255	*GRM2*, Q14416	*GRM3*, Q14832	*GRM4*, Q14833	*GRM5*, P41594
Endogenous agonists	L-glutamic acid [2049]	L-glutamic acid [2049]	L-glutamic acid [2049], NAAG [2316]	L-serine-O-phosphate [2821], L-glutamic acid [2049]	L-glutamic acid [2049]
Agonists	–	–	–	L-AP4 [2821]	–
Selective agonists	–	–	–	LSP4-2022 [856]	(S)-(+)-CBPG (Partial agonist) [1638] – Rat, CHPG [1837]
Antagonists	LY367385 (pIC_50_ 5.1) [467]	–	–	MAP4 (pKi 4.6) [928] – Rat	–
Selective antagonists	3-MATIDA (pIC_50_ 5.2) [1808] – Rat, (S)-(+)-CBPG (pIC_50_ 4.2) [1638] – Rat, (S)-TBPG (pIC_50_ 4.2) [489] – Rat, AIDA (pA_2_ 4.2) [1809]	PCCG-4 (pIC_50_ 5.1) [2012] – Rat	–	–	ACDPP (pIC_50_ 6.9) [240]
Allosteric modulators (Positive)	–	CBiPES (pEC_50_ 7) [1180], 4-MPPTS (pIC_50_ 5.8) [131, 1179, 1180, 2288]	–	SIB-1893 (obtained in the presence of L-AP4) (pEC_50_ 6.3–6.8) [1669], MPEP (obtained in the presence of L-AP4) (pEC_50_ 6.3–6.6) [1669], PHCCC (obtained in the presence of L-AP4) (pEC_50_ 4.5) [1621]	CDPPB (pEC_50_ 7.6–8) [1289, 1532]
Allosteric modulators (Negative)	–	–	MNI-137 (pIC_50_ 7.7) [993] – Rat, VU0650786 (pIC_50_ 6.4) [654]	–	alloswitch-1 (pIC_50_ 8.1) [2057] – Rat, MTEP (p*K*_i_ 7.8) [292], MPEP (pIC_50_ 7.4–7.7) [777, 779], fenobam (pIC_50_ 7.2) [2069]
Selective allosteric modulators	BAY 367620 (Negative) (pK_i_ 9.5) [354] – Rat, JNJ16259685 (Negative) (pIC_50_ 8.9) [1422], Ro01-6128 (Positive) (pK_i_ 7.5–7.7) [1312] – Rat, LY456236 (Negative) (pIC_50_ 6.9) [437], CPCCOEt (Negative) (pIC_50_ 5.2–5.8) [1535]	Ro64-5229 (Negative) (pIC_50_ 7) [1330] – Rat, biphenylindanone A (Positive) (pEC_50_ 7) [241]	ML337 (Negative) (pIC_50_ 6.2) [2765] – Rat	VU0361737 (Positive) (pEC_50_ 6.6) [653], VU0155041 (Positive) (pEC_50_ 6.1) [1906]	VU0409551 (Positive) (p*K*_B_ 7.1) [2205], VU0360172 (Positive) (p*K*_B_ 6.6–7) [873, 2196]

**Table:** 

Nomenclature	mGlu_6_ receptor	mGlu_7_ receptor	mGlu_8_ receptor
HGNC, UniProt	*GRM6*, O15303	*GRM7*, Q14831	*GRM8*, O00222
Endogenous agonists	L-glutamic acid [2049]	L-glutamic acid [2049]	L-serine-O-phosphate [1626, 2821], L-glutamic acid [2049]
Agonists	–	LSP4-2022 [856], L-serine-O-phosphate [2821], L-AP4 [2821]	(S)-3,4-DCPG [2572], L-AP4 [1626]
Selective agonists	1-benzyl-APDC [2617] – Rat, homo-AMPA [270]	–	–
Antagonists	MAP4 (pIC_50_ 3.5) [2046] – Rat, THPG [2577]	–	MPPG (pIC_50_ 4.3) [2821]
Allosteric modulators (Positive)	–	AMN082 (pEC_50_ 6.5–6.8) [1752]	VU0422288 (p*K*_B_ 6.7) [1146], VU0155094 (p*K*_B_ 5) [1146]
Allosteric modulators (Negative)	–	MMPIP (pIC_50_ 6.1–7.6) [1905, 2497] – Rat, ADX71743 (pIC_50_ 7.2) [1203], XAP044 (pIC_50_ 5.6) [789]	–

**Comments**: The activity of NAAG as an agonist at mGlu_3_ receptors was questioned on the basis of contamination with glutamate [441, 736], but this has been refuted [1864]. Radioligand binding using a variety of radioligands has been conducted on recombinant receptors (for example, [^3^H]R214127 [1421] and [^3^H]YM298198 [1322] at mGlu_1_ receptors and [^3^H] M-MPEP [777] and [^3^H]methoxymethyl-MTEP [60] at mGlu_5_ receptors; [^3^H]LY341495 and [^3^H]eglumegad for mGlu_2_ and mGlu_3_ receptors [1178, 2316]). Although a number of radioligands have been used to examine binding in native tissues, correlation with individual subtypes is limited. Many pharmacological agents have not been fully tested across all known subtypes of mGlu receptors and may have unappreciated biased or neutral activity at other subtypes [992]. Potential differences linked to the species (*e.g*. human *versus* rat or mouse) of the receptors and the receptor splice variants are generally not known. The influence of receptor expression level on pharmacology and selectivity has not been controlled for in most studies, particularly those involving functional assays of receptor coupling.

(S)-(+)-CBPG is an antagonist at mGlu_1_, but is an agonist (albeit of reduced efficacy) at mGlu_5_ receptors. DCG-IV also exhibits agonist activity at NMDA glutamate receptors [2644], and is an antagonist at all Group-III mGluRs with an IC_50_ of 30μM. A potential novel metabotropic glutamate receptor coupled to phosphoinositide turnover has been observed in rat brain; it is activated by 4-methylhomoibotenic acid (ineffective as an agonist at recombinant Group I metabotropic glutamate receptors), but is resistant to LY341495 [458]. There are also reports of a distinct metabotropic glutamate receptor coupled to phospholipase D in rat brain, which does not readily fit into the current classification [1305, 2010]

A related class C receptor composed of two distinct subunits, T1R1 + T1R3 is also activated by glutamate and is responsible for umami taste detection.

All selective antagonists at metabotropic glutamate receptors are competitive.

Further reading on Metabotropic glutamate receptorsFerragutiF
 (2006) Metabotropic glutamate receptors. Cell Tissue Res
326: 483–50416847639
10.1007/s00441-006-0266-5GregoryKJ
 (2021) International Union of Basic and Clinical Pharmacology. CXI. Pharmacology, Signaling, and Physiology of Metabotropic Glutamate Receptors. Pharmacol Rev
73: 521–56933361406
10.1124/pr.119.019133NicolettiF
 (2011) Metabotropic glutamate receptors: from the workbench to the bedside. Neuropharmacology
60: 1017–4121036182
10.1016/j.neuropharm.2010.10.022PMC3787883NiswenderCM
 (2010) Metabotropic glutamate receptors: physiology, pharmacology, and disease. Annu Rev Pharmacol Toxicol
50: 295–32220055706
10.1146/annurev.pharmtox.011008.145533PMC2904507PinJP
 (2016) Organization and functions of mGlu and GABAB receptor complexes. Nature
540: 60–6827905440
10.1038/nature20566RondardP
 (2011) The complexity of their activation mechanism opens new possibilities for the modulation of mGlu and GABAB class C G protein-coupled receptors. Neuropharmacology
60: 82–9220713070
10.1016/j.neuropharm.2010.08.009

## Motilin receptor

G protein-coupled receptors → Motilin receptor

**Overview**: Motilin receptors (**provisional nomenclature**) are activated by motilin (*MLN*, P12872), a 22 amino-acid peptide derived from a precursor (*MLN*, P12872), which may also generate a motilin-associated peptide (*MLN*, P12872). There are significant species differences in the structure of motilin and its receptor, and in the functions of motilin. In humans and large mammals such as dog, activation of these receptors by motilin released from endocrine cells in the duodenal mucosa during fasting, induces propulsive phase III movements. This activity is associated with promoting hunger in humans. In humans and other mammals drugs and other non-peptide compounds which activate the motilin receptor may generate a more long-lasting ability to increase cholinergic activity within the upper gut, to promote upper gastrointestinal motility; this activity is suggested to be responsible for the gastrointestinal prokinetic effects of certain macrolide antibacterials (often called motilides; *e.g*. erythromycin, azithromycin), although for many of these molecules the evidence is sparse. Relatively high doses may induce vomiting and in humans, nausea.

**Table:** 

Nomenclature	motilin receptor
HGNC, UniProt	*MLNR*, O43193
Endogenous agonists	motilin (*MLN*,P12872) [494, 1674, 1675, 1676]
Agonists	alemcinal [2566], erythromycin [683, 2566], azithromycin [286]
Selective agonists	camicinal [139, 2262], mitemcinal [1319, 2519] – Rabbit
Selective antagonists	MA-2029 (pA_2_ 9.2) [2476], GM-109 (pIC_50_ 8) [945] – Rabbit
Labelled ligands	[^125^I]motilin (human) (Agonist) [683]

**Comments**: In terms of structure, the motilin receptor has closest homology with the ghrelin receptor. Thus, the human motilin receptor shares 52% overall amino acid identity with the human ghrelin receptor and 86% in the transmembrane regions [975, 2519, 2566]. However, differences between the N-terminus regions of these receptors means that their cognate peptide ligands do not readily activate each other [525, 2262]. Where studied the motilin receptor does not appear to have constitutive activity [1042]. Although not proven, the existence of biased agonism at the receptor has been suggested [1676, 1750, 2259]. A truncated 5-transmembrane structure has been identified but this is without activity when transfected into a host cell [5]. Receptor dimerisation has not been reported. It must be noted that for the complex macrolide structures, selectivity of action has often not been rigorously examined and other actions are possible (*e.g*. P2X inhibition by erythromycin; [2940]). Small molecule and selective motilin receptor agonists are now described [1495, 2262, 2773]. Significant species-dependent variations exist. Among mammals, the gene encoding the motilin percursor is absent in laboratory rodents, while the receptor appears to be a pseudogene [975, 2260]. Functions of motilin are not usually detected in rodents, although brain and other responses to motilin and macrolides continue to be reported and the mechanism of these actions is obscure. In some non-laboratory rodents (*e.g*. North American kangaroo rat (*Dipodomys*) and mouse (*Microdipodops*) a functional form of motilin may exist but the motilin receptor is non-functional [1495]. Marked differences in ligand affinities for the motilin receptor in dogs and humans may be explained by significant differences in receptor structure [2261]. Among birds, chicken (*Gallus gallus domesticus*) motilin differs from human motilin at positions 4, 7–10, and 12, and contracts avian upper gastrointestinal tissues more potently than human motilin; in rabbit duodenum, the reverse is apparent [1299]. Chicken motilin receptor has 59% sequence homology with the human motilin receptor [2850]. In chicken, motilin does not mediate phase III activity of the gastric MMC but initiates rhythmic oscillating complexes in the small intestine [2197]. Responsiveness to motilin in the ileum is highest in avian gastrointestinal tract. Among reptiles, caiman/alligator motilin is similar to avian motilin, but markedly different forms of motilin exist in turtles, anole/lizard and snake. Their activities have not been examined in reptiles. Among amphibians, a motilin-like peptide has been identified in newts but not in frogs, with a structure differing from mammalian motilin. There may be some diversity among the anuran, urodelal and gymnophional species. Although endogenous motilin is not present in frogs, human motilin caused contraction of the upper gastrointestinal tract [2932]. However, newt but not human motilin caused strong contraction of the stomach of Japanese fire belly newts [2931]. Among teleost fish, sequences for motilin peptide and motilin receptor have been identified (zebrafish, ballan wrasse, spotted sea bass) but the motilin peptides are short and the structure of motilin receptor differs from that of mammals. Zebrafish motilin activates its cognate motilin receptor but fails to cause contraction of gastrointestinal strips *in vitro*, perhaps because of low expression of the motilin receptor [1300].

Further reading on Motilin receptorKitazawaT
 (2021) Motilin Comparative Study: Structure, Distribution, Receptors, and Gastro-intestinal Motility. Front Endocrinol (Lausanne)
12: 70088434497583
10.3389/fendo.2021.700884PMC8419268SangerGJ
 (2016) Ghrelin and motilin receptors as drug targets for gastrointestinal disorders. Nat Rev Gastroenterol Hepatol
13: 38–4826392067
10.1038/nrgastro.2015.163SingaramK
 (2020) Motilin: a panoply of communications between the gut, brain, and pancreas. Expert Rev Gastroenterol Hepatol
14: 103–11131996050
10.1080/17474124.2020.1718492

## Neuromedin U receptors

G protein-coupled receptors → Neuromedin U receptors

**Overview**: Neuromedin U receptors (**provisional nomenclature as recommended by NC-IUPHAR** [712]) are activated by the endogenous 25 amino acid peptide neuromedin U (neuromedin U-25 (*NMU*, P48645), NmU-25), a peptide originally isolated from pig spinal cord [1745]. In humans, NmU-25 appears to be the sole product of a precursor gene (*NMU*, P48645) showing a broad tissue distribution, but which is expressed at highest levels in the upper gastrointestinal tract, CNS, bone marrow and fetal liver. Much shorter versions of NmU are found in some species, but not in human, and are derived at least in some instances from the proteolytic cleavage of the longer NmU. Despite species differences in NmU structure, the C-terminal region (particularly the C-terminal pentapeptide) is highly conserved and contains biological activity. Neuromedin S (neuromedin S-33 (*NMS*, Q5H8A3)) has also been identified as an endogenous agonist [1797]. NmS-33 is, as its name suggests, a 33 amino-acid product of a precursor protein derived from a single gene and contains an amidated C-terminal heptapeptide identical to NmU. NmS-33 appears to activate NMU receptors with equivalent potency to NmU-25.

**Table:** 

Nomenclature	NMU1 receptor	NMU2 receptor
HGNC, UniProt	*NMUR1*, Q9HB89	*NMUR2*, Q9GZQ4
Agonists	CPN-223 (Partial agonist) [2524]	–
Selective agonists	–	CPN-219 [2526], CPN-116 [2525]
Antagonists	–	R-PSOP (p*K*_B_ 7) [1548]
Comments	CPN-267 is a selective hexapeptidic NMU1 agonist, but the sequence is obscure.	–

**Comments**: NMU1 and NMU2 couple predominantly to G_q/11_ although there is evidence of good coupling to G_i/o_ [280, 1058, 1069]. NMU1 and NMU2 can be labelled with [^125^I]-NmU and [^125^I]-NmS (of various species, *e.g*. [1710]), BODIPYⓇ TMR-NMU or Cy3B-NMU-8 [280]. A range of radiolabelled (^125^I-), fluorescently labelled (*e.g*. Cy3, Cy5, rhodamine and FAM) and biotin labelled versions of neuromedin U-25 (*NMU*, P48645) and neuromedin S-33 (*NMS*, Q5H8A3) are now commercially available.

Further reading on Neuromedin U receptorsBrightonPJ
 (2004) Neuromedin U and its receptors: structure, function, and physiological roles. Pharmacol Rev
56: 231–4815169928
10.1124/pr.56.2.3BudhirajaS
 (2009) Neuromedin U: physiology, pharmacology and therapeutic potential. Fundam Clin Pharmacol
23: 149–5719645813
10.1111/j.1472-8206.2009.00667.xMitchellJD
 (2009) Emerging pharmacology and physiology of neuromedin U and the structurally related peptide neuromedin S. Br J Pharmacol
158: 87–10319519756
10.1111/j.1476-5381.2009.00252.xPMC2795236NovakCM. (2009) Neuromedin S and U. Endocrinology
150: 2985–719549882
10.1210/en.2009-0448PMC2703548

## Neuropeptide FF/neuropeptide AF receptors

G protein-coupled receptors → Neuropeptide FF/neuropeptide AF receptors

**Overview**: The Neuropeptide FF receptor family contains two subtypes, NPFF1 and NPFF2 (**provisional nomenclature** [712]), which exhibit high affinities for neuropeptide FF (*NPFF*, O15130) and RFamide related peptides (RFRP: precursor gene symbol NPVF, Q9HCQ7). NPFF1 is broadly distributed in the limbic system and the hypothalamus. NPFF2 is present in high density in the superficial layers of the mammalian spinal cord where it is involved in nociception and modulation of opioid central nervous system with the highest levels found in the functions.

**Table:** 

Nomenclature	NPFF1 receptor	NPFF2 receptor
HGNC, UniProt	*NPFFR1*, Q9GZQ6	*NPFFR2*, Q9Y5X5
Potency order of endogenous ligands	RFRP-1 (*NPVF*, Q9HCQ7) > RFRP-3 (*NPVF*, Q9HCQ7) > *FMRF* neuropeptide FF (*NPFF*, O15130) > neuropeptide AF (*NPFF*, O15130) > neuropeptide SF (*NPFF*, O15130), QRFP43 (43RFa) (*QRFP*, P83859), PrRP-31 (*PRLH*, P81277) [853]	neuropeptide AF (*NPFF*, O15130), neuropeptide FF (*NPFF*, O15130) > PrRP-31 (*PRLH*, P81277) > FMRF, QRFP43 (43RFa) (*QRFP*, P83859) > neuropeptide SF (*NPFF*, O15130) [853]
Endogenous agonists	neuropeptide FF (*NPFF*, O15130) [853, 854, 1768], RFRP-3 (*NPVF*, Q9HCQ7) [854, 855, 1768]	neuropeptide FF (*NPFF*, O15130) [854, 1767]
Selective agonists	–	dNPA [2217], AC263093 [1402]
Antagonists	RF9 (p*K*_i_ 7.2) [2390]	–
Selective antagonists	AC262620 (p*K*_i_ 7.7–8.1) [1402], AC262970 (p*K*_i_ 7.4–8.1) [1402]	–
Labelled ligands	[^125^I]Y-RFRP-3 (Agonist) [854], [^3^H]NPVF (Agonist) [2532], [^125^I]NPFF (Agonist) [853]	[^125^I]EYF (Agonist) [1768], [^3^H]EYF (Agonist) [2532], [^125^I]NPFF (Agonist) [853]

**Comments**: An orphan receptor *GPR83* (Q9NYM4) shows sequence similarities with NPFF1, NPFF2, PrRP and QRFP receptors. The antagonist RF9 is selective for NPFF receptors, but does not distinguish between the NPFF1 and NPFF2 subtypes (p*K*_i_ 7.1 and 7.2, respectively, [537, 2390]).

Further reading on Neuropeptide FF/neuropeptide AF receptorsMoulédousL
 (2010) Opioid-modulating properties of the neuropeptide FF system. Biofactors
36: 423–920803521
10.1002/biof.116NguyenT
 (2020) Neuropeptide FF and Its Receptors: Therapeutic Applications and Ligand Development. J Med Chem
63: 12387–1240232673481
10.1021/acs.jmedchem.0c00643PMC9590617VyasN
 (2006) Structure-activity relationships of neuropeptide FF and related peptidic and non-peptidic derivatives. Peptides
27: 990–616490282
10.1016/j.peptides.2005.07.024YangHY
 (2008) Modulatory role of neuropeptide FF system in nociception and opiate anal gesia. Neuropeptides
42: 1–1817854890
10.1016/j.npep.2007.06.004

## Neuropeptide S receptor

G protein-coupled receptors → Neuropeptide S receptor

**Overview**: The neuropeptide S receptor (NPS receptor) responds to the 20 amino-acid peptide neuropeptide S derived from a precursor (*NPS*, P0C0P6). NPS activates its receptor at low nanomolar concentrations elevating intracellular cAMP and calcium levels [2161]. Currently, some peptidic and small molecule NPS receptor antagonists are available as research tools [339, 894, 1944, 2233]. No NPS receptor ligands are currently used clinically.

**Table:** 

Nomenclature	NPS receptor
HGNC, UniProt	*NPSR1*, Q6W5P4
Endogenous agonists	neuropeptide S (*NPS*, P0C0P6) [2846]
Selective agonists	PWT1-NPS [2234] – Mouse
Selective antagonists	SHA 68 (pA_2_ 8.1) [2235] – Mouse, [^t^Bu-D-Gly^5^]NPS (pA_2_ 7.1) [894] – Mouse
Labelled ligands	[^125^I]Tyr^10^NPS (human) (Agonist) [2846]

**Comments**: Multiple single-nucleotide polymorphisms (SNP) and several splice variants have been identified in the human NPS receptor. The most interesting of these is an Asn-Ile exchange at position 107 (Ile107Asn, rs324981). The human NPS receptor Ile107Asn displayed similar binding affinity but higher NPS potency (by approx. 10-fold) than human NPS receptor Asn107 [2161]. Several epidemiological studies reported an association between the Ile107Asn receptor variant and susceptibility to panic disorders [596, 600, 1945, 2120]. The SNP Ile107Asn (rs324981) has also been linked to sleep behavior [852], inflammatory bowel disease [514], schizophrenia [1473], increased impulsivity and ADHD symptoms [1392]. Interestingly, a carboxy-terminal splice variant of human NPS receptor was found to be overexpressed in asthmatic patients [1400]. Additionally, the gain-of-function variant Tyr206His has been described in a single family where it appears to dramatically reduce total sleep time [2836].

Further reading on Neuropeptide S receptorGuerriniR
 (2010) Neurobiology, pharmacology, and medicinal chemistry of neuropeptide S and its receptor. Med Res Rev
30: 751–7719824051
10.1002/med.20180ReinscheidRK
 (2021) Pharmacology, Physiology and Genetics of the Neuropeptide S System. Pharmaceuticals (Basel)
14:10.3390/ph14050401PMC814683433922620ReinscheidRK
 (2005) Pharmacological characterization of human and murine neuropeptide s receptor variants. J Pharmacol Exp Ther
315: 1338–4516144971
10.1124/jpet.105.093427RuzzaC
 (2015) Neuropeptide S reduces mouse aggressiveness in the resident/intruder test through selective activation of the neuropeptide S receptor. Neuropharmacology
97: 1–625979487
10.1016/j.neuropharm.2015.05.002RuzzaC
 (2017) Neuropeptide S receptor ligands: a patent review (2005-2016). Expert Opin Ther Pat
27: 347–36227788040
10.1080/13543776.2017.1254195XuYL
 (2004) Neuropeptide S: a neuropeptide promoting arousal and anxiolytic-like effects. Neuron
43: 487–9715312648
10.1016/j.neuron.2004.08.005

## Neuropeptide W/neuropeptide B receptors

G protein-coupled receptors → Neuropeptide W/neuropeptide B receptors

**Overview**: The neuropeptide BW receptor 1 (NPBW1, **provisional nomenclature** [712]) is activated by two 23-amino-acid peptides, neuropeptide W (neuropeptide W-23 (*NPW*, Q8N729)) and neuropeptide B (neuropeptide B-23 (*NPB*, Q8NG41)) [744, 2367]. C-terminally extended forms of the peptides (neuropeptide W-30 (*NPW*, Q8N729) and neuropeptide B-29 (*NPB*, Q8NG41)) also activate NPBW1 [277]. Unique to both forms of neuropeptide B is the N-terminal bromination of the first tryptophan residue, and it is from this post-translational modification that the nomenclature NPB is derived. These peptides were first identified from bovine hypothalamus and therefore are classed as neuropeptides. Endogenous variants of the peptides without the N-terminal bromination, des-Br-neuropeptide B-23 (*NPB*, Q8NG41) and des-Br-neuropeptide B-29 (*NPB*, Q8NG41), were not found to be major components of bovine hypothalamic tissue extracts. The NPBW2 receptor is activated by the short and C-terminal extended forms of neuropeptide W and neuropeptide B [277].

**Table:** 

Nomenclature	NPBW1 receptor	NPBW2 receptor
HGNC, UniProt	*NPBWR1*, P48145	*NPBWR2*, P48146
Potency order of endogenous ligands	neuropeptide B-29 (*NPB*, Q8NG41) > neuropeptide B-23 (*NPB*, Q8NG41) > neuropeptide W-23 (*NPW*, Q8N729) > neuropeptide W-30 (*NPW*, Q8N729) [277]	neuropeptide W-23 (*NPW*, Q8N729) > neuropeptide W-30 (*NPW*, Q8N729) > neuropeptide B-29 (*NPB*, Q8NG41) > neuropeptide B-23 (*NPB*, Q8NG41) [277]
Selective agonists	Ava3 [1210], Ava5 [1210]	–
Labelled ligands	[^125^I]NPW-23 (human) (Agonist) [2392]	[^125^I]NPW-23 (human) (Agonist) [2367]

**Comments**: Potency measurements were conducted with heterologously-expressed receptors with a range of 0.14–0.57 nM (NPBW1) and 0.98–21 nM (NPBW2). NPBW1^−/−^ mice show changes in social behavior, suggesting that the NPBW1 pathway may have an important role in the emotional responses of social interaction [1843]. For a review of the contribution of neuropeptide B/W to social dominance, see Watanabe and Yamamoto, 2015 [2748]. It has been reported that neuropeptide W may have a key role in the gating of stressful stimuli when mice are exposed to novel environments [1815]. Two antagonists have been discovered and reported to have affinity for NPBW1, ML181 and ML250, the latter exhibiting improved selectivity (~100 fold) for NPBW1 compared to MCH1 receptors [891, 892]. Computational insights into the binding of antagonists to this receptor have also been described [2000, 2004].

Further reading on Neuropeptide W/neuropeptide B receptorsSakuraiT. (2013) NPBWR1 and NPBWR2: Implications in Energy Homeostasis, Pain, and Emotion. Front Endocrinol (Lausanne)
4: 2323515889
10.3389/fendo.2013.00023PMC3600615SinghG
 (2006) Neuropeptide B and W: neurotransmitters in an emerging G-protein-coupled receptor system. Br J Pharmacol
148: 1033–4116847439
10.1038/sj.bjp.0706825PMC1752024

## Neuropeptide Y receptors

G protein-coupled receptors → Neuropeptide Y receptors

**Overview**: Neuropeptide Y (NPY) receptors (**nomenclature as agreed by the NC-IUPHAR Subcommittee on Neuropeptide Y Receptors** [1725]) are activated by the endogenous peptides neuropeptide Y (*NPY*, P01303), neuropeptide Y-(3–36), peptide YY (*PYY*, P10082), PYY-(3-36) and pancreatic polypeptide (*PPY*, P01298) (PP). The receptor originally identified as the Y3 receptor has been identified as the CXCR4 chemokine recepter (originally named LESTR, [1558]). The y6 receptor is a functional gene product in mouse, absent in rat, but contains a frame-shift mutation in primates producing a truncated non-functional gene [870]. Three-dimensional structures have been determined for subtype active receptors Y_1_, Y_2_ and Y_4_ [1211, 2544] and inactive antagonist bound Y_1_ and Y_2_ receptors [2543, 2874]. Many of the agonists exhibit differing degrees of selectivity dependent on the species examined. For example, the potency of PP is greater at the rat Y_4_ receptor than at the human receptor [660]. In addition, many agonists lack selectivity for individual subtypes, but can exhibit comparable potency against pairs of NPY receptor subtypes, or have not been examined for activity at all subtypes. [^125^I]-PYY or [^125^I]-NPY can be used to label Y_1_, Y_2_, Y_5_ and y_6_ subtypes non-selectively, while [^125^I] [cPP(1–7), NPY(19–23), Ala^31^, Aib^32^, Gln^34^]hPP may be used to label Y_5_ receptors preferentially (note that cPP denotes chicken peptide sequence and hPP is the human sequence).

**Table:** 

Nomenclature	Y_1_ receptor	Y_2_ receptor	Y_4_ receptor	Y_5_ receptor	y_6_ receptor
HGNC, UniProt	*NPY1R*, P25929	*NPY2R*, P49146	*NPY4R*, P50391	*NPY5R*, Q15761	NPY6R, Q99463
Potency order of endogenous ligands	neuropeptide Y = peptide YY ≫ pancreatic polypeptide	peptide YY = peptide YY(3–36) = neuropeptide Y = neuropeptide Y(3–36) ≫ pancreatic polypeptide	pancreatic polypeptide ≫ neuropeptide Y = peptide YY	neuropeptide Y > peptide YY > pancreatic polypeptide	neuropeptide Y = peptide YY > pancreatic polypeptide
Endogenous agonists	neuropeptide Y (*NPY*, P01303), peptide YY (*PYY*, P10082)	PYY-(3-36) (*PYY*, P10082) [790, 805], neuropeptide Y (*NPY*, P01303), neuropeptide Y-(3-36) (*NPY*, P01303), peptide YY (*PYY*, P10082)	pancreatic polypeptide (*PPY*, P01298) [129, 1578, 2604, 2858]	–	–
Agonists	[Leu^31^, Pro^34^]NPY [502], [Leu^31^-, Pro^34^]PYY (human), [Pro^34^]NPY, [Pro^34^]PYY (human)	–	–	–	–
Selective agonists	–	–	–	[Ala^31^, Aib^32^]NPY (pig) [330]	–
Selective antagonists	BIBO3304 (pIC_50_ 9.5) [2783], BIBP3226 (p*K*_i_ 8.1–9.3) [603, 2784]	BIIE0246 (pIC_50_ 8.5) [601], JNJ-5207787 (pIC_50_ 6.9–7.1) [235]	–	L-152,804 (p*K*_i_ 7.6) [1209]	–
Selective allosteric modulators	–	–	(S)-VU0637120 (Negative) (pIC_50_ 5.6) [2285], tert-butylphenoxycyclohexanol (Positive) [2311]	–	–
Labelled ligands	[^3^H]BIBP3226 (Antagonist) (p*K*_d_ 8.7), [^125^I][Leu^31^, Pro^34^]NPY (Agonist)	[^125^I]PYY-(3-36) (human) (Agonist)	[^125^I]PP (human) (Agonist)	[^125^I][cPP(1-7), NPY(19-23), Ala^31^, Aib^32^, Gln^34^]hPP (Agonist) [626] – Rat	–
Comments	Note that Pro^34^-containing NPY and PYY can also bind Y_4_ and Y_5_ receptors, so strictly speaking are not selective, but are the’preferred’ agonists.	–	–	–	–

**Comments**: The Y_1_ agonists indicated are selective relative to Y_2_ receptors. BIBP3226 is selective relative to Y_2_, Y_4_ and Y_5_ receptors [804]. NPY-(13-36) is Y_2_ selective relative to Y_1_ and Y_5_ receptors. PYY-(3-36) is Y_2_ selective relative to Y_1_ receptors. Note that Pro34-containing NPY and PYY can also bind Y_4_ and Y_5_, thus they are selective only relative to Y_2_. The y_6_ receptor is a pseudogene in humans, but is functional in mouse, rabbit and some other mammals.

Further reading on Neuropeptide Y receptorsBowersME
 (2012) Neuropeptide regulation of fear and anxiety: Implications of cholecystokinin, endogenous opioids, and neuropeptide Y. Physiol Behav
107: 699–71022429904
10.1016/j.physbeh.2012.03.004PMC3532931MichelMC
 (1998) XVI. International Union of Pharmacology recommendations for the nomenclature of neuropeptide Y, peptide YY, and pancreatic polypeptide receptors. Pharmacol Rev
50: 143–509549761
Pedragosa-BadiaX
 (2013) Neuropeptide Y receptors: how to get subtype selectivity. Front Endocrinol (Lausanne)
4: 523382728
10.3389/fendo.2013.00005PMC3563083ZhangL
 (2011) The neuropeptide Y system: pathophysiological and therapeutic implications in obesity and cancer. Pharmacol Ther
131: 91–11321439311
10.1016/j.pharmthera.2011.03.011

## Neurotensin receptors

G protein-coupled receptors → Neurotensin receptors

**Overview**: Neurotensin receptors (**nomenclature as recommended by NC-IUPHAR** [712]) are activated by the endogenous tridecapeptide neurotensin (pGlu-Leu-Tyr-Glu-Asn-Lys-Pro-Arg-Arg-Pro-Tyr-Ile-Leu) derived from a precursor (*NTS*, 30990), which also generates neuromedin N, an agonist at the NTS_2_ receptor. [^3^H]neurotensin (human, mouse, rat) and [^125^I]neurotensin (human, mouse, rat) may be used to label NTS_1_ and NTS_2_ receptors at 0.1-0.3 and 3–5 nM concentrations respectively.

**Table:** 

Nomenclature	NTS_1_ receptor	NTS_2_ receptor
HGNC, UniProt	*NTSR1*, P30989	*NTSR2*, O95665
Potency order of endogenous ligands	neurotensin (*NTS*, P30990) > neuromedin N {Mouse, Rat} [997]	neurotensin (*NTS*, P30990) = neuromedin N {Mouse, Rat} [1685]
Agonists	ABS-201 [439, 2946] – Mouse, ABS-212 [1084, 1494] – Rat	–
Selective agonists	JMV449 [2400] – Rat	levocabastine [1685, 2177]
Selective antagonists	meclinertant (pIC_50_ 7.5–8.2) [897]	–
Labelled ligands	[^3^H]meclinertant (Antagonist) (pK_d_ 8.5) [1394] – Rat	–

**Comments**: Neurotensin (*NTS*, P30990) appears to be a low-efficacy agonist at the NTS_2_ receptor [2690], while the NTS_1_ receptor antagonist meclinertant is an agonist at NTS_2_ receptors [2690]. An additional protein, provisionally termed NTS_3_ (also known as NTR3, gp95 and sortilin; ENSG00000134243), has been suggested to bind lipoprotein lipase and mediate its degradation [1895]. It has been reported to interact with the NTS_1_ receptor [1660] and the NTS_2_ receptor [180], and has been implicated in hormone trafficking and/or neurotensin uptake. A splice variant of the NTS_2_ receptor bearing 5 transmembrane domains has been identified in mouse [250] and later in rat [2026]. The neurotensinergic system is implicated in various physiological and pathological processes related to neuropsychiatric and metabolic functions, cancer growth, food, and drug intake [1127].

Further reading on Neurotensin receptorsBoulesM
 (2013) Diverse roles of neurotensin agonists in the central nervous system. Front Endocrinol (Lausanne)
4: 3623526754
10.3389/fendo.2013.00036PMC3605594ChristouN
 (2020) Neurotensin pathway in digestive cancers and clinical applications: an overview. Cell Death Dis
11: 102733268796
10.1038/s41419-020-03245-8PMC7710720MazellaJ
 (2012) Neurotensin and its receptors in the control of glucose homeostasis. Front Endocrinol (Lausanne)
3: 14323230428
10.3389/fendo.2012.00143PMC3515879MyersRM
 (2009) Cancer, chemistry, and the cell: molecules that interact with the neurotensin receptors. ACS Chem Biol
4: 503–2519462983
10.1021/cb900038eOuyangQ
 (2017) Oncogenic role of neurotensin and neurotensin receptors in various cancers. Clin Exp Pharmacol Physiol
44: 841–84628556374
10.1111/1440-1681.12787SchroederLE
 (2018) Role of central neurotensin in regulating feeding: Implications for the development and treatment of body weight disorders. Biochim Biophys Acta Mol Basis Dis
1864: 900–91629288794
10.1016/j.bbadis.2017.12.036PMC5803395

## Opioid receptors

G protein-coupled receptors → Opioid receptors

**Overview**: Opioid and opioid-like receptors are activated by a variety of endogenous peptides including [Met]enkephalin (*PENK*, P01210) (met), [Leu]enkephalin (*PENK*, P01210) (leu), β-endorphin (*POMC*, P01189) (β-end), α-neodynorphin (*PDYN*, P01213), dynorphin A (*PDYN*, P01213) (dynA), dynorphin B (*PDYN*, P01213) (dynB), big dynorphin (*PDYN*, P01213) (Big dyn), nociceptin/orphanin FQ (*PNOC*, Q13519) (N/OFQ); endo-morphin-1 and endomorphin-2 are also potential endogenous peptides. The Greek letter nomenclature for the opioid receptors, μ, δ and κ, is well established, and **NC-IUPHAR** considers this nomenclature appropriate, along with the symbols spelled out (mu, delta, and kappa), and the acronyms, MOP, DOP, and KOP [500, 572, 712]. However the acronyms MOR, DOR and KOR are still widely used in the literature. The human N/OFQ receptor, NOP, is considered’opioid-related’ rather than opioid because, while it exhibits a high degree of structural homology with the conventional opioid receptors [1770], it displays a distinct pharmacology. Currently there are numerous clinically used drugs, such as morphine and many other opioid analgesics, as well as antagonists such as naloxone. The majority of clinically used opiates are relatively selective μ agonists or partial agonists, though there are some μ/κ compounds, such as butorphanol, in clinical use. κ opioid agonists, such as the alkaloid nalfurafine and the peripherally acting peptide difelikefalin, are in clinical use for itch.

**Table:** 

Nomenclature	δ receptor	κ receptor
HGNC, UniProt	*OPRD1*,P41143	*OPRK1*,P41145
Principal endogenous agonists	β-endorphin (*POMC*,P01189), [Leu]enkephalin (*PENK*,P01210), [Met]enkephalin (*PENK*,P01210)	big dynorphin (*PDYN*,P01213), dynorphin A (*PDYN*,P01213)
Agonists	DADLE [2593], BU08028 (Partial agonist) [1259], etorphine [2593], ethylketocyclazocine [2593], cebranopadol [1534], PN6047 (Biased agonist) [481]	(−)-cyclazocine (Partial agonist) [2593], etorphine [2593], ethyketazocine [2593, 2954], cebranopadol [1534], BU08028 [1259]
Selective agonists	UFP-512 [2682], BW373U86 [1433], ADL5859 [1433], DPDPE [1814, 2593], [D-Ala^2^]deltorphin II [661], ADL5747 [1434], SNC80 [335, 2118]	difelikefalin [2310], U50488 [394, 2005, 2389, 2593, 2698, 2952, 2954], enadoline [1088, 1881], U69593 [1398, 2593], salvinorin A [160, 2212]
Antagonists	UFP-505 (p*K*_i_ 9.8) [582, 583], naltrexone (p*K*_i_ 8) [2593], AT-076 (p*K*_i_ 7.7) [2593, 2914], naloxone (p*K*_i_ 7.2) [2593]	buprenorphine (p*K*_i_ 9.1–10.2) [2593, 2954], nalmefene (p*K*_i_ 9.5) [2593], naltrexone (p*K*_i_ 8.4–9.4) [2005, 2389, 2593], AT-076 (p*K*_i_ 8.9) [2593, 2915], naloxone (p*K*_i_ 7.6–8.6) [2005, 2389, 2593, 2952, 2954]
Selective antagonists	naltriben (p*K*_i_ 10) [2424, 2593], naltrindole (p*K*_i_ 9.7) [2071, 2593], TIPPy (Inverse agonist) (p*K*_i_ 9) [2293, 2593]	nor-binaltorphimine (p*K*_i_ 8.9–11) [2005, 2070, 2389, 2593, 2952, 2954], 5’-guanidinonaltrindole (p*K*_i_ 9.7–9.9) [1187, 2005, 2456], JDTic (p*K*_i_ 9–9.4) [1829, 2571, 2915]
Labelled ligands	[^3^H]naltrindole (Antagonist) (p*K*_d_ 10.4) [2853] – Rat, [^3^H][D-Ala2]deltorphin I (Selective Agonist) [2452], [^3^H]diprenorphine (Agonist) [65, 2593], [^3^H]DPDPE (Agonist) [32], [^3^H] deltorphin II (Agonist) [326], [^3^H]naltriben (Antagonist) [1488]	[^3^H]diprenorphine (Antagonist) (p*K*_d_ 9.1) [65, 2389], [^3^H]U69593 (Agonist) [1398, 2005, 2389], [^3^H]enadoline (Agonist) [2391]

**Table:** 

Nomenclature	μ receptor	NOP receptor
HGNC, UniProt	*OPRM1*, P35372	*OPRL1*, P41146
Potential endogenous agonists	endomorphin-1, endomorphin-2	–
Principal endogenous agonists	β-endorphin (*POMC*, P01189), [Met]enkephalin (*PENK*, P01210), [Leu]enkephalin (*PENK*, P01210)	nociceptin/orphanin FQ (*PNOC*, Q13519) [14, 201, 1946]
Endogenous agonists	Several additional extended enkephalin peptides or truncated beta-endorphin peptides. [839]	–
Agonists	levorphanol [933], hydromorphone [2766], etorphine [2593], fentanyl [2593], cebranopadol [1534], morphine [836, 2593], buprenorphine (Partial agonist) [2593], BU08028 (Partial agonist) [1259], methadone [2073], UFP-505 [582, 583], codeine [2593], tapentadol [2622], pethidine [2073]	nociceptin/orphanin FQ (*PNOC*, Q13519) [14, 201, 1946], cebranopadol [1534], BU08028 (Partial agonist) [1259]
Selective agonists	sufentanil [2693], DAMGO [932, 2593], loperamide [416], PL017 [385, 2593]	N/OFQ-(1-13)-NH_2_ [201, 893, 1693, 1946], MCOPPB [973], Ac-RYYRWK-NH_2_ (Partial agonist) [604, 1693], UFP-112 [338, 2192], SCH221510 [2674], Ro64-6198 [1156, 2781], AT-403 [73]
Antagonists	naltrexone (p*K*_i_ 9.1–9.7) [1239, 2593], nalmefene (p*K*_i_ 9.5) [2593], nalorphine (p*K*_i_ 8.9) [2593], naloxone (p*K*_i_ 8.9) [2593], AT-076 (p*K*_i_ 8.8) [2593, 2915], methylnaltrexone (p*K*_i_ 8.7) [2766]	AT-076 (p*K*_i_ 8.8) [2915]
Selective antagonists	CTOP (p*K*_i_ 9.7) [898, 2152], alvimopan (peripheral) (p*K*_i_ 9.3) [1432], CTAP (p*K*_i_ 8.6) [385, 2593]	UFP-101 (p*K*_i_ 10.2) [337], LY2940094 (p*K*_i_ 10) [693, 2591], compound 24 (p*K*_i_ 9.6) [705], SB 612111 (p*K*_i_ 9.2–9.5) [2438, 2913], J-113397 (pIC_50_ 8.3) [1232]
Allosteric modulators (Positive)	BMS-986121 (p*K*_B_ 5.7) [316], BMS-986122 (p*K*_B_ 5.3) [316]	–
Allosteric modulators (Neutral)	BMS-986123 (p*K*_B_ 6) [316], BMS-986124 (p*K*_B_ 5.7) [316]	–
Labelled ligands	[^3^H]diprenorphine (Antagonist) (p*K*_d_ 10.1) [2152] – Mouse, [^3^H]DAMGO (Agonist) [2152] – Rat, [^3^H]CTOP (Selective Antagonist) [2592]	[^3^H]N/OFQ (Agonist) [604, 1769]

**Comments**: Three genes for naloxone-sensitive opioid receptors have been identified in humans, and while the μ receptor in particular may be subject to extensive alternative splicing [1991], these putative isoforms have not been correlated with any of the subtypes of receptor proposed in years past. Opioid receptors may heterodimerize with each other or with other 7TM receptors [1189], and give rise to complexes with a unique pharmacology, however, evidence for such heterodimers in native cells is equivocal and the consequences of this heterodimerization for signalling remains largely unknown. For μ-opioid receptors at least, dimerization does not seem to be required for signalling [1388]. A distinct met-enkephalin receptor lacking structural resemblance to the opioid receptors listed has been identified (*OGFR*, 9NZT2) and termed an opioid growth factor receptor [2910].

endomorphin-1 and endomorphin-2 have been identified as highly selective, putative endogenous agonists for the μ-opioid receptor. At present, however, the mechanisms for endomorphin synthesis *in vivo* have not been established, and there is no gene identified that encodes for either. Thus, the status of these peptides as endogenous ligands remains unproven.

Two areas of increasing importance in defining opioid receptor function are the presence of functionally relevant single nucleotide polymorphisms in human μ-receptors [1927] and the identification of biased signalling by opioid receptor ligands, both agonists and antagonists [301, 1238]. Despite the identification of biased ligands for the μ receptor, the relevance with respect to physiological and behavioral actions *in vivo* is not clear [820]. Pathway bias for agonists makes general rank orders of potency and efficacy somewhat obsolete, so these do not appear in the table. As ever, the mechanisms underlying the acute and long term regulation of opiod receptor function are the subject of intense investigation and debate.

The richness of opioid receptor pharmacology has been enhanced with the recent discovery of allosteric modulators of μ and δ receptors, notably the positive allosteric modulators and silent allosteric “antagonists” outlined in [316, 317]. Negative allosteric modulation of opioid receptors has been previously suggested [1223], whether all compounds are acting at a similar site remains to be established.

In the last decade, several opioid receptors structures have been solved in their inactive and active forms: δ receptor [688, 689, 864, 2740]; κ receptor [395, 396, 2740, 2816]; μ receptor [679, 1077, 1318, 1636, 2109, 2195, 2723, 2740, 2959]; NOP [1743, 2574, 2740]. This effort is of great importance for novel structure-based drug design studies.

Further reading on Opioid receptorsButelmanER
 (2012) κ-opioid receptor/dynorphin system: genetic and pharmacotherapeutic implications for addiction. Trends Neurosci
35: 587–9622709632
10.1016/j.tins.2012.05.005PMC3685470CoxBM
 (2015) Challenges for opioid receptor nomenclature: IUPHAR Review 9. Br J Pharmacol
172: 317–2324528283
10.1111/bph.12612PMC4292949GillisA
 (2020) Intrinsic Efficacy of Opioid Ligands and Its Importance for Apparent Bias, Operational Analysis, and Therapeutic Window. Mol Pharmacol
98: 410–42432665252
10.1124/mol.119.119214PradhanAA
 (2011) The delta opioid receptor: an evolving target for the treatment of brain disorders. Trends Pharmacol Sci
32: 581–9021925742
10.1016/j.tips.2011.06.008PMC3197801ValentinoRJ
 (2020) Opioid Research: Past and Future. Mol Pharmacol
98: 389–39132660966
10.1124/molpharm.120.000093PMC7562970WilliamsJT
 (2013) Regulation of μ-opioid receptors: desensitization, phosphorylation, internalization, and tolerance. Pharmacol Rev
65: 223–5423321159
10.1124/pr.112.005942PMC3565916

## Orexin receptors

G protein-coupled receptors → Orexin receptors

**Overview**: Orexin receptors (**nomenclature as agreed by the NC-IUPHAR Subcommittee on Orexin receptors** [712]) are activated by the endogenous polypeptides orexin-A(*HCRT*, O43612) and orexin-B (*HCRT*,O43612) (also known as hypocretin-1 and -2; 33 and 28 aa) derived from a common precursor, preproorexin or orexin precursor, by proteolytic cleavage and some typical peptide modifications [2248]. Orexin signaling has been associated with regulation of sleep and wakefulness, reward and addiction, appetite and feeding, pain gating, stress response, anxiety and depression. Currently the orexin receptor ligands in clinical use are the dual orexin receptor antagonists suvorexant and lemborexant and daridorexant, which are used as hypnotics, and several dual and OX_2_-selective antagonists are under development. Multiple orexin agonists are in development for the treatment of narcolepsy and other sleep disorders. Orexin receptor 3D structures have been solved [84, 991, 1048, 2139, 2493, 2886, 2888, 2889].

**Table:** 

Nomenclature	OX_1_ receptor	OX_2_ receptor
HGNC, UniProt	*HCRTR1*, O43613	*HCRTR2*, O43614
Potency order of endogenous ligands	orexin-A (*HCRT*, O43612) > orexin-B (*HCRT*, O43612) (for Ca^2+^ elevation, unclear/variable for other responses)	orexin-A (*HCRT*, O43612) = orexin-B (*HCRT*, O43612)
Agonists	RTOXA-43 [2921]	RTOXA-43
Selective agonists	–	[Ala^11^, D-Leu^15^]orexin-B [85, 2102], danavorexton (TAK-925) [2908], Nag 26 [1113, 1841, 2184], YNT-185 [1113, 1841]
Antagonists	SB-649868 (p*K*_i_ 9.1–9.6) [336, 501, 573], daridorexant (p*K*_B_ 8.8–9.3) [2612], suvorexant (p*K*_i_ 8.7–9.3) [336, 501, 1816, 2139], filorexant (p*K*_i_ 9.2) [2139], filorexant (p*K*_i_ 8.4–9.1) [336, 501, 2798], TCS 1102 (p*K*_B_ 8–8.5) [183, 2184], almorexant (p*K*_i_ 8.3–8.5) [1625, 1628, 2612]	SB-649868 (p*K*_i_ 8.9–9.8) [336, 501], TCS 1102 (p*K*_B_ 8.8–9.7) [183, 2184], filorexant (p*K*_i_ 9.7) [2139], suvorexant (p*K*_i_ 8.9–9.5) [336, 501, 1816, 2139], almorexant (p*K*_i_ 8.6–9.4) [1624, 1625, 1628, 2612], daridorexant (p*K*_B_ 8.9–9.1) [2139, 2612], filorexant (p*K*_i_ 8.9–9.1) [336, 501, 2798]
Selective antagonists	SB-674042 (70–120-fold selective pro-OX_1_) (p*K*_i_ 8.7–9.3) [1411, 1625], SB-334867 (40–150-fold selective pro-OX1) (p*K*_i_ 7.2–7.9) [236, 1411, 1625, 2068, 2139], SB-408124 (32–65-fold selective pro-OX1) (p*K*_i_ 7.5) [1625]	EMPA (790–3500-fold selective pro-OX_2_) (p*K*_i_ 8.4–9.2) [1624, 1625, 2139], JNJ-10397049 (500–630-fold selective pro-OX2) (p*K*_i_ 7.7–8.4) [1687]
Labelled ligands	[^3^H]SB-674042 (Antagonist) (p*K*_d_ 8.3–9.1) [1411, 1625, 1628], [^3^H]-almorexant (Antagonist) (p*K*_d_ 8.6–8.9) [1625, 1628], [^125^I]orexin A (human, mouse, rat) (Useful working concentration sub nM-low nM.) [1371, 2101, 2248]	[^3^H]-almorexant (Selective Antagonist) (p*K*_d_ 8.9–9.8) [1625, 1628], [^3^H] Cp-1 (Selective Antagonist) (p*K*_d_ 9.2–9.4) [1625], [^3^H]EMPA (Selective Antagonist) (p*K*_d_ 8.6–9) [1624, 1628, 1751], [^125^I]orexin A (human, mouse, rat) (Useful working concentration sub nM-low nM.) [1371, 2101, 2248]

**Comments**: The primary coupling of orexin receptors to G_q/11_ proteins is rather speculative and based on the strong activation of phospholipase C, though recent studies in recombinant cells also stress the importance of G_q/11_ [1372]. Coupling of both receptors to G_i/o_, G_s_ and and G_12/13_ has also been reported [1109, 1221, 1375, 1474, 2136]. For most native cellular responses observed, the G protein pathway is unknown. The relative potency order of endogenous ligands depends on the cellular signal transduction machinery [1373]. Similarly, [Ala^11^, D-Leu^15^] orexin-B, Nag 26 and YNT-185 may show variable selectivity for OX_2_ receptors and may also activate OX_1_ receptors [2102, 2184, 2849]. Thorough characterization of many antagonists and radioligands has not been published, but the situation has recently improved for many commercially available ones. Among radioligands, [^3^H]SB-674042, [^3^H]EMPA, [^3^H]-almorexant, [^125^I]orexin A (human, mouse, rat), [^125^I]-orexin-B and [^125^I][Ala^11^, D-Leu^15^] orexin-B are commercially available. [^3^H]-TCS 1102, [^3^H]Cp-1 and Rhodamine Green-orexin-A [522] are also useful labelled tools. Orexin receptors have been reported to be able to form complexes with each other and some other GPCRs as well as σ1-receptors, which might affect the signaling and pharmacology [1374, 1860]. Antagonists of the orexin receptors are the focus of major drug discovery efforts for their potential to treat insomnia and other disorders of wakefulness [2198], while agonists would likely be useful in human narcolepsy [674, 2849].

Further reading on Orexin receptorsBurdakovD. (2019) Reactive and predictive homeostasis: Roles of orexin/hypocretin neurons. Neuropharmacology
154: 61–6730347195
10.1016/j.neuropharm.2018.10.024JacobsonLH
 (2022) Hypocretins (orexins): The ultimate translational neuropeptides. J Intern Med
291: 533–55635043499
10.1111/joim.13406JamesMH
 (2022) Orexin Reserve: A Mechanistic Framework for the Role of Orexins (Hypocretins) in Addiction. Biol Psychiatry
92: 836–84436328706
10.1016/j.biopsych.2022.06.027PMC10184826KukkonenJP
 (2021) Cellular Signaling Mechanisms of Hypocretin/Orexin. Front Neurol Neurosci
45: 91–10234052812
10.1159/000514962SakuraiT
 (2021) Interaction between Orexin Neurons and Monoaminergic Systems. Front Neurol Neurosci
45: 11–2134052806
10.1159/000514955

## Oxoglutarate receptor

G protein-coupled receptors → Oxoglutarate receptor

**Overview**: **Nomenclature as recommended by NC-IUPHAR** [532].

**Table:** 

Nomenclature	oxoglutarate receptor
HGNC, UniProt	*OXGR1*, Q96P68
Endogenous agonists	α-ketoglutaric acid [979, 2436]

Further reading on Oxoglutarate receptorDavenportAP
 (2013) International Union of Basic and Clinical Pharmacology. LXXXVIII. G protein-coupled receptor list: recommendations for new pairings with cognate ligands. Pharmacol Rev
65: 967–8623686350
10.1124/pr.112.007179PMC3698937

## P2Y receptors

G protein-coupled receptors → P2Y receptors

**Overview**: P2Y receptors (**nomenclature as agreed by the NC-IUPHAR Subcommittee on P2Y Receptors** [3, 4, 1133]) are activated by the endogenous ligands ATP, ADP, UTP, UDP, UDP-glucose and adenosine. The eight mammalian P2Y receptors are activated by distinct nucleotides: P2Y_1_, P2Y_11_, P2Y_12_ and P2Y_13_ are activated by adenosine-nucleotides; P2Y_2_, P2Y_4_ can be activated by both adenosine and uridine nucleotides, with some species-specific differences; P2Y_6_ is mainly activated by UDP; P2Y_14_ is preferentially activated by sugar-uracil nucleotides. The missing numbers in the receptor nomenclature refer either to non-mammalian orthologs or receptors having some sequence homology to P2Y receptors but for which there is no functional evidence of responsiveness to nucleotides [2696]. Based on their G protein coupling P2Y receptors can be divided into two subfamilies: P2Y_1_, P2Y_2_, P2Y_4_, P2Y_6_ and P2Y_11_ receptors couple *via* Gq proteins to stimulate phospholipase C followed by increases in inositol phosphates and mobilization of Ca^2+^ from intracellular stores. P2Y_11_ receptors couple in addition to Gs proteins followed by increased adenylate cyclase activity. In contrast, P2Y_12_^, P2Y^_13_^, and P2Y^_14_ receptors signal primarily through activation of Gi proteins and inhibition of adenylate cyclase activity or control of ion channel activity [2696]. Clinically used drugs acting on these receptors include the dinucleoside polyphosphate diquafosol, agonist of the P2Y_2_ receptor subtype, approved in Japan and South Korea for the management of dry eye disease [1419], and the P2Y_12_ receptor antagonists prasugrel, ticagrelor and cangrelor, all approved as antiplatelet drugs [343, 2081].

**Table:** 

Nomenclature	P2Y_1_ receptor	P2Y_2_ receptor	P2Y_4_ receptor	P2Y_6_ receptor
HGNC, UniProt	*P2RY1*, P47900	*P2RY2*, P41231	*P2RY4*,P51582	*P2RY6*, Q15077
Potency order of endogenous ligands	ADP > ATP	UTP > ATP	UTP > ATP (at rat recombinant receptors, UTP = ATP)	UDP ≫ UTP > ADP
Endogenous agonists	ATP (Partial agonist) [2286, 2707], ADP [2286, 2707]	UTP [1273, 1429]	UTP [1246], ATP [2]	UDP [477]
Agonists	ADPβS [2522], 2MeSADP [2286, 2707]	–	–	–
Sub/family-selective agonists	–	diquafosol [2015], denufosol [1430, 2015, 2883], UTPγS [1429]	diquafosol [306], denufosol [2883], UTPγS [1430]	–
Selective agonists	MRS2365 [425], 2-Cl-ADP(α-BH_3_) [102]	MRS2698 [1123], 2-thioUTP [643], PSB1114 (EC_50_ value determined using an IP_3_ functional assay) [643, 644, 1122]	MRS4062 [1662], MRS2927 [1662], (N) methanocarba-UTP [1273]	Rp-5-OMe-UDPαB [824, 905], MRS2957 [1661], MRS2693 [192]
Antagonists	suramin (p*K*_i_ 5.3) [2707], PPADS (p*K*_i_ 5.2) [2707]	–	–	–
Sub/family-selective antagonists	–	reactive blue-2 (pIC_50_ 6) [1149], suramin (pIC_50_ 4.3) [1149, 2286]	PPADS (pEC_50_ 2–5) [1135], reactive blue-2 (pIC_50_ 4.7) [222] – Rat	reactive blue-2 (pK_B_ 6) [2696], PPADS (pK_B_ 4) [2696], suramin (pK_B_ 4) [2696]
Selective antagonists	MRS2500 (p*K*_i_ 8.8–9.1) [362, 1272], MRS2279 (p*K*_i_ 7.9) [2707], MRS2179 (p*K*_i_ 7–7.1) [260, 2707]	AR-C118925XX (pIC_50_ ∼6) [1242], AR-C126313 (pEC_50_ 6) [1123], PSB-416 (pIC_50_ 4.7) [1017]	PSB-16133 (pIC_50_ 6.6) [2121], ATP (pK_d_ 6.2) [1246]	MRS2578 (pIC_50_ 7.4) [1631], MRS2567 (pIC_50_ 6.9) [1631], TIM-38 (pIC_50_ 5.4) [1119]
Selective allosteric modulators	BMS compound 16 (Negative) (p*K*_i_ 6.9) [2920], 2,2’-pyridylisatogen tosylate (Negative) (pIC_50_ 6.8) [766]	–	–	–
Labelled ligands	[^3^H]MRS2279 (Antagonist) (p*K*_d_ 8.1) [2707], [^3^H]2MeSADP (Agonist) [2522], [^35^S]ADPbetaS (Agonist)	–	–	MRS4162-BODIPY conjugate (Selective Agonist) [1155]

**Table:** 

Nomenclature	P2Y_11_ receptor	P2Y_12_ receptor	P2Y_13_ receptor	P2Y_14_ receptor
HGNC, UniProt	*P2RY11*, Q96G91	*P2RY12*, Q9H244	*P2RY13*, Q9BPV8	*P2RY14*, Q15391
Potency order of endogenous ligands	ATP > ADP	ADP > ATP	ADP ≫ ATP	UDP= UDP-glucose
Endogenous agonists	ATP [478, 1136, 2777], UTP [2777], ADP [478]	ADP [1040]	ADP [1657]	UDP [356], UDP-glucose [737], UDPgalactose [379]
Sub/family-selective agonists	ADPβS [478]	2MeSADP [1040], ADPβS [2522]	2MeSADP [1657], 2MeSATP [1657], ADPβS [1657]	–
Selective agonists	AR-C67085 [124, 478], NF546 [1706], ATPγS [478]	–	–	α.β-methylene-2-thio-UDP [523], MRS2905 [1132], 2-thio-UDP [523]
Antagonists	–	cangrelor (pIC_50_ 9.4) [1136], Ap_4_A (pIC_50_ 6) [1657], 2MeSAMP (pIC_50_ 5.4) [2522]	cangrelor (pIC_50_ 8.3) [1657], Ap_4_A (pIC_50_ 6.7) [1657], 2MeSAMP (pIC_50_ 5.6) [1657]	–
Sub/family-selective antagonists	suramin (pIC_50_ 4.8–6) [478], reactive blue-2 (pIC_50_ 5) [478]	–	–	–
Selective antagonists	NF157 (pK_i_ 7.3) [2630], NF340 (pIC_50_ 6.4–7.1) [1706]	AZD1283 (pK_i_ 8) [105, 2925], ARL66096 (pIC_50_ 7.9) [1086, 1087], ticagrelor (pK_i_ 7.8) [2916]	MRS2603 (pIC_50_ 6.2) [1282], MRS2211 (pIC_50_ 6) [1282]	PPTN (pK_i_ 10.1) [134], MRS4625 (pIC_50_ 7.6) [1821]
Labelled ligands	–	[^3^H]2MeSADP (Agonist) [2522], [^3^H] PSB-0413 (Antagonist) (pK_d_ 8.3–8.5) [642, 1935]	[^33^P]2MeSADP (Agonist) [1657]	MRS4174 (Selective Antagonist) (pK_i_ 10.1) [1294], MRS4183 (Selective Agonist) [1293]

**Comments**: A series of 4-alkyloxyimino derivatives of uridine-5’-triphosphate which could be useful for derivatization as fluorescent P2Y_2/4/6_ receptor probes has been synthesized [1155].

Single nucleotide polymorphisms of the P2YR_1_ gene have been associated to different platelet reactivity to ADP [1006]. Three frequent nonsynonymous P2Y_2_ receptor polymorphisms have been identified, one of which was significantly more common in cystic fibrosis patients. This polymorphism is linked to increases in Ca^2+^ influx in transfected cells, and might therefore play a role in disease development [322]. ATP acts as partial agonist/antagonist at the human P2Y_4_ receptor [2696]. Although UTP was also shown to be a biased agonist at P2Y_11_, this is still under debate [1811, 2777]. Clinically used drugs acting on these receptors include the dinucleoside polyphosphate diquafosol, agonist of the P2Y_2_ receptor subtype that is approved in Japan and South Korea for the management of dry eye disease [1419], and the P2Y_12_ receptor antagonists prasugrel, ticagrelor and cangrelor, all approved as antiplatelet drugs [14, 65]. A group of single nucleotide polymorphisms in the P2Y_12_ gene, forming the so called P2Y_12_ H2 haplotype, has been associated with increased platelet responsiveness to ADP, increased risk of peripheral arterial disease and with coronary artery disease [367]. The platelet-type bleeding disorder due to P2Y_12_ receptor defects is an autosomal recessive condition characterized by mild to moderate mucocutaneous bleeding and excessive bleeding after surgery or trauma. The defect is due to the inability of ADP to induce platelet aggregation [363]. The P2Y_13_ receptor Met-158-Thr polymorphism, which is in linkage disequilibrium with the P2Y_12_ locus, is not associated with acute myocardial infarction, diabetes mellitus or related risk factors [54]. The P2Y_14_ receptor, previously considered to exclusively bind sugar nucleotides such as UDP-glucose, UDP-galactose, UDP-glucuronic acid and UDP-N-acethyl-glucosamine [379], has been demonstrated to bind also UDP [356]. UDP was also shown to competitively antagonise the UDP-glucose response at the human recombinant P2Y_14_ receptor [738].

Further reading on P2Y receptorsAbbracchioMP
 (2006) International Union of Pharmacology LVIII: update on the P2Y G protein-coupled nucleotide receptors: from molecular mechanisms and pathophysiology to therapy. Pharmacol Rev
58: 281–34116968944
10.1124/pr.58.3.3PMC3471216JacobsonKA
 (2020) Update of P2Y receptor pharmacology: IUPHAR Review 27. Br J Pharmacol
177: 2413–243332037507
10.1111/bph.15005PMC7205808JacobsonKA
 (2015) Nucleotides Acting at P2Y Receptors: Connecting Structure and Function. Mol Pharmacol
88: 220–3025837834
10.1124/mol.114.095711PMC4518082von KügelgenI
 (2016) Pharmacology and structure of P2Y receptors. Neuropharmacology
104: 50–6126519900
10.1016/j.neuropharm.2015.10.030

## Parathyroid hormone receptors

G protein-coupled receptors → Parathyroid hormone receptors

**Overview**: The parathyroid hormone receptors (**nomenclature as agreed by the NC-IUPHAR Subcommittee on Parathyroid Hormone Receptors** [772]) are class B G protein-coupled receptors. The parathyroid hormone (PTH)/parathyroid hormone-related peptide (PTHrP) receptor (PTH1 receptor) is activated by precursor-derived peptides: PTH (*PTH*, P01270) (84 amino acids), and PTHrP (*PTHLH*, P12272) (141 amino-acids) and related peptides (PTH-(1–34), PTHrP-(1-36) (*PTHLH*, P12272)). The parathyroid hormone 2 receptor (PTH2 receptor) is activated by the precursor-derived peptide TIP39 (*PTH2*, Q96A98) (39 amino acids). [^125^I]PTH may be used to label both PTH1 and PTH2 receptors. The structure of a long-active PTH analogue (LA-PTH, an hybrid of PTH-(1–13) and PTHrP-(14–36)) bound to the PTH1 receptor-G_s_ complex has been resolved by cryo-electron microscopy [2941]. Another structure of a PTH-(1–34) analog bound to a thermostabilized inactive PTH1 receptor has been obtained with X-ray crytallography [639].

**Table:** 

Nomenclature	PTH1 receptor	PTH2 receptor
HGNC, UniProt	*PTH1R*, Q03431	*PTH2R*, P49190
Potency order of endogenous ligands	PTH (*PTH*, P01270) = PTHrP (*PTHLH*, P12272)	TIP39 (*PTH2*, Q96A98), PTH (*PTH*, P01270) ≫ PTHrP (*PTHLH*, P12272)
Agonists	teriparatide [770]	TIP39 (*PTH2*, Q96A98) [846, 1029]
Selective agonists	PTHrP-(1-34) (human) [771] – Rat, abaloparatide [94]	–

**Comments**: The parathyroid hormone type 1 receptor (PTHR) is the canonical GPCR for PTH and PTHrP. It is coupled to G_s_ and G_q_ and regulates the development of bone, heart, mammary glands and other tissues in response to PTHrP, and blood concentrations of calcium and phosphate ions, as well as vitamin D, in response to PTH. Another important action of the PTH/PTHR system is to stimulate bone formation when the hormone is intermittently administrated (daily injection).

Although PTH (*PTH*, P01270) is an agonist at human PTH2 receptors, it fails to activate the rodent orthologues. TIP39 (*PTH2*, Q96A98) is a weak antagonist at PTH1 receptors [1188].

Further reading on Parathyroid hormone receptorsChelohaRW
 (2015) PTH receptor-1 signalling-mechanistic insights and therapeutic prospects. Nat Rev Endocrinol
11: 712–2426303600
10.1038/nrendo.2015.139PMC4651712GardellaTJ
 (2015) International Union of Basic and Clinical Pharmacology. XCIII. The Parathyroid Hormone Receptors-Family B G Protein-Coupled Receptors. Pharmacol Rev
67: 310–3725713287
10.1124/pr.114.009464PMC4394688SutkeviciuteI
 (2020) Structural insights into emergent signaling modes of G protein-coupled receptors. J Biol Chem
295: 11626–1164232571882
10.1074/jbc.REV120.009348PMC7450137VilardagaJP
 (2014) Endosomal generation of cAMP in GPCR signaling. Nat Chem Biol
10: 700–625271346
10.1038/nchembio.1611PMC4417940

## Platelet-activating factor receptor

G protein-coupled receptors → Platelet-activating factor receptor

**Overview**: Platelet-activating factor (PAF, 1-*O*-alkyl-2-acetyl-sn-glycero-3-phosphocholine) is an ether phospholipid mediator associated with platelet coagulation, but also subserves inflammatory roles. The PAF receptor (**provisional nomenclature recommended by NC-IUPHAR** [712]) is activated by PAF and other suggested endogenous ligands are oxidized phosphatidylcholine [1645] and lysophosphatidylcholine [1929]. It may also be activated by bacterial lipopolysaccharide [1847].

**Table:** 

Nomenclature	PAF receptor
HGNC, UniProt	*PTAFR*, P25105
Selective agonists	methylcarbamyl PAF
Selective antagonists	foropafant (p*K*_i_ 10.3) [996], ABT-491 (p*K*_i_ 9.2) [36], CV-6209 (pIC_50_ 8.1–8.3) [835, 1846], L659989 (p*K*_i_ 7.8) [1091], apafant (p*K*_i_ 5.2–7.5) [1985, 2501]
Labelled ligands	[^3^H]PAF (Agonist) [746, 1846]

**Comments**: Note that a previously recommended radioligand ([^3^H]apafant; K_d_ 44.6 nM) is currently unavailable.

Further reading on Platelet-activating factor receptorFoordSM
 (2005) International Union of Pharmacology. XLVI. G protein-coupled receptor list. Pharmacol Rev
57: 279–8815914470
10.1124/pr.57.2.5IshiiS
 (2000) Platelet-activating factor (PAF) receptor and genetically engineered PAF receptor mutant mice. Prog Lipid Res
39: 41–8210729607
10.1016/s0163-7827(99)00016-8PrescottSM
 (2000) Platelet-activating factor and related lipid mediators. Annu Rev Biochem
69: 419–4510966465
10.1146/annurev.biochem.69.1.419

## Prokineticin receptors

G protein-coupled receptors → Prokineticin receptors

**Overview**: Prokineticin receptors, PKR_1_ and PKR_2_ (**provisional nomenclature as recommended by NC-IUPHAR** [712]) respond to the cysteine-rich 81–86 amino-acid peptides prokineticin-1 (*PROK1*, Q9HC23) (also known as endocrine gland-derived vascular endothelial growth factor, mambakine) and prokineticin-2 (*PROK2*, Q9HC23) (protein Bv8 homologue). An orthologue of PROK1 from black mamba (*Dendroaspis polylepi*s) venom, mamba intestinal toxin 1 (MIT1, [2315]) is a potent, non-selective agonist at prokineticin receptors [1666], while Bv8, an orthologue of PROK2 from amphibians (*Bombina sp*., [1766]), is equipotent at recombinant PKR_1_ and PKR_2_ [1870], and has high potency in macrophage chemotaxis assays, which are lost in PKR_1_-null mice.

**Table:** 

Nomenclature	PKR_1_	PKR_2_
HGNC, UniProt	*PROKR1*, Q8TCW9	*PROKR2*, Q8NFJ6
Potency order of endogenous ligands	prokineticin-2 (*PROK2*, Q9HC23) > prokineticin-1 (*PROK1*, Q9HC23) > prokineticin-2β (*PROK2*) [401, 1521, 1666, 2426]	prokineticin-2 (*PROK2*, Q9HC23) > prokineticin-1 (*PROK1*, Q9HC23) > prokineticin-2β (*PROK2*) [401, 1521, 1666, 2426]
Agonists	MIT1 [1666]	MIT1 [1666]
Selective agonists	IS20 [780], IS1 [780]	–
Labelled ligands	[^125^I]BH-MIT1 (Agonist) [1666]	[^125^I]BH-MIT1 (Agonist) [1666]

**Comments**: Genetic mutations in *PROKR1* are associated with Hirschsprung’s disease [2228], while genetic mutations in *PROKR2* are associated with hypogonadotropic hypogonadism with anosmia [593], hypopituitarism with pituitary stalk interruption [2167] and Hirschsprung’s disease [2228]. PKR_2_ has been recently identified as a receptor for *T. cruzi* natural infection [1260]. PROK2 neuropeptide signalling *via* PKR_2_ on spinal neurons generates pleasant touch sensation [1537].

Further reading on Prokineticin receptorsBoulberdaaM
 (2011) Prokineticin receptor 1 (PKR1) signalling in cardiovascular and kidney functions. Cardiovasc Res
92: 191–821856786
10.1093/cvr/cvr228NegriL
 (2018) The Prokineticins: Neuromodulators and Mediators of Inflammation and Myeloid Cell-Dependent Angiogenesis. Physiol Rev
98: 1055–108229537336
10.1152/physrev.00012.2017NegriL
 (2012) Bv8/PK2 and prokineticin receptors: a druggable pronociceptive system. Curr Opin Pharmacol
12: 62–622136937
10.1016/j.coph.2011.10.023NegriL
 (2007) Bv8/Prokineticin proteins and their receptors. Life Sci
81: 1103–1617881008
10.1016/j.lfs.2007.08.011NganES
 (2008) Prokineticin-signaling pathway. Int J Biochem Cell Biol
40: 1679–8418440852
10.1016/j.biocel.2008.03.010

## Prolactin-releasing peptide receptor

G protein-coupled receptors → Prolactin-releasing peptide receptor

**Overview**: The precursor (*PRLH*, P81277) for PrRP generates 31 and 20-amino-acid versions. QRFP43 (43RFa) (*QRFP*, P83859) (named after a pyroglutamylated arginine-phenylalanine-amide peptide) is a 43 amino acid peptide derived from QRFP (P83859) and is also known as P518 or 26RFa. RFRP is an RF amide-related peptide [1018] derived from a FMRFamide-related peptide precursor (*NPVF*, Q9HCQ7), which is cleaved to generate neuropeptide SF (*NPFF*, O15130), neuropeptide RFRP-1 (*NPVF*, Q9HCQ7), neuropeptide RFRP-2 (*NPVF*, Q9HCQ7) and neuropeptide RFRP-3 (*NPVF*, Q9HCQ7) (neuropeptide NPVF).

**Table:** 

Nomenclature	PrRP receptor
HGNC, UniProt	*PRLHR*, P49683
Potency order of endogenous ligands	PrRP-20 (*PRLH*, P81277) = PrRP-31 (*PRLH*, P81277) [1412]
Endogenous agonists	PrRP-20 (*PRLH*, P81277) [655, 1412], PrRP-31 (*PRLH*, P81277) [655, 1412]
Endogenous antagonists	neuropeptide Y (*NPY*, P01303) (p*K*_i_ 5.4) [1396]
Labelled ligands	[^125^I]PrRP-20 (human) (Agonist) [1412], [^125^I]PrRP31 (Agonist) [646]

**Comments**: The orphan receptor *GPR83* (Q9NYM4) shows sequence similarities with NPFF1, NPFF2, PrRP and QRFP receptors.

Further reading on Prolactin-releasing peptide receptorSamsonWK
 (2006) Prolactin releasing peptide (PrRP): an endogenous regulator of cell growth. Peptides
27: 1099–10316500730
10.1016/j.peptides.2006.01.008TakayanagiY
 (2010) Roles of prolactin-releasing peptide and RFamide related peptides in the control of stress and food intake. FEBS J
277: 4998–500521126313
10.1111/j.1742-4658.2010.07932.x

## Prostanoid receptors

G protein-coupled receptors → Prostanoid receptors

**Overview**: Prostanoid receptors (**nomenclature as agreed by the NC-IUPHAR Subcommittee on Prostanoid Receptors** [2810]) are activated by the endogenous ligands prostaglandins PGD_2_, PGE_1_, PGE_2_ , PGF_2__α_, PGH_2_, prostacyclin [PGI_2_] and thromboxane A_2_. Differences and similarities between human and rodent prostanoid receptor orthologues, and their specific roles in pathophysiologic conditions are reviewed in [1912]. Measurement of the potency of PGI_2_ and thromboxane A_2_ is hampered by their instability in physiological salt solution; they are often replaced by cicaprost and U46619, respectively, in receptor characterization studies.

**Table:** 

Nomenclature	DP_1_ receptor	DP_2_ receptor
HGNC, UniProt	*PTGDR*, Q13258	*PTGDR2*, Q9Y5Y4
Potency order of endogenous ligands	PGD_2_>PGE_1_*^≫^* PGE_2_>PGF_2__α_> PGI_2_, thromboxane A_2_	PGD_2_ *^≫^* PGF_2__α_, PGE_2_> PGI_2_, thromboxane A_2_
Agonists	treprostinil [2505, 2780]	–
Selective agonists	BW 245C [225, 2811, 2812], L-644,698 [2811, 2812]	15(R)-15-methyl-PGD_2_ [959, 1778, 2481]
Antagonists	laropiprant (p*K*_i_ 10.1) [1399, 2473]	fevipiprant (p*K*_d_ 9) [2506, 2507], AZD1981 (pIC_50_ 8.4) [1576], ramatroban (p*K*_i_ 7.4) [2481]
Selective antagonists	BWA868C (p*K*_i_ 8.6–9.3) [225, 815, 2811], ONO-AE3-237 (p*K*_i_ 7.7) [1020, 2597, 2600]	TM30089 (pIC_50_ 8.9) [2222, 2634]
Labelled ligands	[^3^H]PGD_2_ (Agonist) [2793, 2811]	[^3^H]PGD_2_ (Agonist) [1668, 2361]

**Table:** 

Nomenclature	EP_1_ receptor	EP_2_ receptor	EP_3_ receptor	EP_4_ receptor
HGNC, UniProt	*PTGER1*,P34995	*PTGER2*,P43116	*PTGER3*, P43115	*PTGER4*, P35408
Potency order of endogenous ligands	PGE_2_ > PGE_1_ > PGF_2α_, PGI_2_ > PGD_2_, thromboxane A_2_	PGE_2_ = PGE_1_ > PGF_2__α_, PGI_2_ > PGD_2_, thromboxane A_2_	PGE_2_, PGE_1_> PGF_2__α_, PGI_2_> PGD_2_, thromboxane A_2_	PGE_2_ = PGE_1_ > PGF_2__α_, PGI_2_ > PGD_2_, thromboxane A_2_
Endogenous agonists	–	PGE_2_ [9, 2459, 2793]	PGE_2_ (EP_3_-III isoform) [9]	–
Agonists	17-phenyl-ω-trinor-PGE_2_ [2352]	treprostinil [2505, 2780], PGE_1_ [145]	misoprostol (methyl ester) (EP_3_-III isoform) [9]	L-161,982 (EP_4_A) [1599, 2902], 17-phenyl-ω-trinor-PGE_2_ [2562]
Selective agonists	ONO-DI-004 [2496] – Mouse	ONO-AE1-259 [2496] – Mouse, omidenepag [1292], butaprost (free acid form) [9, 2459]	sulprostone (EP_3_-III isoform) [9], ONO-AE-248 [719, 1563]	L902688 [720, 1450], KMN-159 [135], ONO-AE1-329 [719, 720]
Selective antagonists	ONO-8711 (p*K*_i_ 9.2) [2747], SC-51322 (p*K*_i_ 7.9) [9]	PF-04418948 (PF-04418948 has weaker affinity at the EP2-receptor in guineapigs) (p*K*_B_ 8.3) [17, 205], TG6-129 (p*K*_B_ 8.1) [763], TG8-260 (p*K*_B_ 7.9) [48, 2149]	L-826266 (EP_3_-III isoform (pK_i_=8.04 in the presence of HSA)) (p*K*_i_ 9.1) [1196], ONO-AE3-240 (pIC_50_ 8.8) [47] – Mouse, DG-041 (p*K*_i_ 8.4) [1194]	ONO-AE3-208 (p*K*_i_ 8.5), GW 627368 (p*K*_i_ 7–7.1) [2793, 2794]
Labelled ligands	[^3^H]PGE_2_ (Agonist) [9, 2352, 2793]	[^3^H]PGE_2_ (Agonist) [9, 2793]	[^3^H]PGE_2_ (Agonist) [9, 2793]	[^3^H]PGE_2_ (Agonist) [9, 539, 2780, 2793]

**Table:** 

Nomenclature	FP receptor	IP receptor	TP receptor
HGNC, UniProt	*PTGFR*,P43088	*PTGIR*,P43119	TBXA2R,P21731
Potency order of endogenous ligands	PGF_2__α_ >PGD_2_> PGE_2_ > PGI_2_, thromboxane A_2_	PGI_2_ *^≫^*PGE_1_> PGD_2_, PGF_2__α_ >thromboxane A_2_	thromboxane A_2_ = PGH_2_ *^≫^* PGD_2_, PGE_2_, PGF_2__α_, PGI_2_
Endogenous agonists	–	PGI_2_ [2380], PGE_1_ [1663, 2461]	–
Agonists	ONO-9054 [2855]	iloprost [9, 2793], treprostinil [2780]	–
Selective agonists	fluprostenol [9], latanoprost (free acid form) [9]	cicaprost [9], MRE-269 [179, 1389]	U46619 [9]
Antagonists	–	–	ramatroban (p*K*_i_ 8) [2563], laropiprant (p*K*_i_ 6.1) [1399]
Selective antagonists	AS604872 (p*K*_i_ 7.5) [463]	CAY10441 (p*K*_i_ 8.7) [213], RO3244794 (pA2 8.5) [213]	vapiprost (p*K*_i_ 8.3–9.4) [79, 1577], SQ-29548 (p*K*_i_ 8.1–9.1) [9, 2504, 2793]
Labelled ligands	[^3^H]PGF_2__α_ (Agonist) [9, 10, 2793], [^3^H](+)-fluprostenol (Agonist)	[^3^H]iloprost (Agonist) [9, 224, 2780, 2793]	[^125^I]SAP (Antagonist) (p*K*_d_ 7.7–9.3) [1845], [^125^I] BOP (Agonist) [1800], [^3^H]SQ-29548 (Antagonist) (p*K*_d_ 7.4–8.2) [9, 2793]

**Comments**: Whilst cicaprost is selective for IP receptors, it does exhibit moderate agonist potency at EP_4_ receptors [9]. Apart from IP receptors, iloprost also binds to EP_1_ receptors.

The EP_1_ agonist 17-phenyl-ω-trinor-PGE_2_ also shows agonist activity at EP_3_ and EP_4_ receptors [719, 2562]. Butaprost and SC46275 may require de-esterification within tissues to attain full agonist potency. There is evidence for subtypes of FP [1517] and TP receptors [1356, 2151]. mRNA for the EP_3_ receptor undergoes alternative splicing to produce variants which can interfere with signalling [1948] or generate complex patterns of G-protein (G_i/o_, G_q/11_, G_s_ and G_12,13_) coupling (*e.g*. [1343, 1868]). The number of EP_3_ receptor (protein) variants are variable depending on species. For the human prostaglandin EP_3_ receptor, there exist five different EP_3_ isoform proteins (EP_3_-I, EP_3_-II, EP_3_-III, EP_3_-IV and EP_3_-e). Three isoforms exist in rat and mouse. Putative receptor(s) for prostamide F (which as yet lack molecular correlates) and which preferentially recognize PGF2-1-ethanolamide and its analogues (*e.g*. Bimatoprost) have been identified, together with moderate-potency antagonists (*e.g*. AGN 211334) [2809].

The free acid form of AL-12182, AL12180, used in *in vitro* studies, has a EC_50_ of 15nM which is the concentration of the compound giving half-maximal stimulation of inositol phosphate turnover in HEK-293 cells expressing the human FP receptor [2353].

References given alongside the TP receptor agonists I-BOP [1683] and STA_2_ [79] use human platelets as the source of TP receptors for competition radio-ligand binding assays to determine the indicated activity values.

Pharmacological evidence for a second IP receptor, denoted IP_2_, in the central nervous system [2528, 2750] and in the BEAS-2B human airway epithelial cell line [2796] is available. This receptor is selectively activated by 15R-17,18,19,20-tetranor-16-m-tolyl-isocarbacyclin (15R-TIC) and 15R-deoxy 17,18,19,20-tetranor-16-m-tolyl-isocarbacyclin (15-deoxy-TIC). However, molecular biological evidence for an IP_2_ subtype is currently lacking.

Further reading on Prostanoid receptorsNorelX
 (2020) International Union of Basic and Clinical Pharmacology. CIX. Differences and Similarities between Human and Rodent Prostaglandin E_2_ Receptors (EP1–4) and Prostacyclin Receptor (IP): Specific Roles in Pathophysiologic Conditions. Pharmacol Rev
72: 910–96832962984
10.1124/pr.120.019331PMC7509579WoodwardDF
 (2011) International union of basic and clinical pharmacology. LXXXIII: classification of prostanoid receptors, updating 15 years of progress. Pharmacol Rev
63: 471–53821752876
10.1124/pr.110.003517

## Proteinase-activated receptors

G protein-coupled receptors → Proteinase-activated receptors

**Overview**: Proteinase-activated receptors (PARs, **nomenclature as agreed by the NC-IUPHAR Subcommittee on Proteinase-activated Receptors** [1037]) are unique members of the GPCR superfamily activated by proteolytic cleavage of their amino terminal exodomains. Agonist proteinase-induced hydrolysis unmasks a tethered ligand (TL) at the exposed amino terminus, which acts intramolecularly at the binding site in the body of the receptor to effect transmembrane signalling. TL sequences at human PAR1-4 are SFLLRN-NH_2_, SLIGKV-NH_2_, TFRGAP-NH_2_ and GYPGQV-NH_2_, respectively. With the exception of PAR3, synthetic peptides with these sequences (as carboxyl terminal amides) are able to act as agonists at their respective receptors. Several proteinases, including neutrophil elastase, cathepsin G and chymotrypsin can have inhibitory effects at PAR1 and PAR2 such that they cleave the exodomain of the receptor without inducing activation of Gαq-coupled calcium signalling, thereby preventing activation by activating proteinases but not by agonist peptides. Neutrophil elastase (NE) cleavage of PAR1 and PAR2 can however activate MAP kinase signaling by exposing a TL that is different from the one revealed by trypsin [2128]. PAR2 activation by NE regulates inflammation and pain responses [1822, 2942] and triggers mucin secretion from airway epithelial cells [2948].

**Table:** 

Nomenclature	PAR1	PAR2	PAR3	PAR4
HGNC, UniProt	*F2R*, P25116	*F2RL1*, P55085	*F2RL2*, O00254	*F2RL3*, Q96RI0
Agonist proteases	thrombin (*F2*, P00734), activated protein C (*PROC*, P04070), matrix metalloproteinase 1 (*MMP1*, P45452), matrix metalloproteinase 13 (*MMP13*, P45452) [96]	Trypsin, tryptase, TF/VIIa, Xa; elastase, neutrophil expressed; cathepsin S [1171, 2126]	thrombin (*F2*, P00734)	thrombin (*F2*, P00734), trypsin, cathepsin G (*CTSG*, P08311)
Agonists	F16357	–	–	–
Selective agonists	TFLLR-NH_2_ [1039]	Isox-Cha-Chg-Ala-Arg-Dpr(4FB)-NH2 [1484], AY77 [2879], AZ2429 [1244], GB110 [138], 2-furoyl-LIGRLO-amide [1695], SLIGKV-NH_2_ [1457], SLIGRL-NH_2_ [1457]	–	AYPGKF-NH2, GYPGKF-NH2, GYPGQV-NH_2_
Selective antagonists	vorapaxar (p*K*_i_ 8.1) [375], atopaxar (pIC_50_ 7.7) [1321], SCH-79797 (pIC_50_ 7.2) [23], RWJ-56110 (pIC_50_ 6.4) [61]	I-191 (pIC_50_ 7.1) [1170], AZ8838 (p*K*_d_ 6.5) [421], GB88 (pIC_50_ 5.7) [2478], P2pal18S (pIC_50_ 5.4)	–	BMS-986120 (pIC_50_ 9.3) [2083, 2805], BMS-986141 (pIC_50_ 9.3) [2083], YD-3 (pIC_50_ 6.9) [2761], ML354 (pIC_50_ 6.8) [2761], P4pal-10 [499], RAG8 [2127]
Allosteric modulators (Negative)	–	AZ3451 (pIC_50_ 7.6) [421], I-287 (functionally selective) (pIC_50_ 7.1) [97], I-287 (functionally selective) (pIC_50_ 6.4) [97]	–	–
Labelled ligands	[^3^H]haTRAP (Agonist) [22]	Isox-Cha-Chg-ARK(Sulfo-Cy5)-NH2 (Selective Agonist) [1483], 2-furoyl-LIGRL[N-(Alexa Fluor 594)-O]-NH_2_ (Agonist) [1038], 2-furoyl-LIGRL[N[^3^H] propionyl]-O-NH_2_ (Agonist) [1038], [^3^H]2-furoyl-LIGRL-NH_2_ (Selective Agonist) [1214], trans-cinnamoyl-LIGRLO [N-[^3^H]propionyl]-NH_2_ (Agonist) [34]	–	–
Comments	TFLLR-NH_2_ is selective relative to the PAR_2_ receptor [207, 1228].	2-Furoyl-LIGRLO-NH_2_ activity was measured via calcium mobilisation in HEK 293 cells which constitutively coexpress human PAR_1_ and PAR_2_.	–	–

**Comments**: Endogenous serine proteases (EC 3.4.21.) active at the proteinase-activated receptors include: thrombin (*F2*, P00734), generated by the action of Factor X (*F10*, P00742) on liver-derived prothrombin (*F2*, P00734); trypsin, generated by the action of enterokinase (*TMPRSS15*, P98073) on pancreatic-derived trypsinogen (*PRSS1*, P07477); tryptase, a family of enzymes (α/β1 *TPSAB1*, Q15661 ; γ1 *TPSG1*, Q9NRR2; δ1 *TPSD1*, Q9BZJ3) secreted from mast cells; cathepsin G (*CTSG*, P08311) generated from leukocytes; liver-derived protein C (*PROC*, P04070) generated in plasma by thrombin (*F2*, P00734) and matrix metalloproteinase 1 (*MMP1*, P45452).

Further reading on Proteinase-activated receptorsAdamsMN
 (2011) Structure, function and pathophysiology of protease activated receptors. Pharmacol Ther
130: 248–8221277892
10.1016/j.pharmthera.2011.01.003CantoI
 (2012) Allosteric modulation of protease-activated receptor signaling. Mini Rev Med Chem
12: 804–1122681248
10.2174/138955712800959116PMC4237590HollenbergMD
 (2002) International Union of Pharmacology. XXVIII. Proteinase-activated receptors. Pharmacol Rev
54: 203–1712037136
10.1124/pr.54.2.203PeachCJ
 (2023) Protease-activated receptors in health and disease. Physiol Rev
103: 717–78535901239
10.1152/physrev.00044.2021PMC9662810RamachandranR
 (2012) Targeting proteinase-activated receptors: therapeutic potential and challenges. Nat Rev Drug Discov
11: 69–8622212680
10.1038/nrd3615SohUJ
 (2010) Signal transduction by protease-activated receptors. Br J Pharmacol
160: 191–20320423334
10.1111/j.1476-5381.2010.00705.xPMC2874842

## QRFP receptor

G protein-coupled receptors → QRFP receptor

**Overview**: The human gene encoding the QRFP receptor (**nomenclature as agreed by the NC-IUPHAR Subcommittee on the QRFP receptor** [1478]; QRFPR, formerly known as the Peptide P518 receptor), previously designated as an orphan GPCR receptor was identified in 2001 by Lee *et al*. from a hypothalamus cDNA library [1452]. However, the reported cDNA (AF411117) is a chimera with bases 1–127 derived from chromosome 1 and bases 155–1368 derived from chromosome 4. When corrected, QRFPR (also referred to as SP9155 or AQ27) encodes a 431 amino acid protein that shares sequence similarities in the transmembrane spanning regions with other peptide receptors. These include neuropeptide FF2 (38%), neuropeptide Y_2_ (37%) and galanin Gal_1_ (35%) receptors. QRFP receptor was identified as a Gs-coupled GPCR [392, 1169] that’s activated by the endogenous peptides QRFP43 (43RFa) and QRFP26 (26RFa) [392, 748, 1169]. However, Gq- and Gi/o-mediated signaling was also reported [748, 2130]. Two naturally occurring mutations in the human QRFP receptor lead to distinct and opposite 26RFa-evoked signaling bias [1592].

**Table:** 

Nomenclature	QRFP receptor
HGNC, UniProt	*QRFPR*,Q96P65
Endogenous agonists	QRFP43 (43RFa) (*QRFP*, P83859) [747, 2436], QRFP26 (26RFa) (*QRFP*) [392, 1169]
Agonists	LV-2186 [46], LV-2172 [1882], LV-2211 [1462]
Selective antagonists	compound 25e (pIC_50_ 7.3) [800, 801]
Labelled ligands	[^125^I]QRFP43 (human) (Agonist) [748, 2527], [^125^I]26RFa (human) (Agonist) [307]

**Comments**: The orphan receptor *GPR83* (9NYM4) shows sequence similarities with the QRFP receptor, as well as with the NPFF1, NPFF2, and PrRP receptors.

Further reading on QRFP receptorChartrelN
 (2011) The RFamide neuropeptide 26RFa and its role in the control of neuroendocrine functions. Front Neuroendocrinol
32: 387–9721530572
10.1016/j.yfrne.2011.04.001FukusumiS
 (2006) Recent advances in mammalian RFamide peptides: the discovery and functional analyses of PrRP, RFRPs and QRFP. Peptides
27: 1073–8616500002
10.1016/j.peptides.2005.06.031LeprinceJ
 (2017) The Arg-Phe-amide peptide 26RFa/glutamine RF-amide peptide and its receptor: IUPHAR Review 24. Br J Pharmacol
174: 3573–360728613414
10.1111/bph.13907PMC5610166PrévostG
 (2019) Neuropeptide 26RFa (QRFP) is a key regulator of glucose homeostasis and its activity is markedly altered in obese/hyperglycemic mice. Am J Physiol Endocrinol Metab
317: E147–E15731084498
10.1152/ajpendo.00540.2018

## Relaxin family peptide receptors

G protein-coupled receptors → Relaxin family peptide receptors

**Overview**: Relaxin family peptide receptors (RXFP, **nomenclature as agreed by the NC-IUPHAR Subcommittee on Relaxin family peptide receptors** [146, 919]) may be divided into two pairs, RXFP1/2 and RXFP3/4. Endogenous agonists at these receptors are heterodimeric peptide hormones structurally related to insulin: relaxin-1 (*RLN1*, P04808), relaxin (*RLN2*, P04090), relaxin-3 (*RLN3*, Q8WXF3) (also known as INSL7), insulin-like peptide 3 (INSL3 (*INSL3*, P51460)) and INSL5 (*INSL5*, Q9Y5Q6). Species homologues of relaxin have distinct pharmacology and relaxin (*RLN2*, P04090) interacts with RXFP1, RXFP2 and RXFP3, whereas mouse and rat relaxin selectively bind to and activate RXFP1 [2322]. Relaxin-3 (*RLN3*, Q8WXF3) is the ligand for RXFP3 but it also binds to RXFP1 and RXFP4 and has differential affinity for RXFP2 between species [2321]. INSL5 (*INSL5*, Q9Y5Q6) is the ligand for RXFP4 but is a weak antagonist of RXFP3. Relaxin (*RLN2*, P04090) and INSL3 (*INSL3*, P51460) have multiple complex binding interactions with RXFP1 [2342] and RXFP2 [1028] which direct the N-terminal LDLa modules of the receptors together with a linker domain to act as a tethered ligand to direct receptor signaling [2323]. INSL5 (*INSL5*, Q9Y5Q6) and relaxin-3 (*RLN3*, Q8WXF3) interact with their receptors using distinct residues in their B-chains for binding, and activation, respectively [1073, 2804].

**Table:** 

Nomenclature	RXFP1	RXFP2
HGNC, UniProt	*RXFP1*, Q9HBX9	*RXFP2*, Q8WXD0
Potency order of endogenous ligands	relaxin (*RLN2*, P04090) = relaxin-1 (*RLN1*, P04808) > relaxin-3 (*RLN3*, Q8WXF3) [2477]	INSL3 (*INSL3*, P51460) > relaxin (*RLN2*, P04090) ≫ relaxin-3 (*RLN3*, Q8WXF3) [1379, 2477]
Agonists	LY3540378 [2680], SA10SC-RLX [1103, 1629], (B7-33)H2 [569, 1059]	compound 6641 [666]
Antagonists	B-R13/17K H2 relaxin (pIC_50_ 5.7–6.7) [1062, 1880]	–
Selective antagonists	–	A(9-26)INSL3 (p*K*_i_ 9.1) [1061], A(10-24)INSL3 (p*K*_i_ 8.7) [1061], A(C10/15S)INSL3 (p*K*_i_ 8.6) [2930], INSL3 B chain dimer analogue 8 (p*K*_i_ 8.5) [2349], A(D10/15C)INSL3 (p*K*_i_ 8.3) [2930], cyclic INSL3 B-chain analogue 6 (p*K*_i_ 6.7) [2347], INSL3 B-chain analogue (p*K*_i_ 5.1) [560], (des 1–8) A-chain INSL3 analogue [314]
Labelled ligands	[^33^P]relaxin (human) (Agonist) [920, 2477], europium-labelled relaxin (Agonist) [2346], TamRLX (Agonist) [1028], Nanoluciferase-labelled relaxin (Agonist) [2820], [^125^I]relaxin (human) (Agonist)	[^125^I]INSL3 (human) (Agonist) [1819], [^33^P]relaxin (human) (Agonist) [920, 2477], europium-labelled INSL3 (Agonist) [2348], TamRLX (Agonist) [1028]

**Table:** 

Nomenclature	RXFP3	RXFP4
HGNC, UniProt	*RXFP3*, Q9NSD7	*RXFP4*, Q8TDU9
Potency order of endogenous ligands	relaxin-3 (*RLN3*, Q8WXF3) > relaxin-3 (B chain) (*RLN3*, Q8WXF3) > relaxin (RLN2, P04090) [1540]	INSL5 (*INSL5*, Q9Y5Q6) = relaxin-3 (*RLN3*, Q8WXF3) > relaxin-3 (B chain) (*RLN3*, Q8WXF3) [1538, 1539]
Agonists	compound 4 [558], B1-27 [1454], WNN0109-C011 [1523]	compound 4 [558], hINSL5: A8-21 (T15K) [1998], DC591053 [414], JK1 [1524]
Endogenous antagonists	INSL5 (INSL5, Q9Y5Q6) (p*K*_i_ 7) [2953]	–
Antagonists	R3(BΔ23-27)R/I5 chimeric peptide (pIC_50_ 9.2) [1368], R3 B1-22R (p*K*_i_ 7.7) [963]	R3(BΔ23-27)R/I5 chimeric peptide (pIC_50_ 8–8.6) [962, 1368], INSL5-A13NR (pIC_50_ 7.4) [2100]
Selective antagonists	minimised relaxin-3 analogue 3 (p*K*_i_ 7.6) [2345], R3 B1-22R (p*K*_i_ 7.4) [962]	minimised relaxin-3 analogue 3 (pIC_50_ 6.6) [2345]
Labelled ligands	[^125^I]relaxin-3 (human) (Agonist) [1540], [^125^I]relaxin-3-B/INSL5 A chimera (Agonist) [1538], europium-labelled relaxin-3-B/INSL5 A chimera (Agonist) [962], NanoLuc R3/I5 chimera (Agonist) [2728, 2729]	[^125^I]relaxin-3 (human) (Agonist) [1539], [^125^I]relaxin-3-B/INSL5 A chimera (Agonist) [1538], europium-labelled mouse INSL5 (Agonist) [169], europium-labelled relaxin-3-B/INSL5 A chimera (Agonist) [962], europium-labelled INSL5 (p*K*_d_ 8.3) [962], NanoLuc R3/I5 chimera (Agonist) [1072, 2728]

**Comments**: Relaxin (*RLN2*, P04090) is the cognate peptide ligand for RXFP1 and is a potential treatment for heart failure [614]. Relaxin has vasodilatory, anti-fibrotic, angiogenic, anti-apoptotic and anti-inflammatory effects. A small molecule allosteric agonist ML290 has been developed [2357, 2832], that displays anti-fibrotic properties [1198], and a relaxin B-chain mimetic peptide B7–33 has been developed which has cell specific signaling properties [1060]. The antifibrotic actions of relaxin are dependent on the angiotensin receptor AT_2_ [444] and are blocked by either AT_1_ or AT_2_ receptor antagonists. INSL3 (*INSL3*, P51460) is the cognate peptide for RXFP2 and is a circulating hormone that in males is essential for testicular descent *in utero* [1867] and in females has important roles in ovarian follicle function [1124]. In adults, INSL3 has potential roles in testicular function [1125] and the musculo-skeletal system [552]. RXFP2 is also present in brain, associated with cortico-thalamic motor circuits [2329]. cAMP elevation is the major signalling pathway for both RXFP1 and RXFP2 [1070, 1071], but RXFP1 also activates MAP kinases, nitric oxide signalling, and tyrosine kinase phosphorylation; and relaxin can interact with glucocorticoid receptors [921]. RXFP1 displays ultra-sensitive responses to sub picomolar levels of relaxin [464]. Receptor expression profiles suggest that RXFP3 is a brain neuropeptide receptor [1593, 1594, 2409] and RXFP4 a gut hormone receptor [762]. The brain relaxin-3/RXFP3 system modulates feeding [761, 762, 962, 2345, 2408] *via* effects in hypothalamus [542, 761, 1212, 1213], anxiety [1665, 2236, 2240, 2917], reward and motivated, goal-directed behaviours [1054, 2236, 2708], and spatial and social memory [38, 913, 914]. Of the other relaxin peptides, relaxin-3 (*RLN3*, Q8WXF3) is an agonist at RXFP3 and RXFP4 whereas INSL5 (*INSL5*, Q9Y5Q6) is an agonist at RXFP4 and a weak antagonist at RXFP3. Single chain peptide agonists and antagonists have been developed for RXFP3 [961, 1454] and a small molecular weight agonist active at RXFP3 and RXFP4 [558]. INSL5 (*INSL5*, Q9Y5Q6) is secreted from enteroendocrine L cells and the INSL5/RXFP4 system affects food intake [880], colon motility [591] and glucose homeostasis [1582]. RXFP3 and RXFP4 couple to G_i/o_ and inhibit adenylyl cyclase [1540, 2654], and also cause Erk1/2 phosphorylation [2654]. RXFP4 also causes phosphorylation of p38MAPK, Akt and S6RP [64] and GLP-1 secretion *in vitro* [63]. There is evidence that at RXFP3, relaxin (*RLN2*, P04090) is a biased ligand compared to the cognate ligand relaxin-3 (*RLN3*, Q8WXF3) [2654].

Further reading on Relaxin family peptide receptorsBathgateRA
 (2013) Relaxin family peptides and their receptors. Physiol Rev
93: 405–8023303914
10.1152/physrev.00001.2012BathgateRAD
 (2018) The relaxin receptor as a therapeutic target - perspectives from evolution and drug targeting. Pharmacol Ther
187: 114–13229458108
10.1016/j.pharmthera.2018.02.008DuXJ
 (2010) Cardiovascular effects of relaxin: from basic science to clinical therapy. Nat Rev Cardiol
7: 48–5819935741
10.1038/nrcardio.2009.198Esteban-LopezM
 (2020) Diverse functions of insulin-like 3 peptide. J Endocrinol
247: R1–R1232813485
10.1530/JOE-20-0168PMC7453995Gil-MiravetI
 (2021) Involvement of the *Nucleus Incertus* and Relaxin-3/RXFP3 Signaling System in Explicit and Implicit Memory. Front Neuroanat
15: 63792233867946
10.3389/fnana.2021.637922PMC8044989HallsML
 (2015) International Union of Basic and Clinical Pharmacology. XCV. Recent advances in the understanding of the pharmacology and biological roles of relaxin family peptide receptors 1–4, the receptors for relaxin family peptides. Pharmacol Rev
67: 389–44025761609
10.1124/pr.114.009472PMC4394689IvellR
 (2011) Relaxin family peptides in the male reproductive system–a critical appraisal. Mol Hum Reprod
17: 71–8420952422
10.1093/molehr/gaq086KlonischT. (2019) Editorial to the mini-review series on relaxin, related peptides and receptors?
Mol Cell Endocrinol
487: 130831203
10.1016/j.mce.2019.02.021SamuelCS
 (2022) Relaxin as an anti-fibrotic treatment: Perspectives, challenges and future directions. Biochem Pharmacol
197: 11488434968489
10.1016/j.bcp.2021.114884

## Somatostatin receptors

G protein-coupled receptors → Somatostatin receptors

**Overview**: Somatostatin (somatotropin release inhibiting factor) is an abundant neuropeptide, which acts on five subtypes of somatostatin receptor (SST_1_-SST_5_; **nomenclature as agreed by the NC-IUPHAR Subcommittee on Somatostatin Receptors** [899]). Activation of these receptors produces a wide range of physiological effects throughout the body including the inhibition of secretion of many hormones. Endogenous ligands for these receptors are somatostatin-14 (SRIF-14 (*SST*, P61278)) and somatostatin-28 (SRIF-28 (*SST*, P61278)). Cortistatin-14 {Mouse, Rat} has also been suggested to be an endogenous ligand for somatostatin receptors [547].

**Table:** 

Nomenclature	SST_1_ receptor	SST_2_ receptor	SST_3_ receptor	SST_4_ receptor	SST_5_ receptor
HGNC, UniProt	*SSTR1*, P30872	*SSTR2*, P30874	*SSTR3*,P32745	*SSTR4*, P31391	*SSTR5*, P35346
Agonists	pasireotide [2298]	pasireotide [2298], veldoreotide [18]	pasireotide [2298]	NNC269100 [1552], veldoreotide [18]	pasireotide [2298], veldoreotide [18]
Selective agonists	L-797,591 [2199], Des-Ala1,2,5-[D-Trp8, IAmp9]SRIF [659]	L-054,522[2868], BIM 23027[359], L-779,976[2199], octreotide[304, 1997, 2381, 2382, 2383, 2868], lanreotide [304, 1997, 2381, 2382, 2383]	L-796,778[2199]	L-803,087[2199], J-2156[656]	BIM 23052[1716, 2381, 2382, 2383], L-817,818 [2199]
Selective antagonists	SRA880 (p*K*_d_ 8–8.1) [1065]	DOTA-JR11 [373]	MK-4256 (pIC_50_ 9.2) [978], ACQ090 (p*K*_i_ 7.9) [1066]	–	S5A1 (p*K*_i_ 9.3) [680]

**Comments**: [125I]Tyr11-SRIF-14, [125I]LTT-SRIF-28, [125I]CGP 23996 and [125I]Tyr10-CST14 may be used to label somatostatin receptors nonselectively. A number of nonpeptide subtype-selective agonists have been synthesised [2199]. Octreotide and lanreotide are being used in the treatment of SST_2_-expressing neuroendocrine tumors and pasireotide for SST_5_-expressing neuroendocrine tumors. A novel peptide somatostatin analogue, veldoreotide (COR-005), has affinity for SST_2_, SST_4_ and SST_5_ receptors and is a potent inhibitor of GH secretion [2060, 2368].

Further reading on Somatostatin receptorsColaoA
 (2011) Resistance to somatostatin analogs in acromegaly. Endocr Rev
32: 247–7121123741
10.1210/er.2010-0002GüntherT
 (2018) International Union of Basic and Clinical Pharmacology. CV. Somatostatin Receptors: Structure, Function, Ligands, and New Nomenclature. Pharmacol Rev
70: 763–83530232095
10.1124/pr.117.015388PMC6148080HoyerD
 (2000) In The IUPHAR Compendium of Receptor Characterization and Classification, 2nd edn. Edited by WatsonSP, GirdlestoneD: IUPHAR Media: 354–364SchulzS
 (2014) Fine-tuning somatostatin receptor signalling by agonist-selective phosphorylation and dephosphorylation: IUPHAR Review 5. Br J Pharmacol
171: 1591–924328848
10.1111/bph.12551PMC3966740WeckbeckerG
 (2003) Opportunities in somatostatin research: biological, chemical and therapeutic aspects. Nat Rev Drug Discov
2: 999–101714654798
10.1038/nrd1255

## Succinate receptor

G protein-coupled receptors → Succinate receptor

**Overview**: **Nomenclature as recommended by NC-IUPHAR** [532]. The succinate receptor (GPR91, *SUCNR1*) is activated by the tricarboxylic acid (or Krebs) cycle intermediate succinate and other dicarboxylic acids with less clear physiological relevance such as maleate [979]. Since its pairing with its endogenous ligand in 2004, intense research has focused on the receptor-ligand pair role in various (patho)physiological processes such as regulation of renin production [979, 2594], ischemia injury [979], fibrosis [1600], retinal angiogenesis [2266], inflammation [1536, 1600], immune response [2223], obesity [1237, 1692, 2687], diabetes [1499, 2594, 2655], platelet aggregation [2441, 2545] or cancer [2119, 2818]. The succinate receptor is coupled to G_i/o_ [817, 979] and G_q/11_ protein families [979, 2194, 2610]. Although the receptor is, upon ligand addition, rapidly desensitized [1035, 2194], and in some cells internalized [979], it seems to recruit arrestins weakly [809]. The cellular activation of the succinate receptor triggers various signalling pathways such as decrease of cAMP levels, [Ca^2+^]^i^ mobilization and activation of kinases (ERK, c-Jun, Akt, Src, p38, PI3Kβ, *etc*.) [818]. The receptor is broadly expressed but is notably abundant in immune cells (M2 macrophages [1237, 2610], monocytes [2223], immature dendritic cells [2223], adipocytes [2687], platelets [2441, 2545], *etc*.) and in the kidney [979].

**Table:** 

Nomenclature	succinate receptor
HGNC, UniProt	*SUCNR1*, Q9BXA5
Endogenous agonists	succinic acid [979, 2436], maleic acid [809, 817, 979]
Agonists	compound 31 (Partial agonist) [2165], compound 130 (Partial agonist) [2611], cis-epoxysuccinic acid [809]
Antagonists	NF-56-EJ40 (p*K*_i_ 7.8) [909]

**Comments**: In humans, there is the possibility of two open-reading frames (ORFs) for *SUCNR1*, one giving a protein of 330 amino acids (AA) and the other one 334-AA. Wittenberger *et al*. [2801] noted that the 330-AA protein was more likely to be expressed given the Kozak sequence surrounding the second ATG. Some databases report SUCNR1 as being 334-AA long.

Further reading on Succinate receptorde Castro FonsecaM
 (2016) GPR91: expanding the frontiers of Krebs cycle intermediates. Cell Commun Signal
14: 326759054
10.1186/s12964-016-0126-1PMC4709936GilissenJ
 (2016) Insight into SUCNR1 (GPR91) structure and function. Pharmacol Ther
159: 56–6526808164
10.1016/j.pharmthera.2016.01.008GrimolizziF
 (2018) Multiple faces of succinate beyond metabolism in blood. Haematologica
103: 1586–159229954939
10.3324/haematol.2018.196097PMC6165802KrzakG
 (2021) Succinate Receptor 1: An Emerging Regulator of Myeloid Cell Function in Inflammation. Trends Immunol
42: 45–5833279412
10.1016/j.it.2020.11.004LückmannM
 (2020) Structural basis for GPCR signaling by small polar versus large lipid metabolites-discovery of non-metabolite ligands. Curr Opin Cell Biol
63: 38–4831951921
10.1016/j.ceb.2019.12.005

## Tachykinin receptors

G protein-coupled receptors → Tachykinin receptors

**Overview**: Tachykinin receptors (**provisional nomenclature as recommended by NC-IUPHAR** [712]) are activated by the endogenous peptides substance P (*TAC1*, P20366) (SP), neurokinin A (*TAC1*, P20366) (NKA; previously known as substance K, neurokinin α, neuromedin L), neurokinin B (*TAC3*, Q9UHF0) (NKB; previously known as neurokinin β, neuromedin K), neuro-peptide K (*TAC1*, P20366) and neuropeptide γ (*TAC1*, P20366) (N-terminally extended forms of neurokinin A). The neurokinins (A and B) are mammalian members of the tachykinin family, which includes peptides of mammalian and nonmammalian origin containing the consensus sequence: Phe-x-Gly-Leu-Met. Marked species differences in *in vitro* pharmacology exist for all three receptors, in the context of nonpeptide ligands. Antagonists such as aprepitant and fosaprepitant were approved by FDA and EMA, in combination with other antiemetic agents, for the prevention of nausea and vomiting associated with emetogenic cancer chemotherapy.

**Table:** 

Nomenclature	NK_1_ receptor	NK_2_ receptor	NK_3_ receptor
HGNC, UniProt	*TACR1*, P25103	*TACR2*, P21452	*TACR3*, P29371
Potency order of endogenous ligands	substance P (*TAC1*, P20366) > neurokinin A (*TAC1*, P20366) > neurokinin B (*TAC3*, Q9UHF0)	neurokinin A (*TAC1*, P20366) > neurokinin B (*TAC3*, Q9UHF0) ≫ substance P (*TAC1*, P20366)	neurokinin B (*TAC3*, Q9UHF0) > neurokinin A (*TAC1*, P20366) > substance P (*TAC1*, P20366)
Agonists	substance P-OMe [2580]	–	–
Selective agonists	[Sar^9^, Met(O2)^11^]SP [2580], septide [173, 958], [Pro^9^]SP [2599] – Rat	[Lys^5^, Me-Leu^9^, Nle^10^]NKA-(4-10) [1680] – Rat, GR64349 [555] – Rat, [βAla^8^]neurokinin A-(4-10) [650]	[Phe(Me)^7^]neurokinin B [2268, 2269], senktide [2268, 2269, 2580]
Antagonists	L760735 (pIC_50_ 9.7) [953]	–	–
Selective antagonists	aprepitant (p*K*_i_ 10.1) [915, 916], CP 99994 (p*K*_i_ 9.3–9.7) [66, 2269], RP67580 (pIC_50_ 7.7) [711]	GR94800 (p*K*_i_ 9.8) [264], saredutant (p*K*_i_ 9.4–9.7) [66, 650, 2269], GR 159897 (p*K*_d_ 7.8–9.5) [182, 650, 2417], MEN10627 (p*K*_i_ 9.2) [812], nepadutant (p*K*_i_ 8.5–8.7) [361, 460]	osanetant (p*K*_i_ 8.4–9.7) [66, 152, 459, 649, 1206, 1965, 2268, 2269, 2580], talnetant (p*K*_i_ 7.4–9) [176, 813, 2268, 2269], PD157672 (pIC_50_ 7.8–7.9) [219, 2580]
Labelled ligands	[^125^I]L703,606 (Antagonist) (p*K*_d_ 9.5) [723], [^125^I] BH-[Sar9, Met(O2)11]SP (Agonist) [2606] – Rat, [^3^H]SP (human, mouse, rat) (Agonist) [112], [^125^I]SP (human, mouse, rat) (Agonist), [^18^F]SPA-RQ (Antagonist) [430]	[^3^H]saredutant (Antagonist) (p*K*_d_ 9.7) [878] – Rat, [^125^I]NKA (human, mouse, rat) (Agonist) [2745], [^3^H] GR100679 (Antagonist) (p*K*_d_ 9.2) [911]	[^3^H]osanetant (Antagonist) (p*K*_d_ 9.9), [^3^H]senktide (Agonist) [889] – Guinea pig, [^125^I][MePhe7]NKB (Agonist)

**Comments**: The NK_1_ receptor has also been described to couple to G proteins other than G_q/11_ [2216]. The crystal structure of the human NK_1_ receptor in complex with antagonists has been determined [2306, 2887]. The hexapeptide agonist septide appears to bind to an overlapping but non-identical site to substance P (*TAC1*, P20366) on the NK_1_ receptor. There are additional subtypes of tachykinin receptor; an orphan receptor (SwissProt P30098) with structural similarities to the NK_3_ receptor was found to respond to NKB when expressed in *Xenopus* oocytes or Chinese hamster ovary cells [598, 1355]. NK_1_ receptor antagonists affect cellular physiology including inflammation, apoptosis and cell trafficking and have a role in therapeutics [1828, 2444]. 

Further reading on Tachykinin receptorsDouglasSD
 (2011) Neurokinin-1 receptor: functional significance in the immune system in reference to selected infections and inflammation. Ann N Y Acad Sci
1217: 83–9521091716
10.1111/j.1749-6632.2010.05826.xPMC3058850FoordSM
 (2005) International Union of Pharmacology. XLVI. G protein-coupled receptor list. Pharmacol Rev
57: 279–8815914470
10.1124/pr.57.2.5JonesS
 (2008) The neurokinin 1 receptor: a potential new target for anti-platelet therapy?
Curr Opin Pharmacol
8: 114–918296119
10.1016/j.coph.2008.01.004SteinhoffMS
 (2014) Tachykinins and their receptors: contributions to physiological control and the mechanisms of disease. Physiol Rev
94: 265–30124382888
10.1152/physrev.00031.2013PMC3929113YinJ
 (2018) Crystal structure of the human NK_1_ tachykinin receptor. Proc Natl Acad Sci USA
115: 13264–1326930538204
10.1073/pnas.1812717115PMC6310836

## Thyrotropin-releasing hormone receptors

G protein-coupled receptors → Thyrotropin-releasing hormone receptors

**Overview**: Thyrotropin-releasing hormone (TRH) receptors (**provisional nomenclature as recommended by NC-IUPHAR** [712]) are activated by the endogenous tripeptide TRH (*TRH*, P20396) (pGlu-His-ProNH2). TRH (*TRH*, P20396) and TRH analogues fail to distinguish TRH_1_ and TRH_2_ receptors [2489]. [^3^H]TRH (human, mouse, rat) is able to label both TRH_1_ and TRH_2_ receptors with K_d_ values of 13 and 9 nM respectively. Synthesis and biology of ring-modified L-Histidine containing TRH analogues has been reported [1705].

**Table:** 

Nomenclature	TRH1 receptor	TRH2 receptor
HGNC, UniProt	*TRHR*, P34981	–
Antagonists	diazepam (p*K*_i_ 5.2) [613] – Rat	–
Selective antagonists	midazolam (p*K*_i_ 5.5) [613] – Rat, chlordiazepoxide (p*K*_i_ 4.8) [613] – Rat, chlordiazepoxide (p*K*_i_ 4.7) [2469] – Mouse	–
Comments	–	A class A G protein-coupled receptor: not present in man

Further reading on Thyrotropin-releasing hormone receptorsBílekR
 (2011) TRH-like peptides. Physiol Res
60: 207–1521114375
10.33549/physiolres.932075FoordSM
 (2005) International Union of Pharmacology. XLVI. G protein-coupled receptor list. Pharmacol Rev
57: 279–8815914470
10.1124/pr.57.2.5NillniEA. (2010) Regulation of the hypothalamic thyrotropin releasing hormone (TRH) neuron by neuronal and peripheral inputs. Front Neuroendocrinol
31: 134–5620074584
10.1016/j.yfrne.2010.01.001PMC2849853

## Trace amine receptor

G protein-coupled receptors → Trace amine receptor

**Overview**: Trace amine-associated receptors were discovered from a search for novel 5-HT receptors [244], where 15 mammalian orthologues were identified and divided into two families. The TA_1_ receptor (**nomenclature as agreed by the NC-IUPHAR Subcommittee for the Trace amine receptor** [1616]) has affinity for the endogenous trace amines tyramine, β-phenylethylamine and octopamine in addition to the classical amine dopamine [244]. Emerging evidence suggests that TA_1_ is a modulator of monoaminergic activity in the brain [2835] with TA_1_ and dopamine D_2_ receptors shown to form constitutive heterodimers when co-expressed [665]. In addition to trace amines, receptors can be activated by amphetamine-like psychostimulants, and endogenous thyronamines.

**Table:** 

Nomenclature	TA_1_ receptor
HGNC, UniProt	*TAAR1*, Q96RJ0
Potency order of endogenous ligands	tyramine > β-phenylethylamine > octopamine = dopamine [244]
Agonists	RO5166017 [2164]
Antagonists	EPPTB (Inverse agonist) (pIC_50_ 5.1) [262]
Labelled ligands	[^3^H]tyramine (Agonist) [244]

**Comments**: In addition to TA_1_, in man there are up to 5 functional TAAR genes (TAAR2,5,6,8,9). See [244] for detailed discussion. The product of the gene TAAR2 (also known as GPR58) appears to respond to β-phenylethylamine > tyramine and to couple through G_s_ [244].

*TAAR3*, in some individuals, and *TAAR4* are pseudogenes in man, although functional in rodents. The signalling characteristics and pharmacology of TAAR_5_ (PNR, Putative Neurotransmitter Receptor: *TAAR5*, O14804), TAAR6 (Trace amine receptor 4, TaR-4: *TAAR6*, 96RI8), TAAR_8_ (Trace amine receptor 5, GPR102: *TAAR8*, Q969N4) and TAAR_9_ (trace amine associated receptor 9: *TAAR9*, 96RI9) are lacking. The thyronamines, endogenous derivatives of thyroid hormone, have affinity for rodent cloned trace amine receptors, including TA_1_ [2283]. An antagonist EPPTB has recently been described with a *p*K_i_ of 9.1 at the mouse TA_1_ but > 5.3 for human TA_1_ [2449].

Further reading on Trace amine receptorMaguireJJ
 (2009) International Union of Pharmacology. LXXII. Recommendations for trace amine receptor nomenclature. Pharmacol Rev
61: 1–819325074
10.1124/pr.109.001107PMC2830119PeiY
 (2016) Trace Amines and the Trace Amine-Associated Receptor 1: Pharmacology, Neurochemistry, and Clinical Implications. Front Neurosci
10: 14827092049
10.3389/fnins.2016.00148PMC4820462

## Urotensin receptor

G protein-coupled receptors → Urotensin receptor

**Overview**: The urotensin-II (U-II) receptor (UT, **nomenclature as agreed by the NC-IUPHAR Subcommittee on the Urotensin receptor** [608, 712, 2677]) is activated by the endogenous dodecapeptide urotensin-II (*UTS2*, O95399), originally isolated from the urophysis, the endocrine organ of the caudal neurosecretory system of teleost fish [185, 2676]. Several structural forms of U-II exist in fish and amphibians [2677]. The goby orthologue was used to identify U-II as the cognate ligand for the predicted receptor encoded by the rat gene *gpr14* [52, 497, 1549, 1798, 1915]. Human urotensin-II (*UTS2*, O95399), an 11-amino-acid peptide [497], retains the cyclohexapeptide sequence of goby U-II that is thought to be important in ligand binding [285, 1290, 1479]. This sequence is also conserved in the deduced amino-acid sequence of rat urotensin-II {Rat} (14 amino-acids) and mouse urotensin-II {Mouse} (14 amino-acids), although the N-terminal is more divergent from the human sequence [496]. A second endogenous ligand for the UT has been discovered in rat [2482]. This is the urotensin II-related peptide (*UTS2B*, Q765I0), an octapeptide that is derived from a different gene, but shares the C-terminal sequence (CFWKYCV) common to U-II from other species. Identical sequences to rat urotensin II-related peptide (*UTS2B*, Q765I0) are predicted for the mature mouse and human peptides [616]. UT exhibits relatively high sequence identity with somatostatin, opioid and galanin receptors [2677]. The urotensinergic system displays an unprecedented repertoire of four or five ancient UT in some vertebrate lineages and five U-II family peptides in teleost fish [2603].

**Table:** 

Nomenclature	UT receptor
HGNC, UniProt	*UTS2R*, Q9UKP6
Endogenous agonists	urotensin II-related peptide (*UTS2B*, Q765I0) [616, 2482], urotensin-II (*UTS2*, O95399) [52, 1549, 1798, 1915]
Selective agonists	[Pen^5^]U-(4-11) (human) [876], U-II-(4-11) (human) [876], [3-iodo-Tyr^6^]U-II-(4-11) (human) [1393], Urolinin [126], FL104 [1464, 1466], AC-7954 [508, 1465]
Selective antagonists	DS37001789 (pIC_50_ 9.1) [1902], RCI-0879 (pIC_50_ 9) [2539], urantide (p*K*_i_ 8.3) [1992], SR101099 (pIC_50_ 8) [1970], [Orn^5^]URP (p*K*_i_ 7.2) [579] – Rat, palosuran (pIC_50_ 7.1) [470], SB-611812 (p*K*_i_ 6.6) [2124], [Cha^6^]U-II-(4-11) (pK_i_ 6.4) [393] – Rat
Labelled ligands	[^125^I]U-II (human) (Agonist) [52, 393, 1615], [^125^I]N-biotin-[Ahx^0^, Bpa^3^]U-II (human) [592]

**Comments**: In the human vasculature, human urotensin-II (*UTS2*, O95399) elicits both vasoconstrictor (p*D*_2_ 9.3–10.1, [1615]) and vasodilator (pIC_50_ 10.3–10.4, [2460]) responses.

Further reading on Urotensin receptorFoordSM
 (2005) International Union of Pharmacology. XLVI. G protein-coupled receptor list. Pharmacol Rev
57: 279–8815914470
10.1124/pr.57.2.5HuntBD
 (2010) A rat brain atlas of urotensin-II receptor expression and a review of central urotensin-II effects. Naunyn Schmiedebergs Arch Pharmacol
382: 1–3120422157
10.1007/s00210-010-0503-zMaryanoffBE
 (2010) Urotensin-II receptor modulators as potential drugs. J Med Chem
53: 2695–70820043680
10.1021/jm901294uRossB
 (2010) Role of urotensin II in health and disease. Am J Physiol Regul Integr Comp Physiol
298: R1156–7220421634
10.1152/ajpregu.00706.2009VaudryH
 (2015) International Union of Basic and Clinical Pharmacology. XCII. Urotensin II, urotensin II-related peptide, and their receptor: from structure to function. Pharmacol Rev
67: 214–5825535277
10.1124/pr.114.009480

## Vasopressin and oxytocin receptors

G protein-coupled receptors → Vasopressin and oxytocin receptors

**Overview**: Vasopressin (AVP) and oxytocin (OT) receptors (**nomenclature as recommended by NC-IUPHAR** [712]) are activated by the endogenous cyclic nonapeptides vasopressin (*AVP*, P01185) and oxytocin (*OXT*, P01178). These peptides are derived from precursors which also produce neurophysins (neurophysin I for oxytocin; neurophysin II for vasopressin). Vasopressin and oxytocin differ at only 2 amino acids (positions 3 and 8). There are metabolites of these neuropeptides that may be biologically active [554].

**Table:** 

Nomenclature	V_1A_ receptor	V_1B_ receptor
HGNC, UniProt	*AVPR1A*, P37288	*AVPR1B*, P47901
Potency order of endogenous ligands	vasopressin (*AVP*, P01185) > oxytocin (*OXT*, P01178) [30, 420, 491, 1848, 2247, 2512, 2564]	vasopressin (*AVP*, P01185) > oxytocin (*OXT*, P01178) [30, 420, 567, 877, 1848, 2247, 2512, 2565]
Selective agonists	selepressin [1414], F180[62, 491]	d[Leu^4^]LVP [2014], d[Cha^4^]AVP [567, 877]
Antagonists	conivaptan (p*K*_i_ 8.2–8.4) [2512, 2513]	nelivaptan (p*K*_i_ 8.4–9.3) [877, 2341]
Selective antagonists	relcovaptan (p*K*_i_ 8.1–9.3) [30, 491, 877, 2512, 2564], d(CH2)5[Tyr(Me)2, Arg8]VP (pK_i_ 9)	–
Labelled ligands	[^125^I]OH-LVA (Antagonist) (p*K*_d_ 10.3–10.4) [432, 491], [^3^H]AVP (human, mouse, rat) (Agonist) [275, 432, 2512, 2513, 2564], [^3^H]d(CH2)5[Tyr(Me)2]AVP (Antagonist) (p*K*_d_ 9)	[^3^H]AVP (human, mouse, rat) (Agonist) [567, 2247, 2512, 2513, 2565]

**Table:** 

Nomenclature	V_2_ receptor	OT receptor
HGNC, UniProt	*AVPR2*, P30518	*OXTR*, P30559
Potency order of endogenous ligands	vasopressin (*AVP*, P01185) > oxytocin (*OXT*, P01178) [30, 420, 432, 2014, 2339, 2512, 2854]	oxytocin (*OXT*, P01178) > vasopressin (*AVP*, P01185) [30, 432, 433, 462, 877, 1153, 1285]
Selective agonists	VNA932 [675], OPC-51803 [1848], d[Val^4^, DArg^8^]VP	[Thr^4^, Gly^7^]OT [323, 433, 645, 1153]
Antagonists	–	L-371,257 (p*K*_i_ 8.8) [877]
Selective antagonists	conivaptan (p*K*_i_ 9.4) [507], tolvaptan (p*K*_i_ 9.4) [2854], satavaptan (p*K*_i_ 8.4–9.3) [30, 492, 2338, 2339, 2512], lixivaptan (Inverse agonist) (p*K*_i_ 8.9–9.2) [40, 2339], d(CH_2_)_5_[D-Ile^2^, Ile^4^]AVP (p*K*_i_ 6.9–8.4) [492, 2339], mozavaptan (Inverse agonist) (p*K*_i_ 7.4–8.1) [492, 2339, 2512, 2565, 2854]	retosiban (p*K*_i_ 9–9.2) [1511, 1688], SSR126768A (p*K*_i_ 8.8–9.1) [2340], es-GlyNH_2_-d(CH_2_)_5_[Tyr(Me)^2^, Thr^4^, Orn^8^]OT (p*K*_i_ 8.5), L-372662 (p*K*_i_ 8.4) [170]
Labelled ligands	[^3^H]AVP (human, mouse, rat) (Agonist) [492, 1848, 2512, 2513, 2854], [^3^H]dDAVP (Agonist) [1651], [^3^H]desGly-NH_2_[D-Ile^2^, Ile^4^]VP (p*K*_d_ 8.6)	[^125^I]d(CH_2_)_5_[Tyr(Me)^2^, Thr^4^, Orn^8^, Tyr-NH_2_^9^]OVT (Antagonist) (p*K*_d_ 10), [^3^H]OT (human, mouse, rat) (Agonist) [432, 743, 1153, 1285], [^111^In]DOTA-dLVT (p*K*_d_ 8.3) [431]

**Comments**: Vasopressin and OT receptors have a characteristic and sometimes overlapping distribution in a number of tissues including brain. There are phylogenetic, ontogenetic and sex-specific differences in the levels and distribution of these receptors, particularly in the brain. The V_2_ receptor exhibits marked species differences, such that many ligands (d(CH_2_)_5_[D-Ile^2^, Ile^4^]AVP and [^3^H]desGly-NH_2_[D-Ile^2^, Ile^4^]VP) exhibit low affinity at human V_2_ receptors [35]. Similarly, desmopressin (dDAVP) is more V_2_ selective in the rat than in the human [2247]. The gene encoding the V_2_ receptor is polymorphic in man, underlying nephrogenic diabetes insipidus [200]. Agonist d[Cha^4^]AVP is selective only for the human and bovine V_1B_ receptors [567], while d[Leu^4^]LVP has high affinity for the rat V_1B _receptor [2014]. There are a group of V_1B_ receptor antagonists - TASP0233278, TASP0380325 and TASP0434299 that exhibit good selectivity profiles (human, rat) [1101, 1320]. Agonist and antagonist selectivity for the OT receptor may vary in different species, as shown for Thr^4^Gly^7^OT in mice, rats and humans [323]. Knockouts of vasopressin and OT receptors have system-specific defects (*e.g*., impaired ability to concentrate urine in V_2_ receptor knockouts) which include behavioural deficits (principally in V_1A_^, V^_1B_ and OT receptor knockouts).

Further reading on Vasopressin and oxytocin receptorsCarterCS
 (2020) Is Oxytocin “Nature’s Medicine”?
Pharmacol Rev
72: 829–86132912963
10.1124/pr.120.019398PMC7495339GulliverD
 (2019) Targeting the Oxytocin System: New Pharmacotherapeutic Approaches. Trends Pharmacol Sci
40: 22–3730509888
10.1016/j.tips.2018.11.001KnepperMA. (2012) Systems biology in physiology: the vasopressin signaling network in kidney. Am J Physiol, Cell Physiol
303: C1115–2422932685
10.1152/ajpcell.00270.2012PMC3530773KoshimizuTA
 (2012) Vasopressin V1a and V1b receptors: from molecules to physiological systems. Physiol Rev
92: 1813–6423073632
10.1152/physrev.00035.2011ManningM
 (2012) Oxytocin and vasopressin agonists and antagonists as research tools and potential therapeutics. J Neuroendocrinol
24: 609–2822375852
10.1111/j.1365-2826.2012.02303.xPMC3490377RigneyN
 (2022) Oxytocin, Vasopressin, and Social Behavior: From Neural Circuits to Clinical Opportunities. Endocrinology
163:10.1210/endocr/bqac111PMC933727235863332RussellJA. (2018) Fifty Years of Advances in Neuroendocrinology. Brain Neurosci Adv
2: 239821281881201432166160
10.1177/2398212818812014PMC7058251

## VIP and PACAP receptors

G protein-coupled receptors → VIP and PACAP receptors

**Overview**: Vasoactive intestinal peptide (VIP) and pituitary adenylate cyclase-activating peptide (PACAP) receptors (**nomenclature as agreed by the NC-IUPHAR Subcommittee on Vasoactive Intestinal Peptide Receptors** [950, 951]) are activated by the endogenous peptides VIP (*VIP*, P01282), PACAP-38 (*ADCYAP1*, P18509), PACAP-27 (*ADCYAP1*, P18509), peptide histidine isoleucineamide (PHI {Mouse, Rat}), peptide histidine methionineamide (PHM (*VIP*, P01282)) and peptide histidine valine (PHV (*VIP*, P01282)). VPAC_1_ and VPAC_2_ receptors display comparable affinity for the PACAP peptides, PACAP-27 (*ADCYAP1*, P18509) and PACAP-38 (*ADCYAP1*, P18509), and VIP (*VIP*, P01282), whereas PACAP-27 (*ADCYAP1*, P18509) and PACAP-38 (*ADCYAP1*, P18509) are > 100 fold more potent than VIP (*VIP*, P01282) as agonists of most isoforms of the PAC_1_ receptor. However, one splice variant of the human PAC_1_ receptor has been reported to respond to PACAP-38 (*ADCYAP1*, P18509), PACAP-27 (*ADCYAP1*, P18509) and VIP (*VIP*, P01282) with comparable affinity [529]. PG 99-465 [1789] has been used as a selective VPAC_2_ receptor antagonist in a number of physiological studies, but has been reported to have significant activity at VPAC_1_ and PAC_1_ receptors [581]. The selective PAC_1_ receptor agonist maxadilan, was extracted from the salivary glands of sand flies (*Lutzomyia longipalpis*) and has no sequence homology to VIP (*VIP*, P01282) or the PACAP peptides [1805]. Two deletion variants of maxadilan, M65 [2624] and Max.d.4 [1806] have been reported to be PAC_1_ receptor antagonists, but these peptides have not been extensively characterised.

**Table:** 

Nomenclature	PAC1 receptor	VPAC1 receptor	VPAC2 receptor
HGNC, UniProt	*ADCYAP1R1*, P41586	*VIPR1*, P32241	*VIPR2*,P41587
Potency order of endogenous ligands	PACAP-27 (*ADCYAP1*,P18509), PACAP-38 (*ADCYAP1*,P18509) ≫ VIP (VIP, P01282)	VIP (*VIP*, P01282), PACAP-27 (*ADCYAP1*, P18509), PACAP-38 (*ADCYAP1*, P18509) ≫ GHRH (*GHRH*, P01286), PHI {Pig}, secretin (*SCT*, P09683)	VIP (*VIP*, P01282), PACAP-38 (*ADCYAP1*, P18509), PACAP-27 (*ADCYAP1*, P18509) > PHI {Pig} ≫ GHRH (*GHRH*, P01286), secretin (*SCT*, P09683)
Selective agonists	maxadilan [581], maxadilan [581]	[Lys15, Arg16, Leu27]VIP-(1-7)/GRF-(8-27)-NH2 [1781], [Ala11,22,28]VIP [1892]	Ro 25-1553 [860, 1193, 1781], Ro 25-1392 [2828]
Selective antagonists	–	PG 97-269 (pIC_50_ 8.7) [859, 1193]	–
Labelled ligands	[^125^I]PACAP-27 (Agonist) [2055]	[^125^I]VIP (human, mouse, rat) (Agonist) [1892], [^125^I] PACAP-27 (Agonist)	[^125^I]VIP (human, mouse, rat) (Agonist) [1892], [^125^I] PACAP-27 (Agonist)

**Comments**: Subtypes of PAC_1_ receptors have been proposed based on tissue differences in the potencies of PACAP-27 (*ADCYAP1*, P18509) and PACAP-38 (*ADCYAP1*, P18509); these might result from differences in G protein coupling and second messenger mechanisms [2659], or from alternative splicing of PAC_1_ receptor mRNA [2442].

Further reading on VIP and PACAP receptorsHarmarAJ
 (1998) International Union of Pharmacology. XVIII. Nomenclature of receptors for vasoactive intestinal peptide and pituitary adenylate cyclase-activating polypeptide. Pharmacol Rev
50: 265–709647867
PMC6721840HarmarAJ
 (2012) Pharmacology and functions of receptors for vasoactive intestinal peptide and pituitary adenylate cyclase-activating polypeptide: IUPHAR review 1. Br J Pharmacol
166: 4–1722289055
10.1111/j.1476-5381.2012.01871.xPMC3415633ReglodiD
 (2012) Effects of pituitary adenylate cyclase activating polypeptide in the urinary system, with special emphasis on its protective effects in the kidney. Neuropeptides
46: 61–7021621841
10.1016/j.npep.2011.05.001SmithCB
 (2012) Is PACAP the major neurotransmitter for stress transduction at the adrenomedullary synapse?
J Mol Neurosci
48: 403–1222610912
10.1007/s12031-012-9749-xPMC4180436

Further reading on G protein-coupled receptorsKenakinT. (2018) Is the Quest for Signaling Bias Worth the Effort?
Mol Pharmacol
93: 266–26929348268
10.1124/mol.117.111187MichelMC
 (2018) Biased Agonism in Drug Discovery-Is It Too Soon to Choose a Path?
Mol Pharmacol
93: 259–26529326242
10.1124/mol.117.110890RothBL
 (2017) Discovery of new GPCR ligands to illuminate new biology. Nat Chem Biol
13: 1143–115129045379
10.1038/nchembio.2490PMC5835362SriramK
 (2018) G Protein-Coupled Receptors as Targets for Approved Drugs: How Many Targets and How Many Drugs?
Mol Pharmacol
93: 251–25829298813
10.1124/mol.117.111062PMC5820538

## Figures and Tables

**Table 1:** Class A orphan GPCRs with putative endogenous ligands

*GPR3*	*GPR4*	*GPR6*	*GPR12*	*GPR15*	*GPR17*	*GPR20*
*GPR22*	*GPR26*	*GPR31*	*GPR34*	*GPR35*	*GPR37*	*GPR39*
*GPR50*	*GPR63*	*GPR65*	*GPR68*	*GPR75*	*GPR84*	*GPR87*
*GPR88*	*GPR132*	*GPR149*	*GPR161*	*GPR183*	*LGR4*	*LGR5*
*LGR6*	*MAS1*	*MRGPRD*	*MRGPRX1*	*MRGPRX2*	*P2RY10*	*TAAR2*
